# Probing the substrate specificity of *Trypanosoma brucei* GlcNAc-PI de-*N*-acetylase with synthetic substrate analogues†
†Electronic supplementary information (ESI) available: Additional experimental procedures and characterisation data for the β-anomers **8** and **10** plus ^1^H and ^13^C NMR spectra of all the compounds. See DOI: 10.1039/c3ob42164c



**DOI:** 10.1039/c3ob42164c

**Published:** 2014-02-12

**Authors:** Amy S. Capes, Arthur Crossman, Michael D. Urbaniak, Sophie H. Gilbert, Michael A. J. Ferguson, Ian H. Gilbert

**Affiliations:** a Division of Biological Chemistry and Drug Discovery , College of Life Sciences , University of Dundee , Dow Street , Dundee , DD1 5EH , UK . Email: m.a.j.ferguson@dundee.ac.uk ; Email: i.h.gilbert@dundee.ac.uk ; Tel: +44 (0) 1382 386240

## Abstract

A series of synthetic analogues of 1-d-(2-amino-2-deoxy-α-d-glucopyranosyl)-*myo*-inositol 1-(1,2-di-*O*-hexadecanoyl-*sn*-glycerol 3-phosphate), consisting of 7 variants of either the d-*myo*-inositol, d-Glc*p*N or the phospholipid components, were prepared and tested as substrates and inhibitors of GlcNAc-PI de-*N*-acetylase, a genetically validated drug target enzyme responsible for the second step in the glycosylphosphatidylinositol (GPI) biosynthetic pathway of *Trypanosoma brucei*. The d-*myo*-inositol in the physiological substrate was successfully replaced by cyclohexanediol and is still a substrate for *T. brucei* GlcNAc-PI de-*N*-acetylase. However, this compound became sensitive to the stereochemistry of the glycoside linkage (the β-anomer was neither substrate or inhibitor) and the structure of the lipid moiety (the hexadecyl derivatives were inhibitors). Chemistry was successfully developed to replace the phosphate with a sulphonamide, but the compound was neither a substrate or an inhibitor, confirming the importance of the phosphate for molecular recognition. We also replaced the glucosamine by an acyclic analogue, but this also was inactive, both as a substrate and inhibitor. These findings add significantly to our understanding of substrate and inhibitor binding to the GlcNAc-PI de-*N*-acetylase enzyme and will have a bearing on the design of future inhibitors.

## Abstract 2

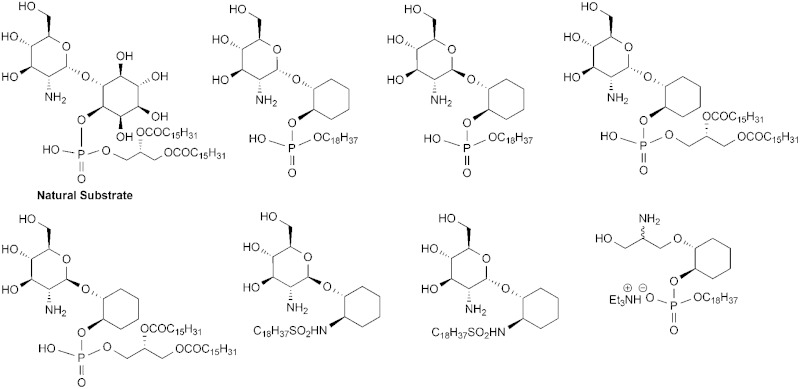
A series of substrates analogues of GlcNAc-PI de-*N*-acetylase were tested as substrates and inhibitors of the *Trypanosoma brucei* enzyme.

## Introduction

**Fig. 1:**
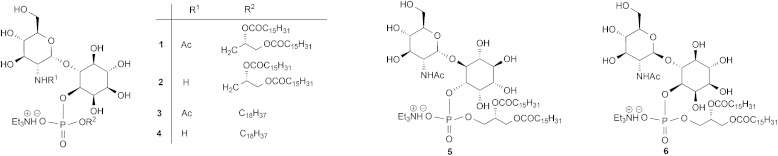
Some previously prepared GPI analogues.

The enzymes of the glycosylphosphatidylinositol (GPI) biosynthetic pathway are located in the endoplasmic reticulum, contain between one and thirteen predicted trans-membrane domains and are mostly present as components of multi-subunit complexes.^1^ No high-resolution structural data are available on any of these enzymes and our research group has been probing the specificities of several of the enzymes in the GPI pathway of the protozoan parasite *Trypanosoma brucei*, the causative agent of African sleeping sickness in humans and the related disease Nagana in cattle, using synthetic substrate analogues *in vitro*.^2–8^ One of the key enzymes of interest is an amidase, the GlcNAc-PI de-*N*-acetylase (EC 3.5.1.89) that de-*N*-acetylates 1-d-(2-acetamido-2-deoxy-α-d-glucopyranosyl)-*myo*-inositol 1-(1,2-di-*O*-hexadecanoyl-*sn*-glycerol 3-phosphate) (**1**, α-d-Glc*p*NAc-PI) to 1-d-(2-amino-2-deoxy-α-d-glucopyranosyl)-*myo*-inositol 1-(1,2-di-*O*-hexadecanoyl-*sn*-glycerol 3-phosphate) (**2**, α-d-Glc*p*N-PI), Fig. 1.

This enzyme catalyses the second step in the *T. brucei* GPI biosynthetic pathway, which is a prerequisite for all subsequent steps in the pathway.^9^ In earlier studies, we showed that *T. brucei* GlcNAc-PI de-*N*-acetylase is a zinc-dependent metalloenzyme^10^ and demonstrated, by construction of a condition-null mutant cell line, that it is essential for the bloodstream form of the parasite and, therefore, a genetically validated drug target.^11^ Previous studies with other substrate analogues showed that the phosphate, 2′-NHAc and 3′-OH groups of the natural substrate α-d-Glc*p*NAc-PI (**1**) are critical for recognition by the *T. brucei* GlcNAc-PI de-*N*-acetylase.^2–4^ In contrast, the diacylglycerol moiety is not strictly required and may be efficiently replaced with an octadecyl chain,^4^ as shown in analogues **3** and **4**. In the case of the *T. brucei* enzyme, we had hypothesised that one or more of the inositol 2, 3, 4 and 5-OH groups is/are not required.^2–4^ We came to this hypothesis from the ability of the enzyme to recognise and process both α-d-Glc*p*NAc-[L]-PI (**5**) and β-d-Glc*p*NAc-PI (**6**). Molecular dynamics simulations, showed that α and β anomers can adopt conformations in which the phosphate, the 2′-amide and the 3′-OH overlay. Given the 2′-amide is where the reaction occurs, and evidence suggests that the 3′-OH and phosphate are important for recognition of the substrate with the enzyme, these conformations are likely to be the active enzyme-bound conformations. In these conformations the inositol 2-, 3-, 4- and 5-hydroxyls are in different orientations for the two α and β anomers, implying the hydroxyls are not critical for interaction with the enzyme. Further evidence was obtained from a compound in which the inositol 2-hydroxyl was alkylated. This was also a substrate for the *T. brucei* enzyme. One of the goals of the study described in this paper was to investigate the hypothesis that the inositol 2, 3, 4 and 5-OH groups are not required.

**Fig. 2:**
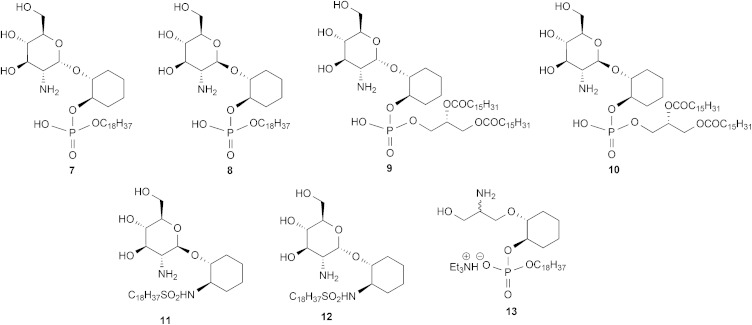
Target molecules.

Bearing in mind these key structural features, we have synthesised a variety of analogues to further probe the requirements for substrate recognition by the *T. brucei* GlcNAc-PI de-*N*-acetylase and, specifically, to test:

1. The hypothesis that the inositol 2, 3, 4 and 5-OH groups are not required for enzyme recognition, a series of pseudodisaccharides (**7–10**, Fig. 2) containing a cyclohexanediol moiety in place of the inositol aglycone were prepared.

2. Whether the phosphate group can be replaced by more cell-permeable sulphonamide isosteres,^12^ compounds **11** and **12** (Fig. 2) were prepared.

3. Whether, given the essentiality of the 2′-NHR and 3′-OH groups but non-essentiality of the 4′- and 6′-OH groups for substrate recognition, the glucosamine residue might be simplified to a simple acyclic structure, as in compound **13**.

The *N*-acetylated derivatives of the above analogues required for biological studies with the de-*N*-acetylase were prepared from the corresponding amines by standard procedures.^13^ All the analogues were examined for their recognition and processing by the *T. brucei* GlcNAc-PI de-*N*-acetylase.

## Results and discussion

### Synthesis of analogues **7–13**

**Scheme 1:**
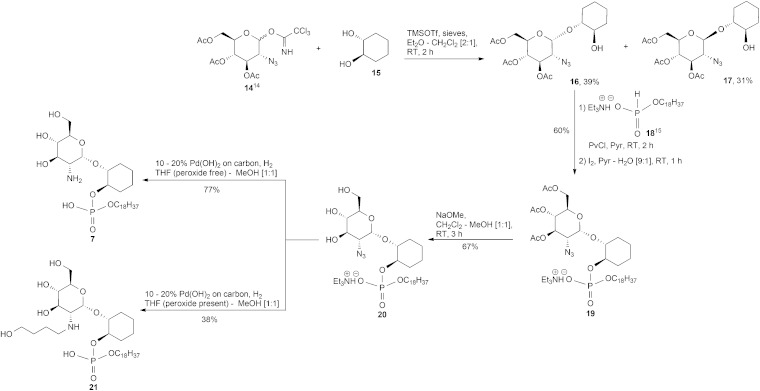
Synthesis of **7**.

The synthesis of the required α and β-glucosaminyl (1′ → 1) cyclohexanediol building blocks **16** and **17**, respectively, began by reacting the known^14^ trichloroacetimidate **14** and the commercially available 1*R*,2*R-trans*-cyclohexanediol **15**, Scheme 1, with a catalytic amount of trimethylsilyl trifluoromethanesulfonate (TMSOTf). The separation of anomers was achievable at this stage and these anomers were vital in providing the glucosamine-phosphodiester target analogues discussed herein. For the sake of brevity, we have chosen to describe in the main text the formation of the α-anomers, while details for the corresponding β-anomers appears in the ESI.† Therefore, the pseudodisaccharide **16** was coupled to the hydrogen phosphonate **18**,^15^ and the ensuing mixture of diastereoisomeric phosphonic diesters, was oxidised with iodine in pyridine–water^16^ to give the corresponding phosphodiester **19**. The diester was transformed into the triol **20** after conventional *O*-deacylation of the latter compound with 0.05 M methanolic NaOMe in CH_2_Cl_2_–MeOH.

Our first attempt at hydrogenolysis of the triethylammonium (TEA) salt **20** over Pd(OH)_2_/C gave, surprisingly, the alkyl alcohol secondary amine **21**. Initially, the azide of **20** was reduced to the primary amine, however, if there are peroxides present in the THF then the formation of THF hydroperoxide is, apparently, possible which could then lead to an amine–THF coupling *via* a free-radical-based mechanism.^17^ This intermediate is susceptible to a Pd-mediated THF ring opening reaction that gives an imine which is then further hydrogenated to an aminobutanol.^17^ Consequently, after purchasing a fresh bottle of anhydrous stabilised THF, the second hydrogenolysis attempt at **20** → **7** proceeded without incident.

**Scheme 2:**
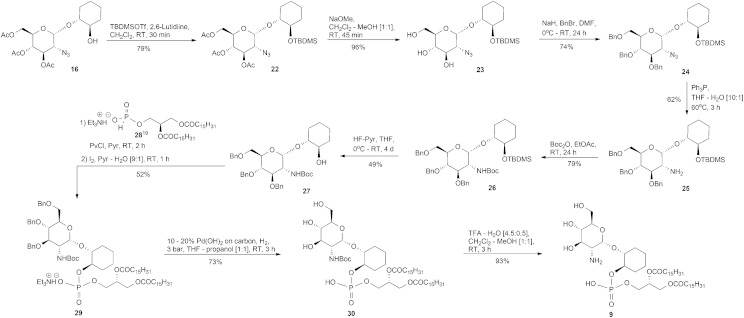
Synthesis of **9**.

The preparation of the dipalmitoyl glycerol pseudodisaccharide **9** was accomplished from the triacetate **16**, Scheme 2. However, the acetate protecting groups in **16** are unsuitable because if they were left in place and removed by base at the final step of the synthesis, then those requisite esters of the lipid fragment would likewise be saponified. Therefore, the acetates of **16** needed to be swapped to a more appropriate protecting group but first, the temporary *tert*-butyldimethylsilyl (TBDMS) protection of the 2-OH, **16** → **22**, was performed and then followed by conventional *O*-deacylation, as previously described for **19** → **20**, furnished the triol **23**.

The benzyl group was chosen as the 3′, 4′ and 6′-OH protecting group because, from our past experiences synthesising GPI analogues, the benzyl group has been a very reliable protecting group *via* ease of installation and removal. Thus, the triol **23** was benzylated with benzyl bromide in the presence of NaH, as the base, to afford compound **24**. We next turned our attention towards the reduction of the azide in **24** and because of the issues with Pd(OH)_2_ catalysed reduction in the presence of peroxidic THF discussed earlier, we chose to reduce the azide *via* the Staudinger reaction^18^ to give the amine **25** which was subsequently *tert*-butyl carbamate (Boc) protected to furnish **26**. Desilylation of **26** using HF–pyridine conditions afforded the alcohol **27**. The known hydrogen phosphonate **28** ^19^ was coupled, as already described, to the 2-OH of **27** which, after oxidation, was isolated and characterised as the TEA salt **29**. Hydrogenolysis of **29** over Pd(OH)_2_/C gave the Boc protected derivative **30** and subsequent cleavage of the Boc group produced the deprotected target analogue **9**.

**Scheme 3:**
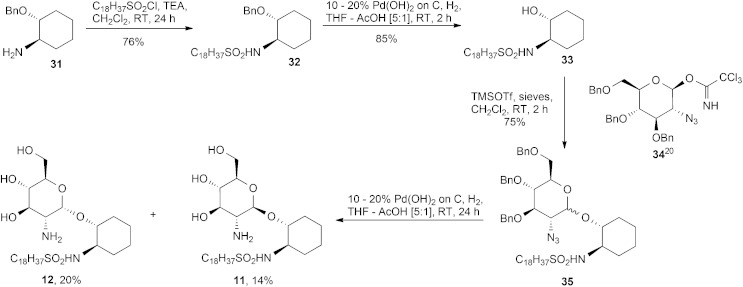
Synthesis of sulphonamides **11** and **12**.

Sulphonamides are potential isosteres for the phosphate group; they have the same tetrahedral shape and polar oxygen atoms. The synthesis of the sulphonamides **11** and **12**, Scheme 3, was accomplished by reacting commercially available 1*R*,2*R*-1-amino-2-benzyloxycyclohexane **31** with 1-octadecanesulfonyl chloride in the presence of triethylamine and CH_2_Cl_2_ to give the benzyl derivative **32**. The benzyl group was removed by hydrogenolysis over Pd(OH)_2_/C to furnish the alcohol **33**. Coupling of **33** with the known trichloroacetimidate **34** ^20^ resulted in an inseparable mixture of the α,β-anomers **35** in the ratio of ∼1 : 1, as determined by ^1^H NMR spectroscopy. Finally, hydrogenolysis of the aforementioned anomers **35** over Pd(OH)_2_/C and subsequent silica gel column chromatography (9 : 1 CH_2_Cl_2_–MeOH) gave first the β-anomer **11** and then the α-anomer **12**.

**Scheme 4:**
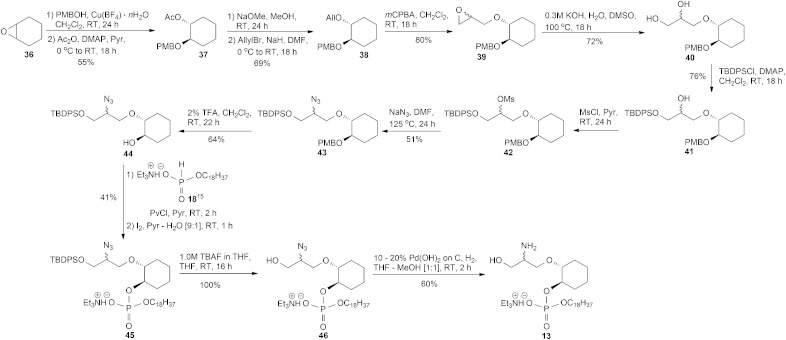
Synthesis of racemic **13**.

We were also interested in seeing if we could replace the glucose ring with an acyclic moiety. From our knowledge of the SAR, retaining the 2-amino and 3-hydroxy groups are important for activity. The synthesis of the amino-phosphate **13**, Scheme 4, began by epoxide ring-opening of cyclohexene oxide **36** with *p*-methoxybenzyl alcohol (PMBOH), using Cu(BF_4_)_2_·*n*H_2_O as a catalyst^21^ to give the known racemic PMB monoprotected cyclohexanediol^22^ contaminated with unreacted PMBOH. Chromatographic separation of this PMB cyclohexyl derivative from the excess of PMBOH was not achievable, in our hands, and so the entire PMB reaction residue was acetylated with acetic anhydride in the presence of pyridine and a catalytic amount of 4-(dimethylamino)pyridine DMAP to furnish the acetate **37**, which was easily separated from the acetate of *p*-methoxybenzyl alcohol by silica gel column chromatography.^23^ After deacetylation, the resulting alcohol^22^ residue was alkylated using sodium hydride and allyl bromide to give the allyl derivative **38**. The epoxide **39** was prepared upon reacting **38** with 3-chloroperbenzoic acid (*m*CPBA), and the subsequent hydrolysis of epoxide **39** with DMSO, H_2_O and a catalytic amount of KOH,^24^ worked smoothly to furnish diol **40**. The primary alcohol of **40** was protected using *tert*-butyl(chloro)diphenylsilane (TBDPSCl) and DMAP to give compound **41**. The azido group of **43** was satisfactorily installed (51% yield over two steps) *via* the mesylate **42** obtained by reacting the secondary alcohol **41** with methanesulfonyl chloride in the presence of pyridine, followed by treatment of **42** with sodium azide under forcing conditions. The crude mesylate **42** was used directly in the displacement reaction but a small portion of **42** was purified for a full characterisation of this intermediate. The *p*-methoxybenzyl protecting group of **43** was removed with mild acid to give alcohol **44** and then it was phosphorylated, as previously described, to give the phosphoric diester **45**. Thereafter, the removal of the silyl protecting group of **45** with 1.0 M tetrabutylammonium fluoride (TBAF) in THF proceeded smoothly to give **46** which, after hydrogenolysis over Pd(OH)_2_/C, provided the TEA salt **13**.

## Biological results

### Substrate analogues

**Fig. 3:**
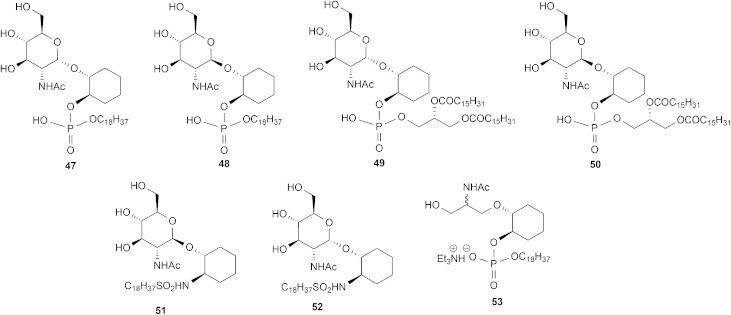
*N*-Acetylated analogues.

The ability of the *T. brucei* GlcNAc-PI de-*N*-acetylase to recognise and process the synthetic pseudodisaccharides **7–13** was tested in *T. brucei* cell-free system using an LC-MS/MS assay.^8,10^ The *N*-acetylated analogues of these compounds were prepared as previously described to give compounds **47–53** (Fig. 3).^13^

Using LC-MS/MS, multiple reaction monitoring of characteristic transitions for the *N*-acetylated and corresponding amine form of each compound was used to directly measure the rate of conversion of the *N*-acetylated compound to the free amine (Table 1). As no suitable transition was identified for the amine form of **13**, the enzymatic turnover was accessed by reacting any free amine formed with *d*_6_-Ac_2_O, and measuring the formation of the *d*_3_-*N*-acetylated form by LC-MS/MS.

**Table 1:** Recognition of synthetic analogues by *T. brucei* GlcNAc-PI de-*N*-acetylase

Compound	*m*/*z* Transition for NH_2_	*m*/*z* Transition for NHAc	Fragment assignment	Turnover/pmol/10^6^ cells equiv.	Relative turnover^*a*^
**1**	1012 > 241	972 > 241	C_6_H_10_O_8_P	6.1 ± 0.9	100%
**3**	673 > 223	715 > 223	C_6_H_8_O_7_P	27.0 ± 6.0	450%
**47**	608 > 100	650 > 100	C_6_H_12_O	ND	—
**48**	608 > 100	650 > 100	C_6_H_12_O	ND	—
**49**	906 > 255	948 > 255	O_2_CC_15_H_31_	1.3 ± 0.3	22%
**50**	906 > 255	948 > 255	O_2_CC_15_H_31_	ND	—
**51**	633 > 332	592 > 332	NHSO_2_C_18_H_37_	ND	—
**52**	633 > 332	592 > 332	NHSO_2_C_18_H_37_	ND	—
**53** ^*b*^		563 > 447	C_24_H_47_O_5_P	ND	—

^*a*^Turnover relative to α-d-Glc*p*NAc-PI (**1**).

^*b*^No suitable MRM, ND – turnover not detected. The multiple reaction monitoring (MRM) transition is shown as [parent ion *m*/*z*] > [daughter ion *m*/*z*].

Over the range of enzyme concentration that gave a linear turnover for α-d-Glc*p*NAc-PI (**1**) the substrate analogue α-d-Glc*p*NAc-I*P*C_18_ (**3**) was de-*N*-acetylated at 450% the rate of α-d-Glc*p*NAc-PI (**1**). The increased turnover is most likely due to improved accessibility of the compound, conferred by the single alkyl chain, to the membranes that contain the de-*N*-acetylase enzyme in the cell-free system. Of the synthetic pseudodisaccharides (**47–50**) tested, only the α-anomer of the dipalmitoylated compound **49** showed any appreciable turnover at 22% the rate of α-d-Glc*p*NAc-PI (**1**).

### Inhibitors

**Fig. 4:**
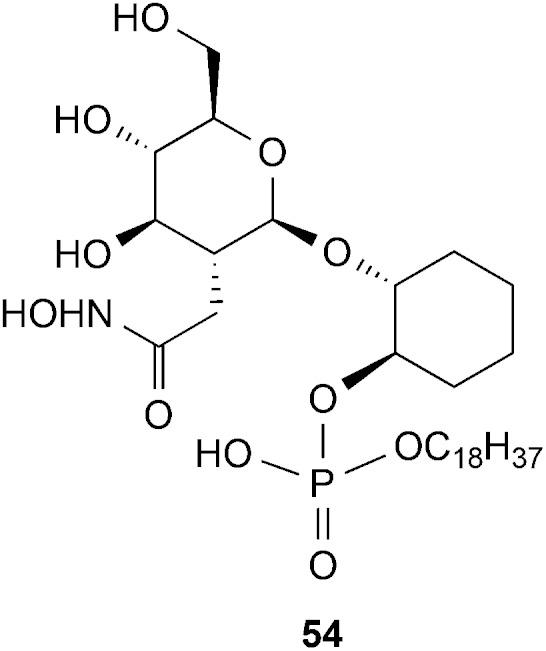
The hydroxamic acid derivative.

Since the majority of the compounds were not processed by the *T. brucei* GlcNAc-PI de-*N*-acetylase, we tested their ability to inhibit the turnover of the α-d-Glc*p*NAc-I*P*C_18_ (**3**) substrate by the *T. brucei* GlcNAc-PI de-*N*-acetylase in the LC-MS/MS assay. Most compounds showed no inhibitory activity at 100 μM. However, compounds **47** and **48** showed significant inhibition, with IC_50_ values of 11 ± 4 μM and 37 ± 20 μM, respectively, indicating that they can be recognised but not processed by the *T. brucei* GlcNAc-PI de-*N*-acetylase. Interestingly, the potency of **47** and **48** is comparable to that observed with the, hydroxamic acid pseudodisaccharide analogue **54** (Fig. 4), where the *N*-acetyl group is replaced with the zinc-chelating hydroxamic acid (IC_50_ = 19 ± 0.5 μM)^8^ and suggests that the zinc-chelating group may not be driving the potency of the latter compound.

**Fig. 5:**
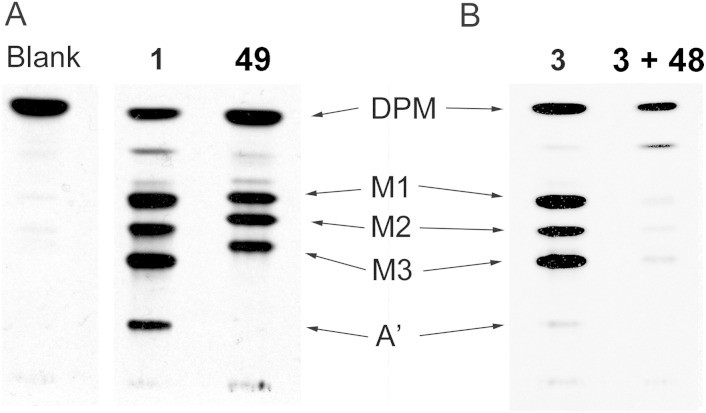
The trypanosome GPI biosynthesis in the cell-free system. A. The *T. brucei* cell-free system was incubated without exogenous substrate, with **1**, α-d-Glc*p*NAc-PI (10 μM), or with **49** (100 μM) in the presence of GDP-[^3^H]Mannose to stimulate the production of radiolabelled mannosylated GPI intermediates. B. Inhibition of the turnover of **3**, α-d-Glc*p*NAc-I*P*C_18_ (10 μM), in the presence of **48** (100 μM). Glycolipid products were extracted, separated by high-performance thin-layer chromatography, and visualised by fluorography. DPM – dolichol-phosphate-mannose, M1 – Man_1_ species, M2 – Man_2_ species, M3 – Man_3_ species, A′ – EtN*P*Man_3_ species, where the identity and migration of the species depends on the glycolipid substrate employed.

The ability of **49** and **48** to act as a substrate and inhibitor, respectively, of the *T. brucei* GPI pathway was confirmed using the trypanosome cell-free system with [^3^H]-mannose labelling (Fig. 5). Priming the cell-free system with **49** produced three bands corresponding to the addition of 1–3 mannose residues (Fig. 5A), and, consistent with this assignment, the bands were sensitive to jackbean α-mannosidase. As these mannosylated compounds lack the inositol 2-OH group they cannot undergo inositol acylation, a prerequisite for the transfer of ethanolamine, and thus are not processed past the Man_3_-species.^25^ Priming the cell-free system with **3** (α-d-Glc*p*NAc-I*P*C_18_ at 10 μM) was efficiently prevented by incubation with **48** at 100 μM (Fig. 5B), confirming that inhibition of the GlcNAc-PI de-*N*-acetylase is sufficient to prevent the formation of downstream GPI precursors.

Previous studies (see the introduction for more detail) have shown that both α-d-Glc*p*NAc-PI (**1**) and β-d-Glc*p*NAc-PI (**6**) are recognised and processed by the *T. brucei* GlcNAc-PI de-*N*-acetylase, leading to the hypothesis that one or more of the inositol 2, 3, 4, and 5-OH groups is/are not required. Our data supports this hypothesis with an important caveat; when these hydroxyl groups are removed, substrate recognition and turnover is dependent on both the stereochemistry of the glycosidic linkage and the lipid composition. With the diacylglycerol lipid containing compounds **49** and **50**, only the natural α-anomer **49** is both recognised and processed, whereas the β-anomer **50** is neither a substrate nor an inhibitor. However, neither the α nor β-anomer of the octadecyl lipid containing compounds **47** and **48** is processed, although both appear to be recognised and act as inhibitors. These sets of diastereoisomers differ only in the identity of their lipid component, with the more flexible diacylglycerol moiety allowing the glycan to be recognised and processed. Thus, it appears that the requirement for the presence of inositol 2, 3, 4, and 5-OH groups for recognition by the *T. brucei* GlcNAc-PI de-*N*-acetylase is nuanced and may depend on the conformational flexibility of the substrate analogue.

The inability of the sulphonamide-containing compounds, **51** and **52**, to act as substrates or inhibitors confirms the importance of the phosphate group in substrate recognition. It may be that the presence of the negatively charged phosphate is essential for binding at the enzyme active site.

The inactivity of compound **53** is difficult to interpret. It may be that removal of the inositol 2, 3, 4, and 5-OH groups is not compatible with having a modified glucosamine moiety, or that the entire glucosamine ring is required. Having said this, the glucosamine “replacement” in compound **53** is likely to have a considerable degree of conformational flexibility, which could allow it to take up multiple orientations within the active site.

## Conclusions

In summary, we have prepared a series of compounds to probe the substrate specificity and inhibition of enzymes involved at an early stage of GPI biosynthesis. The enzyme of interest to us, GlcNAc-PI de-*N*-acetylase, proved to be fastidious in its processing of variants of α-d-Glc*p*N-PI. We conclude that the glucosamine and the phospholipid moieties are essential for binding and that, while the d-*myo*-inositol residue is the preferred aglycone for recognition by the enzyme, the dispensing of it entirely with a cyclohexanediol group is tolerated but should be used with caution. Further work on this enzyme should focus on using the emerging structure activity relationship data to develop less synthetically complex, cell permeable analogues, which will be valuable chemical tools and may serve as leads for a drug discovery programme.

## Experimental

### Synthesis general methods

^1^H, ^13^C, ^31^P NMR spectra were recorded on a Bruker AVANCE 500 MHz spectrometer using tetramethylsilane or the residual solvent as the internal standard. High resolution electrospray ionisation mass spectra [HRMS (ESI)] were recorded with a Bruker microTof spectrometer. Melting points were determined on a Reichert hot-plate apparatus and are uncorrected. Optical rotations were measured with a Perkin-Elmer 343 polarimeter. Thin layer chromatography (TLC) was performed on Kieselgel 60 F_254_ (Merck) plates with various solvent systems as developers, followed by detection under UV light or by charring with sulfuric acid–water–ethanol (15 : 85 : 5). Column chromatography was performed on Kieselgel 60 (0.040–0.063 mm) (Merck). Radial-band chromatography (RBC) was performed using a Chromatotron (model 7924 T, TC Research UK) with silica gel F_254_ TLC standard grade as the adsorbent. Iatrobeads (6RS-8060) were purchased from SES Analysesysteme. All reactions were carried out under argon in commercially available dry solvents, unless otherwise stated.

### 1*R*,2*R*-1-*O*-(2-Azido-3,4,6-tri-*O*-acetyl-2-deoxy-α-d-glucopyranosyl)-cyclohexanediol 16 *and* the β-anomer **17**

After drying overnight over P_2_O_5_ in a vacuum desiccator, the glycosyl donor^14^ **14** (731 mg, 1.54 mmol) and the acceptor **15** (Sigma-Aldrich) were dissolved in 1 : 1 Et_2_O–CH_2_Cl_2_ (10 mL). To this solution was added activated 4 Å molecular sieves (1 g) and TMSOTf (5.4 μL, 0.03 mmol) at rt under argon. The reaction mixture was stirred at rt overnight, whereafter it was neutralised with TEA, percolated through a short column of silica gel (further elution with EtOAc) and the subsequent eluent was concentrated under reduced pressure. RBC [elution first with PE (40–60°) and then with 3 : 2 PE (40–60°)–EtOAc] of the residue gave first the α-linked pseudodisaccharide **16** (255 mg, 39%) as a waxy solid; [*α*]25D +83.7° (*c* 2.37, CHCl_3_); *δ*_H_ (500 MHz, CDCl_3_) 5.42 (dd, 1H, *J*_2′,3′_ = *J*_3′,4′_ = 10.3 Hz, H-3′), 5.09 (d, 1H, *J*_1′,2′_ = 3.7 Hz, H-1′), 4.95 (dd, 1H, *J*_3′,4′_ = *J*_4′,5′_ = 10.3 Hz, H-4′), 4.18 (dd, 1H, *J*_5′,6′a_ = 4.8, *J*_6′a,6′b_ = 12.2 Hz, H-6′a), 4.08 (m, 1H, H-5′), 4.01 (dd, 1H, *J*_5′,6′b_ = 2.2, *J*_6′a,6′b_ = 12.2 Hz, H-6′b), 3.58 (dd, 1H, H-2′), 3.44 (m, 1H, H-2), 3.23 (m, 1H, H-1), 3.05 (s, 1H, 2-OH), 2.50–1.90 (m, 11H, 3 × CH_3_, H-3a and 6a), 1.64 (m, 2H, H-4a and 5a), 1.35–1.15 (m, 4H, H-3b, 4b, 5b and 6b); *δ*_C_ (125 MHz, CDCl_3_) 170.6–169.8 (3 × *C*OCH_3_), 99.3 (C-1′), 87.5 (C-1), 74.1 (C-2), 71.7 (C-3′), 68.6 (C-4′), 67.8 (C-5′), 62.1 (C-2′), 61.9 (C-6′), 32.0, 31.7, 24.4, 23.8, 20.7 (CO*C*H_3_) 20.6 (CO*C*H_3_); HRMS (ESI) calcd for C_18_H_27_N_3_NaO_9_ [M + Na]^+^ 452.1640, found 452.1622 *and then the β-anomer* **17** (202 mg, 31%) *as a white solid*; mp 120–122 °C; [*α*]25D +21.5° (*c* 1.15, CHCl_3_); *δ*_H_ (500 MHz, CDCl_3_) 4.91 (m, 2H, H-3′ and 4′), 4.43 (d, 1H, *J*_1′,2′_ = 8.1 Hz, H-1′), 4.12 (m, 2H, H6′a and 6′b), 3.67 (m, 1H, H-5′), 3.45 (m, 1H, H-2′), 3.40–3.30 (m, 2H, H-1 and 2), 2.50–1.93 (m, 11H, 3 × CH_3_ and 2H-cyclitol), 1.70–1.60 (m, 2H, cyclitol) 1.35 (m, 1H, cyclitol), 1.17 (m, 3H, cyclitol); *δ*_C_ (125 MHz, CDCl_3_) 170.6–169.6 (3 × *C*OCH_3_), 102.1 (C-1′), 88.2, 73.2, 72.1, 71.8 (C-5′), 68.3, 63.7 (C-2′), 61.8 (C-6′), 32.2, 30.9, 24.2, 23.6, 20.7 (CO*C*H_3_), 20.6 (CO*C*H_3_); HRMS (ESI) calcd for C_18_H_28_N_3_O_9_ [M + H]^+^ 430.1820, found 430.1831.

### Triethylammonium 1*R*,2*R*-1-*O*-(2-azido-3,4,6-tri-*O*-acetyl-2-deoxy-α-d-glucopyranosyl)-cyclohexanediol 2-(*n*-octadecylphosphate) **19**

Each of the compounds **16** (117 mg, 0.27 mmol) and **18** ^15^ (237 mg, 0.54 mmol) were dried overnight over P_2_O_5_ in a vacuum desiccator, whereafter anhyd pyridine was evaporated therefrom. They were then dissolved in dry pyridine (10 mL), pivaloyl chloride (216 μL, 1.76 mmol) was added and the resulting solution was stirred under argon at rt for 1 h. A freshly prepared solution of iodine (274 mg, 1.08 mmol) in 9 : 1 pyridine–water was then added and stirring of the reaction mixture was continued for 45 min. After the addition of CH_2_Cl_2_ (20 mL), the organic solution was washed successively with 5% aq. NaHSO_3_ (25 mL), water (25 mL), 1 M TEAB buffer solution (3 × 15 mL), dried (MgSO_4_) and concentrated under reduced pressure. RBC of the residue (elution first with CH_2_Cl_2_ and then with 9 : 1 CH_2_Cl_2_–MeOH) afforded the TEA phosphate derivative **19** (140 mg, 60%); [*α*]25D +68.6° (*c* 1.07, CHCl_3_); *δ*_H_ (500 MHz, CDCl_3_) 5.40 (dd, 1H, *J*_3′,4′_ = 9.2 Hz, H-3′), 5.27 (d, 1H, *J*_1′,2′_ = 3.7 Hz, H-1′), 4.94 (t, 1H, *J*_4′,5′_ = 9.6 Hz, H-4′), 4.24–4.13 (m, 2H, H6′a and H-1 or 2), 4.07–3.98 (m, 2H, H-5′ and 6′b), 3.84–3.76 (m, 3H, OCH_2_ and H-1 or 2), 3.17 (dd, 1H, *J*_2′,3′_ = 10.6 Hz, H-2′), 2.83 (q, 6H, *J* = 6.8 Hz, 3 × C*H*_2_CH_3_), 2.05–1.95 (m, 10H, 3 × COCH_3_ and 1H-cyclitol), 1.87 (m, 1H, cyclitol), 1.62–1.45 (m, 6H, OCH_2_C*H*_2_ and 4H-cyclitol), 1.33–1.14 (41H, [CH_2_]_15_, 3 × CH_2_C*H*_3_ and 2H-cyclitol), 0.81 (t, 3H, *J* = 6.8 Hz, CH_2_C*H*_3_); *δ*_C_ (125 MHz, CDCl_3_) 169.5–168.7 (3 × *C*OCH_3_), 96.9 (C-1′), 76.4 (C-1 or C-2), 73.9 (C-1 or C-2), 69.4 (C-3′), 67.9 (C-4′), 66.7 (C-5′), 65.2 (OCH_2_), 61.1 (C-6′), 60.0 (C-2′), 44.4 [N(*C*H_2_CH_3_)_3_], 30.9, 29.7, 28.7–28.1, 24.8, 21.7, 21.0, 20.3, 19.6, 13.1 (CH_2_ *C*H_3_), 7.5 [N(CH_2_ *C*H_3_)_3_]; *δ*_P_ (202 MHz, CDCl_3_) –0.05 (with heteronuclear decoupling); HRMS (ESI) calcd for C_36_H_63_N_3_O_12_P [M – NEt_3_ – H]^–^ 760.4155, found 760.4154.

### Triethylammonium 1*R*,2*R*-1-*O*-(2-azido-2-deoxy-α-d-glucopyranosyl)-cyclohexanediol 2-(*n*-octadecylphosphate) **20**

To a solution of compound **19** (78 mg, 0.09 mmol) in 1 : 1 CH_2_Cl_2_–MeOH (10 mL) was added 5.4 M NaOMe in MeOH (0.10 mL). The mixture was kept for 3 h at rt and was then neutralised with Amberlite IR-120 (H^+^) ion-exchange resin, filtered and the filtrate concentrated under reduced pressure. Column chromatography (elution first with 3 : 1 CH_2_Cl_2_–MeOH and then with 2 : 1 → 1 : 1) of the residue furnished the TEA salt **20** (45 mg, 67%) as a waxy solid; [*α*]25D +51.6° (*c* 4.5, 1 : 1 THF–MeOH); *δ*_H_ (500 MHz, 1 : 1 CDCl_3_–MeOH-d_4_) 5.18 (d, 1H, *J*_1′,2′_ = 3.5 Hz, H-1′), 4.25 (m, 1H, H-1 or 2), 3.96–3.65 (7H, OCH_2_, H-3′, 5′, 6′a,b and H-1 or 2) 3.40 (t, 1H, *J*_3′,4′_ = *J*_4′,5′_ = 9.6 Hz, H-4′), 3.13 (q, 6H, *J* = 7.3 Hz, 3 × C*H*_2_CH_3_), 3.06 (dd, 1H, *J*_2′,3′_ = 10.5 Hz, H-2′), 2.03 (m, 1H, cyclitol), 1.90 (m, 1H, cyclitol), 1.64 (m, 6H, OCH_2_C*H*_2_ and 4H-cyclitol), 1.43–1.22 (41H, [CH_2_]_15_, 3 × CH_2_C*H*_3_ and 2H-cyclitol), 0.88 (t, 3H, *J* = 6.8 Hz, CH_2_C*H*_3_); *δ*_C_ (125 MHz, 1 : 1 CDCl_3_–MeOH-d_4_) 98.7 (C-1′), 76.3 (C-1 or C-2), 74.2 (C-1 or C-2), 73.8 72.4, 72.3, 66.9 (OCH_2_), 64.5 (C-2′), 62.7, 47.4 [N(*C*H_2_CH_3_)_3_], 32.0, 31.0–30.6, 29.8, 29.5, 27.1, 23.9, 22.5, 22.1, 15.0 (CH_2_ *C*H_3_), 9.6 [N(CH_2_ *C*H_3_)_3_]; *δ*_P_ (202 MHz, 1 : 1 CDCl_3_–MeOH-d_4_) –0.47 (with heteronuclear decoupling); HRMS (ESI) calcd for C_30_H_57_N_3_O_9_P [M – NEt_3_ – H]^–^ 634.3838, found 634.3850.

### 1*R*,2*R*-1-*O*-[2-(4-Hydroxybutyl)amino-2-deoxy-α-d-glucopyranosyl]-cyclohexanediol 2-(*n*-octadecylphosphate) **21**

A solution of the azido compound **20** (45 mg, 0.06 mmol) in 1 : 1 THF–MeOH (5 mL) containing 10–20% Pd(OH)_2_ on carbon (15 mg) was stirred under a hydrogen atmosphere at rt for 30 min before it was percolated through a short column of Chelex 100 on a bed of Celite (further elution with 1 : 1 THF–MeOH). The eluent was concentrated under reduced pressure and the ensuing residue was purified by column chromatography (elution gradient 6 : 1 → 4 : 1 CH_2_Cl_2_–MeOH) to give the hydroxybutylamino compound **21** (15 mg, 38%); [*α*]25D +34.0° (*c* 1.5, 1 : 1 CHCl_3_–MeOH); *δ*_H_ (500 MHz, 1 : 1 CDCl_3_–MeOH-d_4_) 5.43 (d, 1H, *J*_1′,2′_ = 3.6 Hz, H-1′), 4.05 (m, 1H, H-1 or 2), 3.93 (dd, 1H, *J*_3′,4′_ = 10.4 Hz, H-3′), 3.90–3.50 (8H, OCH_2_ butyl, POCH_2_, H-5′, 6′a,b and H-1 or 2), 3.38 (m, 1H, H-4′), 3.17 (t, 2H, *J* = 7.6 Hz, NCH_2_), 3.00 (dd, 1H, *J*_2′,3′_ = 10.4 Hz, H-2′), 2.18 (m, 1H, cyclitol), 2.08 (m, 1H, cyclitol), 1.86 (m, 2H, CH_2_ butyl), 1.76–1.57 (6H, POCH_2_C*H*_2_, CH_2_ butyl and 2H-cyclitol), 1.45–1.20 (34H, [CH_2_]_15_ and 4H-cyclitol), 0.89 (t, 3H, *J* = 6.8 Hz, CH_2_C*H*_3_); *δ*_C_ (125 MHz, 1 : 1 CDCl_3_–MeOH-d_4_) 97.3 (C-1′), 83.2 (C-1 or C-2), 79.3 (C-1 or C-2), 74.0, 71.8, 71.7, 67.1, 62.3, 61.6 (C-2′), 54.8, 50.6, 48.7 (NCH_2_), 34.2, 33.6, 31.9, 30.9–30.6, 27.0, 25.3, 25.2, 15.0 (CH_2_ *C*H_3_); *δ*_P_ (202 MHz, 1 : 1 CDCl_3_–MeOH-d_4_) 0.66 (with heteronuclear decoupling); HRMS (ESI) calcd for C_34_H_67_NO_10_P [M – H]^–^ 680.4508, found 680.4554.

### 1*R*,2*R*-1-*O*-(2-Amino-2-deoxy-α-d-glucopyranosyl)-cyclohexanediol 2-(*n*-octadecylphosphate) **7**

A solution of the TEA salt **20** (52 mg, 0.07 mmol) in 1 : 1 stabilised THF–MeOH (5 mL) containing 10–20% Pd(OH)_2_ on carbon (5 mg) was stirred under a hydrogen atmosphere at rt for 1 h. Work-up as described for the derivative **21** gave, after column chromatography (5 : 1 CH_2_Cl_2_–MeOH), the amino compound **7** (33 mg, 77%); [*α*]25D +57.5° (*c* 3.3, 1 : 1 CHCl_3_–MeOH); *δ*_H_ (500 MHz, 1 : 1 CDCl_3_–MeOH-d_4_), 5.42 (d, 1H, *J*_1′,2′_ = 3.9 Hz, H-1′), 4.05 (m, 1H, H-1 or 2), 3.90–3.65 (6H, OCH_2_, H-3′, 5′, 6′a,b), 3.58 (m, 1H, H-1 or 2) 3.32 (m, 3H, H-4′ and MeOH-d_4_), 3.05 (dd, 1H, *J*_2′,3′_ = 10.5 Hz, H-2′), 2.10 (m, 2H, 2H-cyclitol), 1.74–1.57 (4H, OCH_2_C*H*_2_ and 2H-cyclitol), 1.44–1.21 (34H, [CH_2_]_15_ and 4H-cyclitol), 0.90 (t, 3H, *J* = 6.8 Hz, CH_2_C*H*_3_); *δ*_C_ (125 MHz, 1 : 1 CDCl_3_–MeOH-d_4_) 97.9 (C-1′), 81.8 (C-1 or C-2), 79.9 (C-1 or C-2), 74.5, 71.8, 71.7, 66.7 (OCH_2_), 62.4 (C-6′), 55.8 (C-2′), 34.0, 33.4, 33.1, 31.9, 31.8, 30.8, 30.5, 26.9, 25.2, 25.1, 23.8, 14.5 (CH_2_ *C*H_3_); *δ*_P_ (202 MHz, 1 : 1 CDCl_3_–MeOH-d_4_) 0.54 (with heteronuclear decoupling); HRMS (ESI) calcd for C_30_H_61_NO_9_P [M + H]^+^ 610.4078, found 610.4050.

### 1*R*,2*R*-1-*O*-(2-Azido-3,4,6-tri-*O*-acetyl-2-deoxy-α-d-glucopyranosyl)-2-*O*-(*tert*-butyldimethylsilyl)-cyclohexanediol **22**

The alcohol **16** (284 mg, 0.66 mmol) was dried overnight in a desiccator over P_2_O_5_ under high vacuum and then dissolved in anhyd. CH_2_Cl_2_ (10 mL). To this solution, at room temperature, was added 2,6-lutidine (154 μL, 1.32 mmol) and *tert*-butyldimethylsilyl trifluoromethane sulfonate (228 μL, 0.99 mmol). After 30 min, CH_2_Cl_2_ (25 mL) and brine (25 mL) were added and the organic layer separated. The aqueous layer was re-extracted with CH_2_Cl_2_ (25 mL) and the combined organic layers were washed with brine (2 × 25 mL), dried (MgSO_4_) and concentrated under reduced pressure. RBC [elution first with PE (40–60°) and then with 1 : 1 PE(40–60°)–Et_2_O] of the residue gave the azide **22** (284 mg, 79%) as an oil; [*α*]25D +90.2° (*c* 1.52, CHCl_3_); *δ*_H_ (500 MHz, CDCl_3_) 5.40 (t, 1H, *J*_2′,3′_ = *J*_3′,4′_ = 9.7 Hz, H-3′), 5.35 (d, 1H, *J*_1′,2′_ = 3.3 Hz, H-1′), 5.20 (t, 1H, *J*_3′,4′_ = *J*_4′,5′_ = 9.7 Hz, H-4′), 4.26 (dd, 1H, *J*_5′,6′a_ = 4.7, *J*_6′a,6′b_ = 12.1 Hz, H-6′a), 4.10 (m, 2H, H-5′ and 6′b), 3.75 (m, 1H, H-1 or 2), 3.57 (m, 1H, H-1 or 2), 3.17 (dd, 1H, H-2′), 2.10–2.03 (3 × s, 9H, 3 × COCH_3_), 1.89 (m, 2H-cyclitol), 1.70–1.25 (6H-cyclitol), 0.89 (s, 9H, 3 × CH_3_), 0.12–0.08 (2 × s, 6H, 2 × CH_3_); *δ*_C_ (125 MHz, CDCl_3_) 170.6–169.7 (3 × *C*OCH_3_), 97.9 (C-1′), 79.7 (C-1 or 2), 72.6 (C-1 or 2), 70.4 (C-3′), 68.7 (C-4′), 67.6 (C-5′), 62.1 (C-6′), 61.0 (C-2′), 32.5, 29.8, 25.9, 22.1, 20.7 (CO*C*H_3_), 20.6 (CO*C*H_3_), 18.0, –4.1, –4.9; HRMS (ESI) calcd for C_24_H_42_N_3_O_9_Si [M + H]^+^ 544.2685, found 544.2698.

### 1*R*,2*R*-1-*O*-(2-Azido-2-deoxy-α-d-glucopyranosyl)-2-*O*-(*tert*-butyldimethylsilyl)-cyclohexanediol **23**

To a solution of the triacetate **22** (185 mg, 0.34 mmol) in 1 : 1 CH_2_Cl_2_–MeOH (92 mL) was added 5.4 M NaOMe in MeOH (230 μL). The mixture was kept for 30 min at rt and was then neutralised with Amberlite IR-120 (H^+^) ion-exchange resin, filtered and the filtrate concentrated under reduced pressure. The residue, so obtained, was percolated through a short silica-gel column (further elution with EtOAc) and the eluent was concentrated under reduced pressure to afford the triol **23** (136 mg, 96%) as a white solid, mp 122–123 °C (from 10 : 1 hexane–Et_2_O); [*α*]25D +84.1° (*c* 1.63, CHCl_3_); *δ*_H_ (500 MHz, CDCl_3_) 5.22 (d, 1H, *J*_1′,2′_ = 3.4 Hz, H-1′), 4.02 (t, 1H, *J*_3′,4′_ = 9.4 Hz, H-3′), 3.90 (dd, 1H, *J*_5′,6′a_ = 2.3, *J*_6′a,6′b_ = 11.6 Hz, H-6′a), 3.81 (dd, 1H, *J*_5′,6′b_ = 2.2, *J*_6′a,6′b_ = 11.6 Hz, H-6′b), 3.73 (m, 2H, H-5′ and H-1 or 2), 3.65 (t, 1H, *J*_4′,5′_ = 9.4 Hz, H-4′), 3.59 (m, 1H, H-1 or 2), 3.15 (dd, 1H, *J*_2′,3′_ = 10.4 Hz H-2′), 1.85 (m, 2H, cyclitol), 1.60 (m, 2H, cyclitol) 1.50–1.20 (4H, cyclitol), 0.89 (s, 9H, 3 × CH_3_), 0.12–0.08 (2 × s, 6H, 2 × CH_3_); *δ*_C_ (125 MHz, CDCl_3_) 98.2 (C-1′), 78.9 (C-1 or 2), 71.5, 71.4, 70.5 (C-4′), 62.9 (C-2′), 61.6 (C-6′), 31.9, 29.4, 25.9, 22.5, 21.7, 18.0, –4.3, –4.9; HRMS (ESI) calcd for C_18_H_36_N_3_O_6_Si [M + H]^+^ 418.2368, found 418.2365.

### 1*R*,2*R*-1-*O*-(2-Azido-3,4,6-tri-*O*-benzyl-2-deoxy-α-d-glucopyranosyl)-2-*O*-(*tert*-butyldimethylsilyl)-cyclohexanediol **24**

To a stirred and cooled (0 °C) solution of the triol **23** (70 mg, 0.17 mmol) in DMF (10 mL) under argon was added NaH (19 mg, 0.78 mmol) and the solution was stirred for 15 min before benzyl bromide (93 μL, 0.78 mmol) was added dropwise. The reaction mixture was stirred at rt overnight and then poured slowly and carefully into ice-cold water (50 mL). After dilution with EtOAc (50 mL), the EtOAc solution was washed with brine (25 mL), dried (Na_2_SO_4_) and concentrated under reduced pressure. RBC [elution gradient PE (40–60°) → 10 : 1 → 7 : 1 → 4 : 1 PE (40–60°)–Et_2_O] of the residue yielded the fully protected compound **24** (86 mg, 74%); [*α*]25D +57.9° (*c* 1.78, CHCl_3_); *δ*_H_ (500 MHz, CDCl_3_) 7.33–7.03 (15H, 3 × Ph), 5.17 (d, 1H, *J*_1′,2′_ = 3.6 Hz, H-1′), 4.84–4.37 (6H, 3 × CH_2_Ar), 3.93 (t, 1H, *J*_3′,4′_ = 9.0 Hz, H-3′), 3.83 (m, 1H, H-5′), 3.72–3.60 (m, 3H, H-4′, 6′a and 1 or 2), 3.57 (dd, 1H, *J*_5′,6′b_ = 2.0, *J*_6′a,6′b_ = 10.7 Hz, H-6′b), 3.48 (m, 1H, H-1 or 2), 3.26 (dd, 1H, *J*_2′,3′_ = 10.3 Hz, H-2′), 1.80 (m, 2H, cyclitol), 1.32 (m, 2H, cyclitol), 1.40–1.10 (4H, cyclitol), 0.82 (s, 9H, 3 × CH_3_), 0.02–0.00 (2 × s, 6H, 2 × CH_3_); *δ*_C_ (125 MHz, CDCl_3_) 137.0–136.8 (Ph), 127.4–126.7 (Ph), 97.0 (C-1′), 79.2 (C-3′), 77.9 (C-1 or 2), 77.4, 74.3, 74.1, 72.5, 71.3, 69.8 (C-5′), 67.3 (C-6′), 62.6 (C-2′), 31.3, 28.7, 24.5, 21.7, 21.0, 18.4, –5.2, –5.8; HRMS (ESI) calcd for C_39_H_54_N_3_O_6_Si [M + H]^+^ 688.3776, found 688.3780.

### 1*R*,2*R*-1-*O*-(2-Amino-3,4,6-tri-*O*-benzyl-2-deoxy-α-d-glucopyranosyl)-2-*O*-(*tert*-butyldimethylsilyl)-cyclohexanediol **25**

To a stirred solution of **24** (86 mg, 0.12 mmol) in 10 : 1 THF–water (5 mL) at 60 °C was added Ph_3_P (98 mg, 0.38 mmol). After 3 h, TLC showed the complete disappearance of the starting material. The reaction was then cooled to rt, poured into water (25 mL) and extracted with CH_2_Cl_2_ (3 × 25 mL). The combined organics were washed successively with water (25 mL), brine (25 mL), dried (MgSO_4_) and concentrated under reduced pressure. RBC [elution gradient hexane → 7 : 1 → 3 : 1 → 1 : 1 → 1 : 3 hexane–EtOAc] of the residue afforded the amino derivative **25** (53 mg, 62%); [*α*]25D +65.6° (*c* 1.09, CHCl_3_); *δ*_H_ (500 MHz, CDCl_3_) 7.35–7.09 (15H, 3 × Ph), 4.98–4.44 (7H, H-1′ and 3 × C*H*_2_Ar), 3.87 (m, 1H, H-5′), 3.75 (dd, 1H, *J*_5′,6′a_ = 3.6, *J*_6′a,6′b_ = 10.6 Hz, H-6′a), 3.61 (m, 3H, H-4′, 6′b and 1 or 2), 3.55 (t, 1H, *J*_3′,4′_ = 9.2 Hz, H-3′), 3.45 (m, 1H, H-1 or 2), 2.77 (dd, 1H, *J*_1′,2′_ = 3.7, *J*_2′,3′_ = 9.7 Hz, H-2′), 1.98 (m, 1H, cyclitol), 1.70 (m, 1H, cyclitol), 1.63–1.16 (6H, cyclitol), 0.84 (s, 9H, 3 × CH_3_), 0.00 (2 × s, 6H, 2 × CH_3_); *δ*_C_ (125 MHz, CDCl_3_) 138.7–138.0 (Ph), 128.5–127.7 (Ph), 100.2 (C-1′), 84.1 (C-3′), 79.9 (C-1 or 2), 78.9, 75.6, 74.9, 73.5, 71.4 (C-5′), 71.0, 68.7 (C-6′), 56.4 (C-2′), 31.9, 29.4, 25.9, 22.4, 21.8, 18.0, –4.3, –4.4; HRMS (ESI) calcd for C_39_H_56_NO_6_Si [M + H]^+^ 662.3871, found 662.3874.

### 1*R*,2*R*-1-*O*-[2-*N*-(*tert*-Butoxycarbonyl)amino-3,4,6-tri-*O*-benzyl-2-deoxy-α-d-glucopyranosyl]-2-*O*-(*tert*-butyldimethylsilyl)-cyclohexanediol **26**

The amine **25** (147 mg, 0.22 mmol) was dissolved in EtOAc (10 mL) at rt. Di-*tert*-butyldicarbonate (58 mg, 0.26 mmol) was then added and the mixture was stirred overnight at rt. Afterwards, the reaction mixture was diluted with EtOAc (25 mL) and then washed successively with water (25 mL), brine (25 mL), dried (Na_2_SO_4_) and concentrated under reduced pressure. RBC [elution gradient hexane → 7 : 1 → 5 : 1 hexane–EtOAc] of the residue afforded the Boc protected derivative **26** (132 mg, 79%); [*α*]25D +39.0° (*c* 1.06, CHCl_3_); *δ*_H_ (500 MHz, CDCl_3_) 7.30–7.05 (15H, 3 × Ph), 4.95 (d, 1H, *J*_1′,2′_ = 3.3 Hz, H-1′), 4.76–4.38 (7H, NH and 3 × C*H*_2_Ar), 3.90 (m, 1H, H-2′) 3.83 (m, 1H, H-5′), 3.69 (dd, 1H, *J*_5′,6′a_ = 4.1, *J*_6′a,6′b_ = 10.7 Hz, H-6′a), 3.61 (m, 3H, H-3′, 4′, and 6′b), 3.48 (m, 1H, H-1 or 2), 3.37 (m, 1H, H-1 or 2), 2.01 (m, 1H, cyclitol), 1.74 (m, 1H, cyclitol), 1.52 (m, 2H, cyclitol), 1.35 (s, 9H, 3 × BocCH_3_), 1.30–1.10 (4H, cyclitol), 0.83 (s, 9H, 3 × CH_3_), 0.00 (2 × s, 6H, 2 × CH_3_); *δ*_C_ (125 MHz, CDCl_3_) 155.3 (C=O), 138.6–138.2 (Ph), 128.4–127.5 (Ph), 99.1 (C-1′), 81.5, 81.3 (C-1 or 2), 79.5, 78.5, 75.3, 75.1, 73.4, 73.2 (C-1 or 2), 71.4 (C-5′), 68.8 (C-6′), 54.6 (C-2′), 33.4, 30.8, 28.5, 26.1, 23.3, 22.9, 18.1, –3.9, –4.3; HRMS (ESI) calcd for C_44_H_64_NO_8_Si [M + H]^+^ 762.4396, found 762.4393.

### 1*R*,2*R*-1-*O*-[2-*N*-(*tert*-Butoxycarbonyl)amino-3,4,6-tri-*O*-benzyl-2-deoxy-α-d-glucopyranosyl]-cyclohexanediol **27**

To a stirred solution of the silyl derivative **26** (114 mg, 0.15 mmol) in THF (10 mL) at 0 °C was added ∼70% HF-pyridine (90 μL). The solution was stirred overnight at rt whereafter a further aliquot of ∼70% HF-pyridine (90 μL) was added and the solution was left to stir overnight; this process was continued on day 3. On day 4, TLC revealed the complete disappearance of the starting material, whereafter satd NaHCO_3_ (1 mL) was added dropwise to quench the reaction and the resulting solution was poured into brine (25 mL) and extracted with EtOAc (3 × 25 mL). The EtOAc extracts were combined and washed with brine (25 mL), dried (MgSO_4_) and concentrated under reduced pressure. RBC [elution gradient hexane → 2 : 1 hexane–EtOAc] of the residue furnished the alcohol **27** (97 mg, 49%); [*α*]25D +27.2° (*c* 1.08, CHCl_3_); *δ*_H_ (500 MHz, CDCl_3_) 7.36–7.10 (15H, 3 × Ph), 5.12 (d, 1H, *J*_1′,2′_ = 3.6 Hz, H-1′), 4.86–4.44 (7H, NH and 3 × C*H*_2_Ar), 3.95 (dd, 1H, *J*_3′,4′_ = *J*_4′,5′_ = 9.7 Hz, H-4′), 3.86 (m, 1H, H-2′), 3.75–3.61 (4H, H-3′, 5′ and 6′a,b), 3.47 (m, 1H, H-1 or 2), 3.32 (m, 1H, H-1 or 2), 2.10–1.91 (2H, cyclitol), 1.65 (m, 2H, cyclitol), 1.43 (s, 9H, 3 × CH_3_), 1.33–1.16 (4H, cyclitol); *δ*_C_ (125 MHz, CDCl_3_) 155.6 (C=O), 138.4–138.0 (Ph), 128.4–127.7 (Ph), 99.5 (C-1′), 85.0 (C-1 or 2), 80.5, 79.7, 78.5, 75.0, 74.0 (C-1 or 2), 73.4, 71.2 (C-4′), 68.7 (C-6′), 54.9 (C-2′), 32.9, 31.7, 28.4, 24.3, 23.9; HRMS (ESI) calcd for C_38_H_50_NO_8_ [M + H]^+^ 648.3531, found 648.3527.

### Triethylammonium 1*R*,2*R*-1-*O*-[2-*N*-(*tert*-butoxycarbonyl)amino-3,4,6-tri-*O*-benzyl-2-deoxy-α-d-glucopyranosyl]-cyclohexanediol 2-(1,2-di-*O*-hexadecanoyl-*sn*-glycerol 3-phosphate) **29**

This compound was obtained from the alcohol **27** (55.7 mg, 0.086 mmol) and 1,2-di-*O*-hexadecanoyl-*sn*-glycerol 3-hydrogenphosphonate TEA salt **28** ^19^ (126 mg, 0.17 mmol) in the presence of pivaloyl chloride (69 μL, 0.56 mmol) essentially as described for the 2-(*n*-octadecyl phosphate) **19**. After the oxidation with iodine (87 mg, 0.34 mmol) in 9 : 1 pyridine–water followed by the same aqueous workup as described for **19**, RBC (elution first with CH_2_Cl_2_ and then with 20 : 1 → 15 : 1 CH_2_Cl_2_–MeOH) afforded the TEA phosphate derivative **29** (57 mg, 52%) as an opaque oil; [*α*]25D +27.7° (*c* 1.08, CHCl_3_); *δ*_H_ (500 MHz, CDCl_3_) 12.5 (brs, 1H, NH TEA salt), 7.36–7.10 (15H, 3 × Ph), 5.24 (m, 1H, H-2 glycerol), 4.96 (s, 1H, H-1′), 4.84–4.44 (6H, 3 × C*H*_2_Ar), 4.39 (m, 1H, 1- or 3-CHa glycerol), 4.17 (dd, 1H, *J* = 6.6, *J* = 12.0 Hz, 1- or 3-CHb glycerol), 4.13–3.97 (m, 4H, H-2′, 1 or 2 cyclitol and 1- or 3-CH_2_ glycerol), 3.95 (m, 1H, H-4′), 3.85 (t, *J*_2′,3′_ = *J*_3′,4′_ = 9.9 Hz, H-3′), 3.74 (dd, 1H, *J*_5′,6′a_ = 4.1, *J*_6′a,6′b_ = 10.7 Hz, H-6′a), 3.66 (m, 2H, H-5′ and 6′b), 3.50 (m, 1H, H-1 or 2), 2.97 (q, 6H, *J* = 7.1 Hz, 3 × C*H*_2_CH_3_), 2.27 (m, 4H, 2 × COCH_2_), 2.21–1.95 (2H, cyclitol), 1.72–1.50 (m, 6H, 2 × COCH_2_C*H*_2_ and 2H cyclitol), 1.44 (s, 9H, 3 × CH_3_), 1.33–1.18 (61H, 3 × CH_2_C*H*_3_, 2 × [CH_2_]_12_ and 4H cyclitol), 0.88 (t, 6H, *J* = 7.3 Hz, 2 × CH_2_C*H*_3_); *δ*_C_ (125 MHz, CDCl_3_) 172.4 (C=O), 171.9 (C=O), 155.4 (C=O), 138.9–137.4 (Ph), 127.3–126.3 (Ph), 98.9 (C-1′), 80.0, 76.8, 73.7, 72.7, 72.3, 70.3, 69.8, 69.5, 68.1, 67.6, 62.4, 61.8, 61.4, 53.5, 44.2 [N(*C*H_2_CH_3_)_3_], 33.3, 33.1, 30.9, 30.2, 28.7–28.1, 27.5, 23.4, 21.7, 13.1, 7.40 [N(CH_2_ *C*H_3_)_3_]; *δ*_P_ (202 MHz, CDCl_3_) 0.04 (with heteronuclear decoupling); HRMS (ESI) calcd for C_73_H_115_NO_15_P [M – NEt_3_ – H]^–^ 1276.8010, found 1276.8015.

### 1*R*,2*R*-1-*O*-[2-*N*-(*tert*-Butoxycarbonyl)amino-2-deoxy-α-d-glucopyranosyl]-cyclohexanediol 2-(1,2-di-*O*-hexadecanoyl-*sn*-glycerol 3-phosphate) **30**

A solution of the benzylated compound **29** (57 mg, 0.041 mmol) in 1 : 1 THF–*n*-propanol (10 mL) containing 10–20% Pd(OH)_2_ on carbon (15 mg) was stirred under 3 atm of hydrogen for 3 h before it was percolated through a short column of Chelex 100 on a bed of Celite (further elution with 1 : 1 THF–*n*-propanol). The eluent was concentrated under reduced pressure and the ensuing residue was purified by column chromatography (elution gradient 7 : 1 → 4 : 1 CH_2_Cl_2_–MeOH) to give the Boc protected derivative **30** (30 mg, 73%); [*α*]25D +39.1° (*c* 3.00, 1 : 1 CH_2_Cl_2_–MeOH); *δ*_H_ (500 MHz, 1 : 1 CDCl_3_–MeOH-d_4_) 5.25 (m, 1H, H-2 glycerol), 4.90 (s, 1H, H-1′), 4.45 (m, 1H, 1- or 3-CHa glycerol), 4.20 (dd, 1H, *J* = 6.7, *J* = 11.7 Hz, 1- or 3-CHb glycerol), 4.00 (m, 2H, 1- or 3-CH_2_ glycerol), 3.75 (m, 1H, H-3′ and 4′), 3.65 (m, 1H, H-1 or 2), 3.55 (dd, 1H, *J*_1′,2′_ = 3.0, *J*_2′,3′_ = 10.5 Hz, H-2′), 3.47 (m, 1H, H-1 or 2), 3.40 (m, 3H, H-5′ and 6′a,b), 2.40–2.00 (m, 6H, 2 × COCH_2_ and 2H cyclitol), 1.74–1.57 (m, 6H, 2 × COCH_2_C*H*_2_ and 2H cyclitol), 1.47 (s, 9H, 3 × CH_3_), 1.40–1.20 (52H, 2 × [CH_2_]_12_ and 4H cyclitol), 0.89 (t, 6H, *J* = 7.1 Hz, 2 × CH_2_C*H*_3_); *δ*_C_ (125 MHz, 1 : 1 CDCl_3_–MeOH-d_4_) 174.6 (C=O), 174.1 (C=O), 158.2 (C=O), 98.9 (C-1′), 82.0, 78.0, 73.3, 73.0, 72.0, 71.1, 64.0, 63.1, 62.0, 57.1, 56.4, 34.7, 34.5, 33.0–29.5, 28.5, 25.4, 24.3, 23.5, 23.1, 14.3; *δ*_P_ (202 MHz, 1 : 1 CDCl_3_–MeOH-d_4_) –0.28 (with heteronuclear decoupling); HRMS (ESI) calcd for C_52_H_97_NO_15_P [M – H]^–^ 1006.6601, found 1006.6635.

### 1*R*,2*R*-1-*O*-(2-Amino-2-deoxy-α-d-glucopyranosyl)-cyclohexanediol 2-(1,2-di-*O*-hexadecanoyl-*sn*-glycerol 3-phosphate) **9**

To a solution of the *tert*-butoxycarbonyl protected compound **30** (30 mg, 0.030 mmol) in 1 : 1 CH_2_Cl_2_–MeOH (1 mL) was added 9 : 1 trifluoroacetic acid (TFA)–water (5 mL). After stirring 3 h at rt, toluene (5 mL) was added and the solvents were removed under reduced pressure. Toluene (2 × 5 mL) was evaporated off twice from the residue (to remove traces of TFA and water) to give the pseudodisaccharide phosphate derivative **9** (25 mg, 93%) which did not require any further purification; [*α*]25D +28.0° (*c* 2.50, 1 : 1 CHCl_3_–MeOH); *δ*_H_ (500 MHz, 1 : 1 CDCl_3_–MeOH-d_4_) 5.28 (d, 1H, *J*_1′,2′_ = 3.7 Hz, H-1′), 5.14 (m, 1H, H-2 glycerol), 4.34 (dd, 1H, *J* = 3.2, *J* = 12.0 Hz, 1- or 3-CHa glycerol), 4.10 (dd, 1H, *J* = 6.6, *J* = 12.0 Hz, 1- or 3-CHb glycerol), 3.96 (m, 1H, H-1 or 2), 3.88 (m, 2H, 1- or 3-CH_2_ glycerol), 3.71 (m, 3H, H-3′ and 6′a,b), 3.62 (dd, 1H, *J*_4′,5′_ = 9.4, *J*_5′,6′_ = 3.9 Hz, H-5′), 3.45 (m, 1H, H-1 or 2), 3.33 (t, 1H, *J*_3′,4′_ = 9.4 Hz, H-4′), 2.96 (dd, 1H, *J*_2′,3′_ = 10.5 Hz, H-2′), 2.24 (m, 4H, 2 × COCH_2_), 2.0 (m, 2H, cyclitol), 1.68–1.47 (m, 6H, 2 × COCH_2_C*H*_2_ and 2H cyclitol), 1.35–1.14 (52H, 2 × [CH_2_]_12_ and 4H cyclitol), 0.79 (t, 6H, *J* = 7.1 Hz, 2 × CH_2_C*H*_3_); *δ*_C_ (125 MHz, 1 : 1 CDCl_3_–MeOH-d_4_) 175.3 (C=O), 174.8 (C=O), 98.0 (C-1′), 82.5 (C-1 or 2), 80.2 (C-1 or 2), 74.2 (C-5′), 71.6, 71.5, 71.4, 64.8 (CH_2_ glycerol), 63.8 (CH_2_ glycerol), 62.3 (C-6′), 55.7 (C-2′), 35.5, 35.4, 34.0, 33.5, 33.2, 31.0–30.4, 26.2, 25.3, 23.9, 15.1; *δ*_P_ (202 MHz, 1 : 1 CDCl_3_–MeOH-d_4_) 0.10 (with heteronuclear decoupling); HRMS (ESI) calcd for C_52_H_97_NO_15_P [M – H]^–^ 906.6077, found 906.6094.

### *N*-[(1*R*,2*R*)-2-(Benzyloxy)cyclohexyl]octadecane-1-sulphonamide **32**

To a solution of CH_2_Cl_2_ (10 mL) and triethylamine (2.1 mL) under argon was added (1*R*, 2*R*)-1-amino-2-benzyloxycyclohexane **31** (1.0 g, 4.87 mmol) and 1-octadecanesulfonyl chloride (2.1 g, 5.95 mmol), purchased from Sigma-Aldrich and Alfa Aesar, respectively. The reaction mixture was stirred at rt overnight, whereafter it was diluted with CH_2_Cl_2_ (40 mL), washed successively with water (25 mL), brine (25 mL), dried (MgSO_4_) and concentrated under reduced pressure. RBC (elution first with hexane and then with a gradient of 5 : 1 → 3 : 1 hexane–EtOAc) gave the sulphonamide **32** (1.9 g, 76%) as white needles; mp 63–64 °C; [*α*]25D –35.6° (*c* 1.00, CHCl_3_); *δ*_H_ (500 MHz, CDCl_3_) 7.40–7.20 (m, 5H, Ph), 4.68 (d, 1H, *J* = 1.4 Hz, OCH_2_), 4.46 (d, 1H, *J* = 5.0 Hz, NH), 3.23–3.11 (m, 2H, H1 and 2), 3.03–2.90 (m, 2H, SO_2_CH_2_), 2.23 (m, 2H, H3a and 6a), 1.80–1.60 (m, 4H, SO_2_CH_2_C*H*_2_, H4a and 5a), 1.35–1.11 (m, 34H, 15 × [CH_2_]_15_, H3b, 4b, 5b and 6b), 0.88 (t, 3H, *J* = 6.8 Hz, CH_2_C*H*_3_); *δ*_C_ (125 MHz, CDCl_3_) 138.1, 128.5, 127.8, 127.7, 80.1 (C1 or 2), 70.6 (OCH_2_), 57.6 (C1 or 2), 53.2 (SO_2_CH_2_), 33.2 (C3 or 6), 31.9, 30.1 (C3 or 6), 29.7, 29.5, 29.4, 29.1, 28.3, 24.2, 23.8, 23.5, 22.7, 14.1 (CH_2_ *C*H_3_); HRMS (ESI) calcd for C_31_H_56_NO_3_S [M + H]^+^ 522.3975, found 522.3981.

### *N*-[(1*R*,2*R*)-2-Hydroxycyclohexyl]octadecane-1-sulphonamide **33**

A solution of the benzyloxysulphonamide **32** (200 mg, 0.38 mmol) in 5 : 1 THF–AcOH (6 mL) containing 10–20% Pd(OH)_2_ on carbon (50 mg) was stirred under a slight over pressure of hydrogen at room temperature for 2 h before it was percolated through a short column of Celite on a bed of silica gel (further elution with EtOAc). The eluent was concentrated under reduced pressure to give the deprotected alcohol **33** (140 mg, 85%) as a white solid which was used without any further purification; mp 101–102 °C; [*α*]25D –7.3 (*c* 3.40, CHCl_3_); *δ*_H_ (500 MHz, CDCl_3_) 4.54 (d, 1H, *J* = 7.4 Hz, NH), 3.32 (m, 1H, H2), 3.15–3.02 (m, 3H, SO_2_CH_2_ and H1), 2.54 (brs, 1H, OH), 2.12–2.03 (m, 2H, H3a and 6a), 1.90–1.63 (m, 4H, SO_2_CH_2_C*H*_2_, H4a and 5a), 1.50–1.10 (m, 34H, [CH_2_]_15_, H3b, 4b, 5b and 6b), 0.88 (t, 3H, *J* = 6.8 Hz, CH_2_C*H*_3_); *δ*_C_ (125 MHz, CDCl_3_) 73.8 (C2), 59.8 (C1), 53.5 (SO_2_CH_2_), 34.1 (C3 or 6), 31.9, 29.7, 29.6, 29.5, 29.3, 29.1, 28.3, 24.8 (C4 or 5), 24.0 (C4 or 5), 23.7, 14.1 (CH_2_ *C*H_3_); HRMS (ESI) calcd for C_24_H_50_NO_3_S [M + H]^+^ 432.3506, found 432.3487.

### *N*-(1*R*,2*R*)-2-*O*-(2-Azido-3,4,6-tri-*O*-benzyl-2-deoxy-d-glucopyranosyl)-cyclohexyloctadecane-1-sulphonamide **35**

A mixture of trichloroacetimidate **34** ^20^ (130 mg, 0.21 mmol), acceptor **33** (109 mg, 0.25 mmol) and activated 4 Å molecular sieves (200 mg) in dry CH_2_Cl_2_ (15 mL) was stirred under argon at room temperature for 15 min. Then TMSOTf (5.3 μL, 0.029 mmol) was added and the solution was stirred at room temperature for an additional 2 h. It was then percolated through a short column of silica gel (elution with EtOAc) and the eluent was concentrated under reduced pressure. RBC (elution gradient 1 : 5 → 1 : 3 EtOAc–hexane) of the residue gave the pseudodisaccharide **35** (140 mg, 75%) as an oily mixture of α, β anomers in the ratio of ∼1 : 1, as determined by ^1^H NMR spectroscopy; *δ*_H_ (500 MHz, CDCl_3_) 7.40–7.10 (30H, 6 × Ph α and β), 5.62 (d, 1H, *J* = 2.5 Hz, NH α or β), 5.46 (s, 1H, NH α or β), 4.98 (d, 1H, *J*_1′,2′_ = 3.6 Hz, H1′α), 4.90–4.44 (m, 12H, 6 × CH_2_Ph α and β), 4.30 (d, 1H, *J*_1′,2′_ = 8.0 Hz, H1′β), 3.91 (m, 2H), 3.77–3.57 (m, 7H), 3.47 (m, 1H), 3.42–3.27 (m, 4H), 3.17 (m, 1H, H1 α/β or 2 α/β), 3.10–2.96 (m, 4H, 2 × SO_2_CH_2_), 2.37 (m, 2H, cyclitol), 2.13 (m, 2H, cyclitol), 1.90–1.18 (α and β SO_2_CH_2_C*H*_2_, [CH_2_]_15_ and H's cyclitol), 0.88 (t, 6H, *J* = 7.1 Hz, 2 × CH_2_C*H*_3_ α and β); *δ*_C_ (125 MHz, CDCl_3_) 137.9, 137.8, 137.6, 128.5–127.7 (Ph), 101.2 (C1′β), 99.6 (C1′α), 83.7, 82.7, 82.3, 81.4, 78.1, 77.5, 75.8, 75.6, 75.3, 75.1, 74.9, 73.6, 73.5, 71.4, 68.6, 68.2, 66.2, 64.7, 57.9 (C1 α/β or C2 α/β), 57.5, 53.6, 51.9, 34.0, 32.3, 32.0, 31.9, 31.4, 29.7, 29.5, 29.4, 29.3, 28.6, 28.5, 24.2, 23.8, 23.5, 23.4, 22.7, 14.2 (CH_2_ *C*H_3_ α and β); HRMS (ESI) calcd for C_51_H_76_N_4_O_7_SNa [M + Na]^+^ 911.5372, found 911.5282.

### *N*-(1*R*,2*R*)-2-*O*-(2-Amino-2-deoxy-β-d-glucopyranosyl)-cyclohexyloctadecane-1-sulphonamide 11 and the α-anomer **12**

A solution of the anomeric mixture **35** (108 mg, 0.12 mmol) in 5 : 1 THF–AcOH (6 mL) containing 10–20% Pd(OH)_2_ on carbon (25 mg) was stirred under a slight over pressure of hydrogen at room temperature for 24 h before it was filtered through a bed of Celite. The catalyst was further washed with 1 : 1 THF–MeOH (2 × 10 mL) and the washings were combined and concentrated under reduced pressure. Column chromatography (9 : 1 CH_2_Cl_2_–MeOH) gave first the β anomer **11** (9.8 mg, 14%) as a waxy solid; [*α*]25D +2.5 (*c* 0.98, MeOH); *δ*_H_ (500 MHz, MeOH-d_4_) 4.44 (d, 1H, *J*_1′,2′_ = 8.1 Hz, H1′), 3.94 (dd, 1H, *J*_5′,6′a_ = 2.1, *J*_6a′,6′b_ = 11.7 Hz, H6′a), 3.65–3.50 (m, 2H, H1 or 2 and 6′b), 3.34 (m, 2H, H3′ and 5′), 3.22 (q, 1H, *J*_3′,4′_ = *J*_4′,5′_ = 9.2 Hz, H4′), 3.09 (m, 3H, H1 or 2 and SO_2_CH_2_), 2.63 (t, 1H, *J*_2′,3′_ = 9.0 Hz, H2′), 2.13 (m, 2H, cyclitol), 1.90–1.20 (38H, SO_2_CH_2_C*H*_2_, [CH_2_]_15_ and 6H cyclitol), 0.89 (t, 3H, *J* = 7.0 Hz, CH_2_C*H*_3_); *δ*_C_ (125 MHz, MeOH-d_4_) 100.7 (C1′), 80.5 (C1 or 2), 78.5 (C3′ or 5′), 72.2 (C4′), 62.9 (C6′), 58.1 (C1 or 2 or 2′), 58.0 (C1 or 2 or 2′), 54.1 (SO_2_CH_2_), 35.4, 32.2, 30.8, 30.6, 30.5, 25.4, 25.0, 24.7, 14.5 (CH_2_ *C*H_3_); HRMS (ESI) calcd for C_30_H_61_N_2_O_7_S [M + H]^+^ 593.4194, found 593.4178. *Continued elution gave the α anomer* **12** (14.5 mg, 20%) as an oil; [*α*]25D +66.5 (*c* 1.45, MeOH); *δ*_H_ (500 MHz, MeOH-d_4_) 5.28 (d, 1H, *J*_1′,2′_ = 3.7 Hz, H1′), 3.81 (m, 1H, H6′a), 3.71 (m, 3H, H3′, 5′ and 6′b), 3.42 (m, 1H, H1 or 2), 3.34 (m, 1H, H4′), 3.23 (m, H1 or 2), 3.08 (m, 1H, H2′ and SO_2_CH_2_), 2.24 (m, 1H, cyclitol), 1.96 (m, 1H, cyclitol), 1.84–1.67 (m, 4H, SO_2_CH_2_C*H*_2_ and 2H cyclitol), 1.52–1.21 (34H, [CH_2_]_15_ and 4H cyclitol), 0.90 (t, 3H, *J* = 7.0 Hz, CH_2_C*H*_3_); *δ*_C_ (125 MHz, MeOH-d_4_) 97.3 (C1′), 81.4 (C1 or 2), 73.2 (C3′ or 5′), 70.4 (C3′ or 4′ or 5′), 70.3 (C3′ or 4′ or 5′), 60.8 (C6′), 56.6 (C1 or 2), 54.9 (C2′), 52.6 (SO_2_CH_2_), 32.7, 32.3, 31.6, 29.4, 29.3, 29.2, 29.0, 28.8, 27.8, 24.2, 23.5, 23.3, 22.3, 13.0 (CH_2_ *C*H_3_); HRMS (ESI) calcd for C_30_H_61_N_2_O_7_S [M + H]^+^ 593.4194, found 593.4181.

### *trans*-2-(4-Methoxybenzyloxy)cyclohexyl acetate **37**

Cu(BF_4_)_2_·*n*H_2_O (42 mg, 0.18 mmol) was dissolved in CH_2_Cl_2_ (20 mL) and cyclohexene oxide **36** (1.8 mL, 17.8 mmol) and 4-methoxybenzyl alcohol (10 mL, 80.2 mmol) were added. The reaction mixture was stirred for 24 h, diluted with water (20 mL), and the aqueous layer extracted with CH_2_Cl_2_ (3 × 30 mL). The combined organic extracts were washed with brine, dried over MgSO_4_, filtered and the solvent was removed *in vacuo*. The resulting crude monoprotected PMB cyclohexanediol^22^ was used with no further purification in the next step, whereby it was dissolved in pyridine (7.5 mL), cooled to 0 °C, before DMAP (3 mg, 0.26 mmol) and acetic anhydride (4.5 mL, 48.0 mmol) were added. The reaction mixture was stirred overnight at rt, diluted with water (100 mL) and extracted with EtOAc (3 × 100 mL). The combined organic extracts were washed with water (100 mL), brine (100 mL), dried over MgSO_4_, filtered and the solvent was removed under reduced pressure. Column chromatography (5 : 1 hexane–Et_2_O) of the ensuing residue afforded the oily product **37** (1.37 g, 55%); *δ*_H_ (500 MHz, CDCl_3_) 7.24 (d, 2H, *J* = 8.7 Hz, Ph), 6.86 (d, 2H, *J* = 8.6 Hz, Ph), 4.83–4.79 (m, 1H, H-1), 4.56–4.49 (2 × d, 2H, *J* = 11.7 Hz, C*H*_2_Ar), 3.80 (s, 3H, OCH_3_), 3.38–3.33 (m, 1H, H-2), 2.04 (s, 3H, CH_3_), 2.02–1.98 (m, 2H, H-3a and 6a), 1.70–1.62 (m, 2H, H-4a and 5a), 1.43–1.18 (m, 4H, H-3b, 4b, 5b and 6b); *δ*_C_ (125 MHz, CDCl_3_) 170.5 (C=O), 159.1, 131.0, 129.0, 114.0, 113.7, 78.4 (C-2), 75.3 (C-1), 71.0 (*C*H_2_Ar), 55.3 (OCH_3_), 30.0, 29.9, 23.6, 23.3, 21.4 (CH_3_); HRMS (ESI) calcd for C_16_H_22_NaO_4_ [M + Na]^+^ 301.1410, found 301.1397.

### 1-{[*trans*-2-(Allyloxy)cyclohexyloxy]methyl}-4-methoxybenzene **38**

The acetate **37** (938 mg, 3.37 mmol) was dissolved in MeOH (10 mL) and NaOMe (5.4 M in MeOH, 150 μL) was added and the solution stirred for 1 h at rt. Afterwards, TLC revealed that there was still the presence of **37** and, thus, a further aliquot of NaOMe (5.4 M in MeOH, 100 μL) was added and the reaction mixture was stirred for an additional 24 h. After which, the reaction was neutralised with Amberlite IR-120 (H^+^) ion-exchange resin, filtered and the crude solution was passed down a short plug of silica gel (elution with EtOAc) to afford the known 2-PMB protected alcohol^22^ as a pale yellow oil which was used without further purification in the next step. To a stirred and cooled (0 °C) solution of the alcohol^22^ (886 mg, 3.75 mmol) in DMF (40 mL) was added NaH (60% dispersion in mineral oil, 750 mg, 18.7 mmol) and the solution was stirred for 30 min before allyl bromide (2.92 mL, 22.8 mmol) was added dropwise. The reaction mixture was stirred under argon for a further 18 h at rt, quenched with MeOH (50 mL), H_2_O (250 mL) was added and then the resulting solution was extracted with EtOAc (3 × 250 mL). The combined organic extracts were washed with H_2_O (3 × 250 mL), brine (250 mL), dried over MgSO_4_, filtered and the solvent was removed under reduced pressure. The resulting residue was passed down a short plug of silica gel (elution with EtOAc) and, after evaporation to dryness, purified by RBC (elution gradient hexane → 5 : 1 Et_2_O–hexane) to afford the allyl product **38** (712 mg, 69%) as a clear oil; *δ*_H_ (500 MHz, CDCl_3_) 7.32 (d, 2H, *J* = 8.6 Hz, Ph), 6.90 (d, 2H, *J* = 8.7 Hz, Ph), 6.02–5.94 (m, 1H, CH_2_C*H* =CH_2_), 5.32–5.17 (4 × m, 2H, CH_2_CH=C*H*_2_), 4.63 (dd, 2H, *J* = 11.4 Hz, C*H*_2_Ar), 4.17 (m, 2H, C*H*_2_CH=CH_2_), 3.83 (s, OCH_3_), 3.37–3.29 (m, 2H, H-1 and 2), 2.03–2.00 (m, 2H, cyclitol), 1.69–1.67 (m, 2H, cyclitol), 1.39–1.20 (m, 4H, cyclitol); *δ*_C_ (125 MHz, CDCl_3_) 159.0, 135.8 (CH_2_ *C*H=CH_2_), 131.5, 129.1, 116.1 (CH_2_CH= *C*H_2_), 113.7, 81.0 (C-1 or 2), 80.8 (C-1 or 2), 71.6 (*C*H_2_CH=CH_2_), 71.0 (*C*H_2_Ar), 55.3 (OCH_3_), 30.33, 30.31, 23.59, 23.58; HRMS (ESI) calcd for C_17_H_24_NaO_3_ [M + Na]^+^ 299.1618, found 299.1607.

### 2-{[*trans*-2-((4-Methoxybenzyl)oxy)cyclohexyloxy]methyl} oxirane **39**

Compound **38** (2.22 g, 8.02 mmol) was dissolved in CH_2_Cl_2_ (30 mL) and *m*CPBA (4.15 g, 24.1 mmol) was added and the reaction mixture stirred for 18 h at rt. Afterwards, the reaction mixture was washed successively with 10% aq. sodium sulfite (80 mL), water (100 mL), 10% aq. NaOH (80 mL) and brine (80 mL). The organic phase was then filtered through cotton wool and the solvent was removed *in vacuo*. The crude material was passed down a short plug of silica gel (further elution with EtOAc) and evaporated to dryness under reduced pressure. RBC (elution gradient 1 : 1 → 2 : 1 Et_2_O–hexane) furnished the epoxide **39** (1.50 g, 64%) as a clear oil; *δ*_H_ (500 MHz, CDCl_3_) 7.20 (dd, 2H, *J* = 8.7 Hz, Ph), 6.77 (d, 2H, *J* = 8.7 Hz, Ph), 4.50 (s, 2H, C*H*_2_Ar) 3.76–3.40 (5H, OCH_3_ and 1- or 3-CH_2_ propyl), 3.25 (m, 2H, H-1 and 2), 3.05 (m, 1H, H-2 propyl), 2.67–2.52 (2H, 1- or 3-CH_2_ propyl), 1.90 (m, 2H, cyclitol), 1.57 (m, 2H, cyclitol), 1.22–1.06 (m, 4H, cyclitol); *δ*_C_ (125 MHz, CDCl_3_) 159.0, 131.4, 131.3, 129.14, 129.08, 113.7, 82.3 (C-1 or 2), 82.1 (C-1 or 2), 80.8 (C-1 or 2), 71.5 (*C*H_2_Ar), 71.2 (1- or 3-CH_2_ propyl), 70.4 (1- or 3-CH_2_ propyl), 55.2 (OCH_3_), 51.3 (C-2 propyl), 51.1 (C-2 propyl), 44.44 (1- or 3-CH_2_ propyl), 44.38 (1- or 3-CH_2_ propyl), 30.3, 30.20, 30.16, 23.6, 23.5; HRMS (ESI) calcd for C_17_H_24_NaO_4_ [M + Na]^+^ 315.1567, found 315.1553.

### 3-{[*trans*-2-((4-Methoxybenzyl)oxy)cyclohexyl]oxy}propane-1,2-diol **40**

To a solution of **39** (1.50 g, 5.13 mmol) in DMSO (56.4 mL) was added water (10.8 mL) and aq. 0.3 M KOH (2.4 mL). The reaction mixture was heated to 100 °C for 18 h, and then diluted with water (200 mL) followed by extraction with CH_2_Cl_2_ (3 × 200 mL). The combined organic extracts were washed with water (100 mL), brine (100 mL), dried over MgSO_4_, filtered and the solvent was removed under reduced pressure. The residue, so obtained, was percolated through a short column of silica gel (further elution with EtOAc) and the subsequent eluent was concentrated under reduced pressure. RBC (elution first with hexane → 6 : 1 EtOAc–hexane) of the residue afforded the diol **40** (942 mg, 65) as a clear oil; *δ*_H_ (500 MHz, CDCl_3_) 7.28 (d, 2H, *J* = 8.6 Hz, Ph), 6.88 (dd, 2H, CH, *J* = 8.6, Ph), 4.59–4.50 (2H, C*H*_2_Ar), 3.82–3.47 (8H, OCH_3_, H-2 propyl, 1- and 3-CH_2_ propyl), 3.25 (m, 2H, H-1 and 2), 2.50 (bs, 1H, OH), 2.40 (bs, 1H, OH), 2.11–2.01 (m, 2H, cyclitol), 1.68 (m, 2H, cyclitol), 1.23 (m, 4H, cyclitol); *δ*_C_ (125 MHz, CDCl_3_) 159.3, 159.2, 130.5, 130.4, 129.5, 129.4, 113.84, 113.83, 83.40 (C-1 or 2), 82.39 (CH, C-1 or 2), 80.86 (C-1 or 2), 80.78 (C-1 or 2), 72.31 (1- or 3-CH_2_ propyl), 71.37 (C-2 propyl), 71.03 (1- or 3-CH_2_ propyl), 70.81 (1- or 3-CH_2_ propyl), 70.51 (C-2 propyl), 64.20 (1- or 3-CH_2_ propyl), 63.83 (1- or 3-CH_2_ propyl), 55.24 (OCH_3_), 30.75, 30.54, 29.99, 29.93, 23.78, 23.71, 23.69; HRMS (ESI) calcd for C_17_H_26_NaO_5_ [M + Na]^+^ 333.1672, found 333.1678.

### 1-[(*tert*-Butyldiphenylsilyl)oxy]-3-{[*trans*-2-((4-methoxybenzyl)oxy)cyclohexyl]oxy}propan-2-ol **41**

To a solution of the primary alcohol **40** (942 mg, 3.34 mmol) and DIPA (5.8 mL, 3.75 mmol) in CH_2_Cl_2_ (5 mL) was added TBDPSCl (1.04 mL, 4.00 mmol) dropwise followed by DMAP (5 mg, 0.038 mmol) and the reaction stirred for 24 h at rt. Afterwards, TLC revealed the presence of the starting material **40**; thus an additional aliquot of TBDPSCl (0.521 mL, 2.00 mmol) was added and the reaction mixture was stirred for a further 3 h, whereafter it was quenched with water (60 mL) and then extracted with CH_2_Cl_2_ (3 × 60 mL). The combined organic extracts were washed successively with water (100 mL), brine (50 mL), filtered through cotton wool and the solvent was removed under reduced pressure. The residue so obtained was percolated through a short column of silica gel (further elution with EtOAc) and the subsequent eluent was concentrated under reduced pressure. RBC (elution first with hexane → 1 : 2 EtOAc–hexane) of the residue afforded the silyl protected product **41** (1.33 g, 76%) as a pale yellow oil; *δ*_H_ (500 MHz, CDCl_3_) 7.68–6.79 (14H, Ph), 4.56–4.44 (m, 2H, C*H*_2_Ar), 3.90–3.51 (8H, OCH_3_, H-2 propyl, 1- and 3-CH_2_ propyl), 3.25 (m, 2H, H-1 and 2), 3.12 (bs, 1H, OH), 1.97 (m, 2H, cyclitol), 1.66 (m, 2H, cyclitol), 1.30–1.15 (m, 4H, cyclitol), 1.05 (s, 9H, 3 × CH_3_); *δ*_C_ (125 MHz, CDCl_3_) 159.1, 135.6, 135.6, 134.7, 133.5, 133.49, 133.42, 130.9, 130.8, 129.73, 129.72, 129.3, 129.2, 127.74, 127.71, 113.79, 113.77, 82.9 (C-1 or 2), 82.3 (C-1 or 2), 80.7 (C-1 or 2), 80.6 (C-1 or 2), 71.7, 71.6, 71.2, 71.1, 71.0, 70.4, 65.0, 64.8, 55.3, 55.2, 30.6, 30.4, 30.11, 30.06, 29.7, 26.9, 23.69, 23.66, 19.29, 19.28; HRMS (ESI) calcd for C_33_H_44_NaO_5_Si [M + Na]^+^ 571.2856, found 571.2860.

### 1-[(*tert*-Butyldiphenylsilyl)oxy]-3-{(*trans*-2-[(4-methoxybenzyl)oxy)cyclohexyl]oxy}propan-2-yl methanesulfonate **42**

To the secondary alcohol **41** (1.33 g, 2.54 mmol) in pyridine (5 mL) was added mesyl chloride (0.63 mL, 8.14 mmol) dropwise. The reaction mixture was stirred at room temperature for 24 h and then quenched with saturated NaHCO_3_ (10 mL) followed by extraction with CH_2_Cl_2_ (3 × 20 mL). The combined organic extracts were washed with water (60 mL), brine (60 mL), filtered through cotton wool and then evaporated to dryness; whereafter toluene (5 mL) was added and evaporated therefrom. The resulting oil was passed through a short column of silica gel (elution with EtOAc) to give a clear yellow oil after evaporation to dryness under reduced pressure. This oil was used in the following step without further purification. However, a small sample of the product (100 mg) was purified by RBC (1 : 1 Et_2_O–hexane) to afford a clear, colourless oil of **42** for analytical analyses; *δ*_H_ (500 MHz, CDCl_3_) 7.60–6.73 (14H, Ph), 4.70 (m, 1H, H-2 propyl), 4.61–4.35 (m, 2H, C*H*_2_Ar), 3.83–3.69 (m, 7H, OCH_3_, 1- and 3-CH_2_ propyl), 3.18 (m, 2H, H-1 and 2), 2.90 (s, 3H, SO_2_CH_3_), 1.90 (m, 2H, cyclitol), 1.57 (m, 2H, cyclitol), 1.24–1.08 (m, 4H, cyclitol), 0.98 (s, 9H, 3 × CH_3_); *δ*_C_ (125 MHz, CDCl_3_) 159.1, 135.6, 135.54, 135.52, 132.94, 132.89, 132.8, 131.1, 131.0, 129.92, 129.90, 129.2, 129.1, 127.9, 113.78, 113.77, 82.5 (CH, CH propyl), 82.4 (CH, C-1 or 2), 82.1 (CH, C-1 or 2), 81.9 (CH, C-1 or 2), 80.5 (CH, C9), 71.2, 71.0, 68.9, 68.8, 63.5, 55.3 (OCH_3_), 38.5 (SO_2_CH_3_), 38.4, 30.0, 30.0, 29.8, 26.8, 23.5, 23.4, 19.2; HRMS (ESI) calcd for C_34_H_46_NaO_7_SSi [M + Na]^+^ 649.2626, found 649.2628.

### {2-Azido-3-[(*trans*-2-((4-methoxybenzyl)oxy)cyclohexyl)oxy]propoxy} (*tert*-butyl)diphenylsilane **43**

A solution of the mesylate **42** in DMF (10 mL) containing sodium azide (496 mg, 7.64 mmol) was heated and stirred at 125 °C for 24 h, cooled and then poured into water (40 mL). The resulting aqueous solution was extracted with CH_2_Cl_2_ (3 × 40 mL) and the combined organic extracts were washed successively with water (100 mL), brine (100 mL), filtered through cotton wool and concentrated under reduced pressure. A solution of the residue in EtOAc was percolated through a short column of silica gel (elution with EtOAc) and the eluent concentrated under reduced pressure. RBC of the residue (elution first with hexane → 1 : 1 Et_2_O–hexane) gave the azide **43** (1.30 g, 51%) as a clear oil; *δ*_H_ (500 MHz, CDCl_3_) 7.62–6.73 (14H, Ph), 4.44 (s, 2H, C*H*_2_Ar), 3.71–3.32 (8H, OCH_3_, H-2 propyl, 1- and 3-CH_2_ propyl), 3.22 (m, 2H, H-1 and 2), 1.86 (m, 2H, cyclitol), 1.55 (m, 2H, cyclitol), 1.25–1.04 (m, 4H, cyclitol), 0.99 (s, 9H, 3 × CH_3_); *δ*_C_ (125 MHz, CDCl_3_) 159.0, 135.9, 135.8, 135.6, 135.3, 134.8, 133.10, 133.06, 131.31, 131.29, 129.8, 129.7, 129.2, 129.12, 129.08, 129.0, 127.8, 127.73, 127.67, 113.74, 113.70, 82.2 (C-1 or 2), 82.0 (C-1 or 2), 80.5 (C-1 or 2), 80.4 (C-1 or 2), 71.5, 71.4, 71.19, 69.5, 69.0, 64.08, 64.05, 63.2, 63.0, 55.3 (OCH_3_), 30.0, 29.9, 26.9, 26.8, 26.6, 23.43, 23.40, 23.35, 19.2; HRMS (ESI) calcd for C_33_H_43_N_3_NaO_4_Si [M + Na]^+^ 596.2915, found 596.2926.

### *trans*-2-{2-Azido-3-[(*tert*-butyldiphenylsilyl)oxy]propoxy}cyclohexanol **44**

The PMB derivative **43** (131 mg, 0.240 mmol) was dissolved in a solution of 1% TFA in CH_2_Cl_2_ (8.62 mL). The reaction mixture was stirred at room temperature for 18 h; whereafter an additional aliquot of TFA (43 μL) was added because TLC indicated the presence of the PMB protected starting material (**43**). After a further 4 h, TLC revealed the absence of any starting material (**43**) and the reaction mixture was diluted with CH_2_Cl_2_ (40 mL) and washed with saturated NaHCO_3_ (40 mL), water (40 mL), brine (40 mL) and then filtered through cotton wool. The CH_2_Cl_2_ extract was concentrated under reduced pressure and the residue was percolated through a short column of silica gel (elution with EtOAc) and concentrated to dryness under reduced pressure. RBC purification of the residue (elution first with hexane → 1 : 1 Et_2_O–hexane) afforded the alcohol **44** (67 mg, 64%) as a clear oil; *δ*_H_ (500 MHz, CDCl_3_) 7.60–7.31 (10H, Ph), 3.74–3.11 (6H, H-1 or 2, H-2 propyl, 1- and 3-CH_2_ propyl), 2.99 (m, 1H, H-1 or 2), 2.60 (bd, 1H, OH), 1.96 (m, 2H, cyclitol), 1.65 (m, 2H, cyclitol), 1.23–1.03 (m, 4H, cyclitol), 1.00 (s, 9H, 3 × CH_3_); *δ*_C_ (125 MHz, CDCl_3_) 134.75, 134.58, 134.56, 132.2, 131.91, 131.87, 128.9, 126.81, 126.79, 83.6 (C-1 or 2), 83.5 (C-1 or 2), 72.8, 72.7, 67.3, 67.0, 62.8, 62.7, 62.0, 61.7, 31.02, 30.98, 28.7, 28.1, 25.9, 25.7, 23.1, 22.9, 18.2; HRMS (ESI) calcd for C_25_H_35_N_3_NaO_3_Si [M + Na]^+^ 476.2340, found 476.2351.

### Triethylammonium *trans*-2-{2-azido-3-[(*tert*-butyldiphenylsilyl)oxy]propoxy}cyclohexyl *n*-octadecyl phosphate **45**

This compound was obtained from the alcohol **44** (280 mg, 0.62 mmol) and the hydrogenphosphonate TEA salt **18** ^15^ (537 mg, 1.23 mmol) in the presence of pivaloyl chloride (0.48 mL, 3.86 mmol) essentially as described for the TEA salt **19**. After oxidation with iodine (623 mg, 2.47 mmol) in 9 : 1 pyridine–water followed by the same aqueous workup as described for **19**, column chromatography (CH_2_Cl_2_ → 8 : 1 CH_2_Cl_2_–MeOH) of the residue afforded the octadecyl phosphate TEA salt **45** (276 mg, 50%) as a yellow paste; *δ*_H_ (500 MHz, CDCl_3_) 7.63–7.30 (10H, Ph), 4.10 (m, 1H, H-1 or 2), 3.92–3.33 (7H, OCH_2_, H-2 propyl, 1- and 3-CH_2_ propyl), 3.28 (m, 1H, H-1 or 2), 3.00 (m, 6H, 3 × C*H*_2_CH_3_), 1.95–1.73 (m, 2H, cyclitol), 1.58–1.46 (m, 4H, OCH_2_C*H*_2_ and 2H cyclitol), 1.35–1.14 (43H, [CH_2_]_15_, 3 × CH_2_C*H*_3_ and 4H cyclitol), 1.73 (s, 9H, 3 × CH_3_), 0.80 (t, 3H, CH_3_, *J* = 6.8 Hz, CH_2_C*H*_3_); *δ*_C_ (125 MHz, CDCl_3_) 134.9, 134.8, 134.6, 132.6, 132.4, 132.1, 132.0, 128.9, 128.8, 126.8, 126.7, 78.8 (C-1 or 2), 78.6 (C-1 or 2), 76.6 (C-1 or 2), 70.8, 70.1, 68.2, 67.7, 65.7, 65.6, 65.5, 63.0, 62.9, 44.5 [N(*C*H_2_CH_3_)_3_], 30.9, 29.7, 29.6, 29.5, 29.0, 28.70, 28.65, 28.4, 27.2, 26.0, 25.9, 25.7, 24.8, 24.7, 21.7, 21.1, 21.0, 18.2, 14.3 (CH_2_ *C*H_3_), 8.5 [N(CH_2_ *C*H_3_)_3_]; *δ*_P_ (202 MHz, CDCl_3_) –1.2 (with heteronuclear decoupling); HRMS (ESI) calcd for C_43_H_71_N_3_O_6_PSi [M – NEt_3_ – H]^–^ 784.4885, found 784.4759.

### Triethylammonium *trans*-2-(2-azido-3-hydroxypropoxy)cyclohexyl *n*-octadecyl phosphate **46**

Compound **45** (68 mg, 0.077 mmol) was dissolved in THF (1 mL) and 1.0 M TBAF in THF (153 μL, 0.15 mmol) was added. The reaction mixture was stirred at rt for 16 h and then diluted with water (25 mL), extracted CH_2_Cl_2_ (3 × 25 mL) and the combined organic extracts were washed with aq. 1.0 M TEAB (2 × 10 mL). The organic phase was filtered through cotton wool, the solvent was removed under reduced pressure and the resulting residue was purified by column chromatography (8 : 1 CH_2_Cl_2_–MeOH) to afford the alcohol **46** (50 mg, 100%) as a white paste; *δ*_H_ (500 MHz, CDCl_3_) 4.08–3.33 (8H, OCH_2_, H-1 or 2, H-2 propyl, 1- and 3-CH_2_ propyl), 3.25 (m, 1H, H-1 or 2), 3.09 (m, 6H, 3 × C*H*_2_CH_3_), 2.14–1.99 (m, 2H, cyclitol), 1.66–1.59 (m, 4H, OCH_2_C*H*_2_ and 2H cyclitol), 1.30–1.19 (43H, [CH_2_]_15_, 3 × CH_2_C*H*_3_ and 4H cyclitol), 0.88 (t, 3H, CH_3_, *J* = 6.5 Hz, CH_2_C*H*_3_); *δ*_C_ (125 MHz, CDCl_3_) 81.6 (C-1 or 2), 78.6 (C-1 or 2), 68.6, 68.5, 66.03, 65.99, 62.4, 61.7, 60.9, 60.6, 45.4 [N(*C*H_2_CH_3_)_3_], 32.2, 32.0, 31.9, 30.8, 30.74, 30.68, 29.73, 29.67, 29.42, 29.38, 25.8, 23.9, 23.8, 23.8, 23.7, 22.7, 14.1 (CH_2_ *C*H_3_), 8.5 [N(CH_2_ *C*H_3_)_3_]; *δ*_P_ (202 MHz, CDCl_3_) –1.02 (with heteronuclear decoupling); HRMS (ESI) calcd for C_27_H_53_N_3_O_6_P [M – NEt_3_ – H]^–^ 546.3677, found 546.3673.

### Triethylammonium *trans*-2-(2-amino-3-hydroxypropoxy)cyclohexyl *n*-octadecyl phosphate **13**

Pearlman's catalyst [10–20% Pd(OH)_2_ on carbon, 15 mg] was added to a solution of the azide **46** (45 mg, 0.069 mmol) in 1 : 1 THF–MeOH (10 mL) and the mixture was stirred under a hydrogen atmosphere at rt for 2 h. Processing as described for **21** gave the amino TEA salt **13** (29 mg, 44%) as a white paste, which did not require any chromatographic purification; *δ*_H_ (500 MHz, CDCl_3_) 4.00–3.31 (8H, OCH_2_, H-1 or 2, H-2 propyl, 1- and 3-CH_2_ propyl), 3.10 (m, 1H, H-1 or 2), 3.02 (q, 6H, *J* = 7.4 Hz, 3 × C*H*_2_CH_3_), 2.04–1.93 (m, 2H, cyclitol), 1.63–1.50 (m, 4H, OCH_2_C*H*_2_ and 2H cyclitol), 1.33–1.05 (43H, [CH_2_]_15_, 3 × CH_2_C*H*_3_ and 4H cyclitol), 0.81 (t, 3H, CH_3_, *J* = 6.7 Hz, CH_2_C*H*_3_); *δ*_C_ (125 MHz, CDCl_3_) 81.6 (C-1 or 2), 77.9 (C-1 or 2), 67.1, 65.9, 64.8, 53.2, 44.5 [N(*C*H_2_CH_3_)_3_], 33.0, 31.8, 30.9, 29.7, 29.6, 29.33, 29.29, 29.0, 28.70, 28.65, 28.43, 28.35, 27.9, 24.9, 24.8, 24.8, 24.7, 23.2, 23.1, 22.8, 22.7, 13.1 (CH_2_ *C*H_3_), 7.5 [N(CH_2_ *C*H_3_)_3_]; *δ*_P_ (202 MHz, CDCl_3_) –0.23 (with heteronuclear decoupling); HRMS (ESI) calcd for C_27_H_55_NO_6_P [M – NEt_3_ – H]^–^ 520.3772, found 520.3747.

## Biological assays

### Materials

The synthesis of 1-d-6-*O*-(2-amino-2-deoxy-α-d-glucopyranosyl)-*myo*-inositol 1-(octadecyl phosphate), (**4**, α-d-Glc*p*NH_2_-I*P*C_18_),^15^ has been described previously. The corresponding *N*-acetyl derivate α-d-Glc*p*NAc-I*P*C_18_ (**3**) was prepared by treatment with acetic anhydride,^13^ and the concentration of stock solutions determined by measurement of the inositol content by selected ion-monitoring GC-MS.^8^ Bloodstream form *Trypanosome brucei* (variant MITat1.4) were isolated and membranes (cell-free system) prepared as described previously and stored at –80 °C.^26^

### Activity assays

Substrate recognition assays were performed using 500 pmol of α-d-Glc*p*NAc-PI (**1**) in incorporation buffer (25 mM Tris pH 8.0, 50 mM KCl, 50 mM MnCl_2_) and varying amounts of trypanosome cell-free system (0–15 × 10^6^ cell equivalents per assay) in 96-well plates containing 100 μL final volume, and incubated at 37 °C for 1 h. The reaction was quenched and the glycolipids enriched and analyzed by LC-MS/MS as described below.

Inhibition assays were performed in 96-well plates in 100 μL final volume, with 1% v/v DMSO with or without inhibitor. Trypanosome cell-free system (2.5 × 10^6^ cell equivalents per assay) in incorporation buffer were added to wells containing 500 pmol α-d-Glc*p*NAc-I*P*C_18_ (**3**) with or without inhibitor and incubated at 37 °C for 1 h. The reaction was quenched and the glycolipids enriched and analyzed by LC-MS/MS as described below.

### Glycolipid enrichment

Enrichment of glycolipids was performed in a 96-well plate format. Reactions were quenched by addition of 200 μL of 5% propan-1-ol, 5 mM NH_4_OAc, and the glycolipids were bound to C_18_ resin (50 mg Isolute Array cartridge), washed three times with 200 μL 5% propan-1-ol, 5 mM NH_4_OAc and eluted with 100 μL 40% propan-1-ol, 5 mM NH_4_OAc into a 96-well collection plate.

Prior to subsequent analysis, compound **13** was dried under nitrogen, resuspended in MeOH (100 μL) and any free amine reacted with excess *d*_6_-Ac_2_O (1.5 μL) in the presence of pyridine (10 μL) for 15 min. The reaction was quenched with water (50 μL), dried under nitrogen and resuspended in 100 μL 40% propan-1-ol, 5 mM NH_4_OAc.

### Liquid chromatography – tandem mass spectrometry of glycolipids

Glycolipids were analyzed by liquid chromatography coupled to an electrospray tandem mass spectrometer (LC-MS/MS). Samples (40 μL) were injected directly from a 96-well plate onto a 10 × 1 mm C_18_ column (ACE, 5 μM) and then eluted using a binary gradient of 5–80% propan-1-ol in 5 mM NH_4_OAc (Dionex Ultimate 3000). The gradient consisted of 2 min 0% B, 2–4 min 0–100% B, 4–8 min 100%, 8–9 min 100–0% B, 9–10 min 0% B where buffer A consisted of 5% propan-1-ol, 5 mM NH_4_OAc and buffer B 80% propan-1-ol, 5 mM NH_4_OAc. The glycolipids were analysed on an electrospray triple quadrapole mass spectrometer (Micromass Quattro Ultima) in multiple reaction monitoring mode.

For each pseudodisaccharide analogue (**7–12**), standards of the *N*-acetylated compound and corresponding free amine were analyzed separately in order to identify unique transitions for use in subsequent multiple reaction monitoring experiments (Table 1). The ratio of the integrals for these transitions was used to calculate the percentage of substrate conversion to product in a given sample. For compound **13**, standards of the *N*-acetylated compound and the *d*_3_-*N*-acetylated form were analyzed separately and found to produce a common fragment for use in subsequent multiple reaction monitoring experiments.

For inhibition assays, the turnover of the substrate α-d-Glc*p*NAc-I*P*C_18_ (**3**) was used to calculate the percentage of substrate conversion to product in a given sample.^10^ Inhibitor IC_50_ values were calculated using a four-parameter fit of eight-point potency curves derived from three independent experiments, and are quoted with a standard deviation.

### Trypanosome cell-free system assays

The formation of GPI precursors is monitored by following the incorporation of [^3^H]-mannose and then they were analysed using high-performance liquid chromatography and fluorography as described previously.^8^
