# Supplementary material for: Probing the substrate specificity of Trypanosoma brucei GlcNAc-PI de-N-acetylase with synthetic substrate analogues
Source: Org Biomol Chem. 2014 Feb 12;12(12):1919–34. doi: 10.1039/c3ob42164c (PMC4326964; doi:10.1039/c3ob42164c)
Supplement: Supplementary file 1 [file OB-012-C3OB42164C-s001.pdf]

This journal is © The Royal Society of Chemistry 2014

Probing the substrate specificity of *Trypanosoma brucei* GlcNAc-PI de-*N*-acetylase with synthetic substrate analogues.

Amy S. Capes,† Arthur Crossman,† Michael D. Urbaniak, Sophie H. Gilbert, Michael A. J. Ferguson,* Ian H. Gilbert*

Division of Biological Chemistry and Drug Discovery, College of Life Sciences, University of Dundee, Dow Street, Dundee, DD1 5EH, UK.

†These authors contributed equally to the work.

*Co-corresponding authors. Phone +44 (0) 1382 386240, Email m.a.j.ferguson@dundee.ac.uk and i.h.gilbert@dundee.ac.uk

Contents:

The following is a description of the analytical data for the β-anomers **8** and **10** and their respective intermediates.

Triethylammonium 1*R*,2*R*-1-*O*-(2-azido-3,4,6-tri-*O*-acetyl-2-deoxy-β-D-glucopyranosyl)-cyclohexanediol 2-(*n*-octadecylphosphate) S1

This phosphoric diester was obtained from the pseudodisaccharide derivative **17** (72 mg, 0.17 mmol) and the H-phosphonate **18**[15] (202 mg, 0.46 mmol) essentially as described in the preparation of the 2-(*n*-octadecyl phosphate) **19**; yield (60 mg, 41%); $[\alpha]_D^{25}$ –10.0º (*c* 1.58, $CHCl_3$); δ_H (500 MHz, $CDCl_3$) 4.90 (m, 2H, H-3′ and 4′), 4.50 (d, 1H, $J_{1',2'}$ = 8.0 Hz, H-1′), 4.28 (m, 1H, H-1 or 2), 4.20 (dd, 1H, $J_{5',6'a}$ = 3.7, $J_{6'a,6'b}$ = 12.2 Hz, H-6′a), 4.05 (m, 1H, H-6′b), 3.84 (m, 3H, OCH_2 and H-1 or 2), 3.63 (m, 1H, H-5′), 3.40 (dd, 1H, , $J_{2',3'}$ = 9.8 Hz, H-2′), 2.83 (q, 6H, *J* = 7.3 Hz, 3 x CH_2CH_3), 2.04 – 1.80 (m, 11H, 3 x $COCH_3$ and 2H-

cyclitol), 1.65 – 1.50 (m, 6H, OCH_2CH_2 and 4H-cyclitol), 1.35 – 1.15 (41H, $[CH_2]_{15}$, 3x CH_2CH_3 and 2H-cyclitol), 0.80 (t, 3H, J = 6.8 Hz, CH_2CH_3); δ_C (125 MHz, $CDCl_3$) 169.8 – 168.6 (3 x $COCH_3$), 99.4 (C-1′), 77.0 (C-1 or C-2), 73.1 (C-1 or C-2), 71.6, 71.0 (C-5′), 67.4, 65.3 (OCH_2), 63.1 (C-2′), 60.9 (C-6′), 44.4 [$N(CH_2CH_3)_3$], 30.9, 29.7, 28.7 – 28.4, 27.4, 25.6, 24.8, 19.9 – 19.6, 13.1 [$N(CH_2CH_3)_3$], 7.5 (CH_2CH_3); δ_P (202 MHz, $CDCl_3$) –1.5 (with heteronuclear decoupling); HRMS (ESI) calcd. for $C_{36}H_{63}N_3O_{12}P$ $[M - NEt_3 - H]^-$ 760.4155, found 760.4124.

1*R*,2*R*-1-*O*-(2-Amino-2-deoxy-β-D-glucopyranosyl)-cyclohexanediol 2-(*n*-octadecylphosphate) 8

To a solution of the TEA salt **S1** (55 mg, 0.06 mmol) in 1:1 CH_2Cl_2 – MeOH (10 mL) was added 5.4 M NaOMe in MeOH (0.10 mL). The mixture was kept for 3 h at rt and was then neutralised with Amberlite IR-120 (H^+) ion-exchange resin, filtered and the filtrate concentrated under reduced pressure. This crude product was directly used in the next step. A solution of the crude azide in 1:1 stabilised THF – *n*-propanol (5 mL) containing 10-20% $Pd(OH)_2$ on carbon (5 mg) was stirred under a hydrogen atmosphere at rt for 1 h. Afterwads, it was percolated through a short column of Chelex 100 on a bed of Celite (further elution with 1:1 THF – *n*-propanol). The eluent was concentrated under reduced pressure and the ensuing residue was dissolved in 10:10:3 $CHCl_3$ – MeOH – H_2O (2.3 mL), plus 3 drops of TEA, and then purified by Iatrobead SiO_2 column chromatography (elution with 4:1 CH_2Cl_2 – MeOH) to furnish the amino compound **8** (4 mg, 10%); $[\alpha]_D^{25}$ +28.0° (*c* 0.4, 10:10:3 $CHCl_3$ – MeOH – H_2O); δ_H (500 MHz, 10:10:3 $CDCl_3$ – MeOH-d_4 – D_2O), 4.58 (H-1′ and HOD), 4.16 – 3.26 (8H, OCH_2, H-1, 2, 3′, 4′, 6′a,b), 3.03 (m, 1H, H-5′), 3.05 (dd, 1H, $J_{1',2'}$ = 8.5, $J_{2',3'}$ = 9.0 Hz, H-2′), 2.20 – 1.55 (6H, OCH_2CH_2 and 4H-cyclitol), 1.44 – 1.21 (34H, $[CH_2]_{15}$ and 4H-cyclitol), 0.90 (t, 3H, J = 7.1 Hz, CH_2CH_3); δ_C (HSQC 125 MHz, 10:10:3 $CDCl_3$ – MeOH-d_4 – D_2O) 94.5 (C-1′), 80.5 (C-1 or C-2), 75.4 (C-1 or C-2), 72.7 (C-3′), 70.2, 65.2

(OCH$_2$), 60.8 (C-6′), 54.3 (C-5′), 55.7 (C-2′), 32.1, 31.8, 30.4, 29.3, 27.6, 25.5, 23.4, 22.2, 13.3 (CH$_2$*C*H$_3$); δ_P (202 MHz, 10:10:3 CDCl$_3$ – MeOH-d$_4$ – D$_2$O) –0.6 (with heteronuclear decoupling); HRMS (ESI) calcd. for $C_{30}H_{59}NO_9P$ [M – H]$^-$ 608.3933, found 608.3921.

1*R*,2*R*-1-*O*-(2-Azido-3,4,6-tri-*O*-acetyl-2-deoxy-β-D-glucopyranosyl)-2-*O*-(*tert*-butyldimethylsilyl)-cyclohexanediol S2

This compound was obtained from the alcohol **17** (100 mg, 0.23 mmol), 2,6-lutidine (53.6 μL, 0.46 mmol) and *tert*-butyldimethylsilyl trifluoromethane sulfonate (80.2 μL, 0.35 mmol) essentially as described for the α-anomer **22**, yield (100 mg, 80%) as a white solid; mp 109 – 111 °C; $[\alpha]_D^{25}$ –23.5° (*c* 1.48, CHCl$_3$); δ_H (500 MHz, CDCl$_3$) 5.02 (t, 1H, $J_{4',5'}$ = 9.7 Hz, H-4′), 4.95 (t, 1H, $J_{3',4'}$ = 9.8 Hz, H-3′), 4.49 (d, 1H, $J_{1',2'}$ = 8.1 Hz, H-1′), 4.24 (dd, 1H, $J_{5',6'a}$ = 4.4, $J_{6'a,6'b}$ = 12.2 Hz, H-6′a), 4.09 (dd, 1H, $J_{5',6'b}$ = 2.5, $J_{6'a,6'b}$ = 12.2 Hz, H-6′b), 3.61 (m, 3H, H-5′, 1 and 2), 3.46 (dd, 1H, $J_{2',3'}$ = 10.0 Hz, H-2′), 2.10 – 2.01 (3 x s, 9H, 3 x COCH$_3$), 1.97 (m, 1H, cyclitol), 1.84 (m, 1H, cyclitol), 1.65 (m, 2H, cyclitol), 1.40 (m, 2H, cyclitol), 1.28 (m, 2H, cyclitol), 0.89 (s, 9H, 3 x CH$_3$), 0.12 – 0.08 (2 x s, 6H, 2 x CH$_3$); δ_C (125 MHz, CDCl$_3$) 170.7 – 169.7 (3 x *C*OCH$_3$), 99.4 (C-1′), 80.0, 72.4 (C-3′), 71.6, 71.5, 68.5 (C-4′), 63.9 (C-2′), 62.0 (C-6′), 32.1, 27.4, 25.8, 22.3, 22.1, 20.7 (CO*C*H$_3$), 20.6 (CO*C*H$_3$), 18.2, -4.6, -4.8; HRMS (ESI) calcd. for $C_{24}H_{42}N_3O_9Si$ [M + H]$^+$ 544.2685, found 544.2684.

1*R*,2*R*-1-*O*-(2-Azido-2-deoxy-β-D-glucopyranosyl)-2-*O*-(*tert*-butyldimethylsilyl)-cyclohexanediol S3

To a solution of the triacetate **S2** (147 mg, 0.27 mmol) in 1:1 CH$_2$Cl$_2$ – MeOH (72 mL) was added 5.4 M NaOMe in MeOH (180 μL). The mixture was kept for 30 min at rt and was then neutralised with Amberlite IR-120 (H$^+$) ion-exchange resin, filtered and the filtrate concentrated under reduced pressure. Processing as described for the α-anomer **23** gave the β-anomer **S3** (107 mg, 95%) as a waxy solid; $[\alpha]_D^{25}$ –8.2° (*c* 1.09, CHCl$_3$); δ_H (500 MHz, CDCl$_3$) 4.46 (d, 1H, $J_{1',2'}$ = 7.7 Hz, H-1′), 3.88 (dd, 1H, $J_{5',6'a}$ = 3.1, $J_{6'a,6'b}$ = 12.0 Hz, H-6′a),

3.79 (dd, 1H, $J_{5',6'b}$ = 4.3, $J_{6'a,6'b}$ = 12.0 Hz, H-6′b), 3.58 (m, 3H, H-4′, 1 and 2), 3.36 (t, 1H, $J_{3',4'}$ = 9.9 Hz, H-3′), 3.28 (m, 2H, H-2′ and 5′), 2.00 (m, 1H, cyclitol), 1.85 (m, 1H, cyclitol), 1.66 (m, 2H, cyclitol), 1.41 (m, 2H, cyclitol), 1.28 (m, 2H, cyclitol), 0.89 (s, 9H, 3 x CH_3), 0.07 (s, 6H, 2 x CH_3); δ_C (125 MHz, $CDCl_3$) 99.4 (C-1′), 79.7, 75.1, 74.7 (C-4′), 72.2, 70.4, 66.2, 62.1 (C-6′), 32.3, 27.7, 25.8, 22.4, 21.7, 18.2, -4.5, -4.8; HRMS (ESI) calcd. for $C_{18}H_{36}N_3O_6Si$ $[M + H]^+$ 418.2368, found 418.2358.

1*R*,2*R*-1-*O*-(2-Azido-3,4,6-tri-*O*-benzyl-2-deoxy-β-D-glucopyranosyl)-2-*O*-(*tert*-butyldimethylsilyl)-cyclohexanediol S4

This compound was obtained from the triol **S3** (174 mg, 0.42 mmol), NaH (46 mg, 1.93 mmol) and benzyl bromide (230 μL, 1.93 mmol) essentially as described for the α-anomer **24**, yield (234 mg, 81%); $[\alpha]_D^{25}$ –29.1° (*c* 1.48, $CHCl_3$); δ_H (500 MHz, $CDCl_3$) 7.35 – 7.10 (15H, 3 x Ph), 4.85 – 4.46 (6H, 3 x C*H*$_2$Ar), 4.31 (d, 1H, $J_{1',2'}$ = 7.6 Hz, H-1′), 3.70 – 3.54 (m, 5H, H-1, 2, 3′ and 6′a,b), 3.39 – 3.30 (m, 3H, H-2′, 4′ and 5′), 1.90 (m, 1H, cyclitol), 1.80 (m, 1H, cyclitol), 1.60 (m, 2H, cyclitol), 1.40 (m, 2H, cyclitol), 1.25 (m, 2H, cyclitol), 0.82 (s, 9H, 3 x CH_3), 0.02 (2 x s, 6H, 2 x CH_3); δ_C (125 MHz, $CDCl_3$) 138.2 – 138.0 (Ph), 128.5 – 127.6 (Ph), 99.4 (C-1′), 83.1, 78.8, 77.9, 75.6, 75.1, 73.6, 71.3, 68.8 (C-6′), 66.6, 31.6, 26.9, 25.9, 21.9, 21.8, 18.2, -4.5, -4.8; HRMS (ESI) calcd. for $C_{39}H_{54}N_3O_6Si$ $[M + H]^+$ 688.3776, found 688.3754.

1*R*,2*R*-1-*O*-(2-Amino-3,4,6-tri-*O*-benzyl-2-deoxy-β-D-glucopyranosyl)-2-*O*-(*tert*-butyldimethylsilyl)-cyclohexanediol S5

To a stirred solution of **S4** (234 mg, 0.34 mmol) in 10:1 THF – water (5 mL) at 60 °C was added Ph_3P (268 mg, 1.02 mmol). After 3 h, TLC showed the complete disappearance of the starting material. Processing as described for the α-anomer **25** gave the β-anomer **S5** (90 mg, 40%); $[\alpha]_D^{25}$ –15.2° (*c* 1.06, $CHCl_3$); δ_H (500 MHz, $CDCl_3$) 7.34 – 7.14 (15H, 3 x Ph), 4.97 – 4.49 (6H, 3 x C*H*$_2$Ar), 4.24 (d, 1H, $J_{1',2'}$ = 7.8 Hz, H-1′), 3.72 – 3.39 (7H, H-1, 2, 3′, 4′,

5′ and 6′a,b), 2.85 (t, 1H $J_{2',3'}$ = 9.8 Hz, H-2′),1.92 – 1.19 (8H, cyclitol), 0.84 (s, 9H, 3 x CH_3), 0.01 (2 x s, 6H, 2 x CH_3); δ_C (125 MHz, $CDCl_3$) 138.5 – 138.1 (Ph), 128.6 – 127.5 (Ph), 101.1 (C-1′), 85.5, 78.8, 77.8, 75.5, 75.4, 74.8, 74.5, 73.6, 71.2, 69.0, 57.0 (C-2′), 31.4, 26.9, 25.9, 22.0, 21.6, 18.2, -4.6, -4.8; HRMS (ESI) calcd. for $C_{39}H_{56}NO_6Si$ $[M + H]^+$ 662.3871, found 662.3877.

1*R*,2*R*-1-*O*-[2-*N*-(*tert*-Butoxycarbonyl)amino-3,4,6-tri-*O*-benzyl-2-deoxy-β-D-glucopyranosyl]-2-*O*-(*tert*-butyldimethylsilyl)-cyclohexanediol S6

The amine **S5** (89 mg, 0.13 mmol) was dissolved in EtOAc (10 mL) at rt. Di-tert-butyldicarbonate (35 mg, 0.16 mmol) was then added and the mixture was stirred overnight at rt. Processing as described for the α-anomer **26** gave the β-anomer **S6** (89 mg, 90%) as a white solid, mp 114 – 116 °C; $[\alpha]_D^{25}$ –7.3° (*c* 3.13, $CHCl_3$); δ_H (500 MHz, $CDCl_3$) 7.32 – 7.15 (15H, 3 x Ph), 4.81 – 4.47 (7H, H-1′ and 3 x CH_2Ar), 4.06 (brs, 1H, H-3′), 3.68 (m, 3H, H-6′a,b and H-1 or 2), 3.55 (brt, 2H, *J* = 8.9 Hz, H-4′ and H-1 or 2), 3.44 (brs, 1H, H-5′), 3.10 (brs, 1H, H-2′), 1.83 – 1.30 (15H, 6H-cyclitol and 3 x BocCH_3), 1.23 (m, 2H, cyclitol), 0.83 (s, 9H, 3 x CH_3), 0.00 (s, 6H, 2 x CH_3); δ_C (125 MHz, $CDCl_3$) 155.2 (C=O), 138.4 (Ph), 128.4 – 127.5 (Ph), 97.8 (C-1′), 81.1 (C-3′), 79.3, 78.9, 77.9, 75.0, 74.9 (C-5′), 74.7, 73.5, 70.8, 69.1 (C-6′), 58.3 (C-2′), 30.8, 28.4, 26.4, 25.9, 21.5, 21.3, 18.2, -4.6, -4.8; HRMS (ESI) calcd. for $C_{44}H_{64}NO_8Si$ $[M + H]^+$ 762.4396, found 762.4432.

1*R*,2*R*-1-*O*-[2-*N*-(*tert*-Butoxycarbonyl)amino-3,4,6-tri-*O*-benzyl-2-deoxy-β-D-glucopyranosyl]-cyclohexanediol S7

To a stirred solution of the silyl derivative **S6** (89 mg, 0.12 mmol) in THF (10 mL) at 0° C was added ~70% HF-pyridine (150 μL). The solution was stirred overnight at rt whereafter TLC revealed the complete disappearance of the starting material. Processing as described for the α-anomer **27** gave the β-anomer **S7** (46 mg, 60%) as a white solid, mp 168 – 170 °C;; $[\alpha]_D^{25}$ +10.5° (*c* 0.82, $CHCl_3$); δ_H (500 MHz, $CDCl_3$) 7.34 – 7.15 (15H, 3 x Ph), 4.85

– 4.43 (8H, H-1′, NH and 3 x C*H*$_2$Ar), 4.04 (brs, 1H, H-3′), 3.73 – 3.50 (4H, H-4′, 5′ and 6′a,b), 3.42 (m, 1H, H-1 or 2), 3.27 (m, 1H, H-1 or 2), 3.15 (brs, 1H, H-2′), 2.05 (m, 1H, cyclitol), 1.91 (m, 1H, cyclitol), 1.67 (m, 2H, cyclitol), 1.45 (s, 9H, 3 x CH_3), 1.36 – 1.13 (4H, cyclitol); δ_C (125 MHz, $CDCl_3$) 154.5 (C=O), 136.9 (Ph), 127.4 – 126.6 (Ph), 100.2 (C-1′), 86.3 (C-1 or 2), 80.0 (C-3′), 78.5, 77.4, 74.1, 73.7, 72.5, 72.2 (C-1 or 2), 67.7 (C-6′), 57.0 (C-2′), 31.2, 30.0, 27.3, 23.3, 22.8; HRMS (ESI) calcd. for $C_{38}H_{50}NO_8$ $[M + H]^+$ 648.3531, found 648.3546.

Triethylammonium 1*R*,2*R*-1-*O*-[2-*N*-(*tert*-butoxycarbonyl)amino-3,4,6-tri-*O*-benzyl-2-deoxy-β-D-glucopyranosyl]-cyclohexanediol 2-(1,2-di-*O*-hexadecanoyl-*sn*-glycerol 3-phosphate) S8

This compound was obtained from the alcohol **S7** (62 mg, 0.096 mmol) and 1,2-di-*O*-hexadecanoyl-*sn*-glycerol 3-hydrogenphosphonate TEA salt **28**[19] (141 mg, 0.19 mmol) in the presence of pivaloyl chloride (77 μL, 0.62 mmol) essentially as described for the 2-(*n*-octadecyl phosphate) **19**. After the oxidation with iodine (316 mg, 1.24 mmol) in 9:1 pyridine – water followed by the same aqueous workup as described for **19**, RBC (elution first with CH_2Cl_2 and then with 20:1 → 15:1 CH_2Cl_2 – MeOH) afforded the TEA phosphate derivative **S8** (118 mg, 89%) as an opaque paste; $[\alpha]_D^{25}$ +4.4° (*c* 1.25, $CHCl_3$); δ_H (500 MHz, $CDCl_3$) 7.29 – 7.10 (15H, 3 x Ph), 5.20 (m, 2H, NH and H-2 glycerol), 4.81 (brs, 1H, H-1′), 4.78 – 4.42 (6H, 3 x C*H*$_2$Ar), 4.35 (dd, 1H, *J* = 3.0, *J* = 12.0, 1- or 3-CHa glycerol), 4.05 (m, 3H, H-3′, 1 or 2 cyclitol and 1- or 3-CHb glycerol), 4.00 (m, 1H, 1- or 3-CHc glycerol), 3.90 (m, 1H, 1- or 3-CHd glycerol), 3.70 (m, 3H, H-6′a,b and 1 or 2 cyclitol), 3.52 (t, 1H, $J_{3',4'} = J_{4',5'}$ = 9.3 Hz, H-4′), 3.42 (brd, 1H, *J* = 9.5 Hz, H-5′), 3.00 (brs, 1H, H-2′), 2.63 (q, 6H, *J* = 7.0 Hz, 3 x C*H*$_2$CH$_3$), 2.19 (m, 4H, 2 x $COCH_2$), 2.06 (m, 1H, cyclitol), 1.87 (m, 1H, cyclitol), 1.50 (m, 6H, 2 x COCH$_2$C*H*$_2$ and 2H cyclitol), 1.36 (s, 9H, 3 x CH_3), 1.26 – 1.14 (52H, 2 x $[CH_2]_{12}$ and 4H cyclitol), 1.04 (t, 9H, *J* = 7.3 Hz, 3 x CH$_2$C*H*$_3$), 0.81 (t, 6H, *J* = 7.1

Hz, 2 x CH_2CH_3); δ_C (125 MHz, $CDCl_3$) 172.5 (C=O), 172.1 (C=O), 154.4 (C=O), 137.7 – 137.5 (Ph), 127.3 – 126.4 (Ph), 96.5 (C-1′), 80.0, 77.7 (C-4′), 75.8, 74.0, 73.8, 73.6, 72.3, 69.7, 62.6, 62.4, 62.0, 61.7, 57.2 (C-2′), 44.9, 33.3, 33.1, 30.9, 28.7 – 28.2, 27.4, 26.6, 24.0, 21.7, 21.0 13.1, 9.40; δ_P (202 MHz, $CDCl_3$) –0.84 (with heteronuclear decoupling); HRMS (ESI) calcd. for $C_{73}H_{115}NO_{15}P$ [M – NEt_3 – H]$^-$ 1276.8010, found 1276.7946.

1*R*,2*R*-1-*O*-[2-*N*-(*tert*-Butoxycarbonyl)amino-2-deoxy-β-D-glucopyranosyl]-cyclohexanediol 2-(1,2-di-*O*-hexadecanoyl-*sn*-glycerol 3-phosphate) S9

A solution of the benzylated compound **S8** (50 mg, 0.036 mmol) in 1:1 THF – *n*-propanol (10 mL) containing 10-20% $Pd(OH)_2$ on carbon (15 mg) was stirred under 3 atm of hydrogen for 2.5 h before it was percolated through a short column of Chelex 100 on a bed of Celite (further elution with 1:1 THF – *n*-propanol). The eluent was concentrated under reduced pressure and the ensuing residue was purified by column chromatography (elution gradient 4:1 → 1:1 CH_2Cl_2 – MeOH) to give the Boc protected derivative **S9** (30 mg, 83%); $[\alpha]_D^{25}$ +1.9° (*c* 2.30, 1:1 CH_2Cl_2 – MeOH); δ_H (500 MHz, 1:1 $CDCl_3$ – MeOH-d_4) 5.25 (m, 1H, H-2 glycerol), 4.49 (d, 1H, $J_{1',2'}$ = 8.2, H-1′), 4.43 (dd, 1H, *J* = 2.4, *J* = 12.0 Hz, 1- or 3-CHa glycerol), 4.20 (dd, 1H, 1- or 3-CHb glycerol), 4.05 (m, 1H, 1- or 3-CHc glycerol), 3.95 (m, 1H, 1- or 3-CHd glycerol), 3.88 (m, 1H, H-6′a), 3.75 (m, 1H, H-6′b), 3.60 (m, 2H, H-1 and 2), 3.50 – 3.20 (m, 4H, H-2′, 4′, 3′ and 5′), 2.33 (m, 4H, 2 x $COCH_2$), 2.08 (m, 1H, cyclitol), 1.98 (m, 1H, cyclitol), 1.73 – 1.57 (m, 6H, 2 x $COCH_2CH_2$ and 2H cyclitol), 1.46 (s, 9H, 3 x CH_3), 1.40 – 1.20 (52H, 2 x $[CH_2]_{12}$ and 4H cyclitol), 0.89 (t, 6H, *J* = 7.1 Hz, 2 x CH_2CH_3); δ_C (125 MHz, 1:1 $CDCl_3$ – MeOH-d_4) 174.5 (C=O), 174.2 (C=O), 157.9 (C=O), 101.6 (C-1′), 80.9, 76.7, 74.5, 71.1, 70.8, 64.0, 63.2, 61.1 (C-6′), 57.7, 34.7, 34.6, 32.4, 31.4, 30.2 – 29.6, 28.6, 25.4, 23.2, 14.3; δ_P (202 MHz, 1:1 $CDCl_3$ – MeOH-d_4) –0.60 (with heteronuclear decoupling); HRMS (ESI) calcd. for $C_{52}H_{97}NO_{15}P$ [M – H]$^-$ 1006.6601, found 1006.6585.

1*R*,2*R*-1-*O*-(2-Amino-2-deoxy-β-D-glucopyranosyl)-cyclohexanediol 2-(1,2-di-*O*-hexadecanoyl-*sn*-glycerol 3-phosphate) 10

To a solution of the *tert*-butoxycarbonyl protected compound **S9** (23 mg, 0.023 mmol) in 1:1 CH_2Cl_2 – MeOH (1 mL) was added 9:1 trifluoroacetic acid (TFA) – water (5 mL). After stirring 4 h at rt, toluene (5 mL) was added and the solvents were removed under reduced pressure. Toluene (2 x 5 mL) was evaporated off twice from the residue (to remove traces of TFA and water) to give the crude pseudodisaccharide phosphate derivative **10**. A short column of Iatrobeads was conditioned with 4:1 CH_2Cl_2 – MeOH before the crude residue of **10** (solubilised in 2 mL of 10:10:3 $CHCl_3$ – MeOH – H_2O plus 3 drops of TEA) was applied to the column. Elution with 4:1 CH_2Cl_2 – MeOH afforded pure **10** (15 mg, 71%) after the requisite fractions were combined and the solvents evaporated to dryness under reduced pressure; $[\alpha]_D^{25}$ –5.4° (*c* 1.50, 1:1 $CHCl_3$ – MeOH); δ_H (500 MHz, 1:1 $CDCl_3$ – MeOH-d_4) 5.23 (brs, 1H, H-2 glycerol), 4.45 (d, 1H, *J* = 12.1 Hz, 1- or 3-CHa glycerol), 4.38 (d, 1H, $J_{1',2'}$ = 7.5 Hz, H-1′), 4.21 (dd, 1H, *J* = 8.4, *J* = 12.1 Hz, 1- or 3-CHb glycerol), 4.12 (m, 1H, H-1 or 2), 4.05 (m, 1H, 1- or 3-CHc glycerol), 3.95 (m, 1H, 1- or 3-CHd glycerol), 3.85 (d, 1H, $J_{6'a,6'b}$ = 12.2 Hz, H-6′a), 3.75 (m, 1H, H-1 or 2), 3.68 (d, 1H, $J_{6'a,6'b}$ = 12.2 Hz, H-6′b), 3.33 (m, 3H, H-3′, 4′ and 5′), 2.65 (m, 1H, H-2′), 2.34 (m, 4H, 2 x $COCH_2$), 2.10 (m, 1H, cyclitol), 2.01 (m, 1H, cyclitol) 1.70 – 1.56 (m, 6H, 2 x $COCH_2CH_2$ and 2H cyclitol), 1.53 – 1.24 (52H, 2 x $[CH_2]_{12}$ and 4H cyclitol), 0.89 (t, 6H, *J* = 6.8 Hz, 2 x CH_2CH_3); δ_C (125 MHz, 1:1 $CDCl_3$ – MeOH-d_4) 174.7 (C=O), 174.2 (C=O), 102.2 (C-1′), 79.1 (C-1 or 2), 78.0, 77.4, 77.0, 72.0, 71.6, 64.7 (CH_2 glycerol), 64.3 (CH_2 glycerol), 62.6 (C-6′), 58.0 (C-2′), 35.6, 35.4, 33.3, 31.0 – 30.5, 26.3, 24.0, 23.7, 15.2; δ_P (202 MHz, 1:1 $CDCl_3$ – MeOH-d_4) –0.19 (with heteronuclear decoupling); HRMS (ESI) calcd. for $C_{52}H_{97}NO_{15}P$ $[M – H]^-$ 906.6077, found 906.6057.
