# Supplementary material for: Probing the substrate specificity of Trypanosoma brucei GlcNAc-PI de-N-acetylase with synthetic substrate analogues
Source: Org Biomol Chem. 2014 Feb 12;12(12):1919–34. doi: 10.1039/c3ob42164c (PMC4326964; doi:10.1039/c3ob42164c)
Supplement: Supplementary file 2 [file OB-012-C3OB42164C-s002.pdf]

Electronic Supplementary Material (ESI) for Organic & Biomolecular Chemistry.
This journal is © The Royal Society of Chemistry 2014

Probing the substrate specificity of *Trypanosoma brucei* GlcNAc-PI de-*N*-acetylase with synthetic substrate analogues.

Amy S. Capes,† Arthur Crossman,† Michael D. Urbaniak, Sophie H. Gilbert, Michael A. J. Ferguson,* Ian H. Gilbert*

Division of Biological Chemistry and Drug Discovery, College of Life Sciences, University of Dundee, Dow Street, Dundee, DD1 5EH, UK.

†These authors contributed equally to the work.

*Co-corresponding authors. Phone +44 (0) 1382 386240, Email m.a.j.ferguson@dundee.ac.uk and i.h.gilbert@dundee.ac.uk

Contents:

Supporting NMR data in pdf format for all the new compounds described in this manuscript.

AC441
C13CPDfast.d MeOD {C:\Bruker\TOPSPIN} AC 9

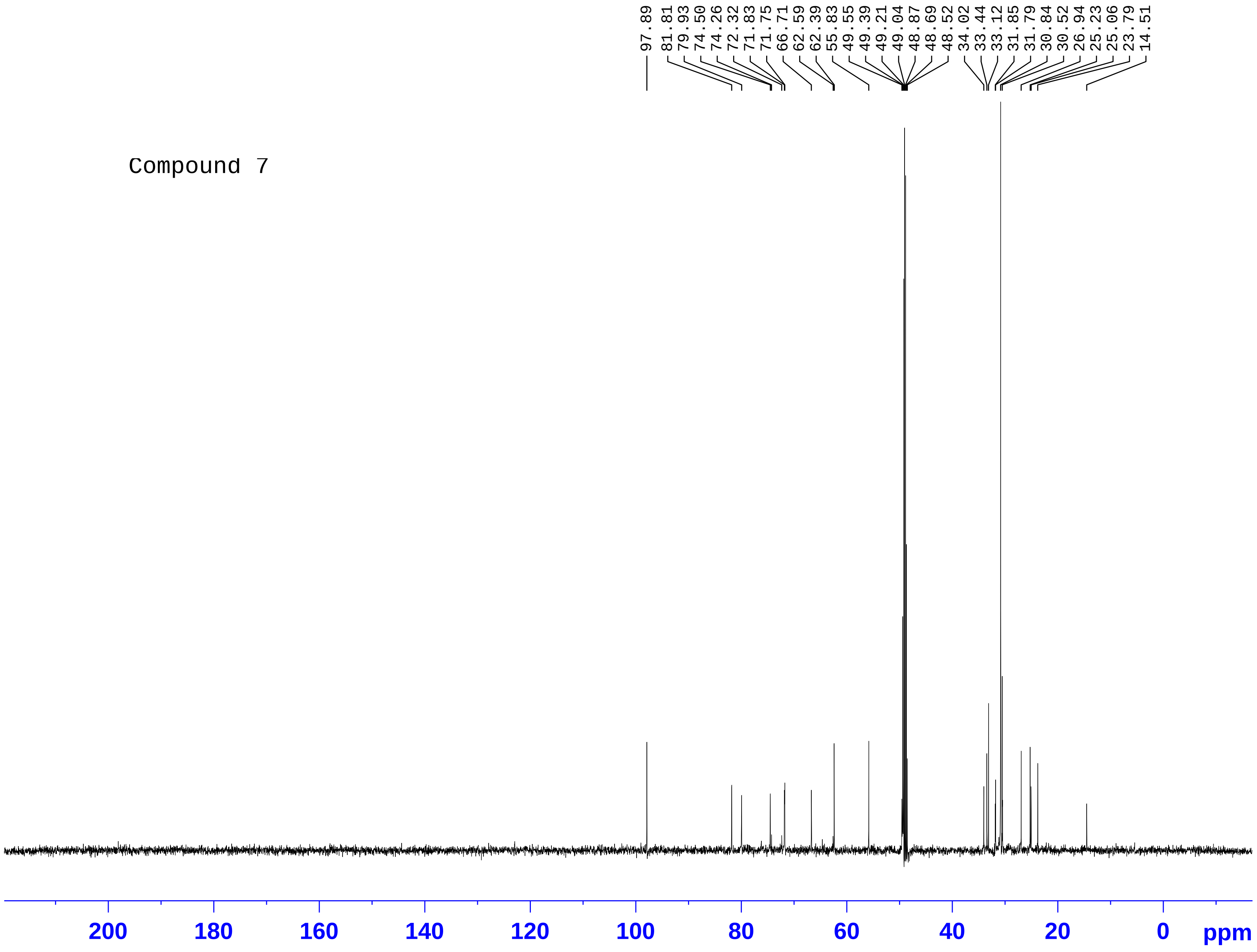

UNIVERSITY OF DUNDEE

NAME	AC-AC441	
EXPNO	7	
PROCNO	1	
Date_	20111005	
Time	16.39	
INSTRUM	spect	
PROBHD	5 mm QNP 1H/13	
PULPROG	zgpg30	
TD	16384	
SOLVENT	MeOD	
NS	800	
DS	4	
SWH	29761.904	Hz
FIDRES	1.816522	Hz
AQ	0.2753012	sec
RG	2050	
DW	16.800	usec
DE	6.00	usec
TE	295.7	K
D1	0.30000001	sec
d11	0.03000000	sec
DELTA	0.20000002	sec
TD0	1	

======== CHANNEL f1 ========

NUC1	13C	
P1	8.18	usec
PL1	0.00	dB
SFO1	125.7703643	MHz

======== CHANNEL f2 ========

CPDPRG2	waltz16	
NUC2	1H	
PCPD2	80.00	usec
PL2	-1.00	dB
PL12	16.00	dB
PL13	16.00	dB
SFO2	500.1320005	MHz
SI	8192	
SF	125.7576104	MHz
WDW	EM	
SSB	0	
LB	1.00	Hz
GB	0	
PC	1.40	

AC441
PROTON.d MeOD {C:\Bruker\TOPSPIN} AC 8

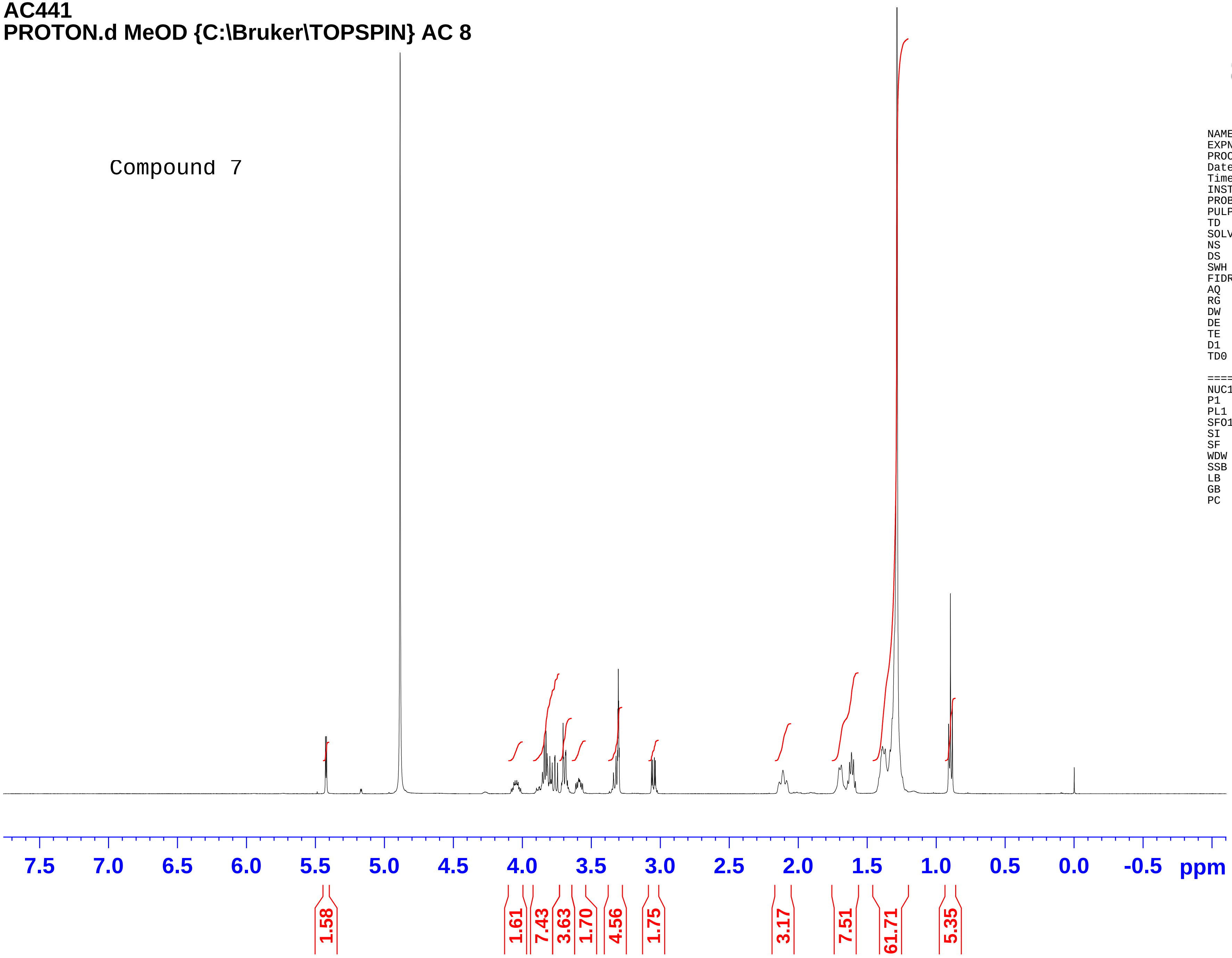

UNIVERSITY OF DUNDEE

NAME	AC-AC441
EXPNO	4
PROCNO	1
Date_	20111005
Time	16.11
INSTRUM	spect
PROBHD	5 mm QNP 1H/13
PULPROG	zg30
TD	65536
SOLVENT	MeOD
NS	16
DS	2
SWH	10330.578 Hz
FIDRES	0.157632 Hz
AQ	3.1719923 sec
RG	80.6
DW	48.400 usec
DE	6.00 usec
TE	294.9 K
D1	1.00000000 sec
TD0	1

======== CHANNEL f1 ========

NUC1	1H
P1	11.20 usec
PL1	-1.00 dB
SFO1	500.1330885 MHz
SI	65536
SF	500.1300135 MHz
WDW	EM
SSB	0
LB	0.30 Hz
GB	0
PC	1.40

AC390P

Compound 8

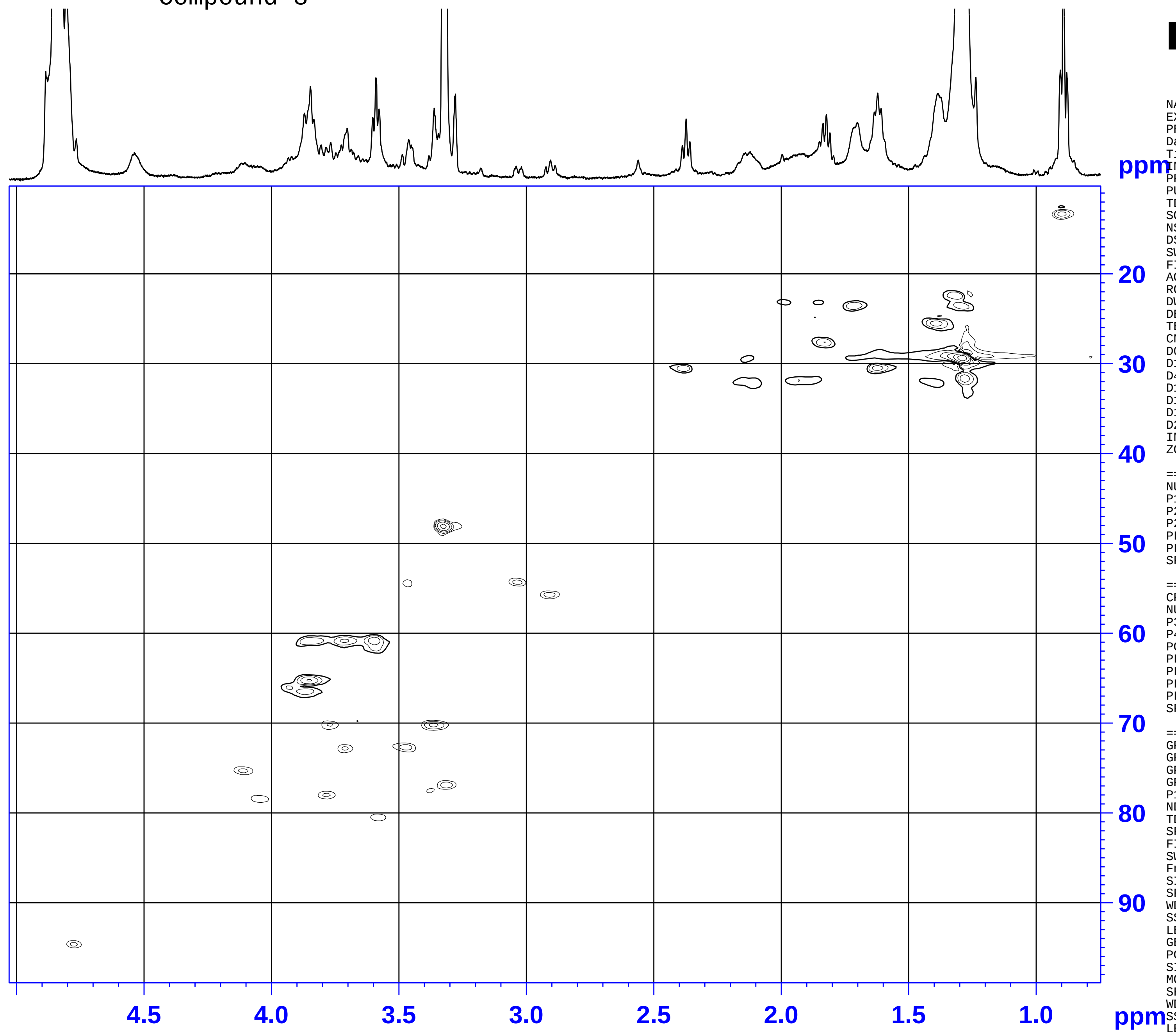

NAME	AC390P2	
EXPNO	3	
PROCNO	1	
Date_	20130725	
Time	21.15	
INSTRUM	AV500	
PROBHD	5 mm CPTXI 1H-	
PULPROG	hsqcedetgp	
TD	1024	
SOLVENT	MeOD	
NS	64	
DS	16	
SWH	6666.667	Hz
FIDRES	6.510417	Hz
AQ	0.0769250	sec
RG	18390.4	
DW	75.000	usec
DE	6.50	usec
TE	298.0	K
CNST2	145.0000000	
D0	0.00000300	sec
D1	1.50000000	sec
D4	0.00172414	sec
D11	0.03000000	sec
D13	0.00000400	sec
D16	0.00020000	sec
D21	0.00345000	sec
IN0	0.00002400	sec
ZGOPTNS		

======== CHANNEL f1 ========

NUC1	1H	
P1	7.70	usec
P2	15.40	usec
P28	0.10	usec
PL1	1.00	dB
PL1W	7.20289707	W
SFO1	500.1330069	MHz

======== CHANNEL f2 ========

CPDPRG2	garp	
NUC2	13C	
P3	14.00	usec
P4	28.00	usec
PCPD2	70.00	usec
PL2	-4.10	dB
PL12	9.88	dB
PL2W	105.88729095	W
PL12W	4.23490667	W
SFO2	125.7671682	MHz

====== GRADIENT CHANNEL =====

GPNAM1	SINE.100	
GPNAM2	SINE.100	
GPZ1	80.00	%
GPZ2	20.10	%
P16	1000.00	usec
ND0	2	
TD	256	
SFO1	125.7672	MHz
FIDRES	81.374924	Hz
SW	165.639	ppm
FnMODE	Echo-Antiecho	
SI	1024	
SF	500.1300000	MHz
WDW	QSINE	
SSB	2	
LB	0.00	Hz
GB	0	
PC	1.00	
SI	1024	
MC2	echo-antiecho	
SF	125.7577890	MHz
WDW	QSINE	
SSB	2	
LB	0.00	Hz
GB	0	

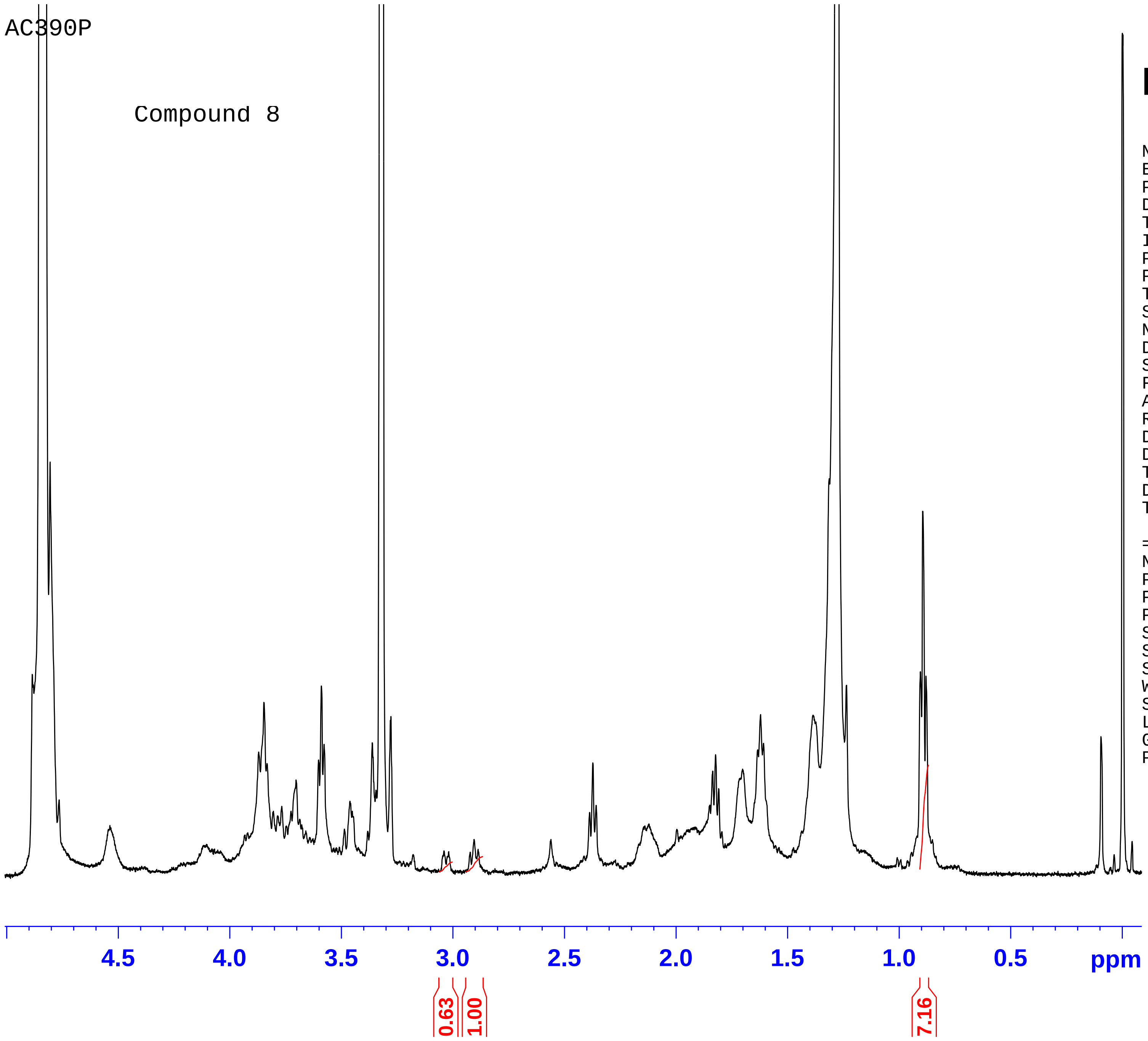

NAME	AC390P2
EXPNO	1
PROCNO	1
Date_	20130725
Time	15.33
INSTRUM	AV500
PROBHD	5 mm CPTXI 1H-
PULPROG	zg30
TD	65536
SOLVENT	MeOD
NS	128
DS	2
SWH	10330.578 Hz
FIDRES	0.157632 Hz
AQ	3.1720407 sec
RG	4
DW	48.400 usec
DE	6.50 usec
TE	298.0 K
D1	1.00000000 sec
TD0	1

======== CHANNEL f1 ========

NUC1	1H
P1	7.70 usec
PL1	1.00 dB
PL1W	7.20289707 W
SFO1	500.1330885 MHz
SI	32768
SF	500.1300059 MHz
WDW	EM
SSB	0
LB	0.30 Hz
GB	0
PC	1.00

AC448
C13CPD.d MeOD {C:\Bruker\TOPSPIN} AC 22

Compound 9

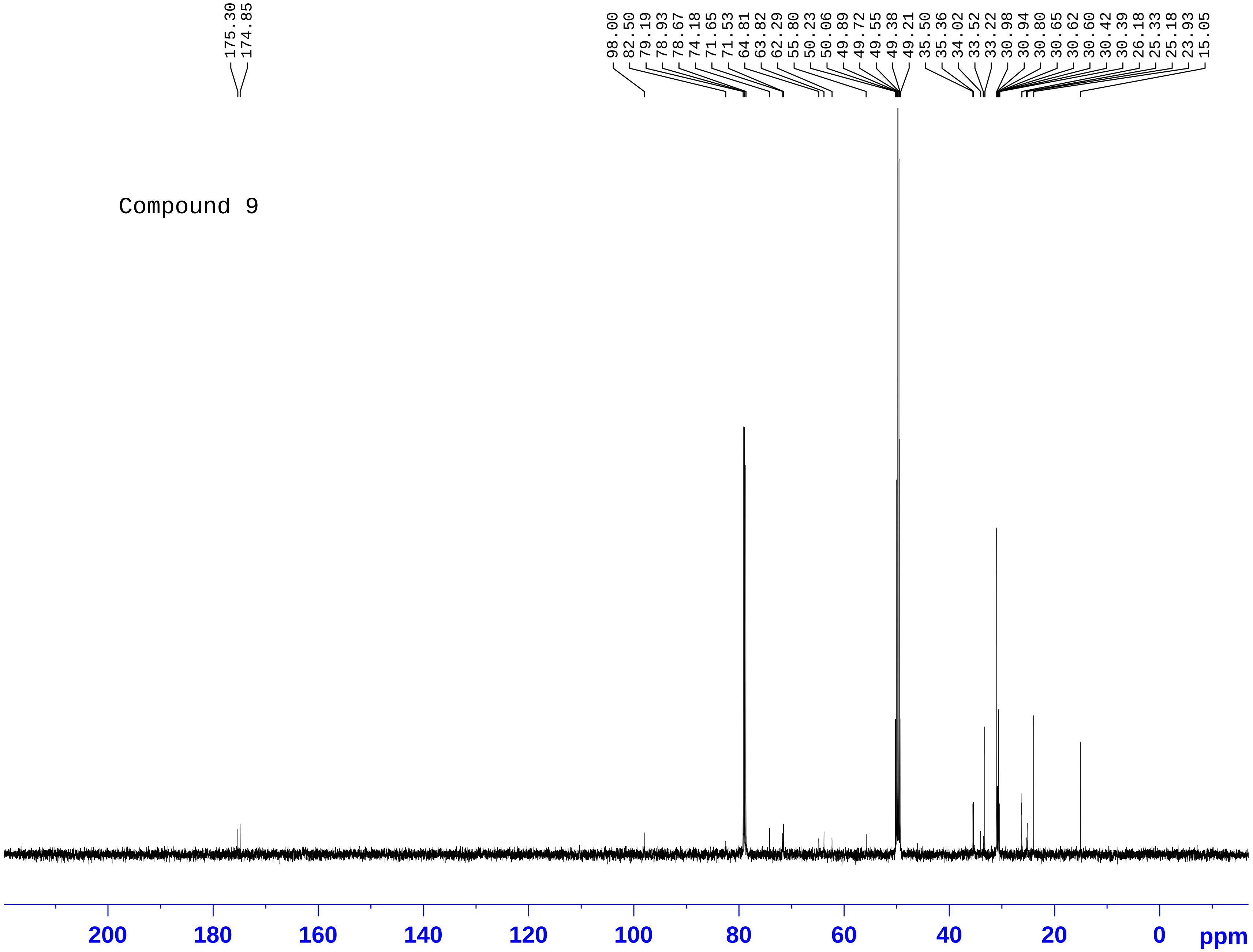

UNIVERSITY OF DUNDEE

NAME	AC-AC448	
EXPNO	8	
PROCNO	1	
Date_	20111209	
Time	15.16	
INSTRUM	spect	
PROBHD	5 mm QNP 1H/13	
PULPROG	zgpg30	
TD	65536	
SOLVENT	MeOD	
NS	256	
DS	4	
SWH	29761.904	Hz
FIDRES	0.454131	Hz
AQ	1.1010548	sec
RG	2050	
DW	16.800	usec
DE	6.00	usec
TE	294.8	K
D1	2.00000000	sec
d11	0.03000000	sec
DELTA	1.89999998	sec
TD0	1	

======== CHANNEL f1 ========

NUC1	13C	
P1	8.18	usec
PL1	0.00	dB
SFO1	125.7703643	MHz

======== CHANNEL f2 ========

CPDPRG2	waltz16	
NUC2	1H	
PCPD2	80.00	usec
PL2	-1.00	dB
PL12	16.00	dB
PL13	16.00	dB
SFO2	500.1320005	MHz
SI	32768	
SF	125.7576104	MHz
WDW	EM	
SSB	0	
LB	1.00	Hz
GB	0	
PC	1.40	

AC448
PROTON.d MeOD {C:\Bruker\TOPSPIN} AC 21

Compound 9

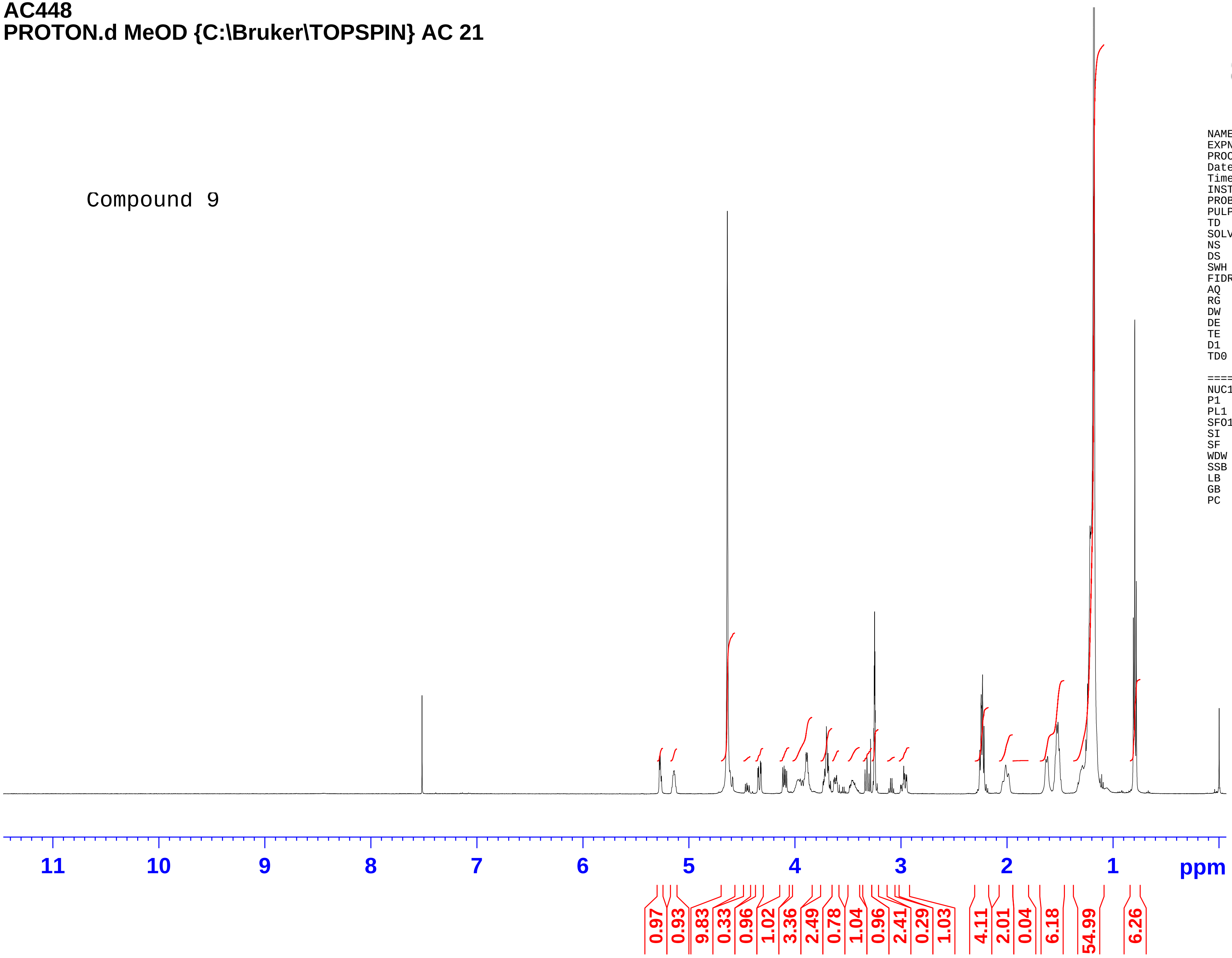

UNIVERSITY OF DUNDEE

NAME	AC-AC448	
EXPNO	3	
PROCNO	1	
Date_	20111209	
Time	11.47	
INSTRUM	spect	
PROBHD	5 mm QNP 1H/13	
PULPROG	zg30	
TD	65536	
SOLVENT	MeOD	
NS	16	
DS	2	
SWH	10330.578	Hz
FIDRES	0.157632	Hz
AQ	3.1719923	sec
RG	181	
DW	48.400	usec
DE	6.00	usec
TE	293.9	K
D1	1.00000000	sec
TD0	1	

======== CHANNEL f1 ========

NUC1	1H	
P1	11.20	usec
PL1	-1.00	dB
SFO1	500.1330885	MHz
SI	65536	
SF	500.1300416	MHz
WDW	EM	
SSB	0	
LB	0.30	Hz
GB	0	
PC	1.40	

AC447
C13CPDlong.d MeOD {C:\Bruker\TOPSPIN} AC 12

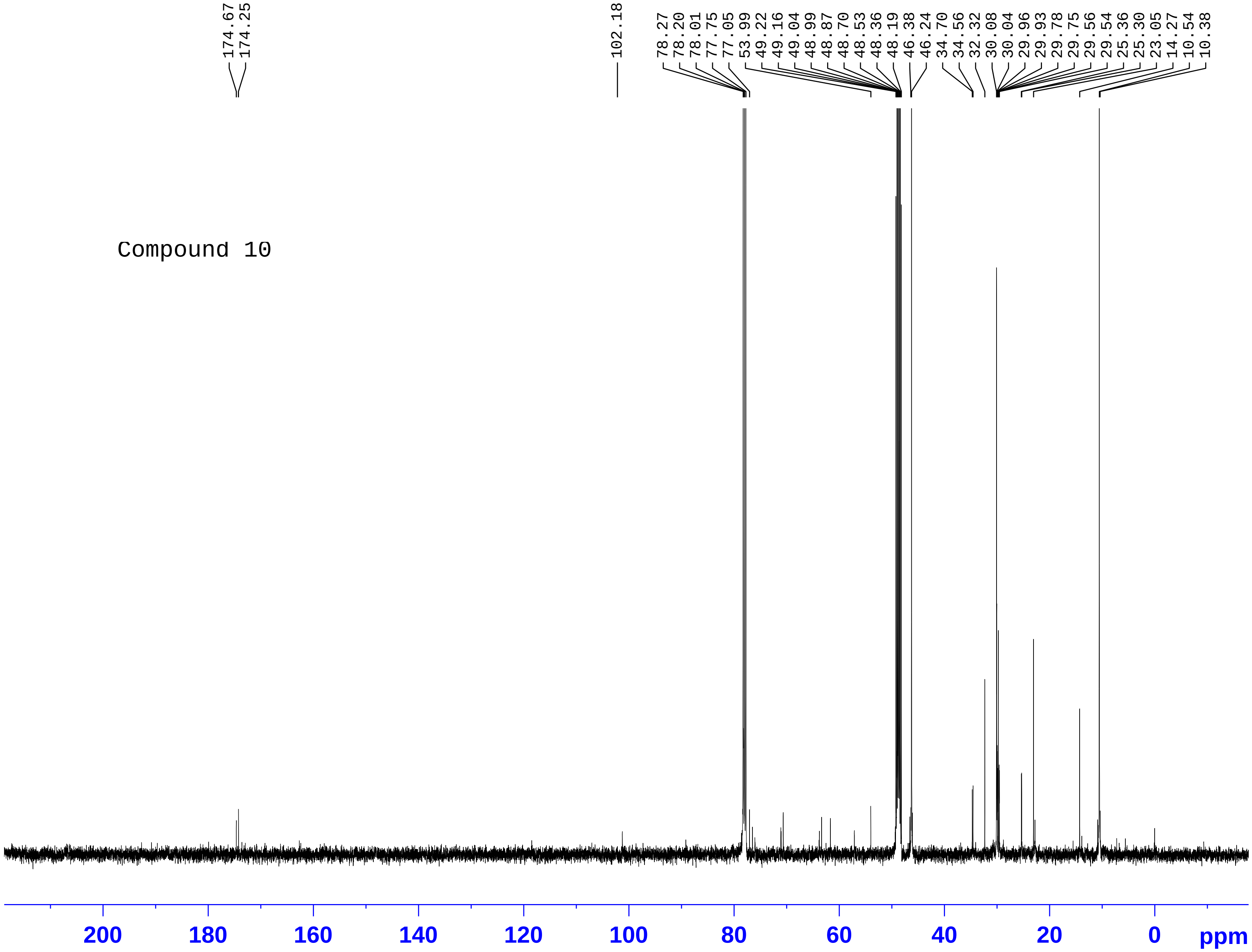

UNIVERSITY OF DUNDEE

NAME	AC-AC447	
EXPNO	9	
PROCNO	1	
Date_	20111216	
Time	0.01	
INSTRUM	spect	
PROBHD	5 mm QNP 1H/13	
PULPROG	zgpg30	
TD	65536	
SOLVENT	MeOD	
NS	8192	
DS	4	
SWH	29761.904	Hz
FIDRES	0.454131	Hz
AQ	1.1010548	sec
RG	2050	
DW	16.800	usec
DE	6.00	usec
TE	294.4	K
D1	2.00000000	sec
d11	0.03000000	sec
DELTA	1.89999998	sec
TD0	32	
======== CHANNEL f1 ========		
NUC1	13C	
P1	8.18	usec
PL1	0.00	dB
SFO1	125.7703643	MHz
======== CHANNEL f2 ========		
CPDPRG2	waltz16	
NUC2	1H	
PCPD2	80.00	usec
PL2	-1.00	dB
PL12	16.00	dB
PL13	16.00	dB
SFO2	500.1320005	MHz
SI	32768	
SF	125.7577279	MHz
WDW	EM	
SSB	0	
LB	1.00	Hz
GB	0	
PC	1.40	

AC447

Compound 10

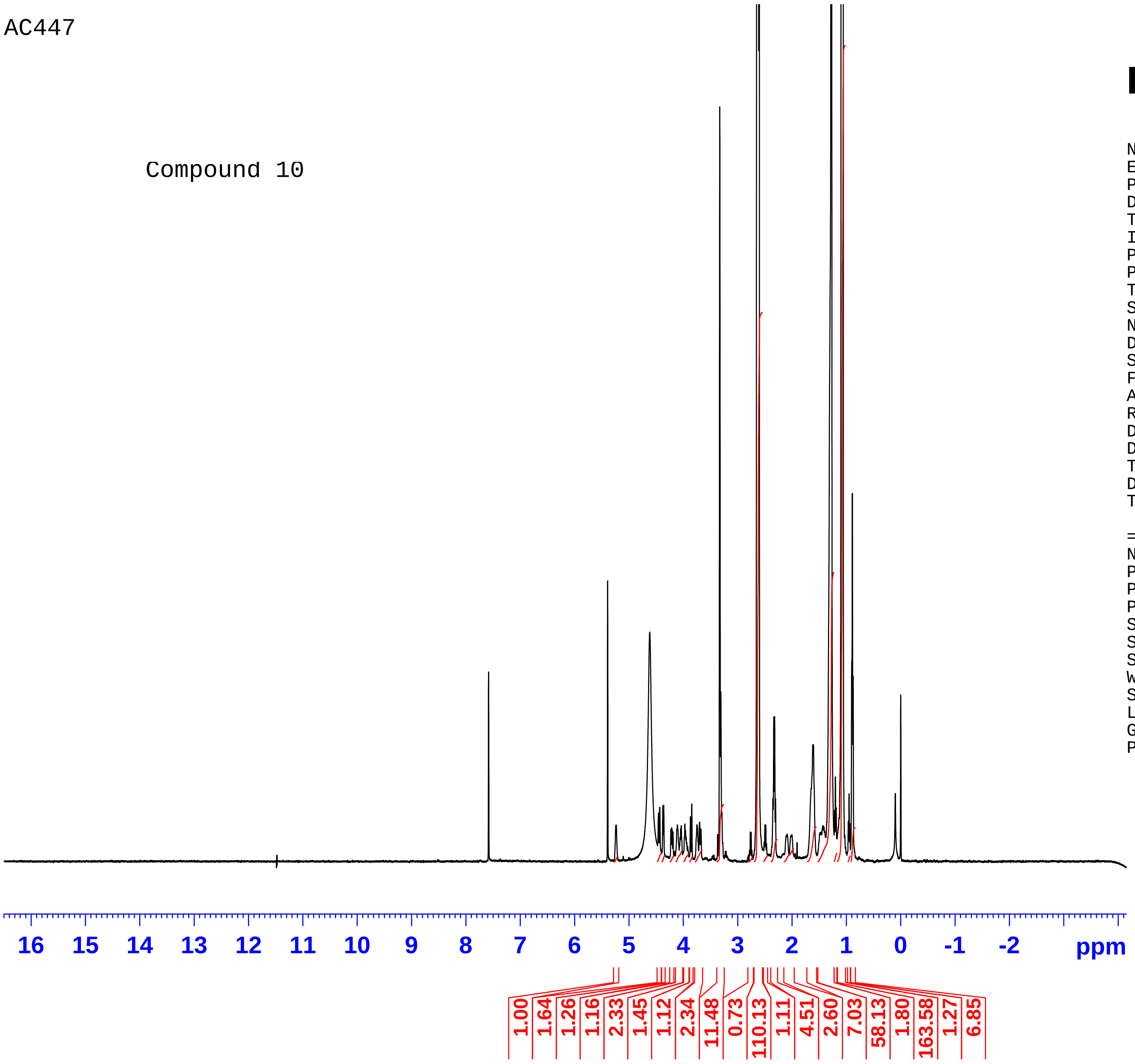

NAME	IHG-ATC447
EXPNO	1
PROCNO	1
Date_	20111213
Time	10.05
INSTRUM	AV500
PROBHD	5 mm CPTXI 1H-
PULPROG	zg30
TD	65536
SOLVENT	MeOD
NS	16
DS	2
SWH	10330.578 Hz
FIDRES	0.157632 Hz
AQ	3.1720407 sec
RG	4
DW	48.400 usec
DE	6.50 usec
TE	298.0 K
D1	1.00000000 sec
TD0	1

======== CHANNEL f1 ========

NUC1	1H
P1	7.70 usec
PL1	1.00 dB
PL1W	7.20289707 W
SFO1	500.1330885 MHz
SI	32768
SF	500.1300015 MHz
WDW	EM
SSB	0
LB	0.30 Hz
GB	0
PC	1.00

AC415 FR17-28
C13DEPT135f.d MeOD {C:\Bruker\TOPSPIN} AC 2

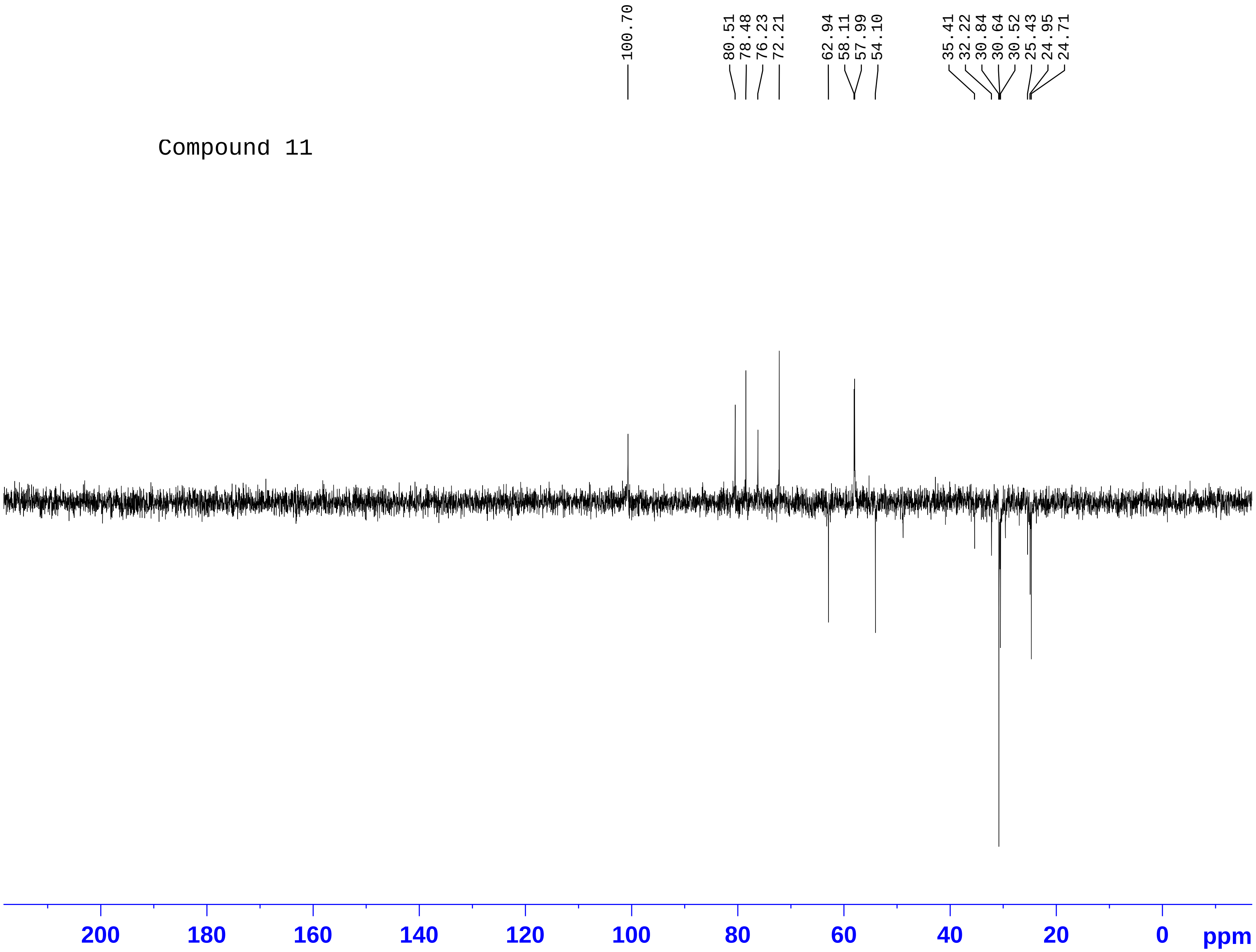

NAME	AC-AC415A
EXPNO	5
PROCNO	1
Date_	20100730
Time	10.52
INSTRUM	spect
PROBHD	5 mm QNP 1H/13
PULPROG	dept135
TD	17258
SOLVENT	MeOD
NS	800
DS	4
SWH	29761.904 Hz
FIDRES	1.724528 Hz
AQ	0.2899844 sec
RG	2050
DW	16.800 usec
DE	6.00 usec
TE	294.0 K
CNST2	145.0000000
D1	0.30000001 sec
d2	0.00344828 sec
d12	0.00002000 sec
DELTA	0.00000993 sec
TD0	1
======== CHANNEL f1 ========	
NUC1	13C
P1	7.80 usec
p2	15.60 usec
PL1	0.00 dB
SFO1	125.7703643 MHz
======== CHANNEL f2 ========	
CPDPRG2	waltz16
NUC2	1H
P3	12.00 usec
p4	24.00 usec
PCPD2	80.00 usec
PL2	-1.00 dB
PL12	16.00 dB
SFO2	500.1320005 MHz
SI	8192
SF	125.7576104 MHz
WDW	EM
SSB	0
LB	1.00 Hz
GB	0
PC	1.40

AC415 FR17-28
PROTON.d MeOD {C:\Bruker\TOPSPIN} AC 26

Compound 11

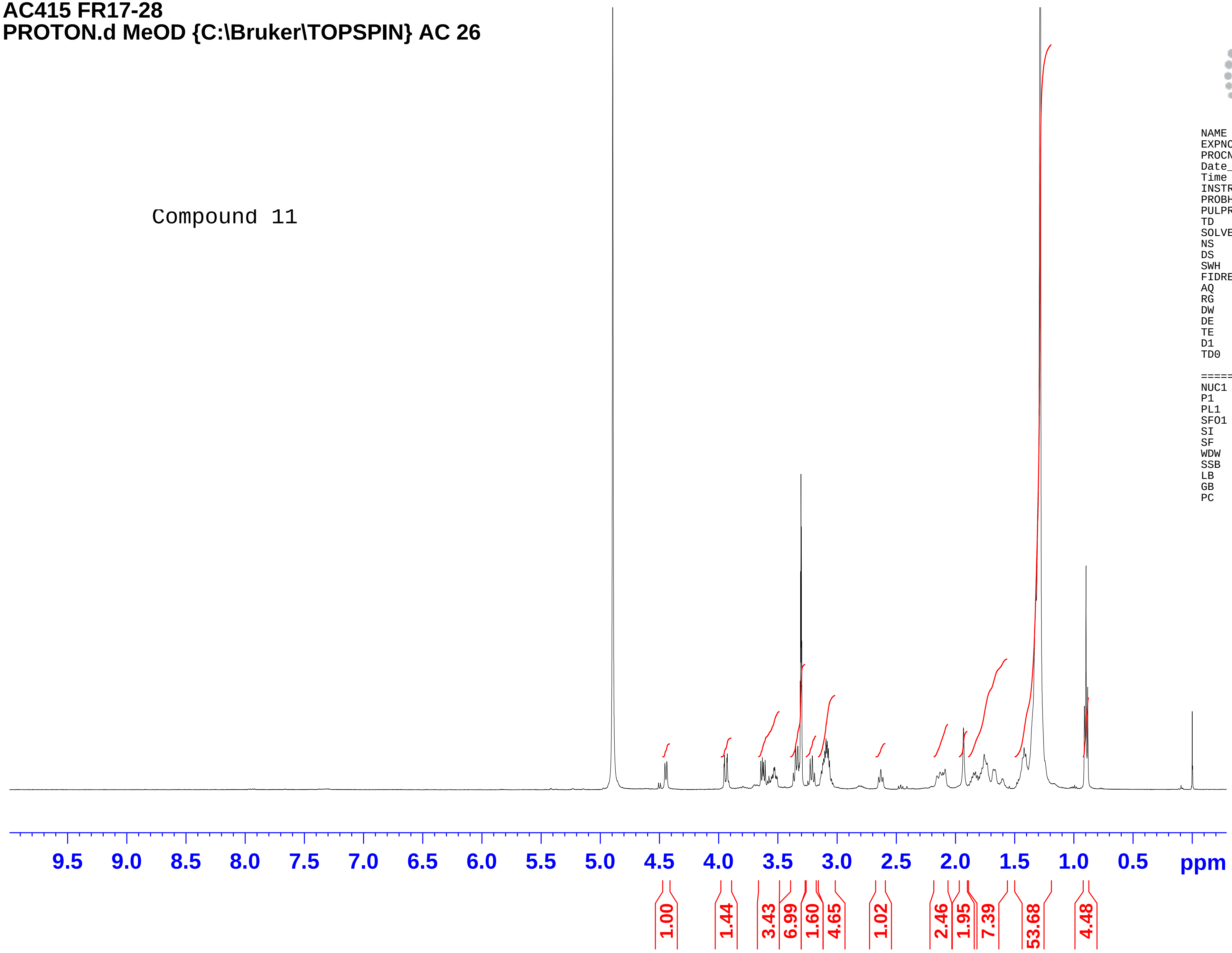

UNIVERSITY OF DUNDEE

```
NAME               AC-AC415A
EXPNO                      2
PROCNO                     1
Date_               20100730
Time                    9.33
INSTRUM                spect
PROBHD   5 mm QNP 1H/13
PULPROG                 zg30
TD                     65536
SOLVENT                 MeOD
NS                        16
DS                         2
SWH              10330.578 Hz
FIDRES            0.157632 Hz
AQ               3.1719923 sec
RG                       203
DW                    48.400 usec
DE                      6.00 usec
TE                     293.2 K
D1            1.00000000 sec
TD0                        1

======== CHANNEL f1 ========
NUC1                      1H
P1                     11.20 usec
PL1                    -1.00 dB
SFO1         500.1330885 MHz
SI                     65536
SF           500.1300138 MHz
WDW                       EM
SSB                        0
LB                      0.30 Hz
GB                         0
PC                      1.40
```

AC415B FR28-34

Compound 12

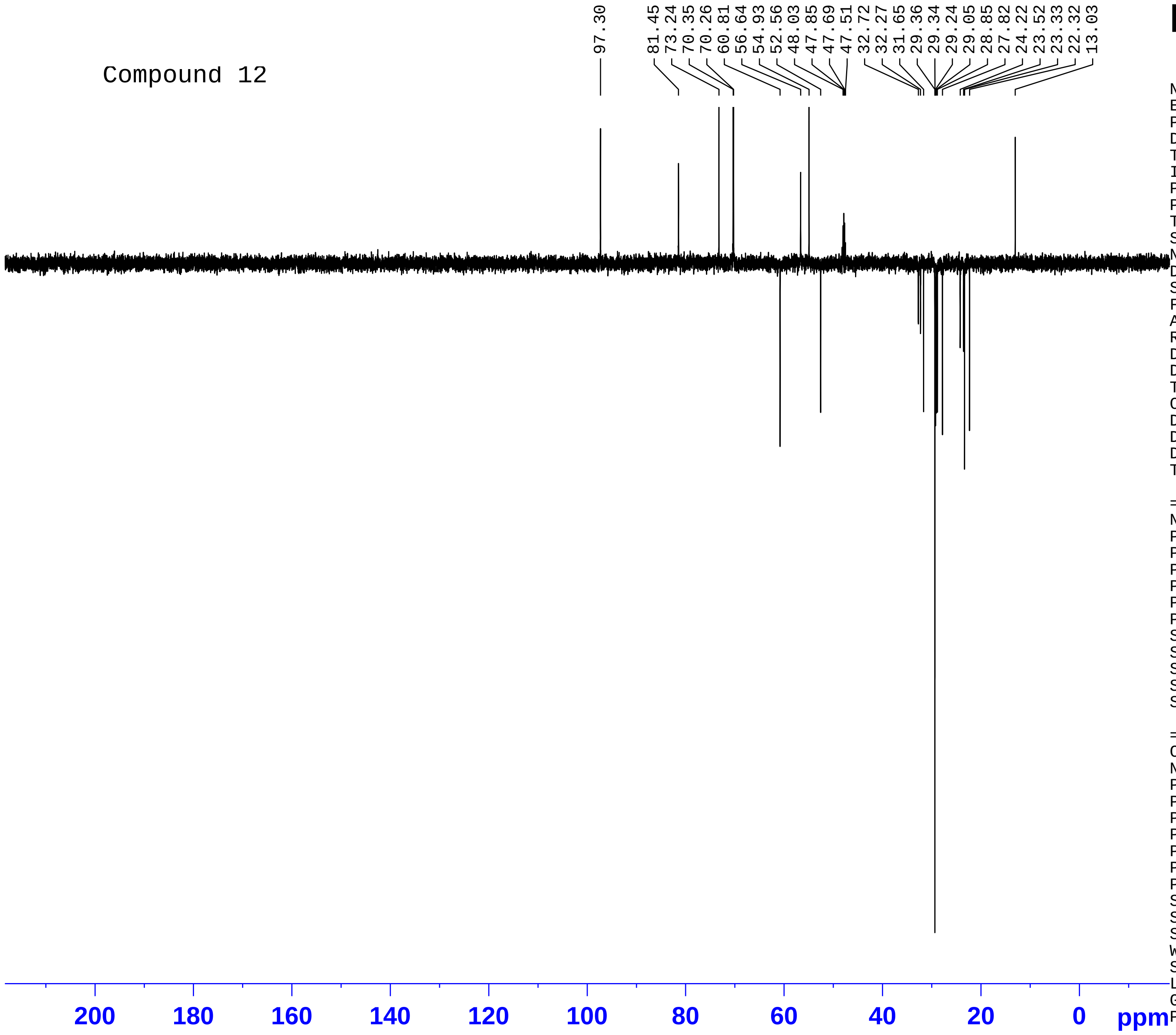

BRUKER

NAME	AC415BFR28-34	
EXPNO	4	
PROCNO	1	
Date_	20120413	
Time	15.50	
INSTRUM	AV500	
PROBHD	5 mm CPTXI 1H-	
PULPROG	deptsp135	
TD	65536	
SOLVENT	MeOD	
NS	512	
DS	4	
SWH	30030.029	Hz
FIDRES	0.458222	Hz
AQ	1.0912410	sec
RG	16384	
DW	16.650	usec
DE	6.50	usec
TE	298.0	K
CNST2	145.0000000	
D1	2.00000000	sec
D2	0.00344828	sec
D12	0.00002000	sec
TD0	1	

======== CHANNEL f1 ========

NUC1	13C	
P1	14.00	usec
P12	2000.00	usec
PL0	120.00	dB
PL1	-4.10	dB
PL0W	0.00000000	W
PL1W	105.88729095	W
SFO1	125.7703643	MHz
SP2	1.14	dB
SPNAM2	Crp60comp.4	
SPOAL2	0.500	
SPOFFS2	0.00	Hz

======== CHANNEL f2 ========

CPDPRG2	waltz16	
NUC2	1H	
P3	7.70	usec
P4	15.40	usec
PCPD2	80.00	usec
PL2	1.00	dB
PL12	21.33	dB
PL2W	7.20289707	W
PL12W	0.06675860	W
SFO2	500.1320005	MHz
SI	32768	
SF	125.7577933	MHz
WDW	EM	
SSB	0	
LB	1.00	Hz
GB	0	
PC	1.40	

AC415B FR28-34

Compound 12

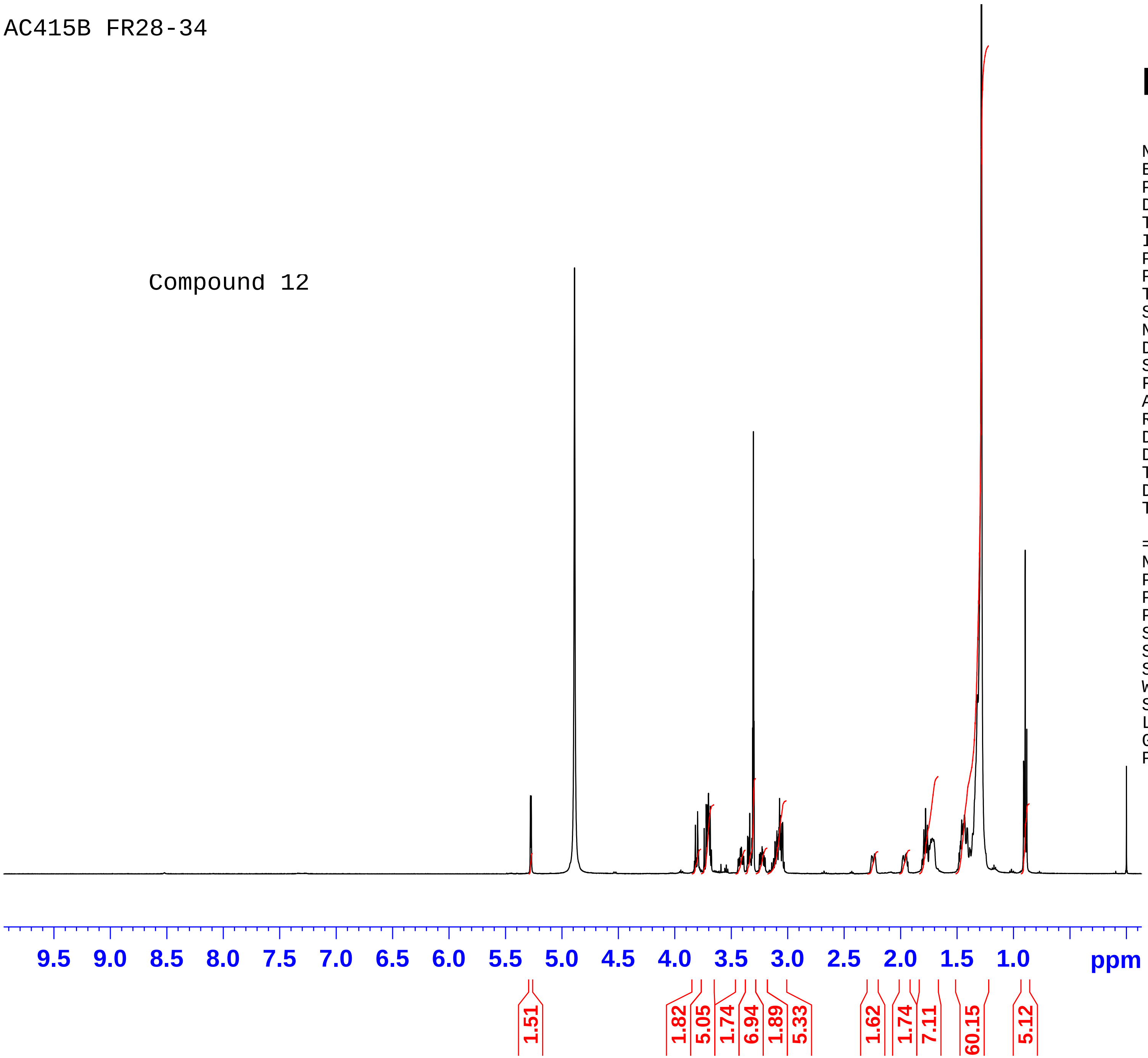

NAME	AC415BFR28-34
EXPNO	1
PROCNO	1
Date_	20120413
Time	14.32
INSTRUM	AV500
PROBHD	5 mm CPTXI 1H-
PULPROG	zg30
TD	65536
SOLVENT	MeOD
NS	16
DS	2
SWH	10330.578 Hz
FIDRES	0.157632 Hz
AQ	3.1720407 sec
RG	4
DW	48.400 usec
DE	6.50 usec
TE	298.0 K
D1	1.00000000 sec
TD0	1

======== CHANNEL f1 ========

NUC1	1H
P1	7.70 usec
PL1	1.00 dB
PL1W	7.20289707 W
SFO1	500.1330885 MHz
SI	32768
SF	500.1300134 MHz
WDW	EM
SSB	0
LB	0.30 Hz
GB	0
PC	1.00

N3 DEPRO
C13CPD6.d CDCl3 {C:\Bruker\TOPSPIN} IG 5

Compound 13

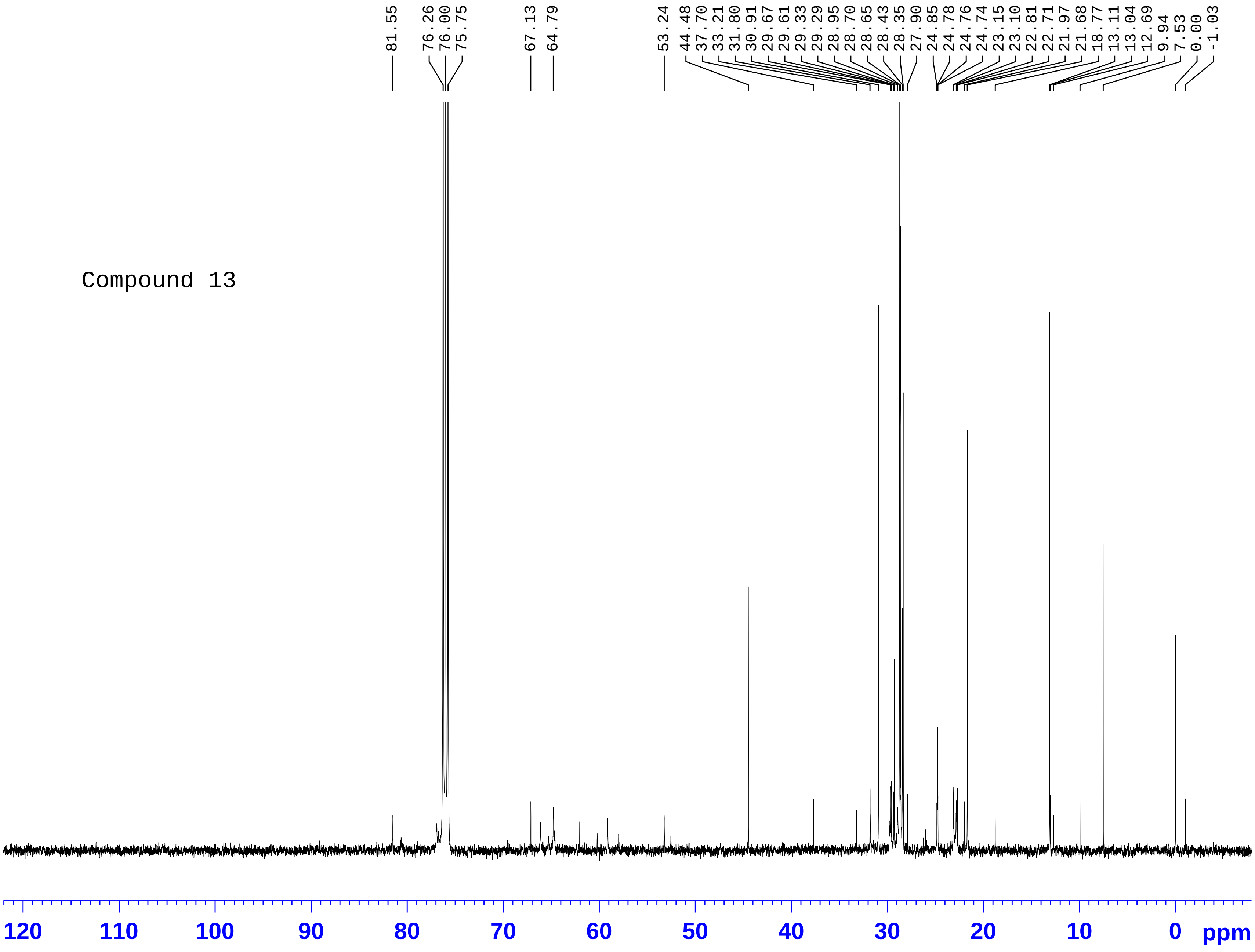

UNIVERSITY OF DUNDEE

NAME	IG-AC-N3DEPRO 1
EXPNO	6
PROCNO	1
Date_	20100519
Time	7.22
INSTRUM	spect
PROBHD	5 mm QNP 1H/13
PULPROG	zgpg30
TD	65536
SOLVENT	CDCl3
NS	6144
DS	4
SWH	29761.904 Hz
FIDRES	0.454131 Hz
AQ	1.1010548 sec
RG	2050
DW	16.800 usec
DE	6.00 usec
TE	295.1 K
D1	2.00000000 sec
d11	0.03000000 sec
DELTA	1.89999998 sec
TD0	1
======== CHANNEL f1 ========	
NUC1	13C
P1	7.80 usec
PL1	0.00 dB
SFO1	125.7703643 MHz
======== CHANNEL f2 ========	
CPDPRG2	waltz16
NUC2	1H
PCPD2	80.00 usec
PL2	-1.00 dB
PL12	16.00 dB
PL13	16.00 dB
SFO2	500.1320005 MHz
SI	32768
SF	125.7579184 MHz
WDW	EM
SSB	0
LB	1.00 Hz
GB	0
PC	1.40

N3 DEPRO
PROTON.d CDCl3 {C:\Bruker\TOPSPIN} IG 5

Compound 13

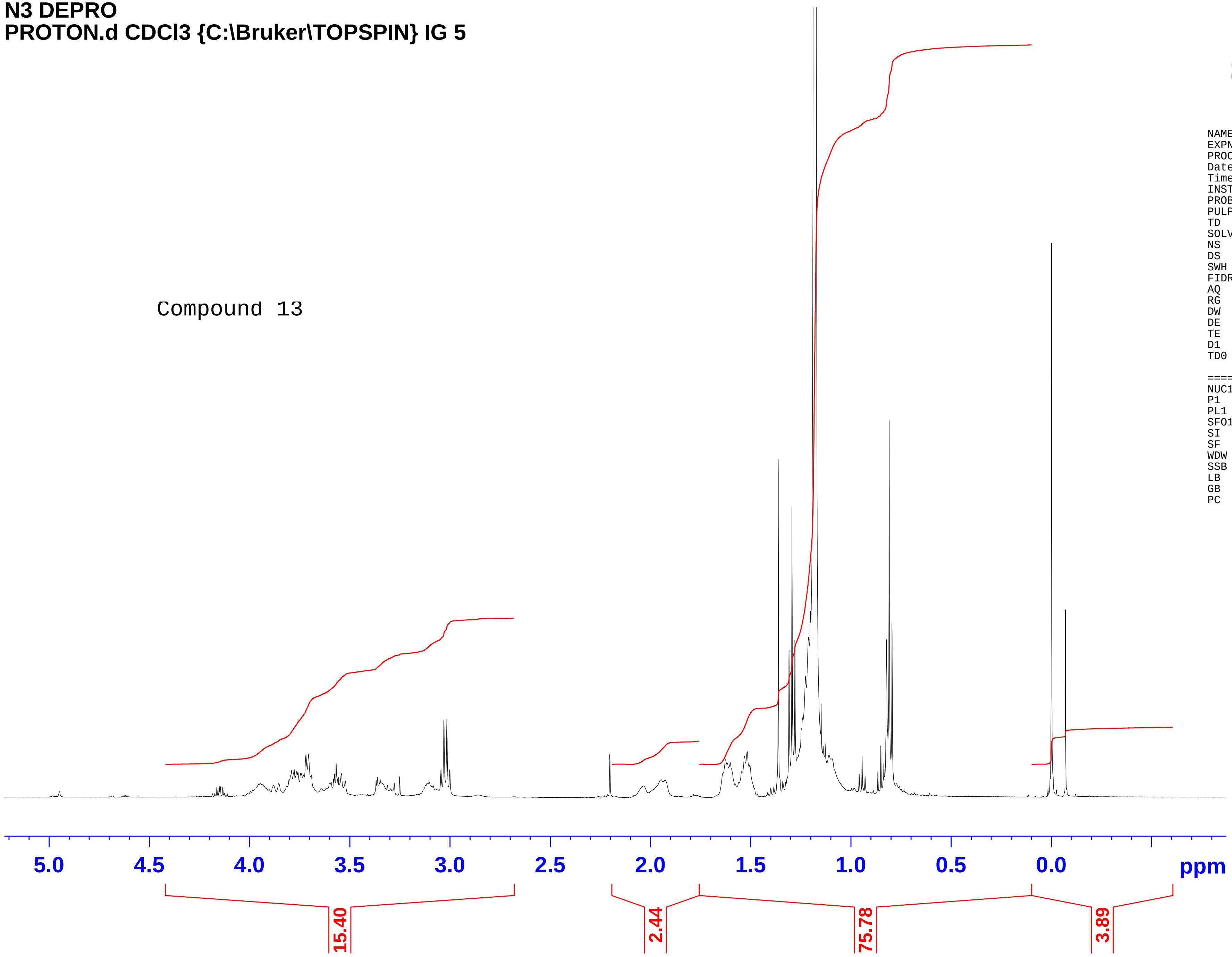

UNIVERSITY OF DUNDEE

```
NAME          IG-AC-N3DEPRO 1
EXPNO                     1
PROCNO                    1
Date_              20100518
Time                  10.45
INSTRUM               spect
PROBHD     5 mm QNP 1H/13
PULPROG                zg30
TD                    65536
SOLVENT               CDCl3
NS                       16
DS                        2
SWH              10330.578 Hz
FIDRES            0.157632 Hz
AQ               3.1719923 sec
RG                      181
DW                   48.400 usec
DE                     6.00 usec
TE                    293.8 K
D1               1.00000000 sec
TD0                       1

======== CHANNEL f1 ========
NUC1                     1H
P1                    11.20 usec
PL1                   -1.00 dB
SFO1            500.1330885 MHz
SI                    65536
SF              500.1300446 MHz
WDW                      EM
SSB                       0
LB                     0.30 Hz
GB                        0
PC                     1.40
```

AC422 FR17-20
C13CPDfast.d CDCl3 {C:\Bruker\TOPSPIN} AC 18

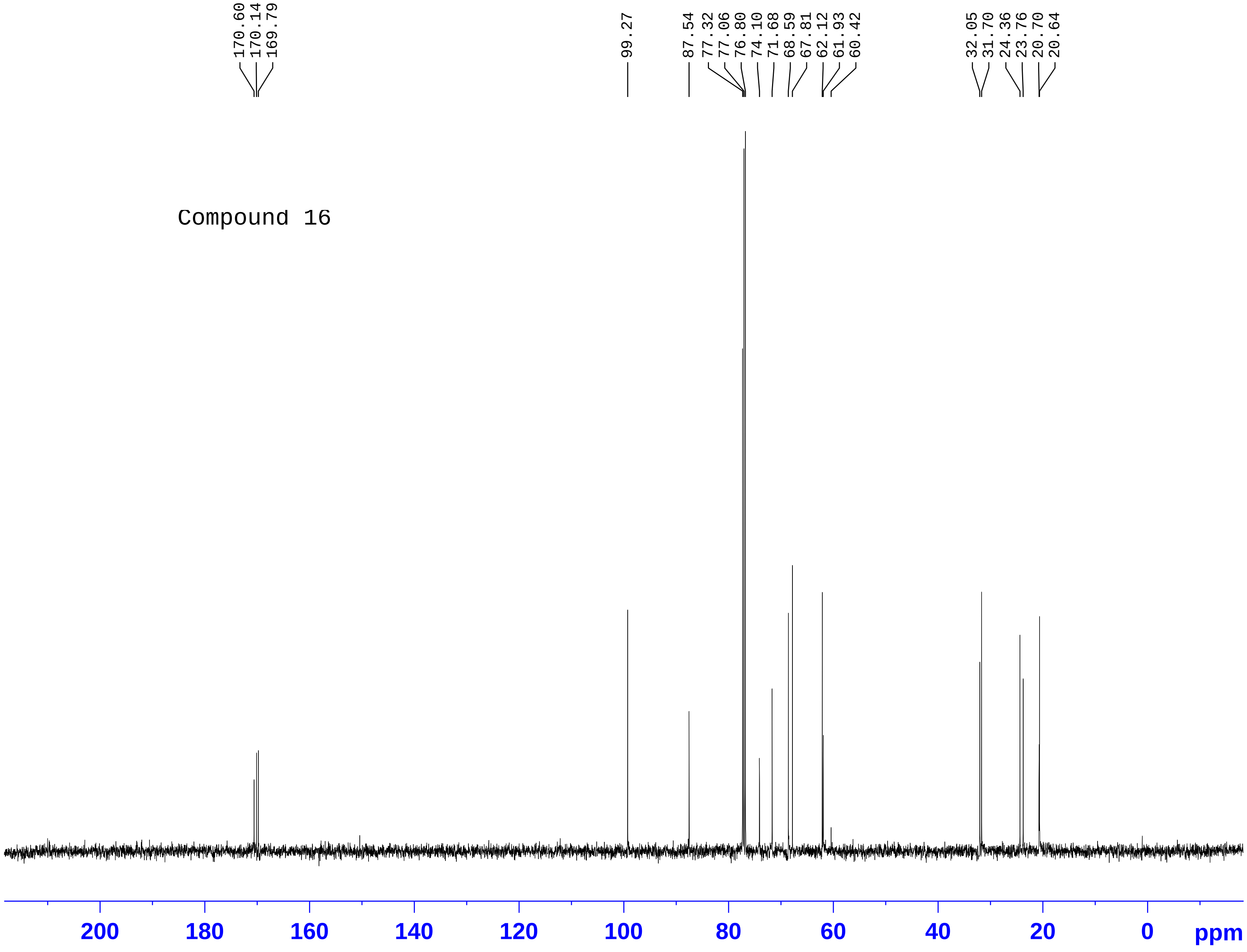

UNIVERSITY OF DUNDEE

```
NAME       AC-AC422F17-20
EXPNO                   4
PROCNO                  1
Date_            20101210
Time                12.20
INSTRUM             spect
PROBHD   5 mm QNP 1H/13
PULPROG           zgpg30
TD                 16384
SOLVENT            CDCl3
NS                   800
DS                     4
SWH           29761.904 Hz
FIDRES         1.816522 Hz
AQ            0.2753012 sec
RG                  2050
DW                16.800 usec
DE                  6.00 usec
TE                 293.0 K
D1            0.30000001 sec
d11           0.03000000 sec
DELTA         0.20000002 sec
TD0                    1

======== CHANNEL f1 ========
NUC1                 13C
P1                  7.80 usec
PL1                 0.00 dB
SFO1         125.7703643 MHz

======== CHANNEL f2 ========
CPDPRG2          waltz16
NUC2                  1H
PCPD2              80.00 usec
PL2                -1.00 dB
PL12               16.00 dB
PL13               16.00 dB
SFO2         500.1320005 MHz
SI                  8192
SF           125.7577890 MHz
WDW                   EM
SSB                    0
LB                  1.00 Hz
GB                     0
PC                  1.40
```

AC422 FR17-20
PROTON.d CDCl3 {C:\Bruker\TOPSPIN} AC 6

Compound 16

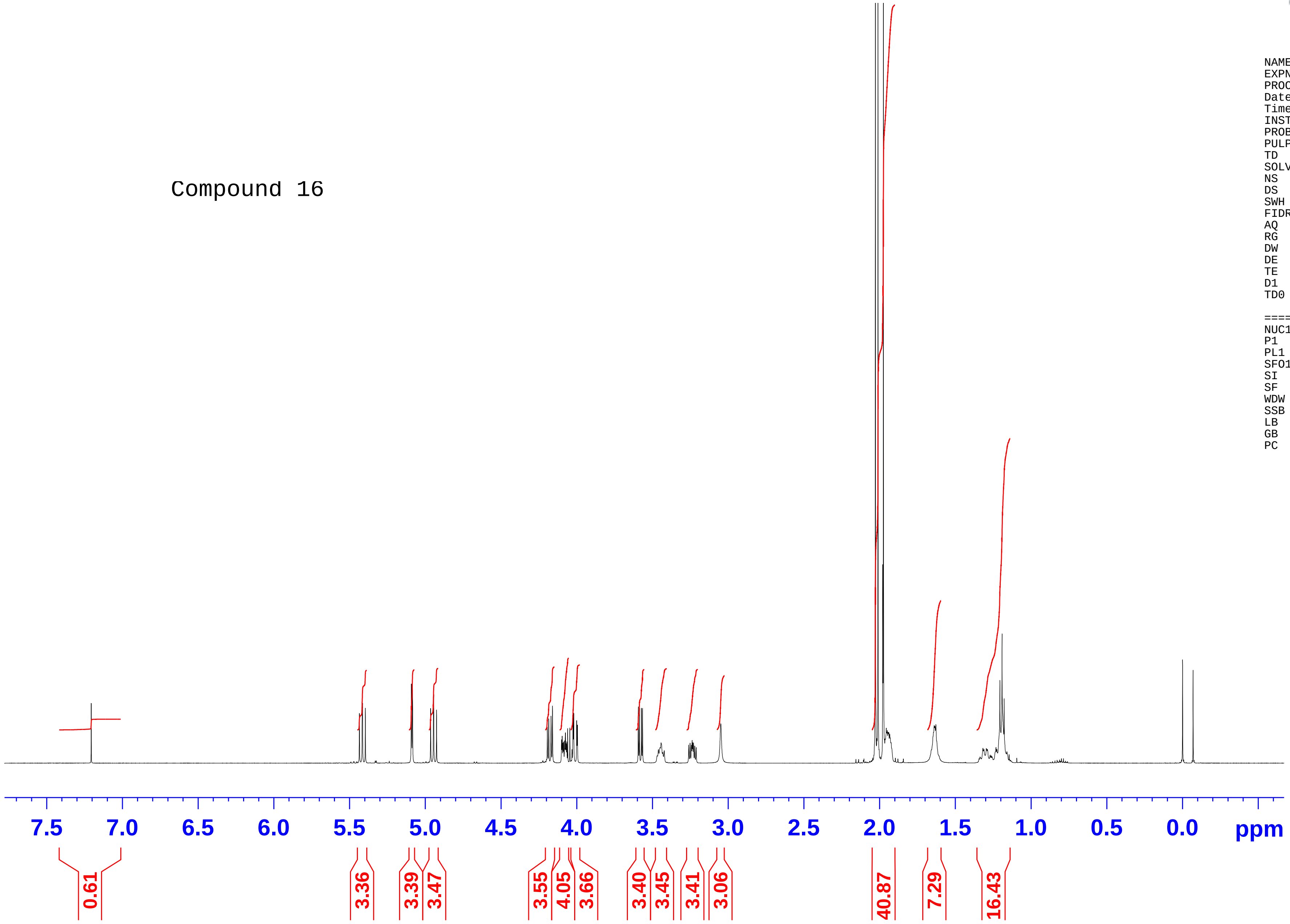

UNIVERSITY OF DUNDEE

NAME	AC-AC422F17-20
EXPNO	1
PROCNO	1
Date_	20101210
Time	10.52
INSTRUM	spect
PROBHD	5 mm QNP 1H/13
PULPROG	zg30
TD	65536
SOLVENT	CDCl3
NS	16
DS	2
SWH	10330.578 Hz
FIDRES	0.157632 Hz
AQ	3.1719923 sec
RG	228
DW	48.400 usec
DE	6.00 usec
TE	291.9 K
D1	1.00000000 sec
TD0	1

======== CHANNEL f1 ========

NUC1	1H
P1	11.20 usec
PL1	-1.00 dB
SFO1	500.1330885 MHz
SI	65536
SF	500.1300407 MHz
WDW	EM
SSB	0
LB	0.30 Hz
GB	0
PC	1.40

AC422 FR23-30
C13CPDfast.d CDCl3 {C:\Bruker\TOPSPIN} AC 23

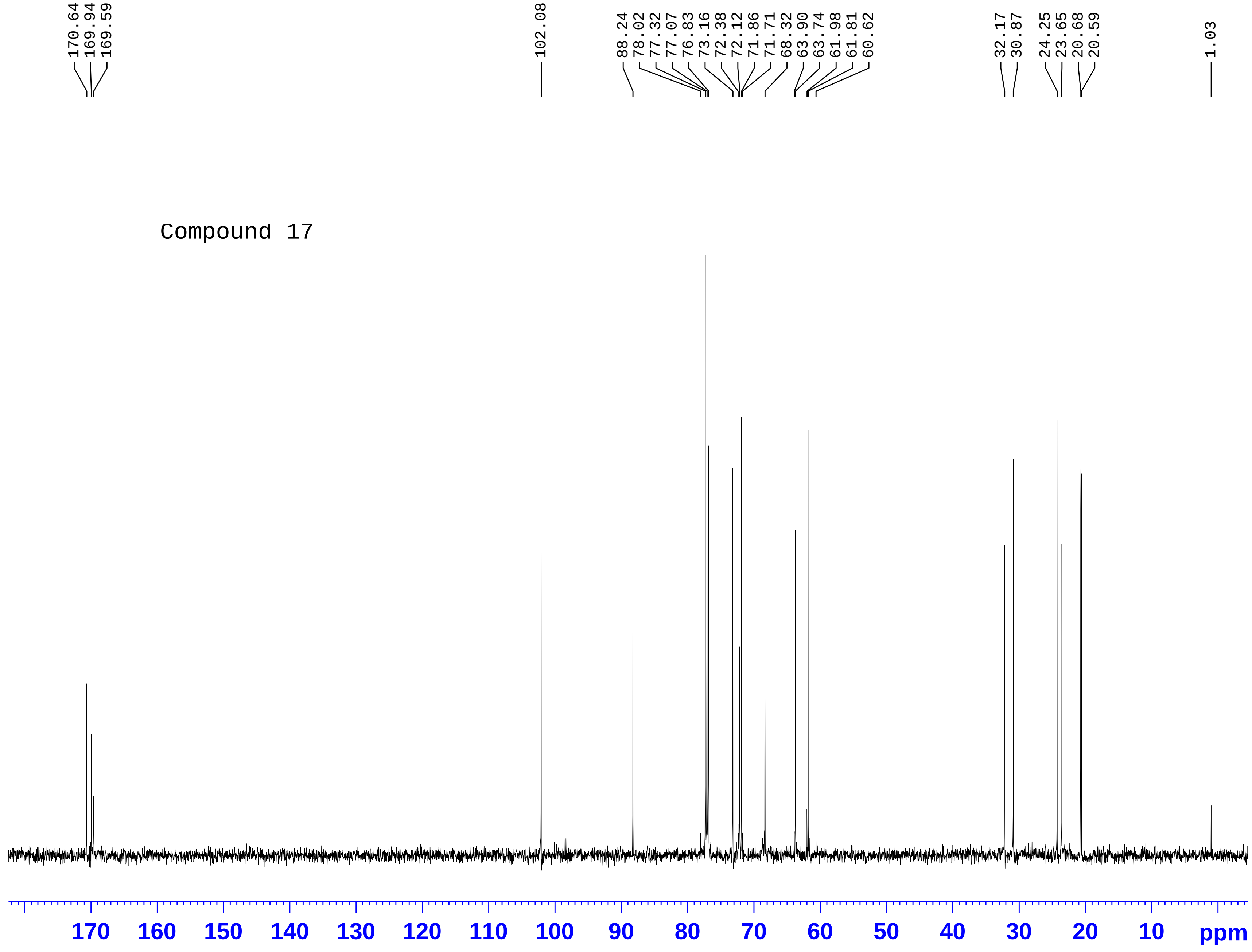


```
NAME      AC-AC422F23-30
EXPNO                  5
PROCNO                 1
Date_           20101210
Time               14.46
INSTRUM            spect
PROBHD   5 mm QNP 1H/13
PULPROG          zgpg30
TD                16384
SOLVENT            CDCl3
NS                   800
DS                     4
SWH            29761.904 Hz
FIDRES          1.816522 Hz
AQ             0.2753012 sec
RG                  2050
DW                16.800 usec
DE                  6.00 usec
TE                 293.0 K
D1            0.30000001 sec
d11           0.03000000 sec
DELTA         0.20000002 sec
TD0                    1

======== CHANNEL f1 ========
NUC1                 13C
P1                  7.80 usec
PL1                 0.00 dB
SFO1         125.7703643 MHz

======== CHANNEL f2 ========
CPDPRG2          waltz16
NUC2                  1H
PCPD2              80.00 usec
PL2                -1.00 dB
PL12               16.00 dB
PL13               16.00 dB
SFO2         500.1320005 MHz
SI                  8192
SF           125.7577866 MHz
WDW                   EM
SSB                    0
LB                  1.00 Hz
GB                     0
PC                  1.40
```

AC422 FR23-30
PROTON.d CDCl3 {C:\Bruker\TOPSPIN} AC 23

Compound 17

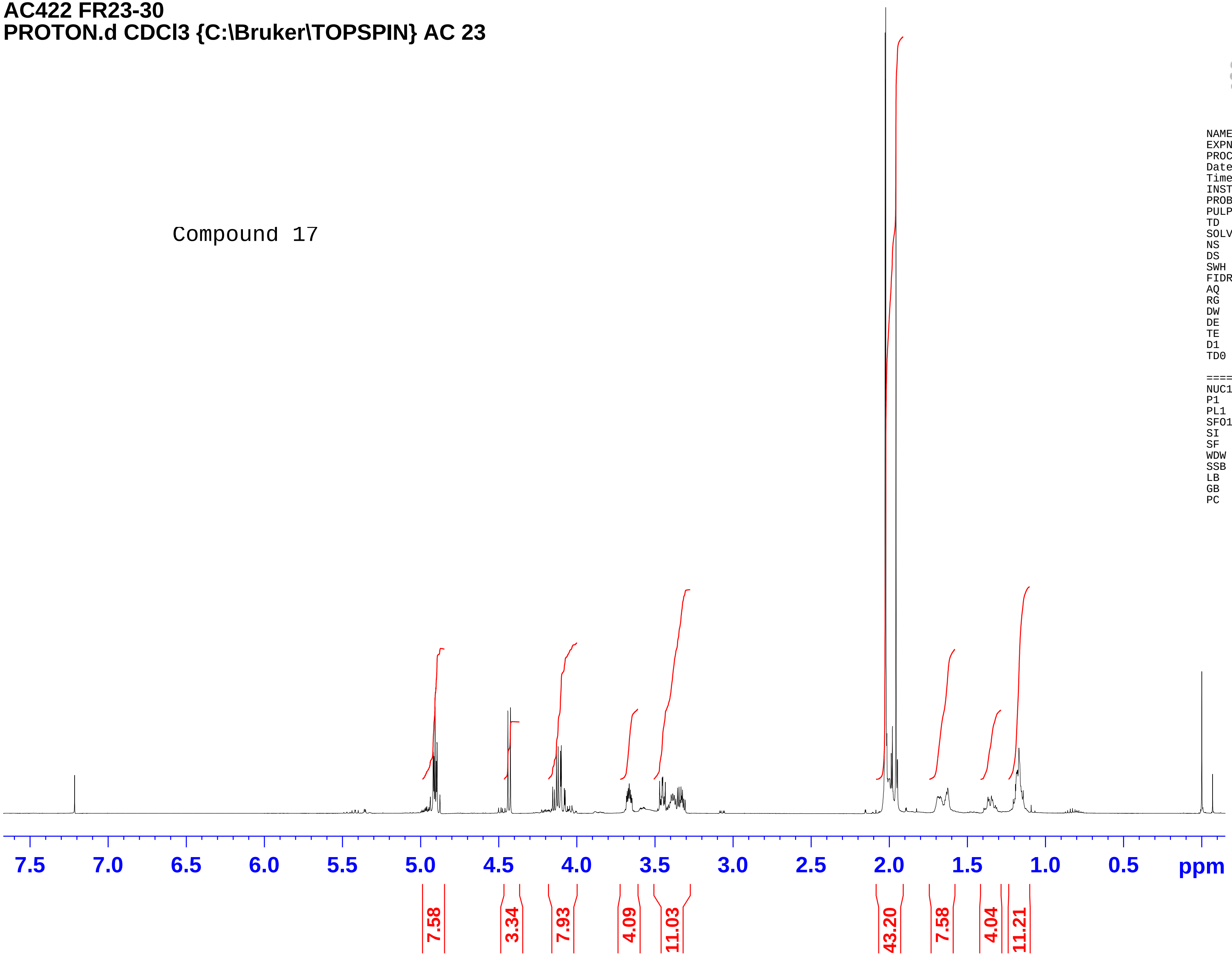

UNIVERSITY OF DUNDEE

NAME	AC-AC422F23-30
EXPNO	2
PROCNO	1
Date_	20101210
Time	14.17
INSTRUM	spect
PROBHD	5 mm QNP 1H/13
PULPROG	zg30
TD	65536
SOLVENT	CDCl3
NS	16
DS	2
SWH	10330.578 Hz
FIDRES	0.157632 Hz
AQ	3.1719923 sec
RG	144
DW	48.400 usec
DE	6.00 usec
TE	292.1 K
D1	1.00000000 sec
TD0	1

======== CHANNEL f1 ========

NUC1	1H
P1	11.20 usec
PL1	-1.00 dB
SFO1	500.1330885 MHz
SI	65536
SF	500.1300362 MHz
WDW	EM
SSB	0
LB	0.30 Hz
GB	0
PC	1.40

AC423 REPURIFIED
C13CPDfast.d CDCl3 {C:\Bruker\TOPSPIN} AC 1

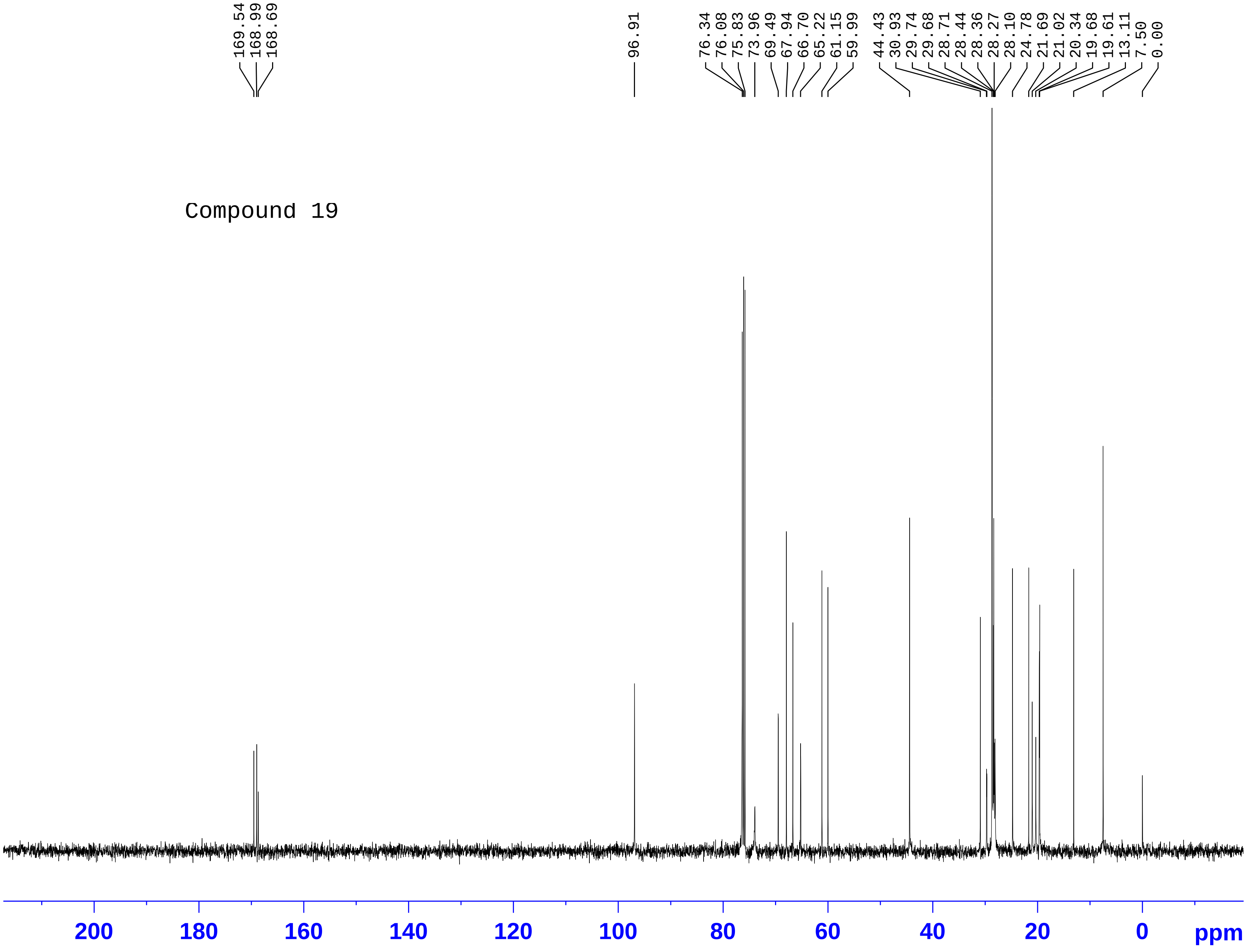

NAME	AC-AC423R
EXPNO	5
PROCNO	1
Date_	20110426
Time	10.12
INSTRUM	spect
PROBHD	5 mm QNP 1H/13
PULPROG	zgpg30
TD	16384
SOLVENT	CDCl3
NS	800
DS	4
SWH	29761.904 Hz
FIDRES	1.816522 Hz
AQ	0.2753012 sec
RG	2050
DW	16.800 usec
DE	6.00 usec
TE	300.2 K
D1	0.30000001 sec
d11	0.03000000 sec
DELTA	0.20000002 sec
TD0	1

======== CHANNEL f1 ========

NUC1	13C
P1	7.80 usec
PL1	0.00 dB
SFO1	125.7703643 MHz

======== CHANNEL f2 ========

CPDPRG2	waltz16
NUC2	1H
PCPD2	80.00 usec
PL2	-1.00 dB
PL12	16.00 dB
PL13	16.00 dB
SFO2	500.1320005 MHz
SI	8192
SF	125.7579114 MHz
WDW	EM
SSB	0
LB	1.00 Hz
GB	0
PC	1.40

AC423 REPURIFIED
PROTON.d CDCl3 {C:\Bruker\TOPSPIN} AC 44

Compound 19

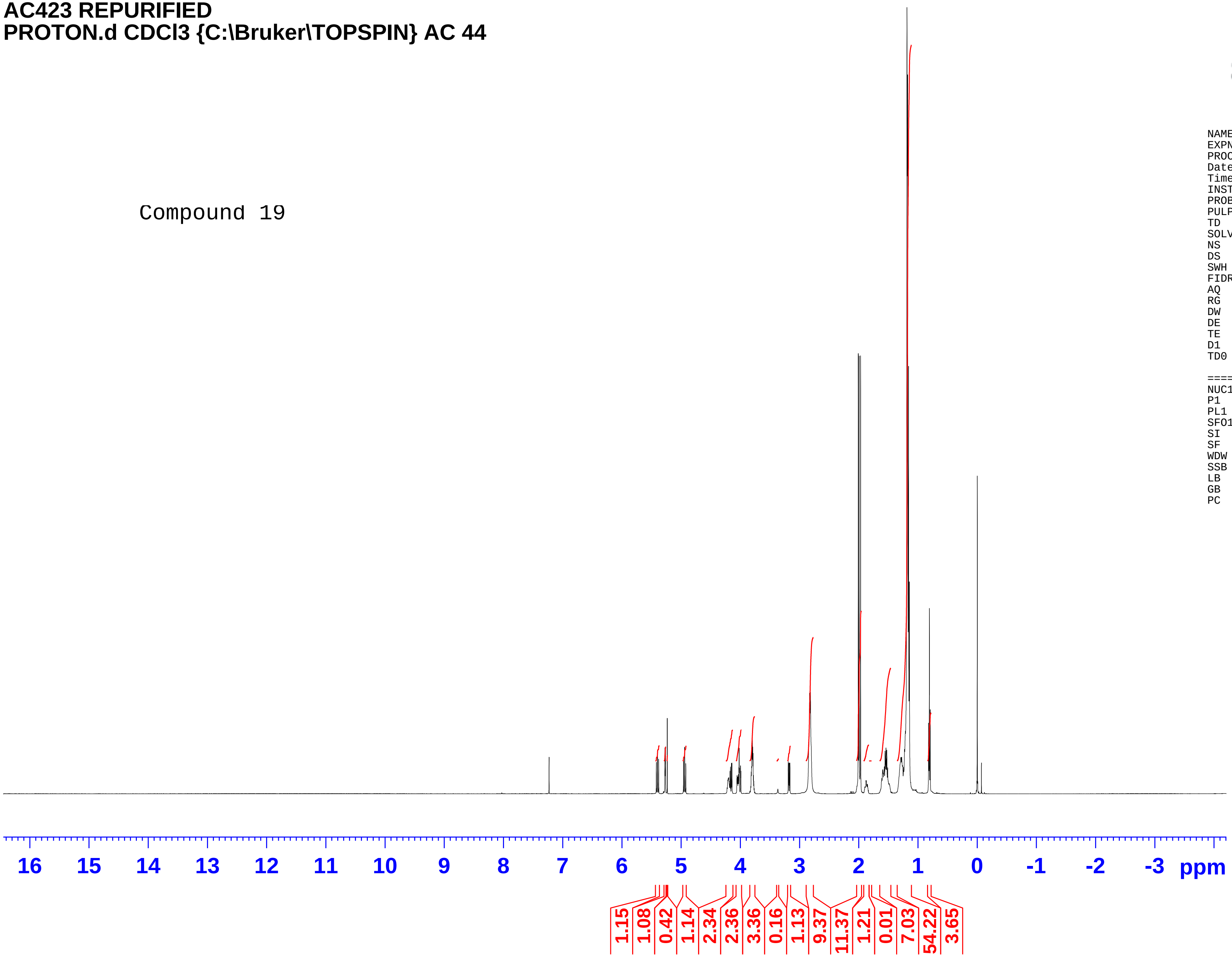

UNIVERSITY OF DUNDEE

```
NAME                AC-AC423R
EXPNO                       1
PROCNO                      1
Date_                20110425
Time                    16.26
INSTRUM                 spect
PROBHD   5 mm QNP 1H/13
PULPROG                  zg30
TD                      65536
SOLVENT                 CDCl3
NS                         16
DS                          2
SWH                 10330.578 Hz
FIDRES               0.157632 Hz
AQ                  3.1719923 sec
RG                         57
DW                     48.400 usec
DE                       6.00 usec
TE                      300.2 K
D1                 1.00000000 sec
TD0                         1

======== CHANNEL f1 ========
NUC1                       1H
P1                      11.20 usec
PL1                     -1.00 dB
SFO1              500.1330885 MHz
SI                      65536
SF                500.1300276 MHz
WDW                        EM
SSB                         0
LB                       0.30 Hz
GB                          0
PC                       1.40
```

AC424
C13CPDfast.d MeOD {C:\Bruker\TOPSPIN} AC 52

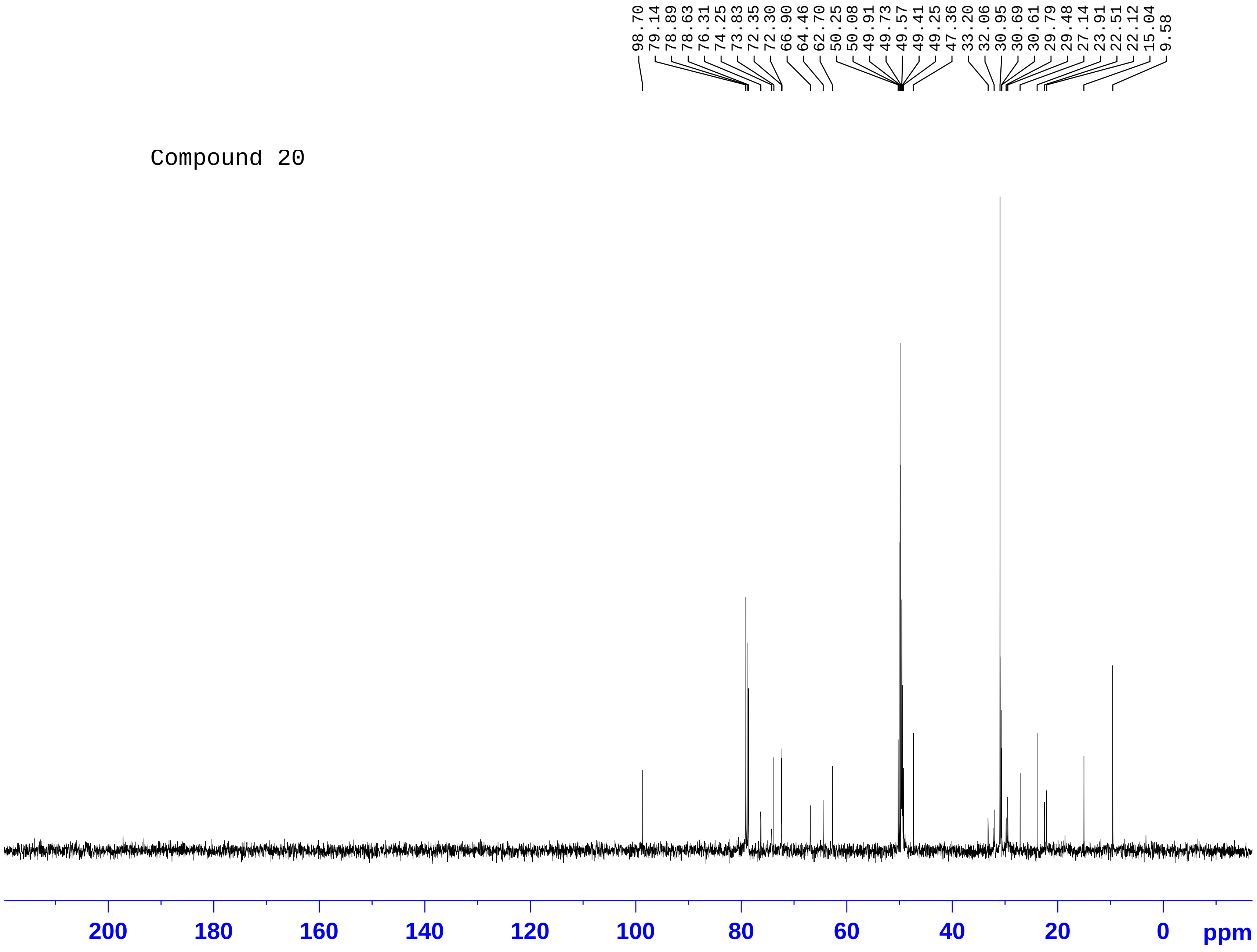

UNIVERSITY OF DUNDEE

NAME	AC-AC424	
EXPNO	4	
PROCNO	1	
Date_	20110505	
Time	15.51	
INSTRUM	spect	
PROBHD	5 mm QNP 1H/13	
PULPROG	zgpg30	
TD	16384	
SOLVENT	MeOD	
NS	800	
DS	4	
SWH	29761.904	Hz
FIDRES	1.816522	Hz
AQ	0.2753012	sec
RG	2050	
DW	16.800	usec
DE	6.00	usec
TE	300.2	K
D1	0.30000001	sec
d11	0.03000000	sec
DELTA	0.20000002	sec
TD0	1	

======== CHANNEL f1 ========

NUC1	13C	
P1	7.80	usec
PL1	0.00	dB
SFO1	125.7703643	MHz

======== CHANNEL f2 ========

CPDPRG2	waltz16	
NUC2	1H	
PCPD2	80.00	usec
PL2	-1.00	dB
PL12	16.00	dB
PL13	16.00	dB
SFO2	500.1320005	MHz
SI	8192	
SF	125.7576104	MHz
WDW	EM	
SSB	0	
LB	1.00	Hz
GB	0	
PC	1.40	

AC424
PROTON.d MeOD {C:\Bruker\TOPSPIN} AC 52

Compound 20

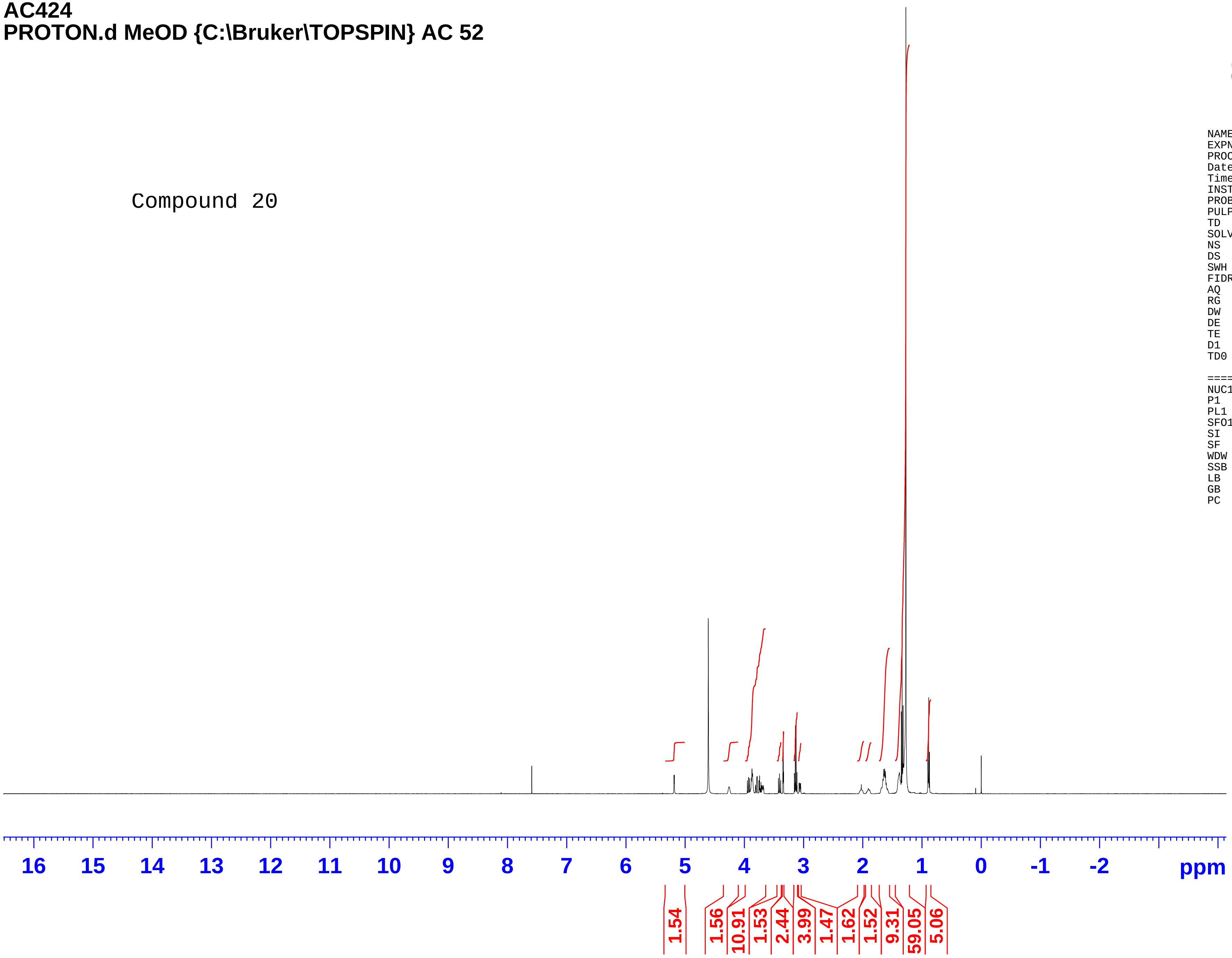

UNIVERSITY OF DUNDEE

```
NAME               AC-AC424
EXPNO                     2
PROCNO                    1
Date_              20110505
Time                  15.31
INSTRUM               spect
PROBHD    5 mm QNP 1H/13
PULPROG                zg30
TD                    65536
SOLVENT                MeOD
NS                       16
DS                        2
SWH              10330.578 Hz
FIDRES            0.157632 Hz
AQ               3.1719923 sec
RG                      203
DW                   48.400 usec
DE                     6.00 usec
TE                    300.2 K
D1               1.00000000 sec
TD0                       1

======== CHANNEL f1 ========
NUC1                     1H
P1                    11.20 usec
PL1                   -1.00 dB
SFO1            500.1330885 MHz
SI                    65536
SF              500.1299937 MHz
WDW                      EM
SSB                       0
LB                     0.30 Hz
GB                        0
PC                     1.40
```

AC425FR2-6
C13CPDfast.d MeOD {C:\Bruker\TOPSPIN} AC 1

Compound 21

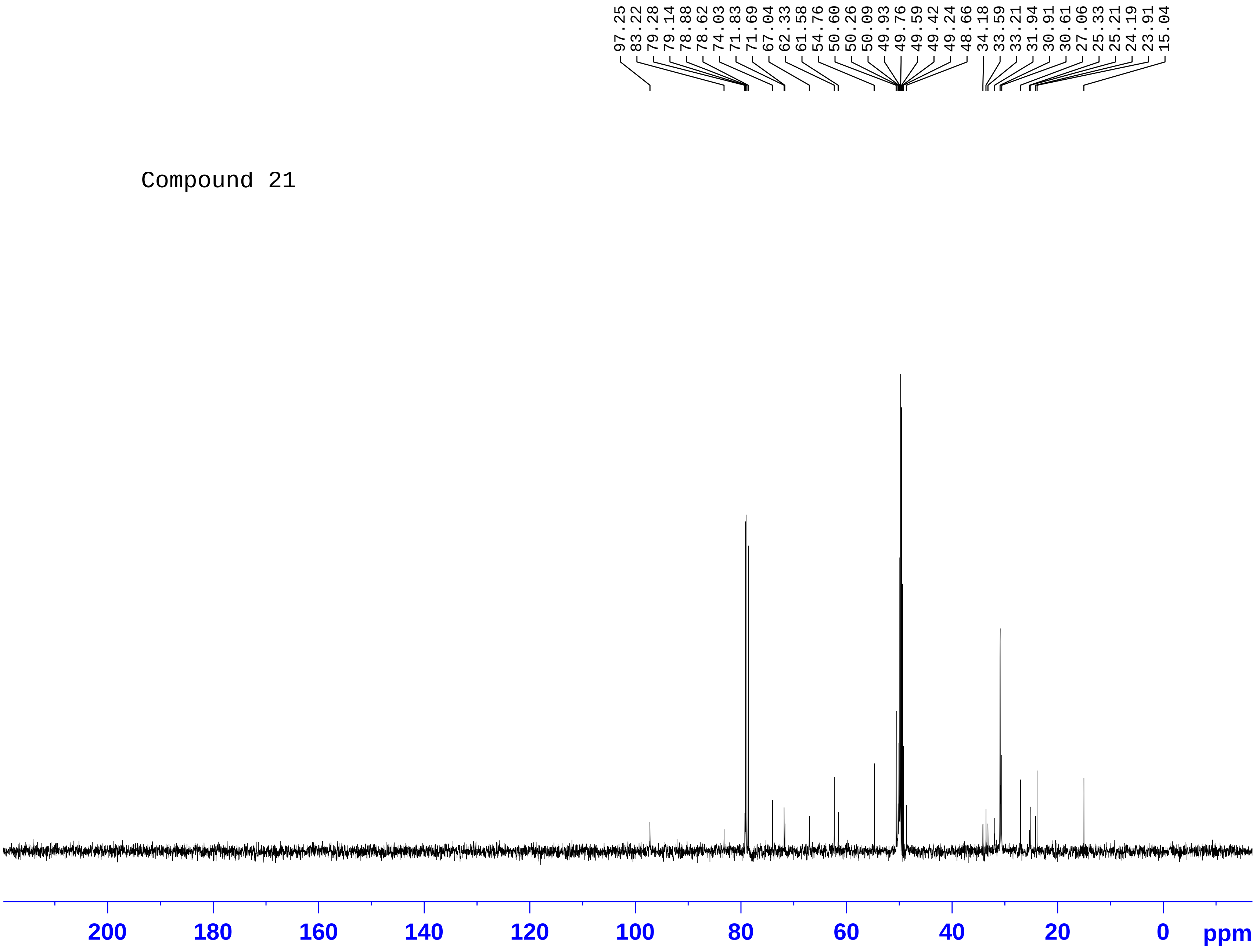

NAME	AC-AC425FR2	
EXPNO	5	
PROCNO	1	
Date_	20110516	
Time	10.18	
INSTRUM	spect	
PROBHD	5 mm QNP 1H/13	
PULPROG	zgpg30	
TD	16384	
SOLVENT	MeOD	
NS	800	
DS	4	
SWH	29761.904	Hz
FIDRES	1.816522	Hz
AQ	0.2753012	sec
RG	2050	
DW	16.800	usec
DE	6.00	usec
TE	300.2	K
D1	0.30000001	sec
d11	0.03000000	sec
DELTA	0.20000002	sec
TD0	1	
======== CHANNEL f1 ========		
NUC1	13C	
P1	7.80	usec
PL1	0.00	dB
SFO1	125.7703643	MHz
======== CHANNEL f2 ========		
CPDPRG2	waltz16	
NUC2	1H	
PCPD2	80.00	usec
PL2	-1.00	dB
PL12	16.00	dB
PL13	16.00	dB
SFO2	500.1320005	MHz
SI	8192	
SF	125.7576104	MHz
WDW	EM	
SSB	0	
LB	1.00	Hz
GB	0	
PC	1.40	

AC425 FR2-6
PROTON.d MeOD {C:\Bruker\TOPSPIN} AC 26

Compound 21

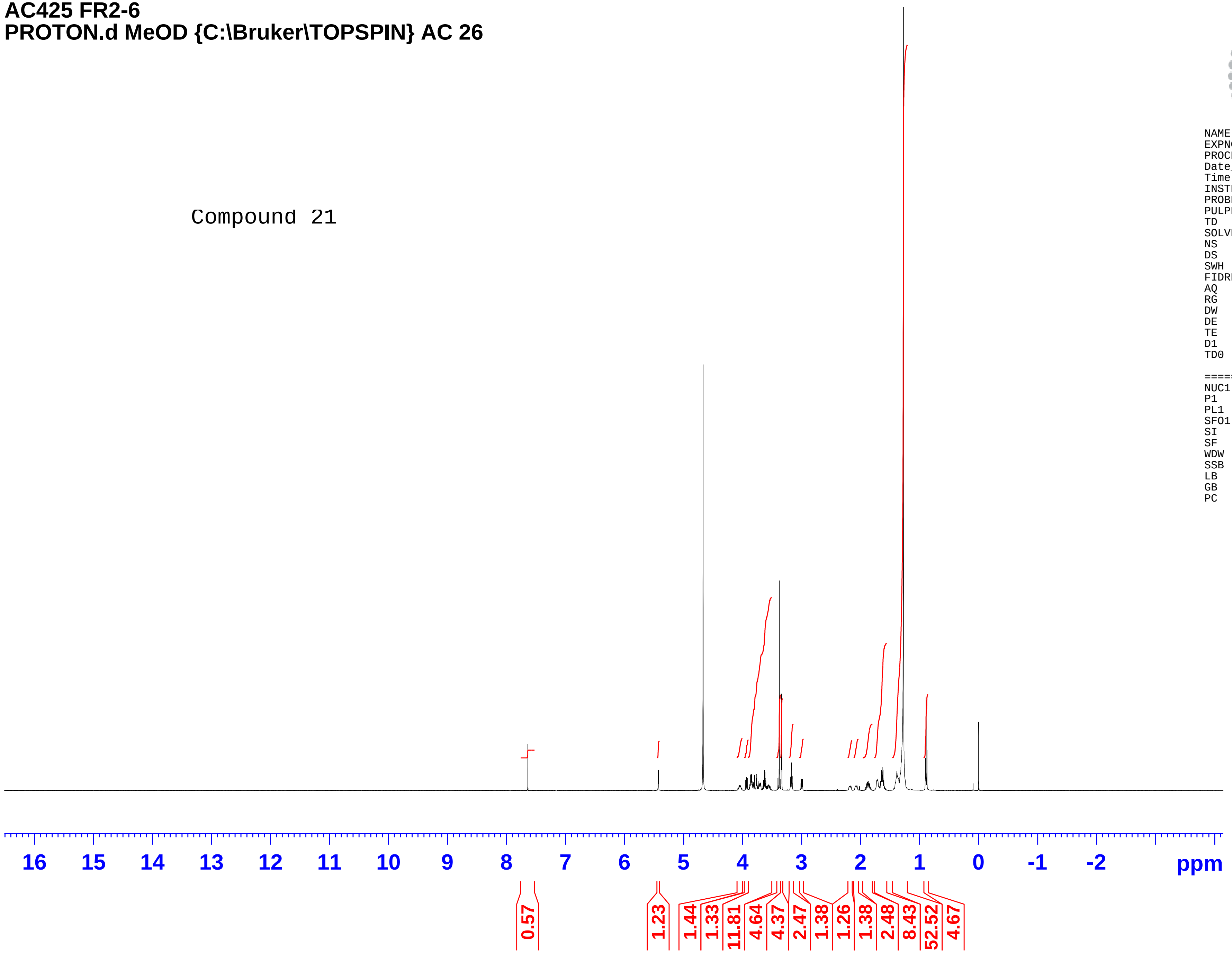

UNIVERSITY OF DUNDEE

NAME	AC-AC425FR2
EXPNO	1
PROCNO	1
Date_	20110513
Time	16.02
INSTRUM	spect
PROBHD	5 mm QNP 1H/13
PULPROG	zg30
TD	65536
SOLVENT	MeOD
NS	16
DS	2
SWH	10330.578 Hz
FIDRES	0.157632 Hz
AQ	3.1719923 sec
RG	287
DW	48.400 usec
DE	6.00 usec
TE	300.2 K
D1	1.00000000 sec
TD0	1

======== CHANNEL f1 ========

NUC1	1H
P1	11.20 usec
PL1	-1.00 dB
SFO1	500.1330885 MHz
SI	65536
SF	500.1299962 MHz
WDW	EM
SSB	0
LB	0.30 Hz
GB	0
PC	1.40

AC427

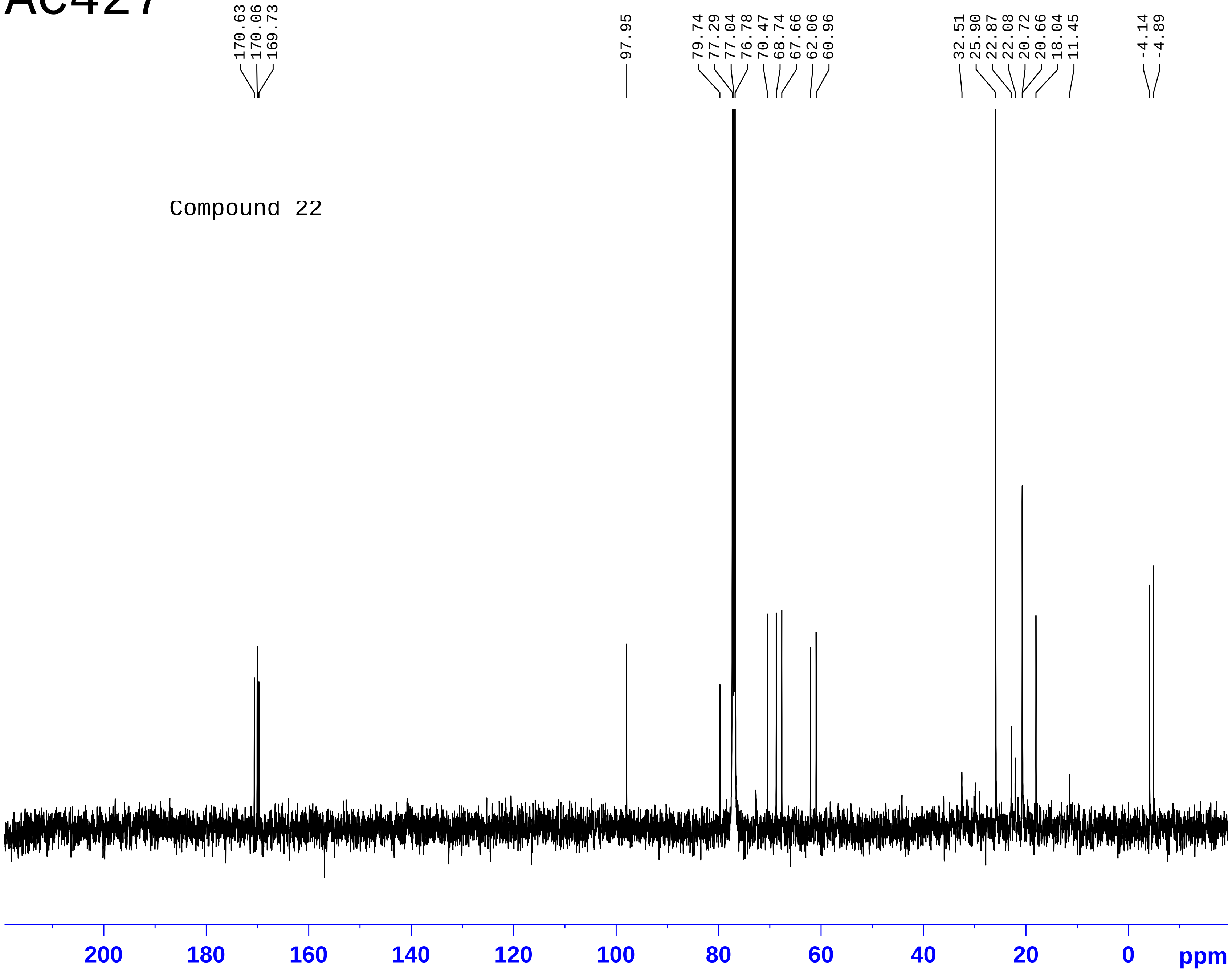

Compound 22

UNIVERSITY OF DUNDEE

NAME	AC-AC427	
EXPNO	2	
PROCNO	1	
Date_	20110530	
Time	14.56	
INSTRUM	AV500	
PROBHD	5 mm CPTXI 1H-	
PULPROG	zgpg30	
TD	65536	
SOLVENT	CDCl3	
NS	256	
DS	2	
SWH	30030.029	Hz
FIDRES	0.458222	Hz
AQ	1.0912410	sec
RG	23170.5	
DW	16.650	usec
DE	30.00	usec
TE	298.0	K
D1	2.00000000	sec
D11	0.03000000	sec
TD0	1	
======== CHANNEL f1 ========		
NUC1	13C	
P1	14.00	usec
PL1	-4.10	dB
PL1W	105.88729095	W
SFO1	125.7703643	MHz
======== CHANNEL f2 ========		
CPDPRG2	waltz16	
NUC2	1H	
PCPD2	80.00	usec
PL2	1.00	dB
PL12	21.33	dB
PL13	120.00	dB
PL2W	7.20289707	W
PL12W	0.06675860	W
PL13W	0.00000000	W
SFO2	500.1320005	MHz
SI	32768	
SF	125.7577890	MHz
WDW	EM	
SSB	0	
LB	3.00	Hz
GB	0	
PC	1.40	

AC427

Compound 22

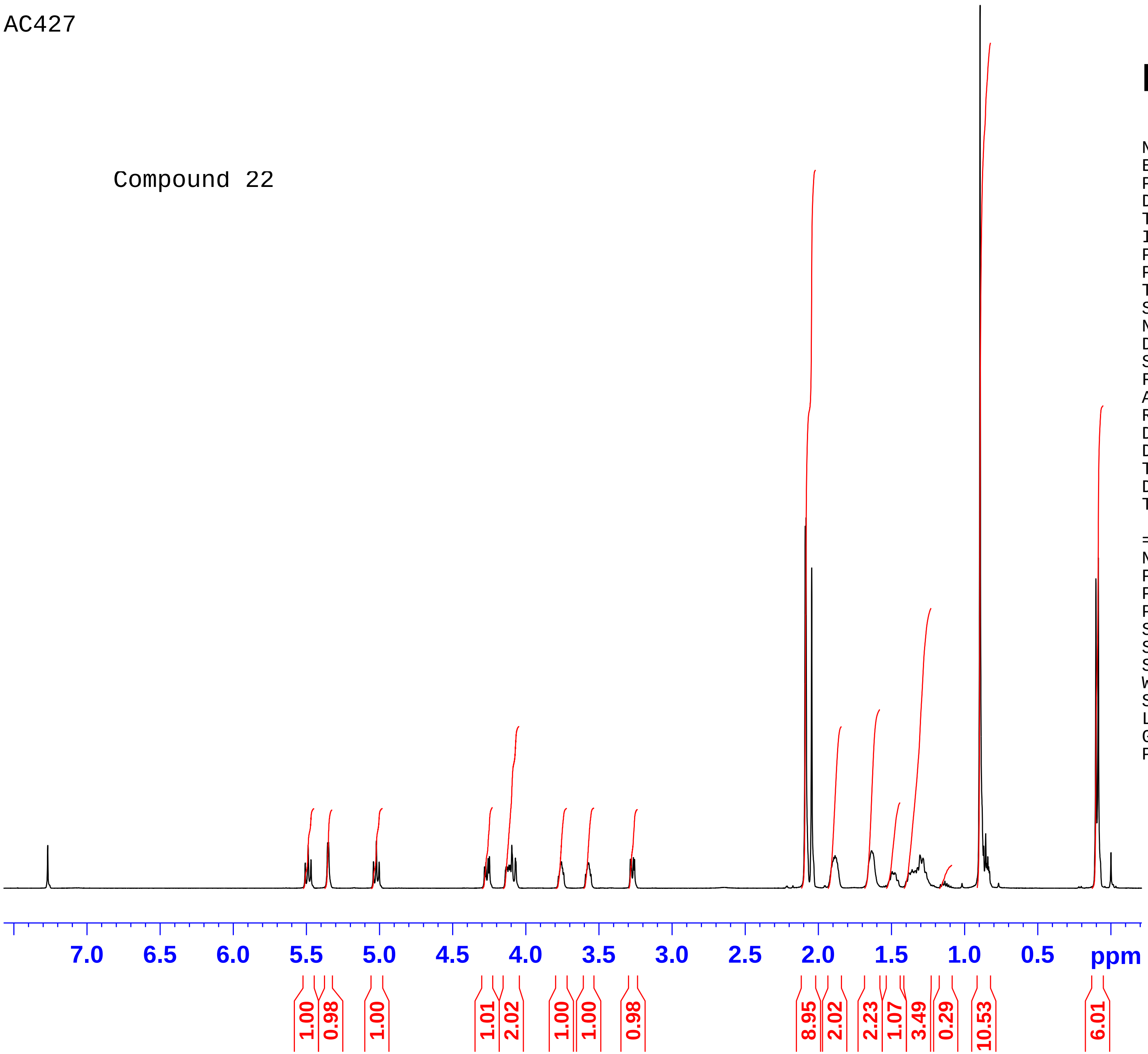

NAME	AC-AC427
EXPNO	1
PROCNO	1
Date_	20110530
Time	14.42
INSTRUM	AV500
PROBHD	5 mm CPTXI 1H-
PULPROG	zg30
TD	65536
SOLVENT	CDCl3
NS	16
DS	2
SWH	10330.578 Hz
FIDRES	0.157632 Hz
AQ	3.1720407 sec
RG	4
DW	48.400 usec
DE	6.50 usec
TE	298.0 K
D1	1.00000000 sec
TD0	1

======== CHANNEL f1 ========

NUC1	1H
P1	7.70 usec
PL1	1.00 dB
PL1W	7.20289707 W
SFO1	500.1330885 MHz
SI	32768
SF	500.1300093 MHz
WDW	EM
SSB	0
LB	0.30 Hz
GB	0
PC	1.00

AC429
C13CPDfast.d CDCl3 {C:\Bruker\TOPSPIN} AC 15

Compound 23

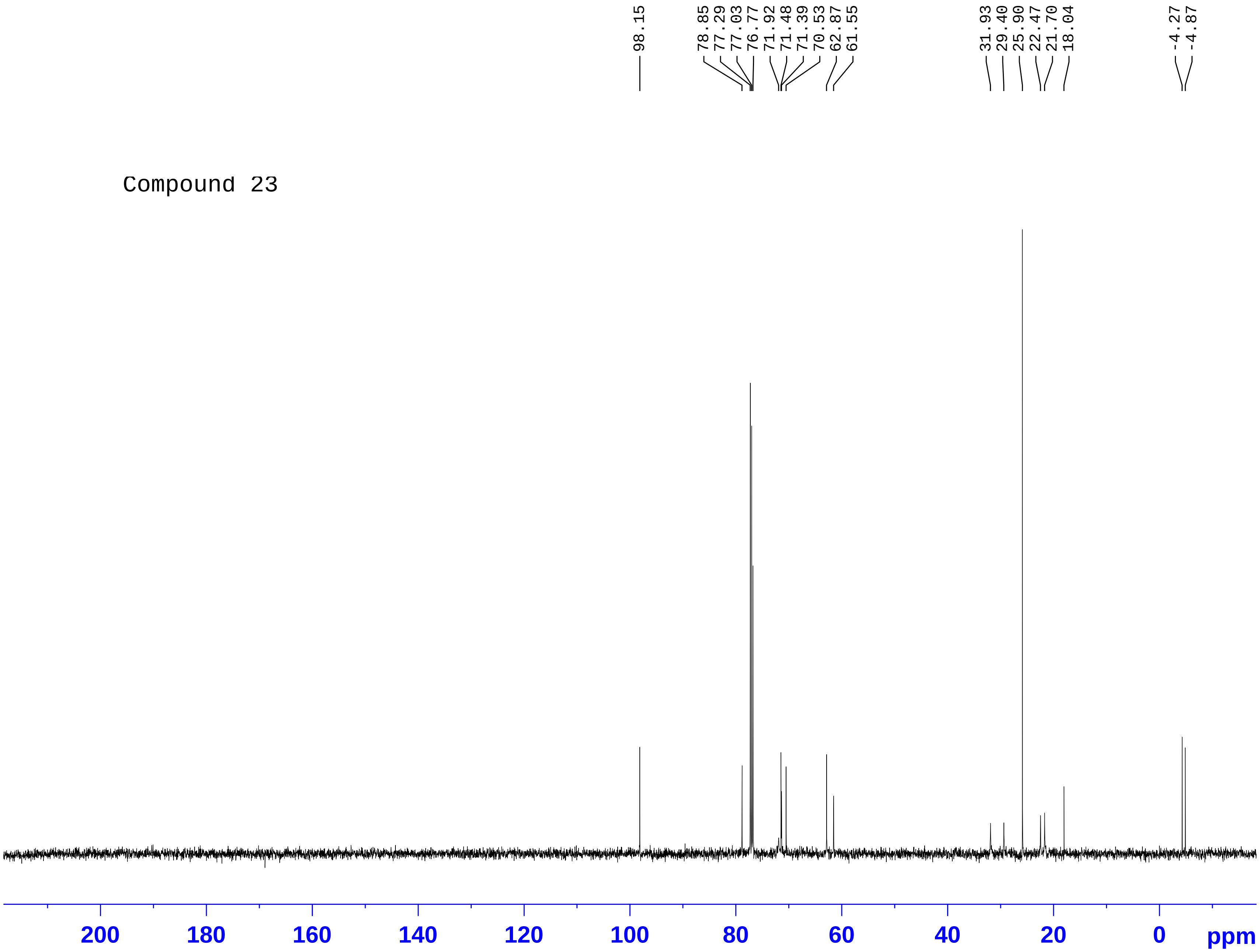

UNIVERSITY OF DUNDEE

NAME	AC-AC429	
EXPNO	5	
PROCNO	1	
Date_	20110621	
Time	10.50	
INSTRUM	spect	
PROBHD	5 mm QNP 1H/13	
PULPROG	zgpg30	
TD	16384	
SOLVENT	CDCl3	
NS	800	
DS	4	
SWH	29761.904	Hz
FIDRES	1.816522	Hz
AQ	0.2753012	sec
RG	2050	
DW	16.800	usec
DE	6.00	usec
TE	294.9	K
D1	0.30000001	sec
d11	0.03000000	sec
DELTA	0.20000002	sec
TD0	1	

======== CHANNEL f1 ========

NUC1	13C	
P1	8.18	usec
PL1	0.00	dB
SFO1	125.7703643	MHz

======== CHANNEL f2 ========

CPDPRG2	waltz16	
NUC2	1H	
PCPD2	80.00	usec
PL2	-1.00	dB
PL12	16.00	dB
PL13	16.00	dB
SFO2	500.1320005	MHz
SI	8192	
SF	125.7577890	MHz
WDW	EM	
SSB	0	
LB	1.00	Hz
GB	0	
PC	1.40	

AC429
PROTON.d CDCl3 {C:\Bruker\TOPSPIN} AC 15

Compound 23

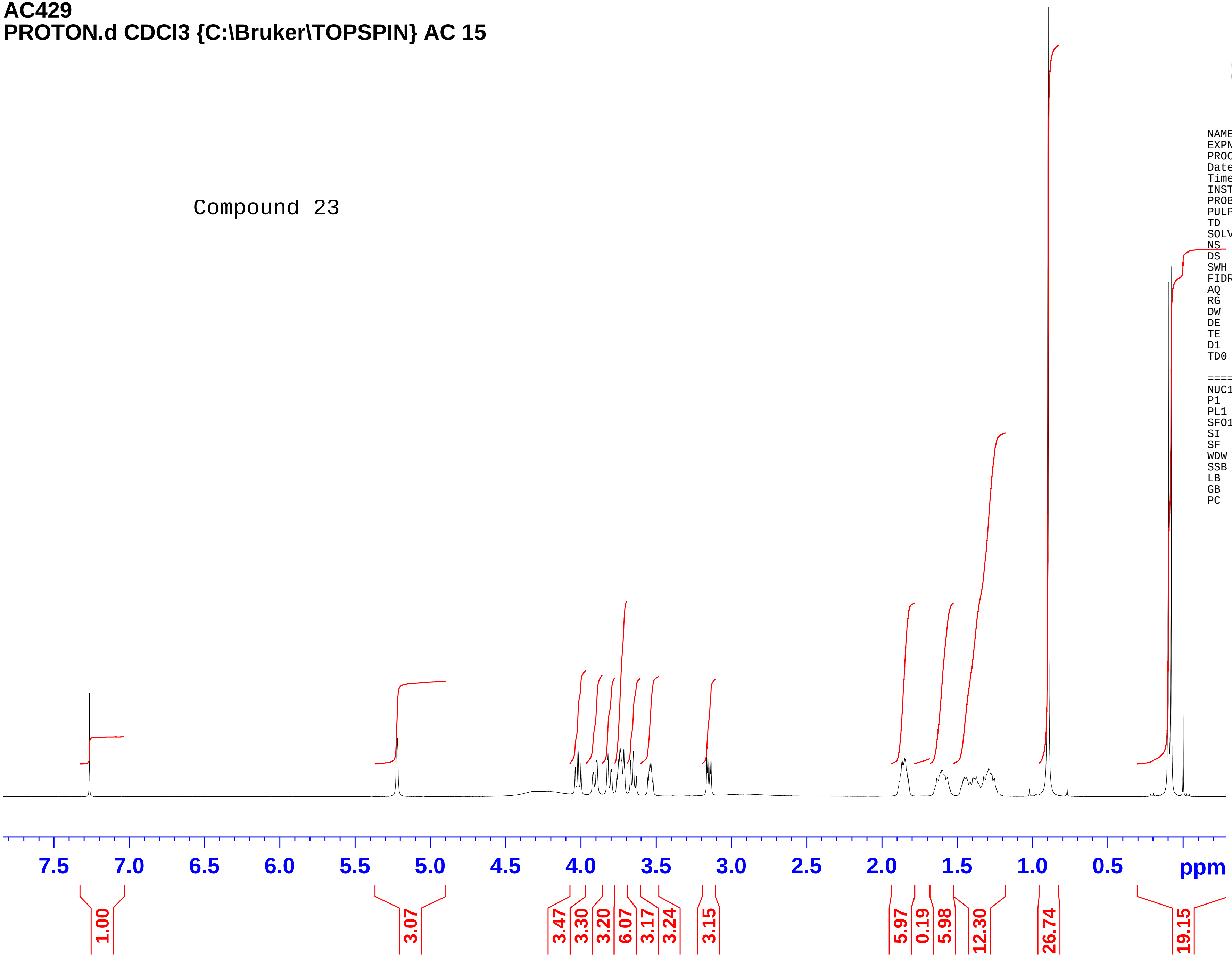

UNIVERSITY OF DUNDEE

NAME	AC-AC429
EXPNO	3
PROCNO	1
Date_	20110621
Time	10.30
INSTRUM	spect
PROBHD	5 mm QNP 1H/13
PULPROG	zg30
TD	65536
SOLVENT	CDCl3
NS	16
DS	2
SWH	10330.578 Hz
FIDRES	0.157632 Hz
AQ	3.1719923 sec
RG	228
DW	48.400 usec
DE	6.00 usec
TE	294.1 K
D1	1.00000000 sec
TD0	1

======== CHANNEL f1 ========

NUC1	1H
P1	11.20 usec
PL1	-1.00 dB
SFO1	500.1330885 MHz
SI	65536
SF	500.1300115 MHz
WDW	EM
SSB	0
LB	0.30 Hz
GB	0
PC	1.40

AC431
C13CPDfast.d CDCl3 {C:\Bruker\TOPSPIN} AC 2

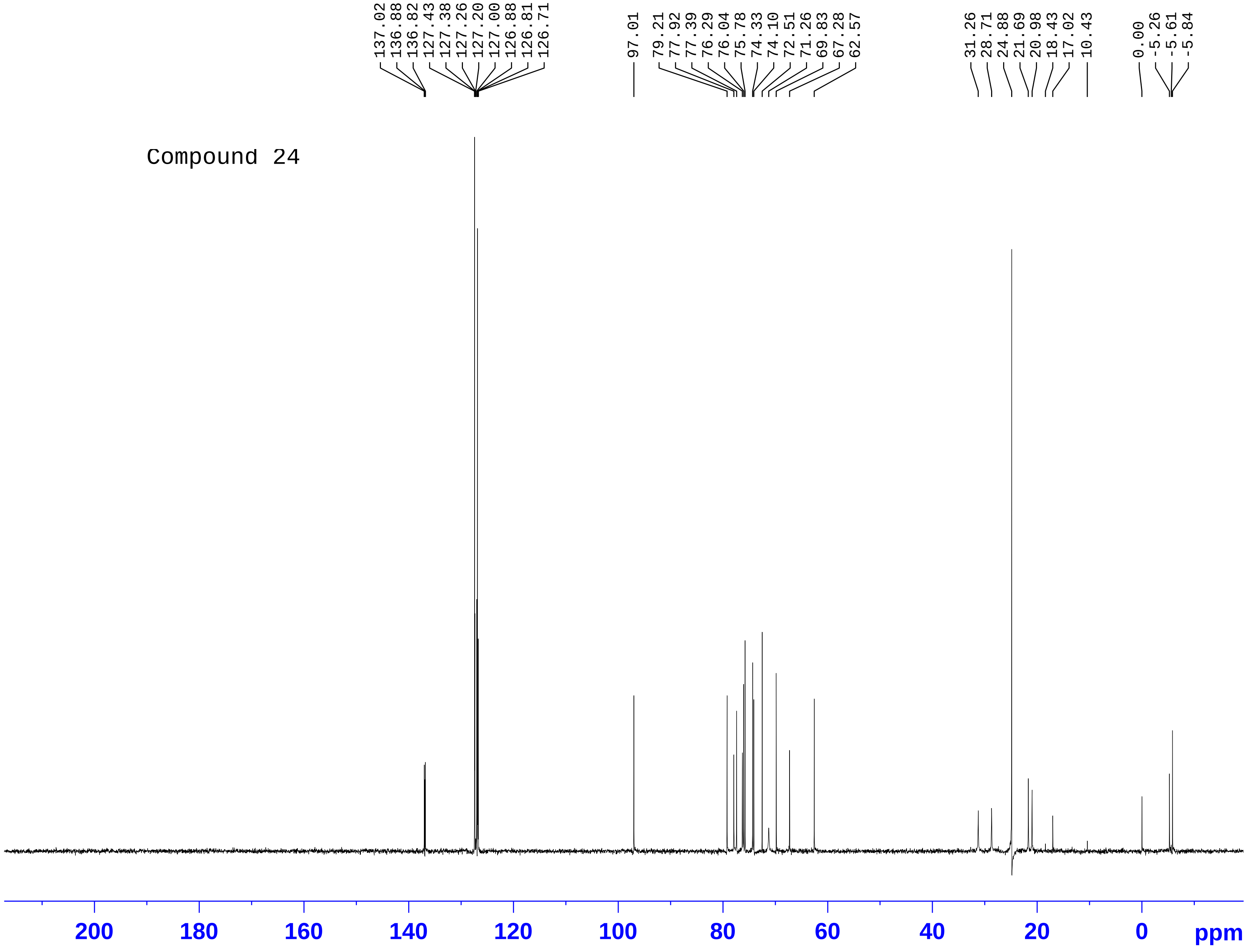

UNIVERSITY OF DUNDEE

NAME	AC-AC431
EXPNO	4
PROCNO	1
Date_	20110706
Time	10.21
INSTRUM	spect
PROBHD	5 mm QNP 1H/13
PULPROG	zgpg30
TD	16384
SOLVENT	CDCl3
NS	800
DS	4
SWH	29761.904 Hz
FIDRES	1.816522 Hz
AQ	0.2753012 sec
RG	2050
DW	16.800 usec
DE	6.00 usec
TE	295.2 K
D1	0.30000001 sec
d11	0.03000000 sec
DELTA	0.20000002 sec
TD0	1

======== CHANNEL f1 ========

NUC1	13C
P1	8.18 usec
PL1	0.00 dB
SFO1	125.7703643 MHz

======== CHANNEL f2 ========

CPDPRG2	waltz16
NUC2	1H
PCPD2	80.00 usec
PL2	-1.00 dB
PL12	16.00 dB
PL13	16.00 dB
SFO2	500.1320005 MHz
SI	8192
SF	125.7579247 MHz
WDW	EM
SSB	0
LB	1.00 Hz
GB	0
PC	1.40

AC431
PROTON.d CDCl3 {C:\Bruker\TOPSPIN} AC 2

Compound 24

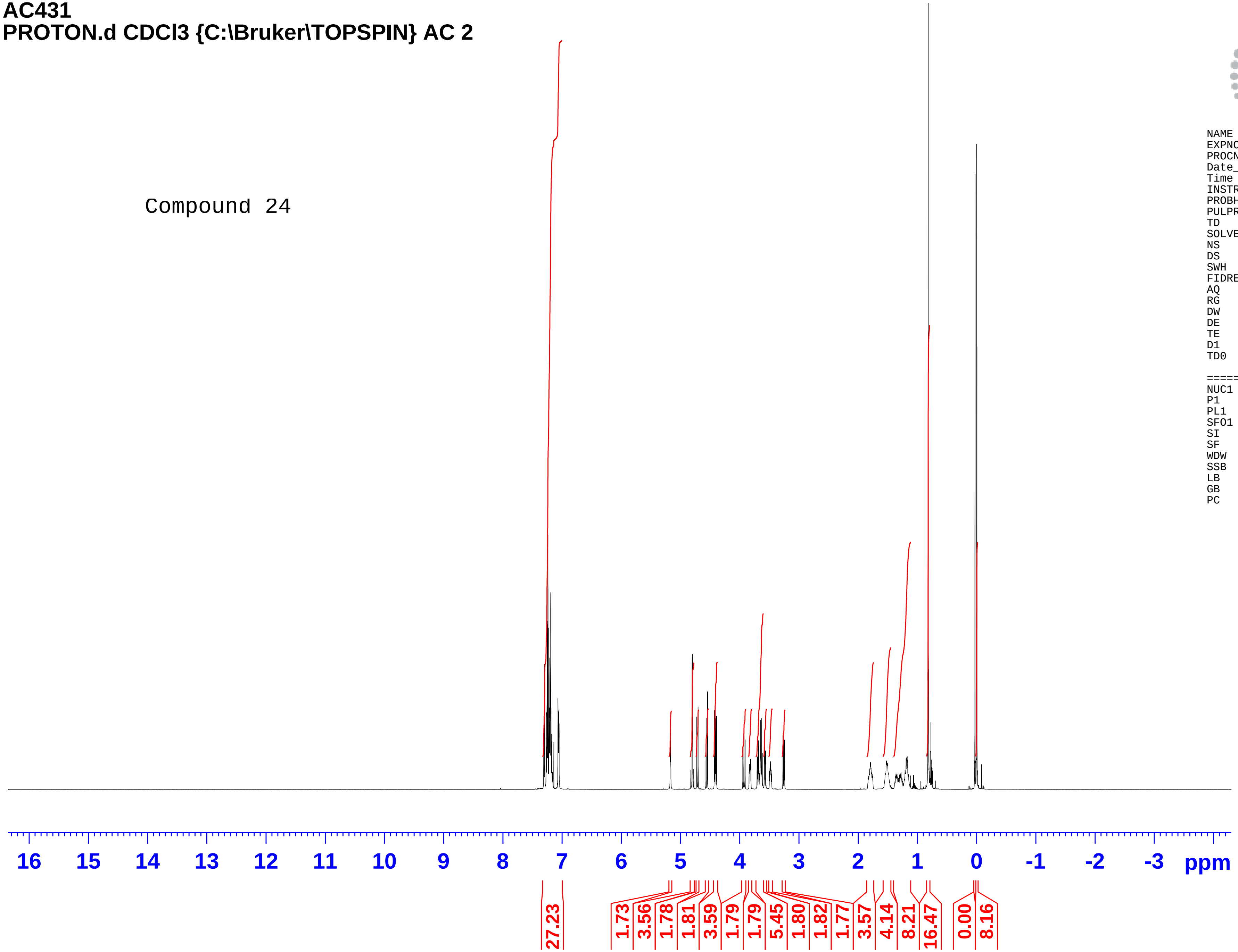

UNIVERSITY OF DUNDEE

```
NAME          AC-AC431
EXPNO                2
PROCNO               1
Date_         20110706
Time             10.01
INSTRUM          spect
PROBHD   5 mm QNP 1H/13
PULPROG           zg30
TD               65536
SOLVENT          CDCl3
NS                  16
DS                   2
SWH         10330.578 Hz
FIDRES       0.157632 Hz
AQ          3.1719923 sec
RG                50.8
DW              48.400 usec
DE                6.00 usec
TE               294.3 K
D1          1.00000000 sec
TD0                  1

======== CHANNEL f1 ========
NUC1                1H
P1               11.20 usec
PL1              -1.00 dB
SFO1       500.1330885 MHz
SI               65536
SF         500.1300735 MHz
WDW                 EM
SSB                  0
LB                0.30 Hz
GB                   0
PC                1.40
```

AC434
C13CPDfast.d CDCl3 {C:\Bruker\TOPSPIN} AC 1

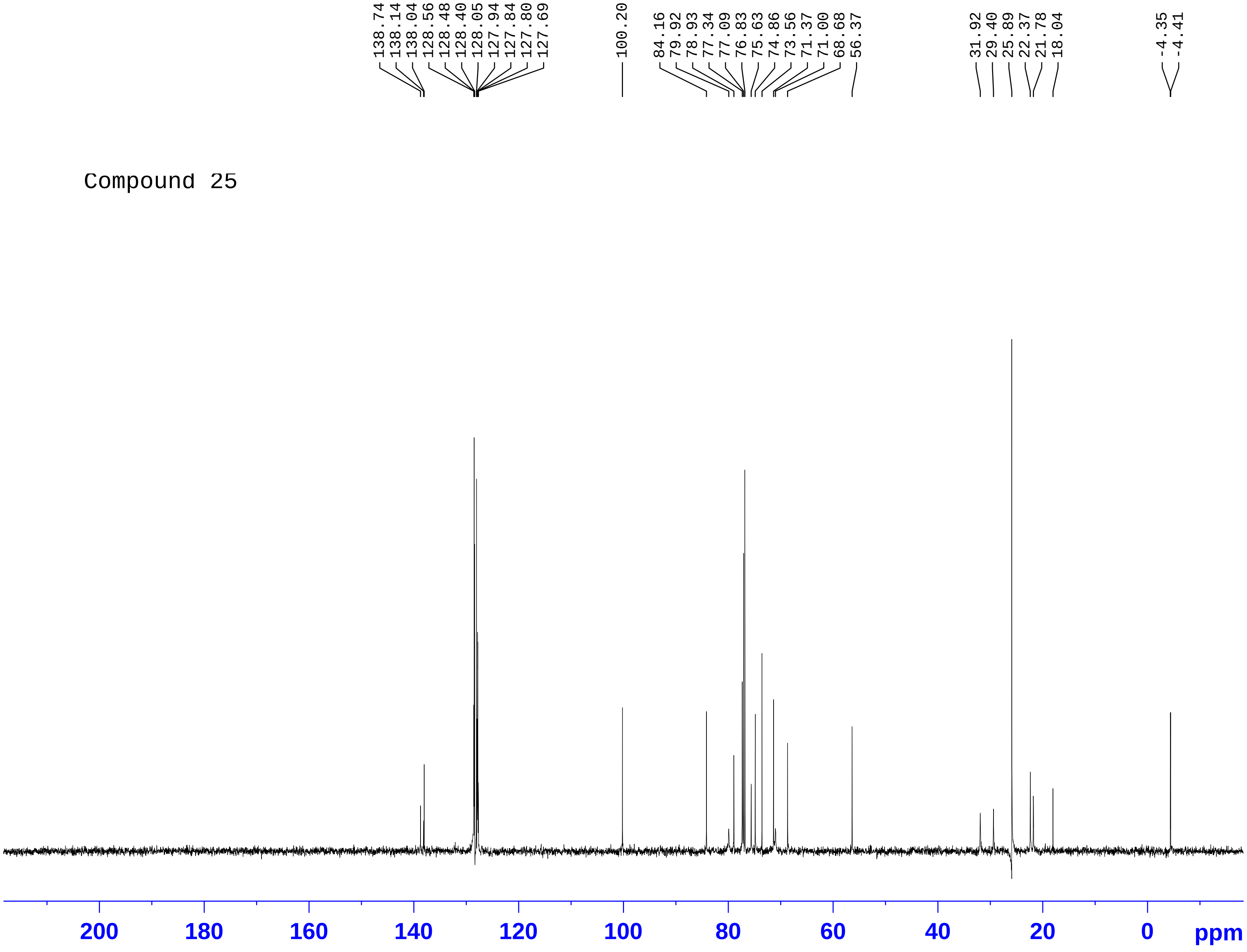

Compound 25

UNIVERSITY OF DUNDEE

NAME	AC-AC424	
EXPNO	11	
PROCNO	1	
Date_	20110729	
Time	9.51	
INSTRUM	spect	
PROBHD	5 mm QNP 1H/13	
PULPROG	zgpg30	
TD	16384	
SOLVENT	CDCl3	
NS	800	
DS	4	
SWH	29761.904	Hz
FIDRES	1.816522	Hz
AQ	0.2753012	sec
RG	2050	
DW	16.800	usec
DE	6.00	usec
TE	294.9	K
D1	0.30000001	sec
d11	0.03000000	sec
DELTA	0.20000002	sec
TD0	1	
======== CHANNEL f1 ========		
NUC1	13C	
P1	8.18	usec
PL1	0.00	dB
SFO1	125.7703643	MHz
======== CHANNEL f2 ========		
CPDPRG2	waltz16	
NUC2	1H	
PCPD2	80.00	usec
PL2	-1.00	dB
PL12	16.00	dB
PL13	16.00	dB
SFO2	500.1320005	MHz
SI	8192	
SF	125.7577890	MHz
WDW	EM	
SSB	0	
LB	1.00	Hz
GB	0	
PC	1.40	

AC434
PROTON.d CDCl3 {C:\Bruker\TOPSPIN} AC 1

Compound 25

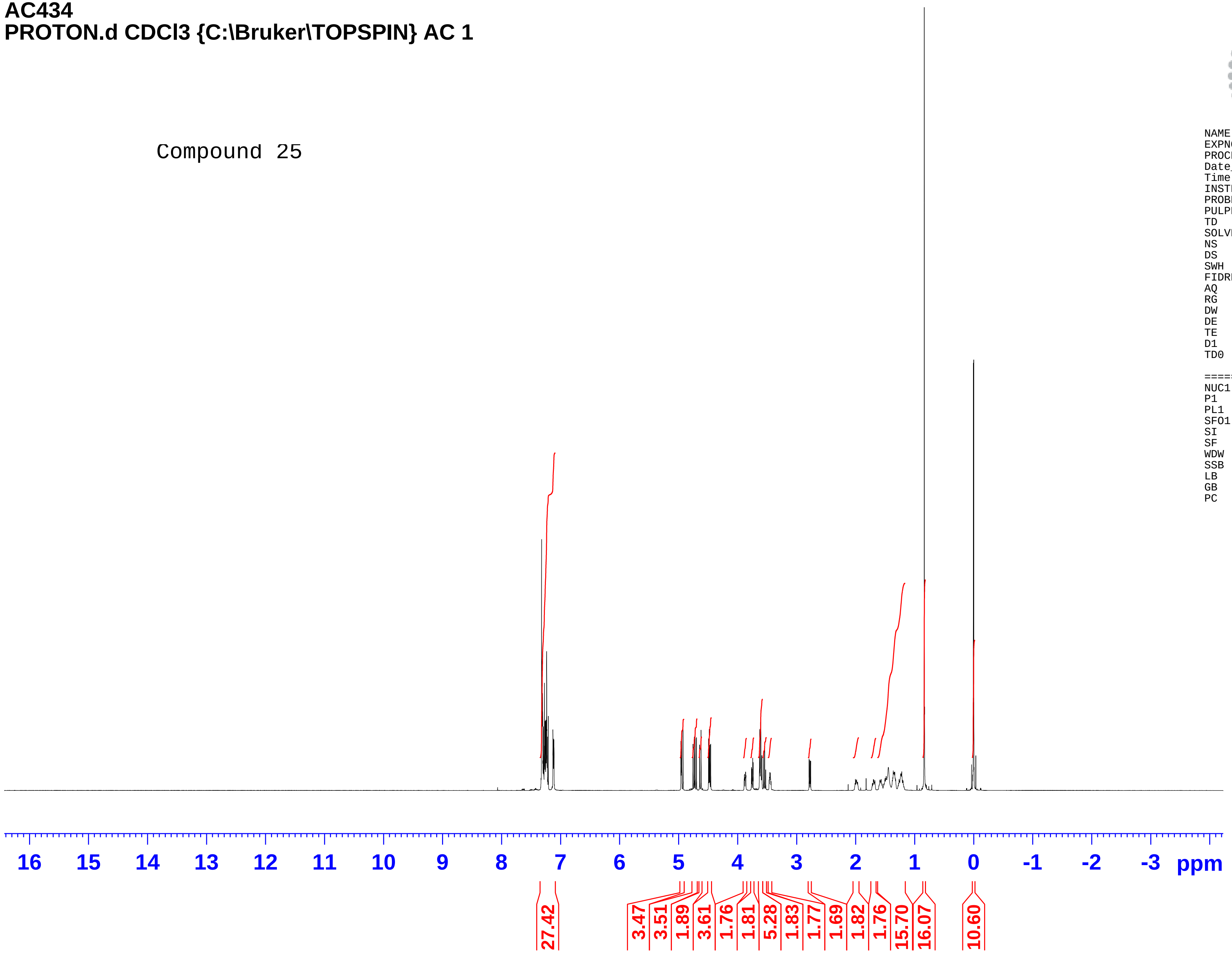

UNIVERSITY OF DUNDEE

NAME	AC-AC424
EXPNO	9
PROCNO	1
Date_	20110729
Time	9.31
INSTRUM	spect
PROBHD	5 mm QNP 1H/13
PULPROG	zg30
TD	65536
SOLVENT	CDCl3
NS	16
DS	2
SWH	10330.578 Hz
FIDRES	0.157632 Hz
AQ	3.1719923 sec
RG	71.8
DW	48.400 usec
DE	6.00 usec
TE	294.0 K
D1	1.00000000 sec
TD0	1

======== CHANNEL f1 ========

NUC1	1H
P1	11.20 usec
PL1	-1.00 dB
SFO1	500.1330885 MHz
SI	65536
SF	500.1300380 MHz
WDW	EM
SSB	0
LB	0.30 Hz
GB	0
PC	1.40

AC435
C13CPDfast.d CDCl3 {C:\Bruker\TOPSPIN} AC 31

Compound 26

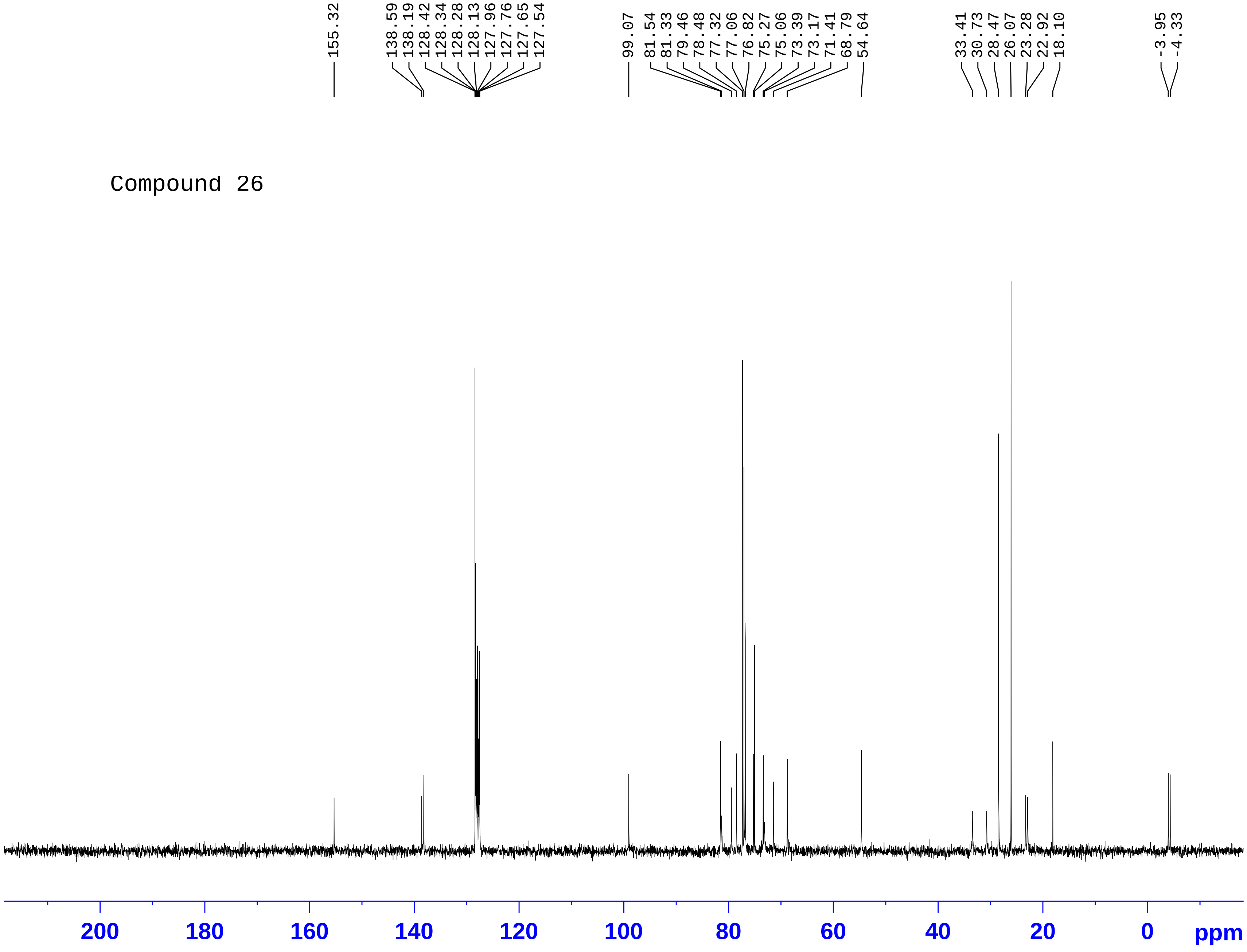

NAME	AC-AC435	
EXPNO	4	
PROCNO	1	
Date_	20110808	
Time	14.12	
INSTRUM	spect	
PROBHD	5 mm QNP 1H/13	
PULPROG	zgpg30	
TD	16384	
SOLVENT	CDCl3	
NS	800	
DS	4	
SWH	29761.904	Hz
FIDRES	1.816522	Hz
AQ	0.2753012	sec
RG	2050	
DW	16.800	usec
DE	6.00	usec
TE	295.3	K
D1	0.30000001	sec
d11	0.03000000	sec
DELTA	0.20000002	sec
TD0	1	
======== CHANNEL f1 ========		
NUC1	13C	
P1	8.18	usec
PL1	0.00	dB
SFO1	125.7703643	MHz
======== CHANNEL f2 ========		
CPDPRG2	waltz16	
NUC2	1H	
PCPD2	80.00	usec
PL2	-1.00	dB
PL12	16.00	dB
PL13	16.00	dB
SFO2	500.1320005	MHz
SI	8192	
SF	125.7577890	MHz
WDW	EM	
SSB	0	
LB	1.00	Hz
GB	0	
PC	1.40	

AC435
PROTON.d CDCl3 {C:\Bruker\TOPSPIN} AC 31

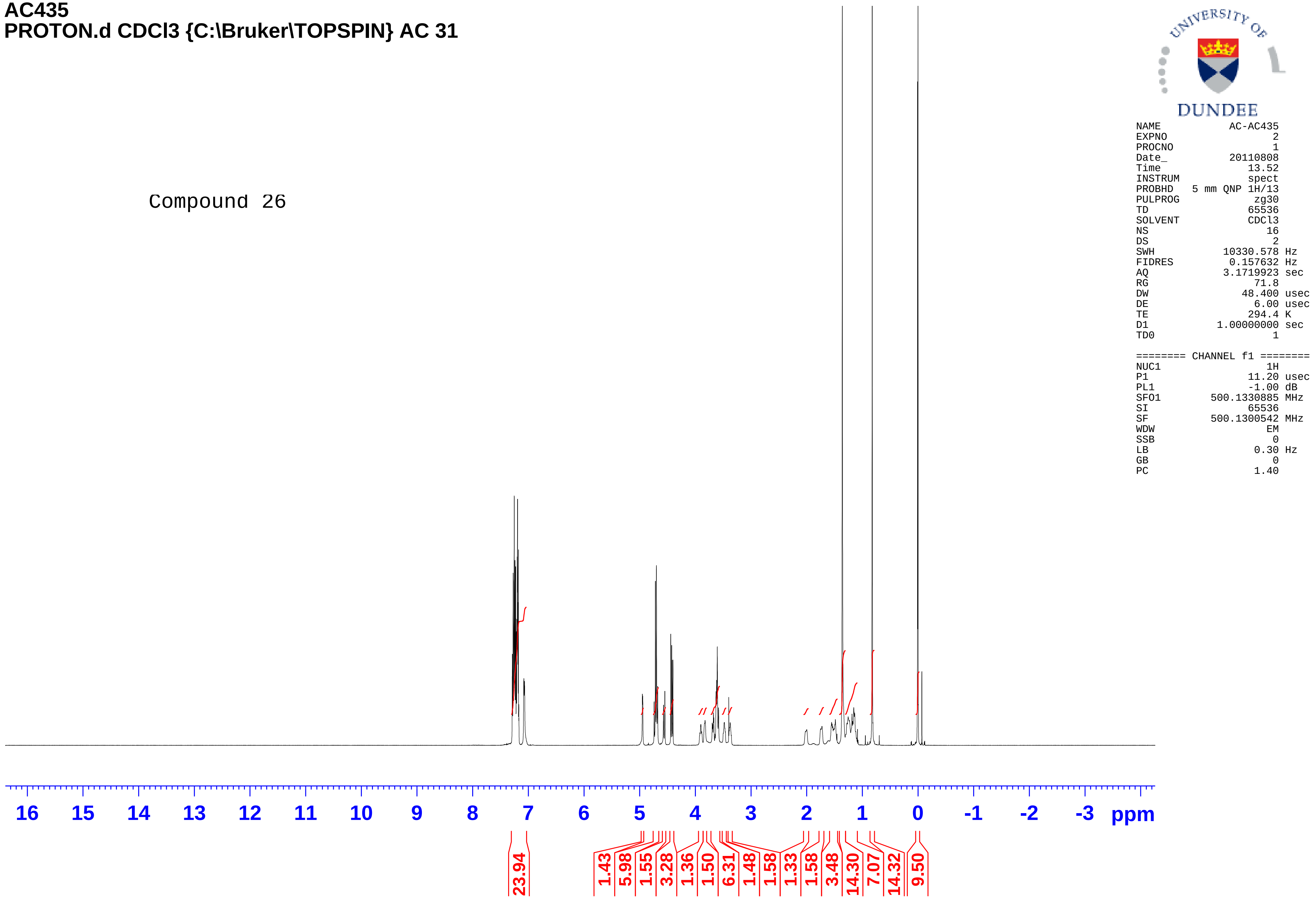

AC442 SCALE UP
C13CPDfast.d CDCl3 {C:\Bruker\TOPSPIN} AC 2

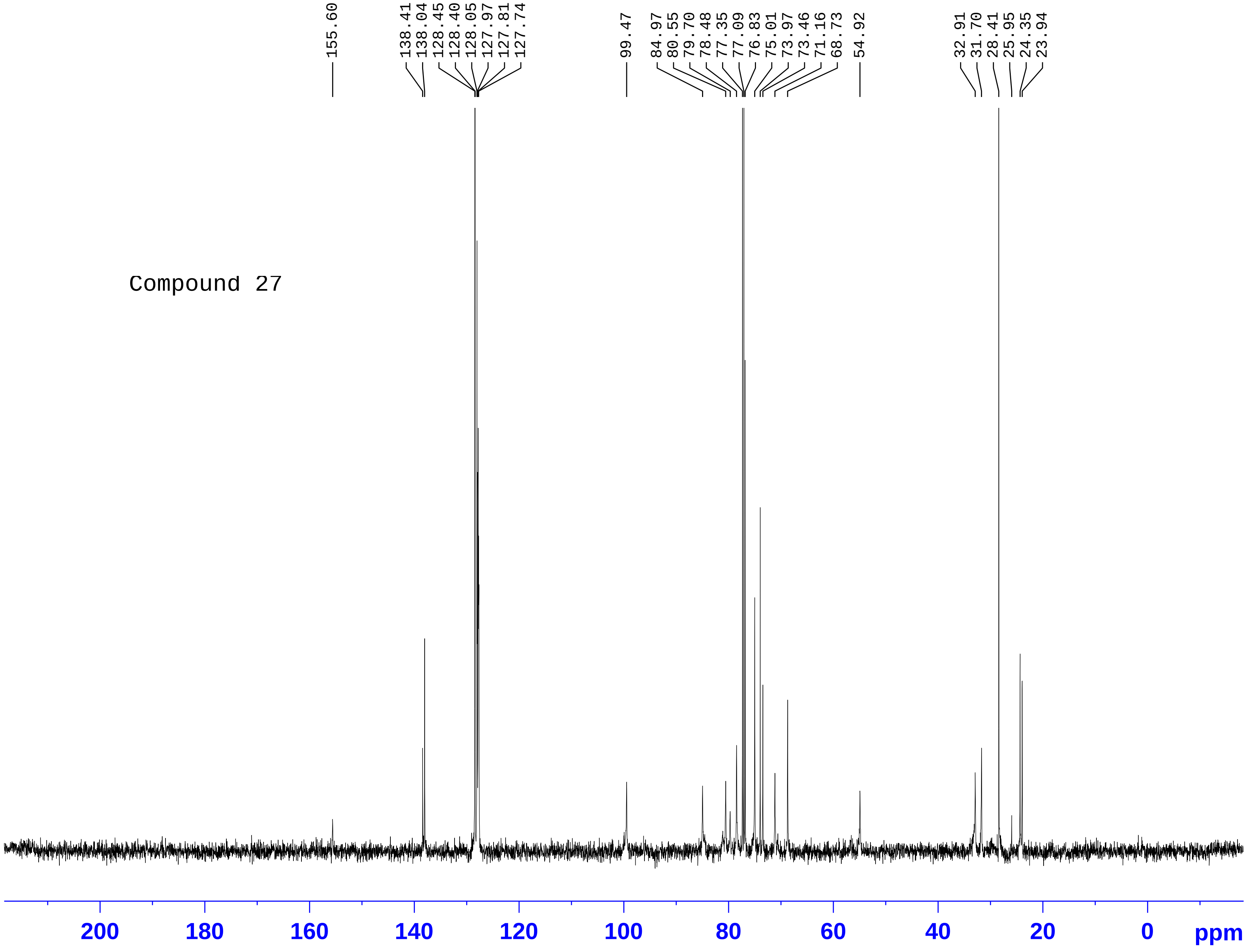

Compound 27

UNIVERSITY OF DUNDEE

NAME	AC-AC442
EXPNO	8
PROCNO	1
Date_	20111020
Time	10.21
INSTRUM	spect
PROBHD	5 mm QNP 1H/13
PULPROG	zgpg30
TD	16384
SOLVENT	CDCl3
NS	800
DS	4
SWH	29761.904 Hz
FIDRES	1.816522 Hz
AQ	0.2753012 sec
RG	2050
DW	16.800 usec
DE	6.00 usec
TE	295.1 K
D1	0.30000001 sec
d11	0.03000000 sec
DELTA	0.20000002 sec
TD0	1

======== CHANNEL f1 ========

NUC1	13C
P1	8.18 usec
PL1	0.00 dB
SFO1	125.7703643 MHz

======== CHANNEL f2 ========

CPDPRG2	waltz16
NUC2	1H
PCPD2	80.00 usec
PL2	-1.00 dB
PL12	16.00 dB
PL13	16.00 dB
SFO2	500.1320005 MHz
SI	8192
SF	125.7577890 MHz
WDW	EM
SSB	0
LB	1.00 Hz
GB	0
PC	1.40

AC442
PROTON.d CDCl3 {C:\Bruker\TOPSPIN} AC 1

Compound 27

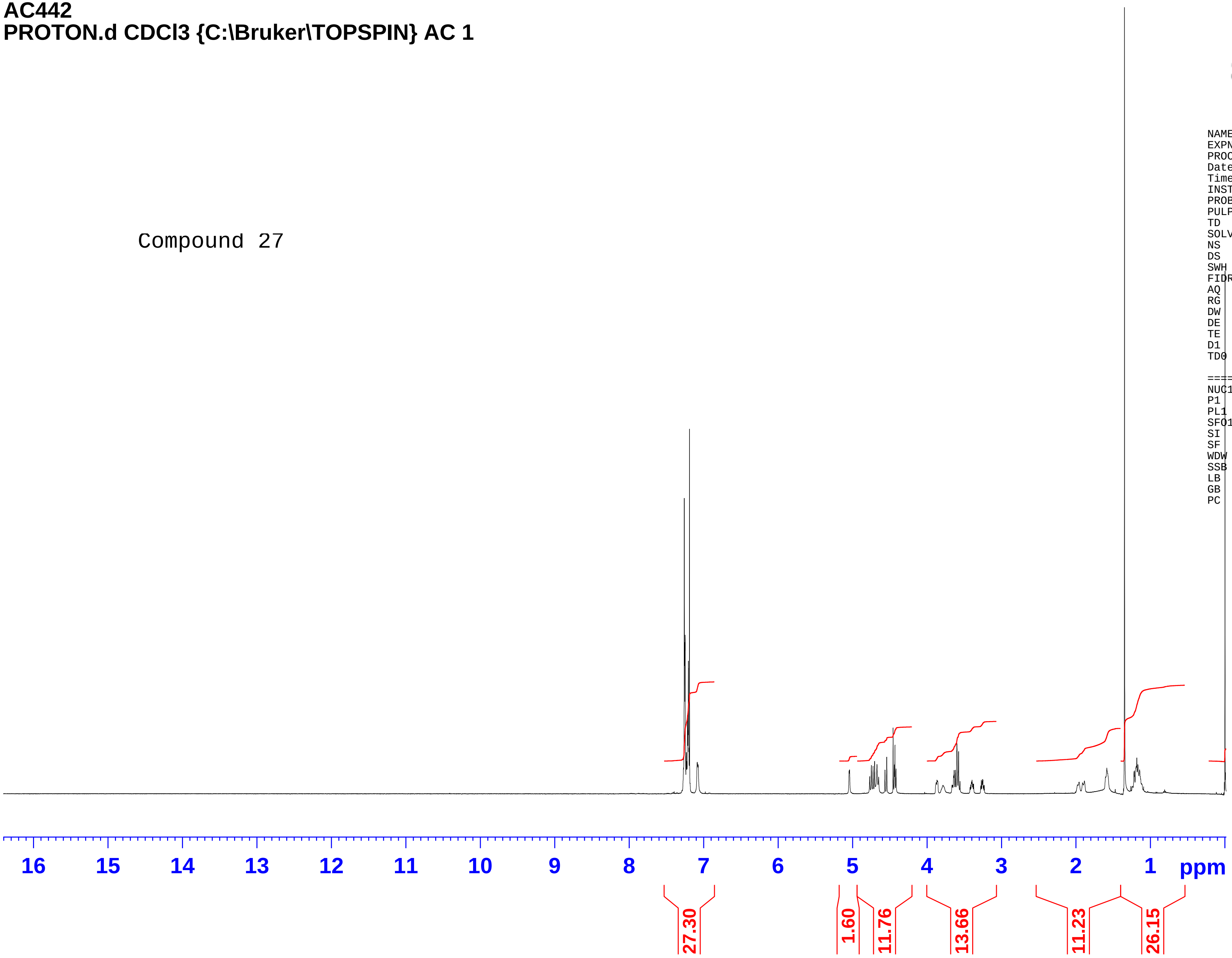

UNIVERSITY OF DUNDEE

NAME	AC-AC442
EXPNO	2
PROCNO	1
Date_	20111011
Time	9.48
INSTRUM	spect
PROBHD	5 mm QNP 1H/13
PULPROG	zg30
TD	65536
SOLVENT	CDCl3
NS	16
DS	2
SWH	10330.578 Hz
FIDRES	0.157632 Hz
AQ	3.1719923 sec
RG	512
DW	48.400 usec
DE	6.00 usec
TE	294.1 K
D1	1.00000000 sec
TD0	1

======== CHANNEL f1 ========

NUC1	1H
P1	11.20 usec
PL1	-1.00 dB
SFO1	500.1330885 MHz
SI	65536
SF	500.1300482 MHz
WDW	EM
SSB	0
LB	0.30 Hz
GB	0
PC	1.40

AC443
C13CPDfast.d CDCl3 {C:\Bruker\TOPSPIN} AC 25

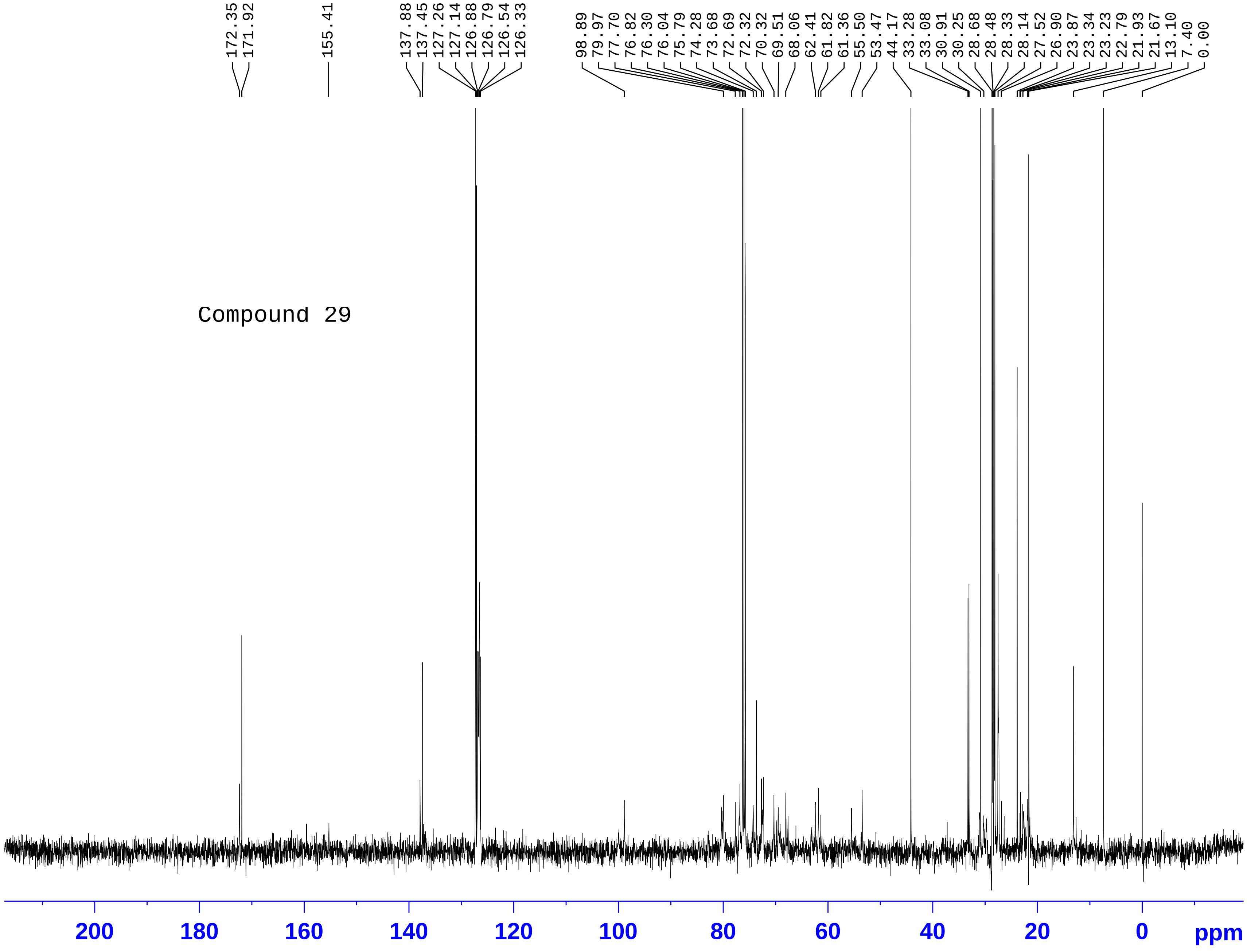

NAME	AC-AC443	
EXPNO	5	
PROCNO	1	
Date_	20111028	
Time	11.35	
INSTRUM	spect	
PROBHD	5 mm QNP 1H/13	
PULPROG	zgpg30	
TD	16384	
SOLVENT	CDCl3	
NS	800	
DS	4	
SWH	29761.904	Hz
FIDRES	1.816522	Hz
AQ	0.2753012	sec
RG	2050	
DW	16.800	usec
DE	6.00	usec
TE	298.2	K
D1	0.30000001	sec
d11	0.03000000	sec
DELTA	0.20000002	sec
TD0	1	

======== CHANNEL f1 ========

NUC1	13C	
P1	8.18	usec
PL1	0.00	dB
SFO1	125.7703643	MHz

======== CHANNEL f2 ========

CPDPRG2	waltz16	
NUC2	1H	
PCPD2	80.00	usec
PL2	-1.00	dB
PL12	16.00	dB
PL13	16.00	dB
SFO2	500.1320005	MHz
SI	8192	
SF	125.7579175	MHz
WDW	EM	
SSB	0	
LB	1.00	Hz
GB	0	
PC	1.40	

AC443
PROTON.d CDCl3 {C:\Bruker\TOPSPIN} AC 25

Compound 29

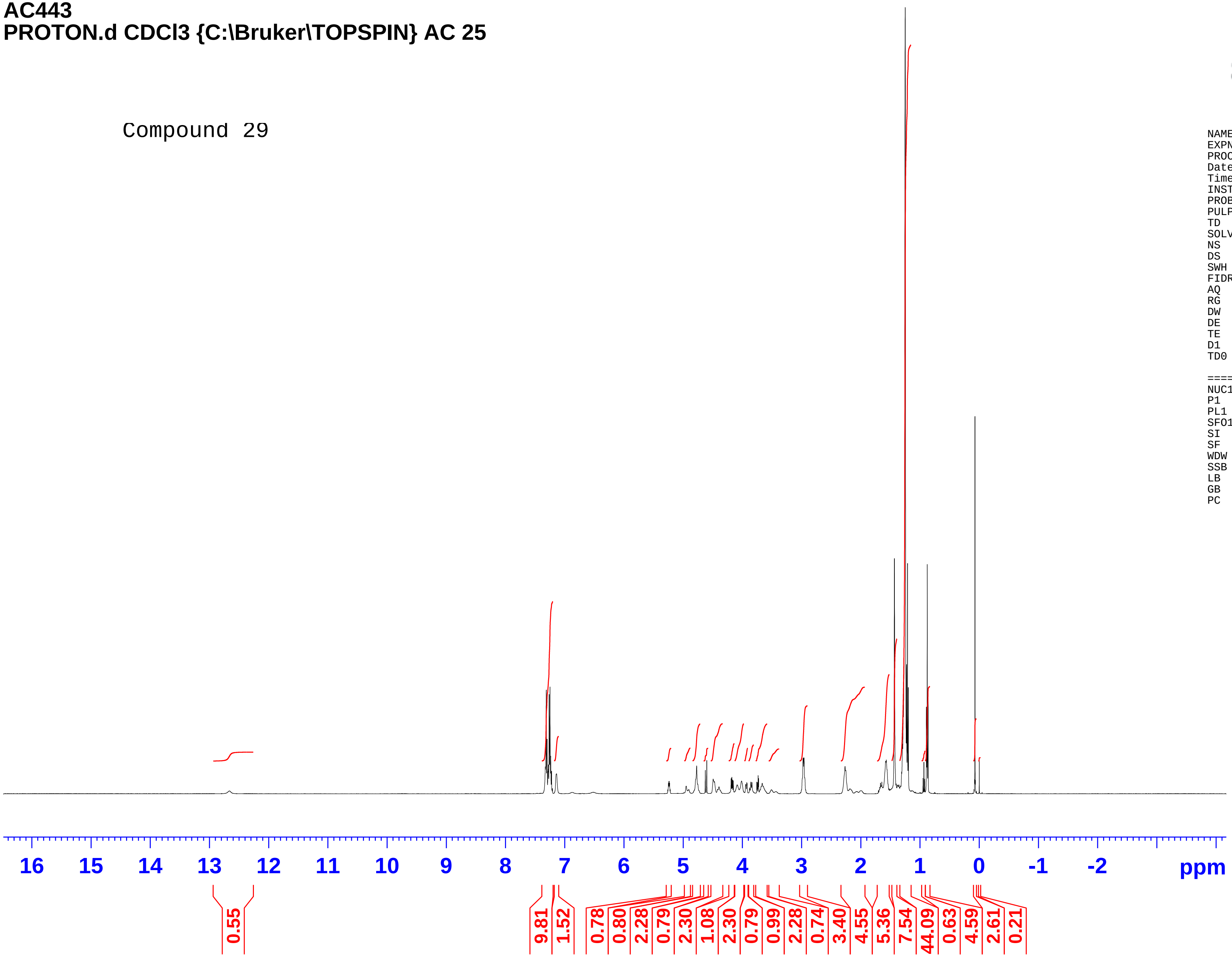

UNIVERSITY OF DUNDEE

NAME	AC-AC443
EXPNO	3
PROCNO	1
Date_	20111028
Time	11.16
INSTRUM	spect
PROBHD	5 mm QNP 1H/13
PULPROG	zg30
TD	65536
SOLVENT	CDCl3
NS	16
DS	2
SWH	10330.578 Hz
FIDRES	0.157632 Hz
AQ	3.1719923 sec
RG	50.8
DW	48.400 usec
DE	6.00 usec
TE	298.2 K
D1	1.00000000 sec
TD0	1

======== CHANNEL f1 ========

NUC1	1H
P1	11.20 usec
PL1	-1.00 dB
SFO1	500.1330885 MHz
SI	65536
SF	500.1300103 MHz
WDW	EM
SSB	0
LB	0.30 Hz
GB	0
PC	1.40

AC446
C13CPDlong.d MeOD {C:\Bruker\TOPSPIN} AC 10

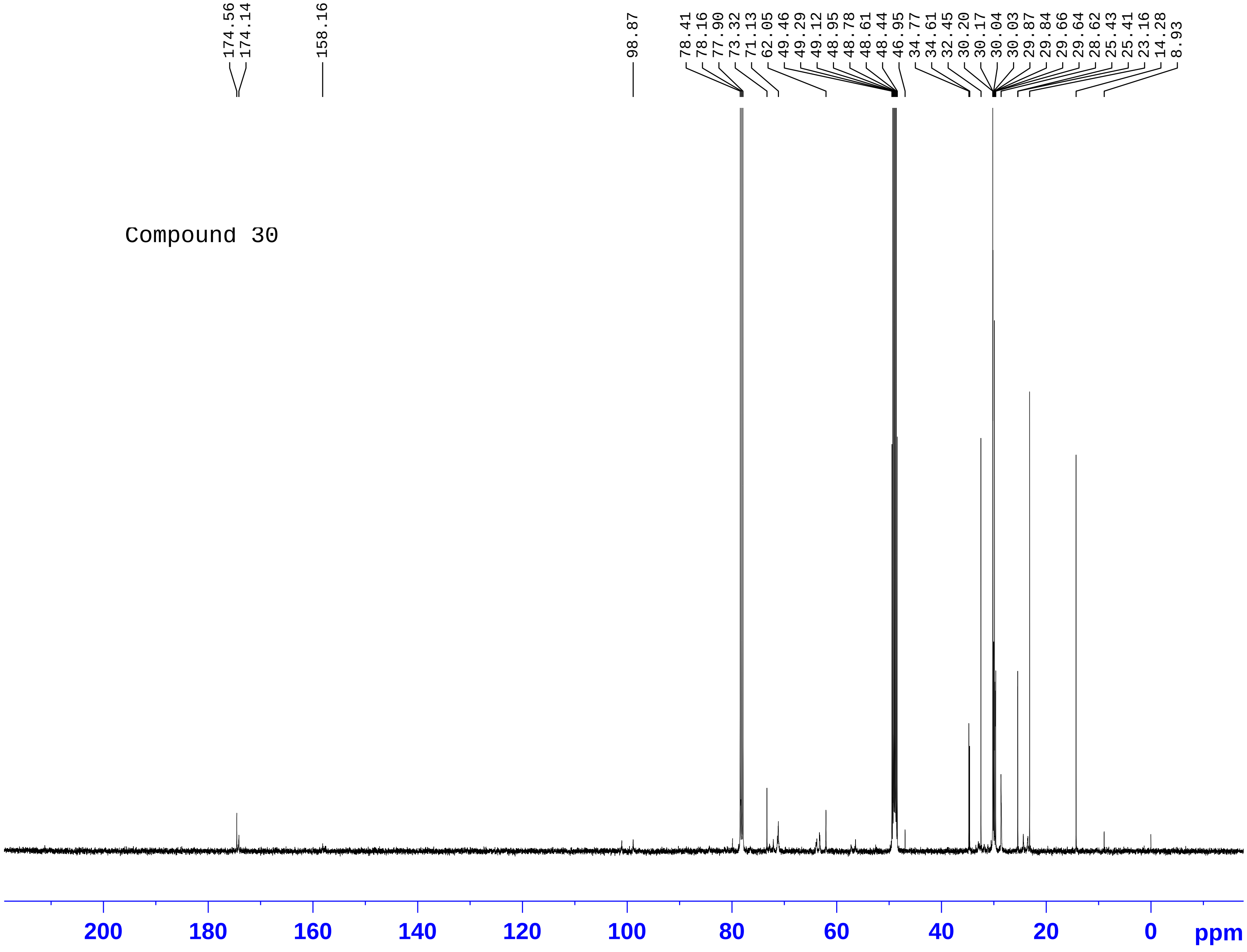

DUNDEE

NAME	AC-AC446
EXPNO	7
PROCNO	1
Date_	20111123
Time	20.02
INSTRUM	spect
PROBHD	5 mm QNP 1H/13
PULPROG	zgpg30
TD	65536
SOLVENT	MeOD
NS	6144
DS	4
SWH	29761.904 Hz
FIDRES	0.454131 Hz
AQ	1.1010548 sec
RG	2050
DW	16.800 usec
DE	6.00 usec
TE	295.3 K
D1	2.00000000 sec
d11	0.03000000 sec
DELTA	1.89999998 sec
TD0	24

======== CHANNEL f1 ========

NUC1	13C
P1	8.18 usec
PL1	0.00 dB
SFO1	125.7703643 MHz

======== CHANNEL f2 ========

CPDPRG2	waltz16
NUC2	1H
PCPD2	80.00 usec
PL2	-1.00 dB
PL12	16.00 dB
PL13	16.00 dB
SFO2	500.1320005 MHz
SI	32768
SF	125.7577079 MHz
WDW	EM
SSB	0
LB	1.00 Hz
GB	0
PC	1.40

SG7 FRACTIONS 3-7
C13CPDfast.d CDCl3 {C:\Bruker\TOPSPIN} AC 4

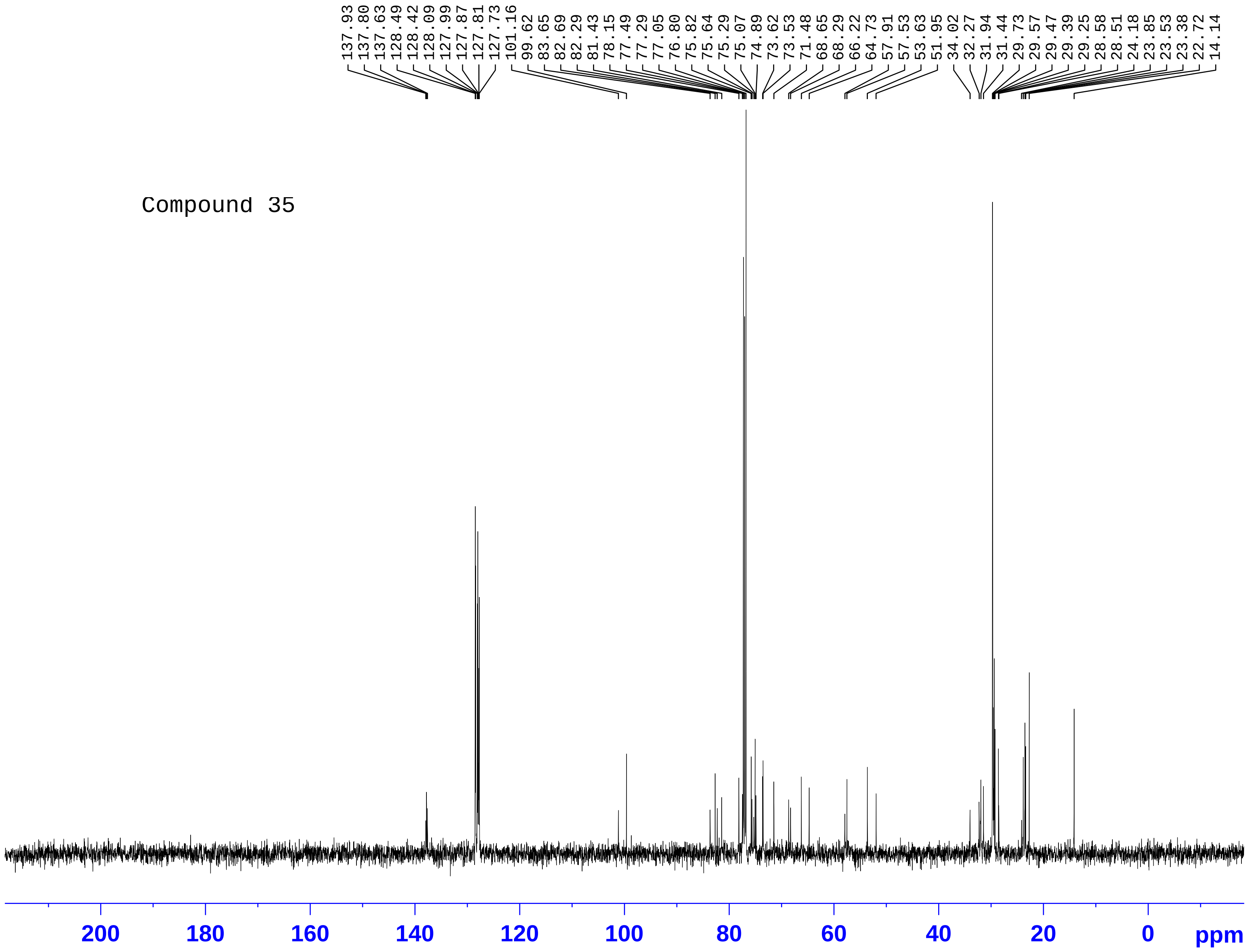

UNIVERSITY OF DUNDEE

NAME	AC-ACSG7	
EXPNO	5	
PROCNO	1	
Date_	20100726	
Time	12.11	
INSTRUM	spect	
PROBHD	5 mm QNP 1H/13	
PULPROG	zgpg30	
TD	16384	
SOLVENT	CDCl3	
NS	800	
DS	4	
SWH	29761.904	Hz
FIDRES	1.816522	Hz
AQ	0.2753012	sec
RG	2050	
DW	16.800	usec
DE	6.00	usec
TE	294.8	K
D1	0.30000001	sec
d11	0.03000000	sec
DELTA	0.20000002	sec
TD0	1	

======== CHANNEL f1 ========

NUC1	13C	
P1	7.80	usec
PL1	0.00	dB
SFO1	125.7703643	MHz

======== CHANNEL f2 ========

CPDPRG2	waltz16	
NUC2	1H	
PCPD2	80.00	usec
PL2	-1.00	dB
PL12	16.00	dB
PL13	16.00	dB
SFO2	500.1320005	MHz
SI	8192	
SF	125.7577890	MHz
WDW	EM	
SSB	0	
LB	1.00	Hz
GB	0	
PC	1.40	

AC446
PROTON.d MeOD {C:\Bruker\TOPSPIN} AC 25

Compound 30

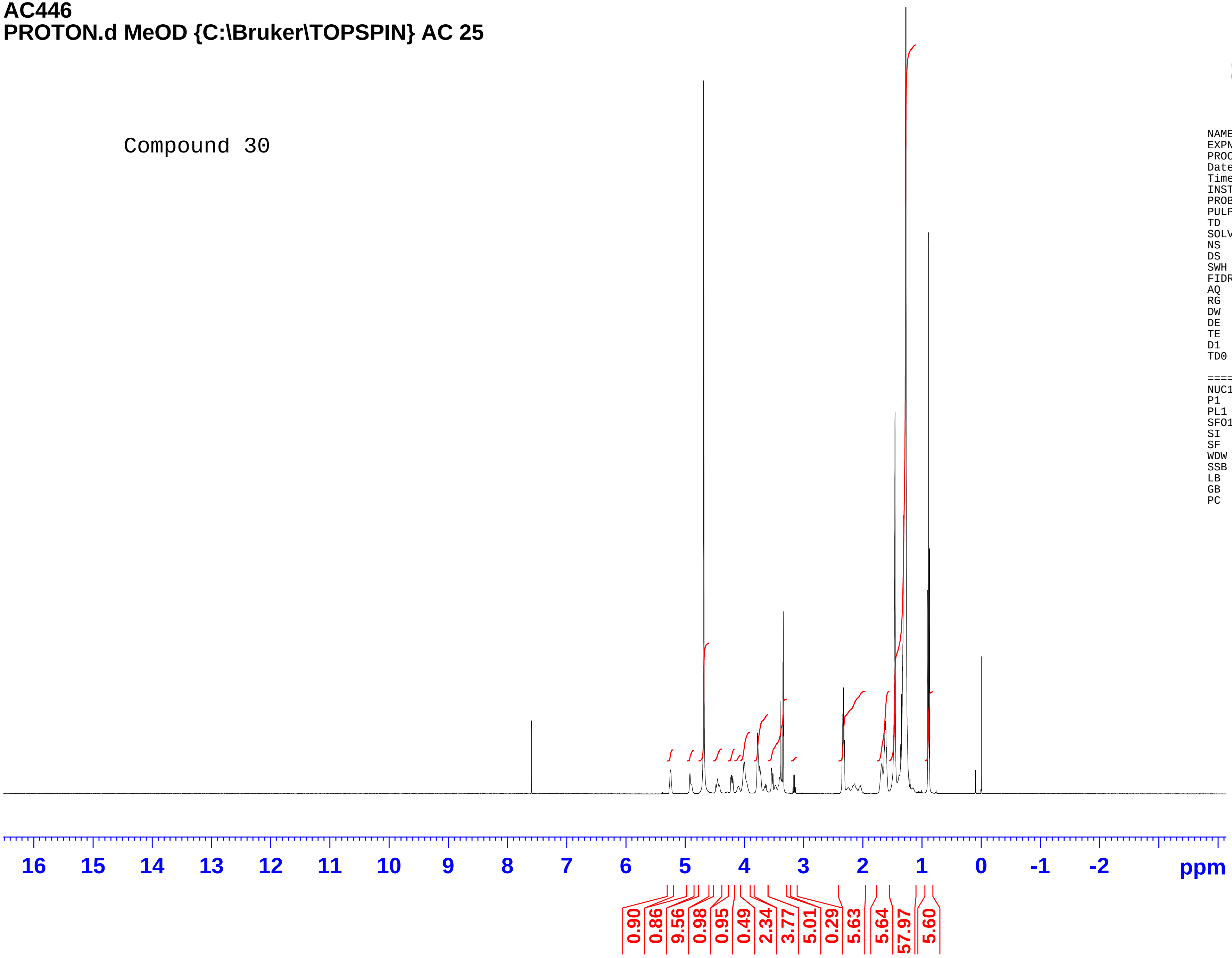

UNIVERSITY OF DUNDEE

NAME	AC-AC446
EXPNO	5
PROCNO	1
Date_	20111121
Time	19.50
INSTRUM	spect
PROBHD	5 mm QNP 1H/13
PULPROG	zg30
TD	65536
SOLVENT	MeOD
NS	16
DS	2
SWH	10330.578 Hz
FIDRES	0.157632 Hz
AQ	3.1719923 sec
RG	161
DW	48.400 usec
DE	6.00 usec
TE	294.3 K
D1	1.00000000 sec
TD0	1

======== CHANNEL f1 ========

NUC1	1H
P1	11.20 usec
PL1	-1.00 dB
SFO1	500.1330885 MHz
SI	65536
SF	500.1299934 MHz
WDW	EM
SSB	0
LB	0.30 Hz
GB	0
PC	1.40

FRACTIONS 5-16 2ND EXPERIMENT SG2
C13CPDfast.d CDCl3 {C:\Bruker\TOPSPIN} AC 7

Compound 32

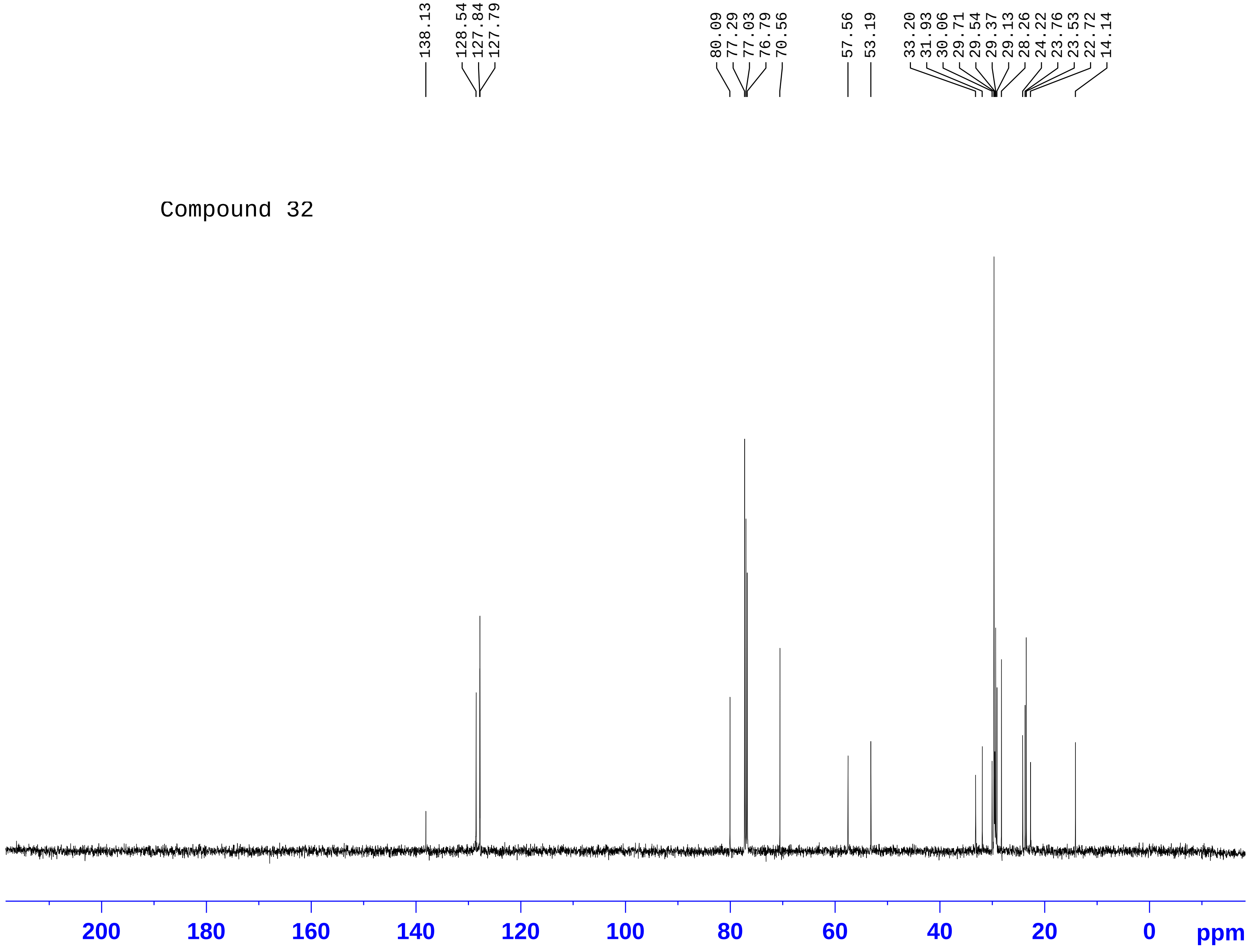

UNIVERSITY OF DUNDEE

NAME	AC-SG2-FR-16	
EXPNO	5	
PROCNO	1	
Date_	20100714	
Time	11.09	
INSTRUM	spect	
PROBHD	5 mm QNP 1H/13	
PULPROG	zgpg30	
TD	16384	
SOLVENT	CDCl3	
NS	800	
DS	4	
SWH	29761.904	Hz
FIDRES	1.816522	Hz
AQ	0.2753012	sec
RG	2050	
DW	16.800	usec
DE	6.00	usec
TE	294.8	K
D1	0.30000001	sec
d11	0.03000000	sec
DELTA	0.20000002	sec
TD0	1	

======== CHANNEL f1 ========

NUC1	13C	
P1	7.80	usec
PL1	0.00	dB
SFO1	125.7703643	MHz

======== CHANNEL f2 ========

CPDPRG2	waltz16	
NUC2	1H	
PCPD2	80.00	usec
PL2	-1.00	dB
PL12	16.00	dB
PL13	16.00	dB
SFO2	500.1320005	MHz
SI	8192	
SF	125.7577890	MHz
WDW	EM	
SSB	0	
LB	1.00	Hz
GB	0	
PC	1.40	

SG7 FRACTIONS 3-7
PROTON.d CDCl3 {C:\Bruker\TOPSPIN} AC 4

Compound 35

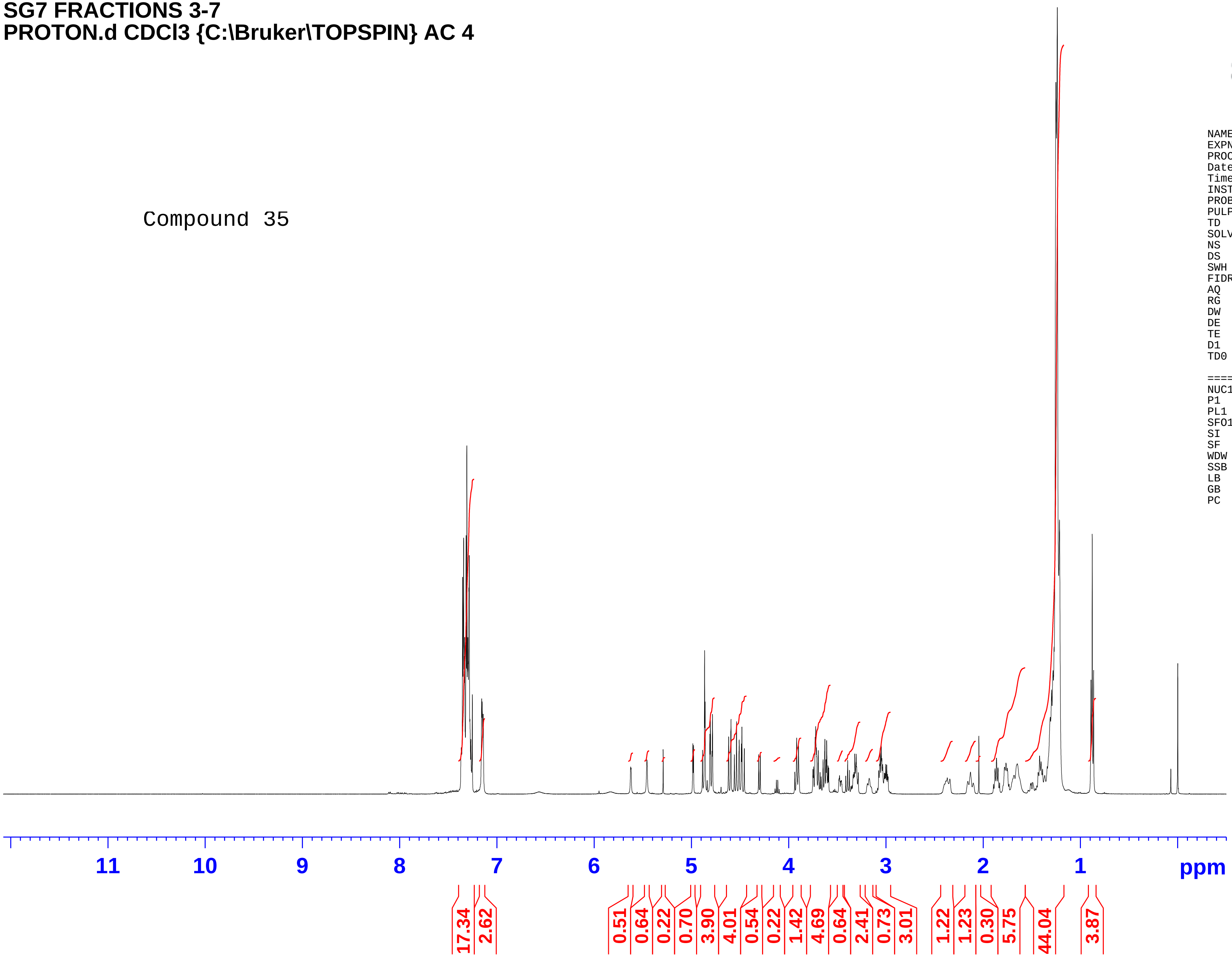

UNIVERSITY OF DUNDEE

NAME	AC-ACSG7
EXPNO	2
PROCNO	1
Date_	20100726
Time	11.42
INSTRUM	spect
PROBHD	5 mm QNP 1H/13
PULPROG	zg30
TD	65536
SOLVENT	CDCl3
NS	16
DS	2
SWH	10330.578 Hz
FIDRES	0.157632 Hz
AQ	3.1719923 sec
RG	144
DW	48.400 usec
DE	6.00 usec
TE	293.3 K
D1	1.00000000 sec
TD0	1

======== CHANNEL f1 ========

NUC1	1H
P1	11.20 usec
PL1	-1.00 dB
SFO1	500.1330885 MHz
SI	65536
SF	500.1300164 MHz
WDW	EM
SSB	0
LB	0.30 Hz
GB	0
PC	1.40

FRACTIONS 5-16 2ND EXPERIMENT SG2
PROTON.d CDCl3 {C:\Bruker\TOPSPIN} AC 7

Compound 32

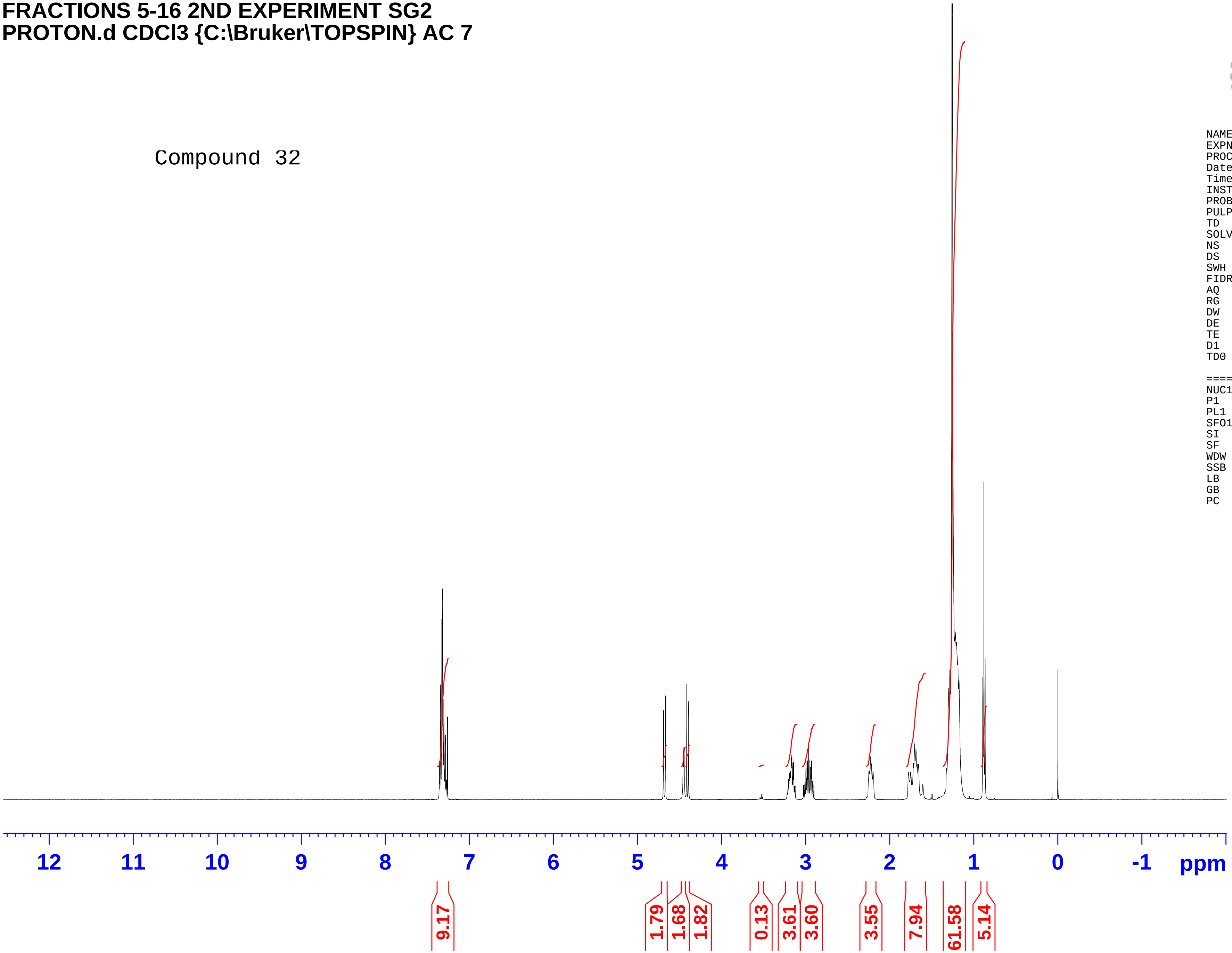

UNIVERSITY OF DUNDEE

NAME	AC-SG2-FR-16	
EXPNO	3	
PROCNO	1	
Date_	20100714	
Time	10.50	
INSTRUM	spect	
PROBHD	5 mm QNP 1H/13	
PULPROG	zg30	
TD	65536	
SOLVENT	CDCl3	
NS	16	
DS	2	
SWH	10330.578	Hz
FIDRES	0.157632	Hz
AQ	3.1719923	sec
RG	144	
DW	48.400	usec
DE	6.00	usec
TE	293.3	K
D1	1.00000000	sec
TD0	1	
======== CHANNEL f1 ========		
NUC1	1H	
P1	11.20	usec
PL1	-1.00	dB
SFO1	500.1330885	MHz
SI	65536	
SF	500.1300130	MHz
WDW	EM	
SSB	0	
LB	0.30	Hz
GB	0	
PC	1.40	

SG DEBENZYLATION
C13CPDfast.d CDCl3 {C:\Bruker\TOPSPIN} AC 2

Compound 33

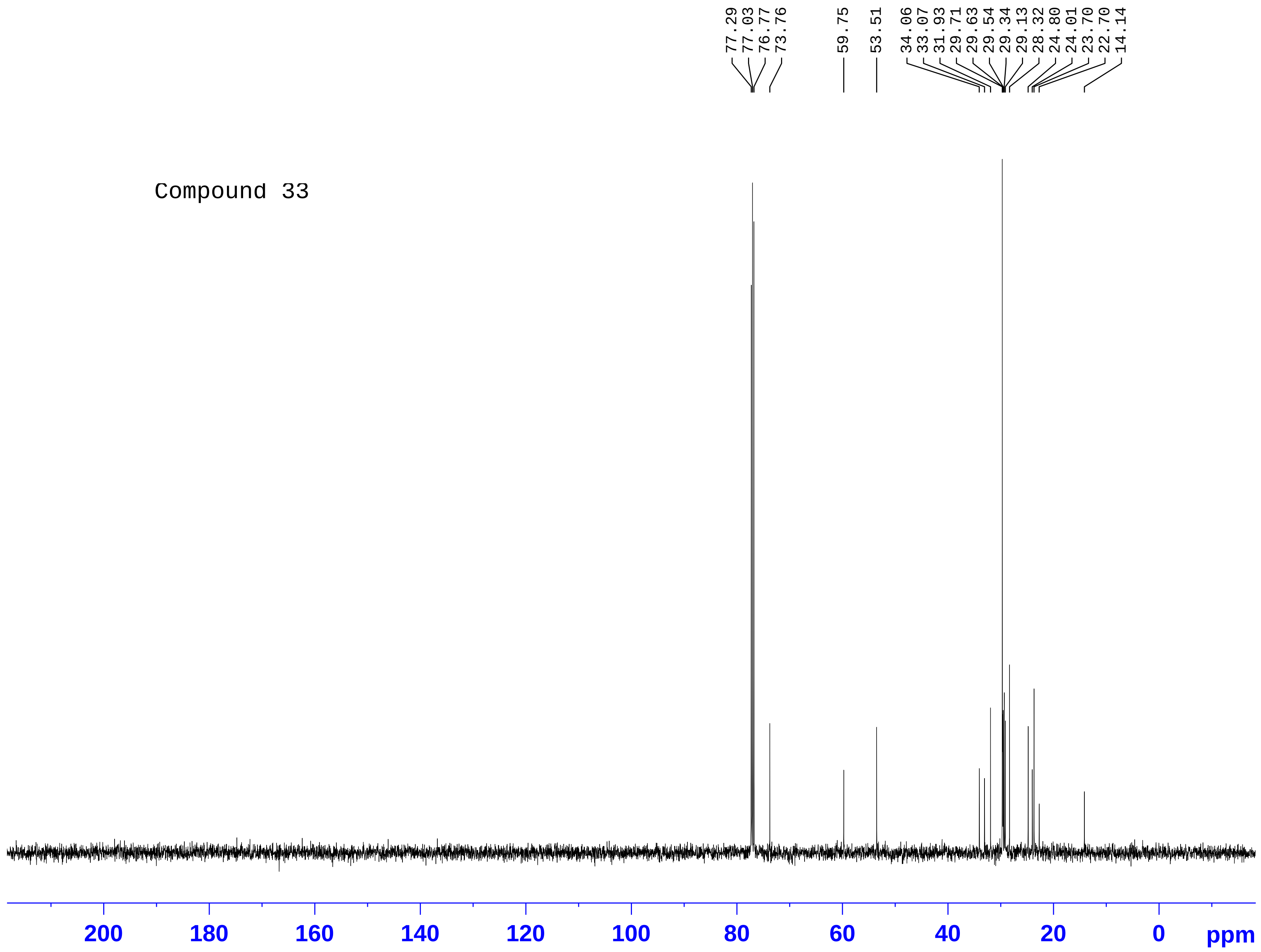

UNIVERSITY OF DUNDEE

NAME	AC-ACSGDEDEBN	
EXPNO	4	
PROCNO	1	
Date_	20100716	
Time	10.27	
INSTRUM	spect	
PROBHD	5 mm QNP 1H/13	
PULPROG	zgpg30	
TD	16384	
SOLVENT	CDCl3	
NS	800	
DS	4	
SWH	29761.904	Hz
FIDRES	1.816522	Hz
AQ	0.2753012	sec
RG	2050	
DW	16.800	usec
DE	6.00	usec
TE	294.8	K
D1	0.30000001	sec
d11	0.03000000	sec
DELTA	0.20000002	sec
TD0	1	
======== CHANNEL f1 ========		
NUC1	13C	
P1	7.80	usec
PL1	0.00	dB
SFO1	125.7703643	MHz
======== CHANNEL f2 ========		
CPDPRG2	waltz16	
NUC2	1H	
PCPD2	80.00	usec
PL2	-1.00	dB
PL12	16.00	dB
PL13	16.00	dB
SFO2	500.1320005	MHz
SI	8192	
SF	125.7577890	MHz
WDW	EM	
SSB	0	
LB	1.00	Hz
GB	0	
PC	1.40	

LWA17B F11-18
C13CPDfast.d CDCl3 {C:\Bruker\TOPSPIN} AC 9

Compound 37

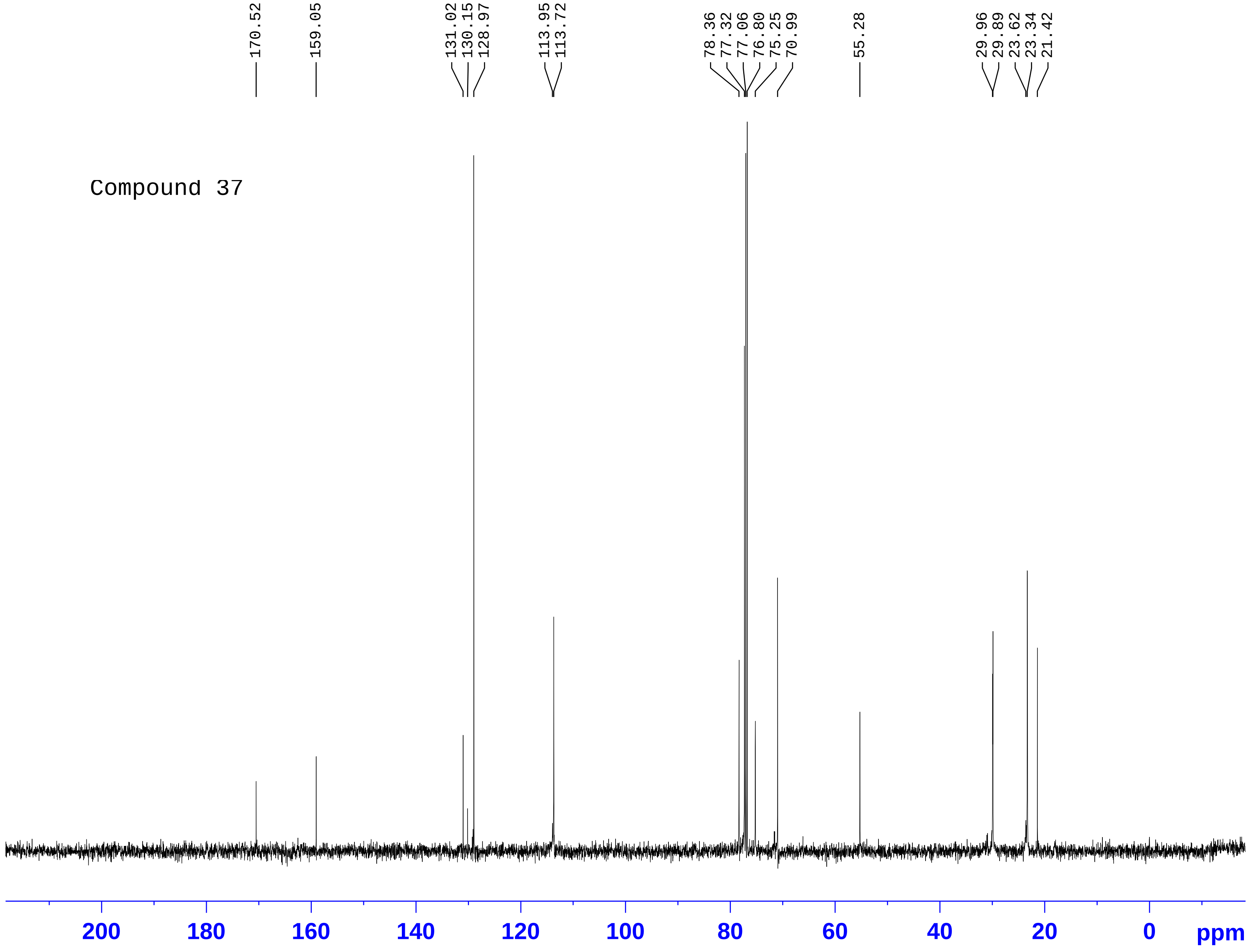

UNIVERSITY OF DUNDEE

```
NAME             AC-LWA17B
EXPNO                    3
PROCNO                   1
Date_             20090825
Time                 11.07
INSTRUM              spect
PROBHD     5 mm QNP 1H/13
PULPROG            zgpg30
TD                   16384
SOLVENT              CDCl3
NS                     800
DS                       4
SWH              29761.904 Hz
FIDRES            1.816522 Hz
AQ               0.2753012 sec
RG                    2050
DW                  16.800 usec
DE                    6.00 usec
TE                   294.2 K
D1              0.30000001 sec
d11             0.03000000 sec
DELTA           0.20000002 sec
TD0                      1

======== CHANNEL f1 ========
NUC1                   13C
P1                    7.80 usec
PL1                   0.00 dB
SFO1           125.7703643 MHz

======== CHANNEL f2 ========
CPDPRG2            waltz16
NUC2                    1H
PCPD2                80.00 usec
PL2                  -1.00 dB
PL12                 16.00 dB
PL13                 16.00 dB
SFO2           500.1320005 MHz
SI                    8192
SF             125.7577890 MHz
WDW                     EM
SSB                      0
LB                    1.00 Hz
GB                       0
PC                    1.40
```

SG DEBENZYLATION
PROTON.d CDCl3 {C:\Bruker\TOPSPIN} AC 2

Compound 33

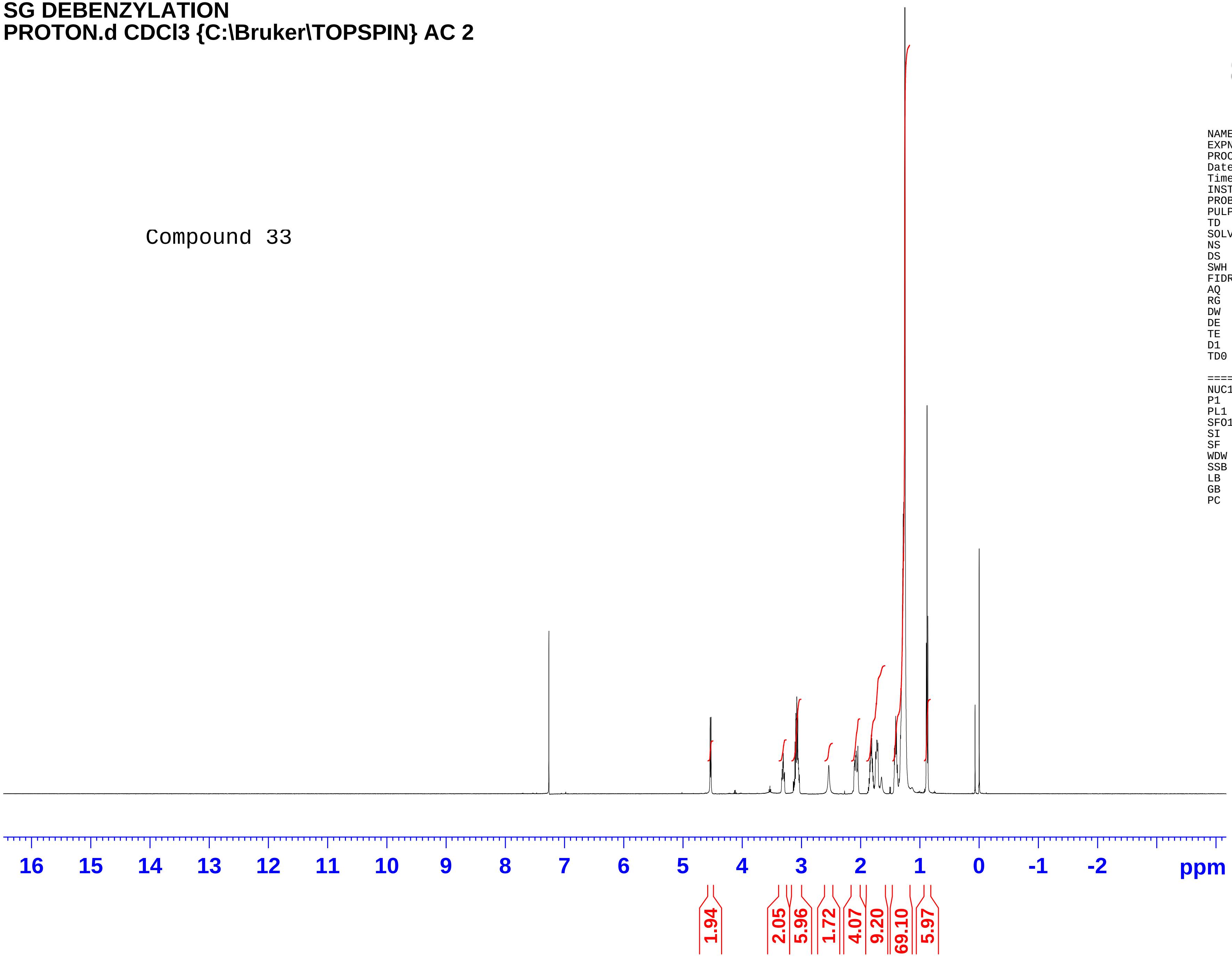

UNIVERSITY OF DUNDEE

NAME	AC-ACSGDEDEBN
EXPNO	2
PROCNO	1
Date_	20100716
Time	10.07
INSTRUM	spect
PROBHD	5 mm QNP 1H/13
PULPROG	zg30
TD	65536
SOLVENT	CDCl3
NS	16
DS	2
SWH	10330.578 Hz
FIDRES	0.157632 Hz
AQ	3.1719923 sec
RG	228
DW	48.400 usec
DE	6.00 usec
TE	293.4 K
D1	1.00000000 sec
TD0	1

======== CHANNEL f1 ========

NUC1	1H
P1	11.20 usec
PL1	-1.00 dB
SFO1	500.1330885 MHz
SI	65536
SF	500.1300109 MHz
WDW	EM
SSB	0
LB	0.30 Hz
GB	0
PC	1.40

OMBNDIOL
PROTON.d CDCl3 {C:\Bruker\TOPSPIN} IG 1

Compound 37

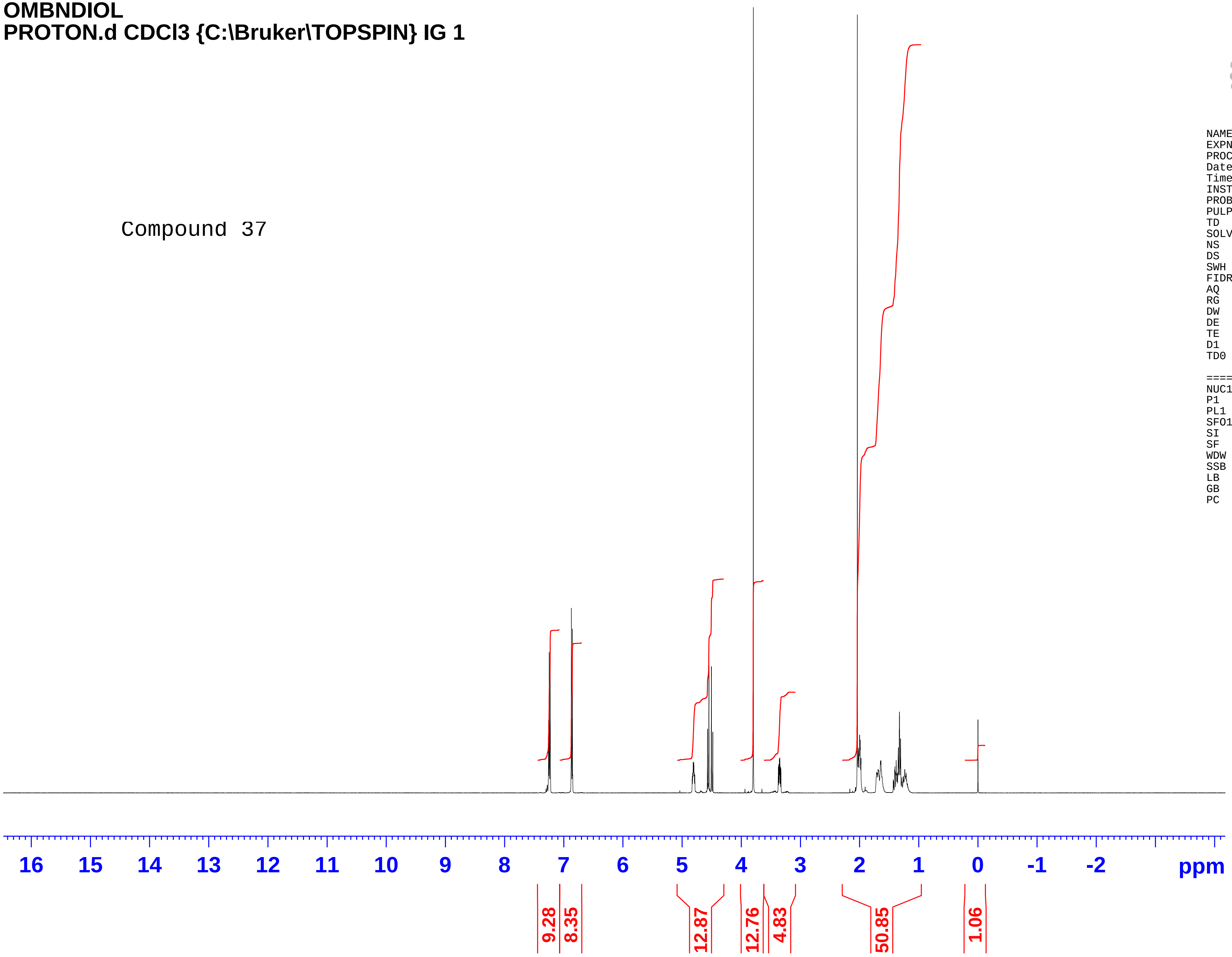

UNIVERSITY OF DUNDEE

NAME	IG-AC-OMBNDIOLCHECK
EXPNO	1
PROCNO	1
Date_	20100119
Time	10.03
INSTRUM	spect
PROBHD	5 mm QNP 1H/13
PULPROG	zg30
TD	65536
SOLVENT	CDCl3
NS	16
DS	2
SWH	10330.578 Hz
FIDRES	0.157632 Hz
AQ	3.1719923 sec
RG	228
DW	48.400 usec
DE	6.00 usec
TE	300.2 K
D1	1.00000000 sec
TD0	1
======== CHANNEL f1 ========	
NUC1	1H
P1	11.20 usec
PL1	-1.00 dB
SFO1	500.1330885 MHz
SI	65536
SF	500.1300142 MHz
WDW	EM
SSB	0
LB	0.30 Hz
GB	0
PC	1.40

ALLYLOMBNDIOL
C13CPD.d CDCl3 {C:\Bruker\TOPSPIN} IG 12

Compound 38

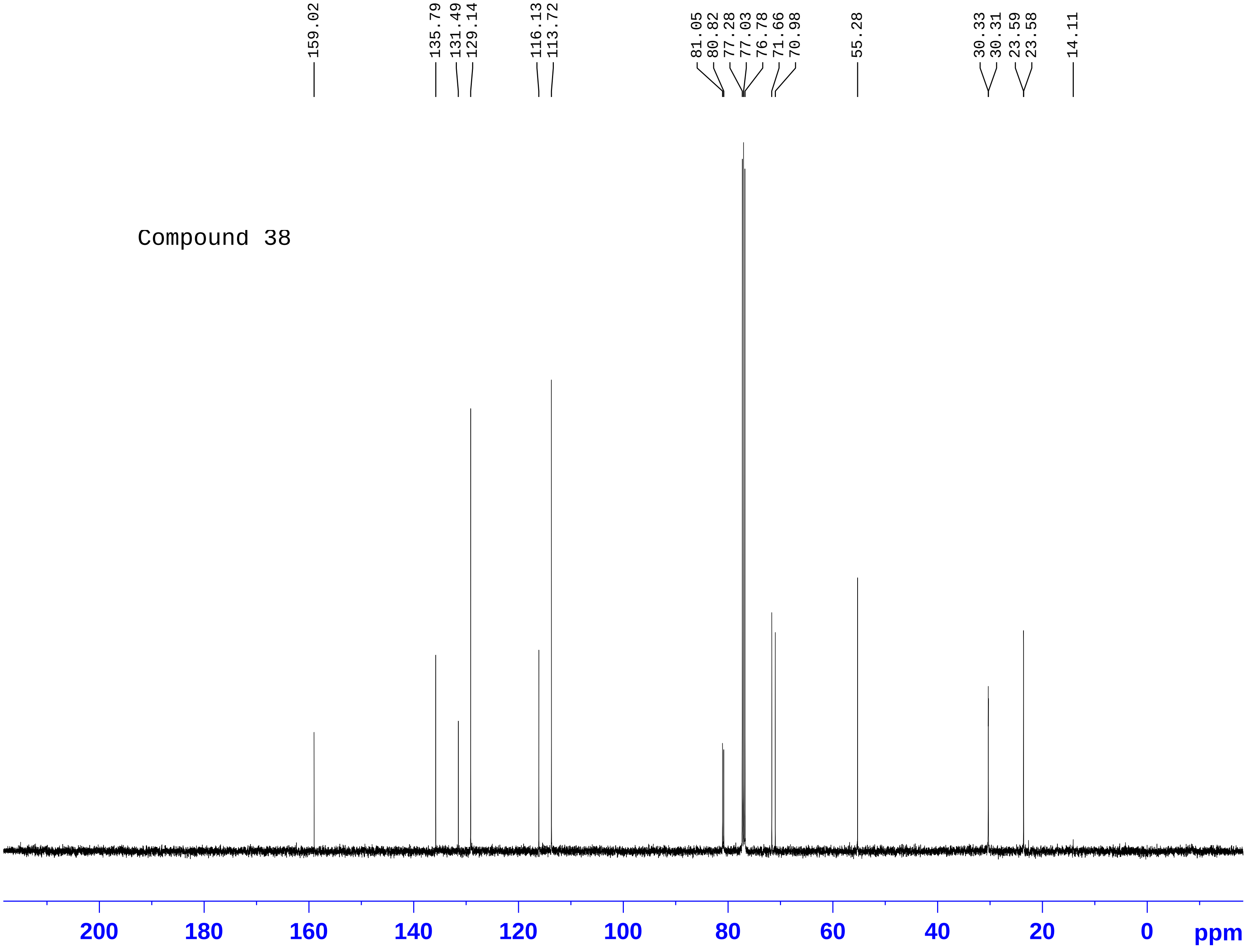

UNIVERSITY OF DUNDEE

NAME	IG-AC-ALLYLOMBNDIOLPURE
EXPNO	2
PROCNO	1
Date_	20100114
Time	17.28
INSTRUM	spect
PROBHD	5 mm QNP 1H/13
PULPROG	zgpg30
TD	65536
SOLVENT	CDCl3
NS	256
DS	4
SWH	29761.904 Hz
FIDRES	0.454131 Hz
AQ	1.1010548 sec
RG	2050
DW	16.800 usec
DE	6.00 usec
TE	300.2 K
D1	2.00000000 sec
d11	0.03000000 sec
DELTA	1.89999998 sec
TD0	1

======== CHANNEL f1 ========

NUC1	13C
P1	7.80 usec
PL1	0.00 dB
SFO1	125.7703643 MHz

======== CHANNEL f2 ========

CPDPRG2	waltz16
NUC2	1H
PCPD2	80.00 usec
PL2	-1.00 dB
PL12	16.00 dB
PL13	16.00 dB
SFO2	500.1320005 MHz
SI	32768
SF	125.7577890 MHz
WDW	EM
SSB	0
LB	1.00 Hz
GB	0
PC	1.40

ALLYLOMBNDIOL
PROTON.d CDCl3 {C:\Bruker\TOPSPIN} IG 12

Compound 38

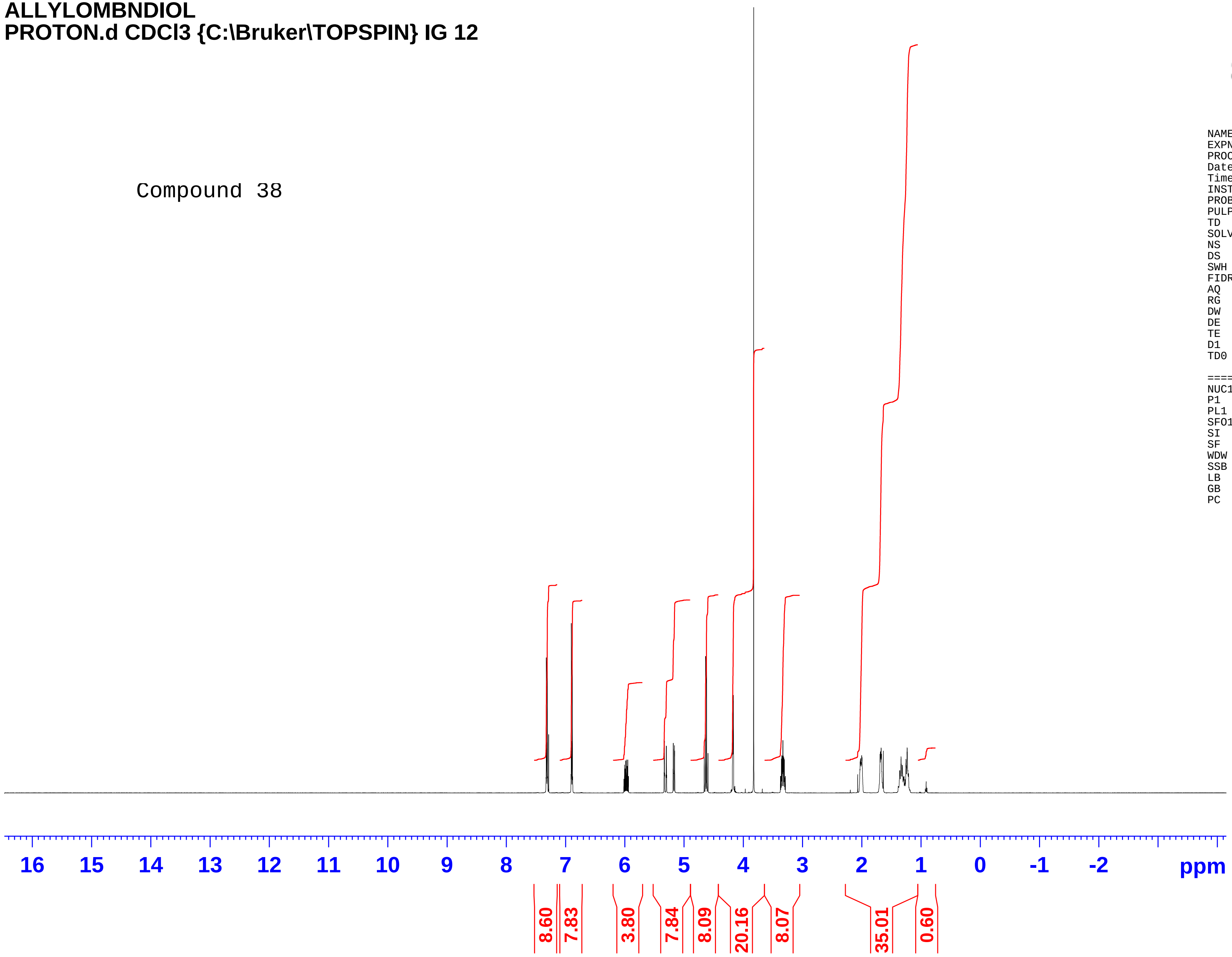

UNIVERSITY OF DUNDEE

NAME	IG-AC-ALLYLOMBNDIOLPURE
EXPNO	1
PROCNO	1
Date_	20100114
Time	17.13
INSTRUM	spect
PROBHD	5 mm QNP 1H/13
PULPROG	zg30
TD	65536
SOLVENT	CDCl3
NS	16
DS	2
SWH	10330.578 Hz
FIDRES	0.157632 Hz
AQ	3.1719923 sec
RG	181
DW	48.400 usec
DE	6.00 usec
TE	300.2 K
D1	1.00000000 sec
TD0	1
======== CHANNEL f1 ========	
NUC1	1H
P1	11.20 usec
PL1	-1.00 dB
SFO1	500.1330885 MHz
SI	65536
SF	500.1300000 MHz
WDW	EM
SSB	0
LB	0.30 Hz
GB	0
PC	1.40

EPOXOMBNDIOL
C13CPD.d CDCl3 {C:\Bruker\TOPSPIN} IG 10

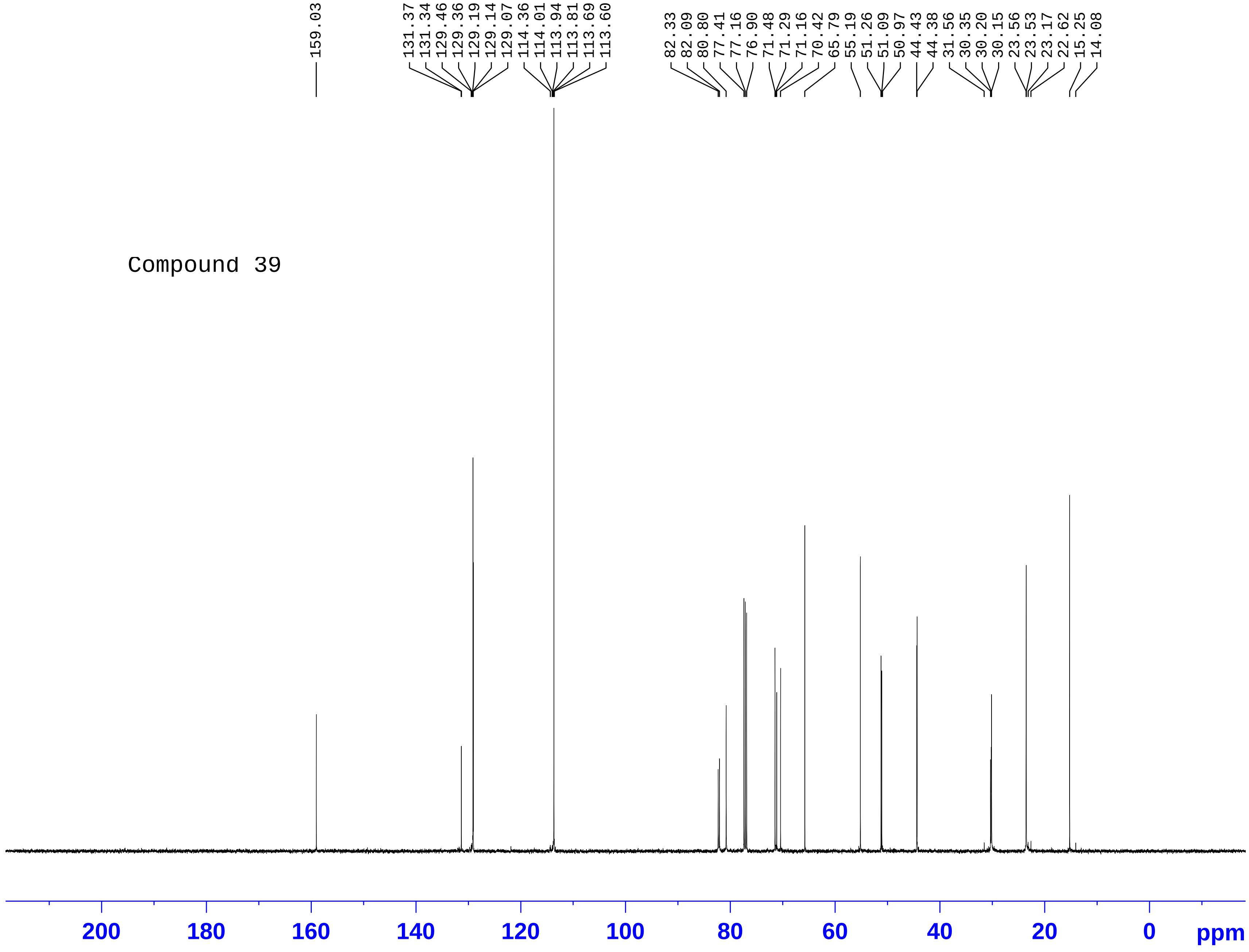

UNIVERSITY OF DUNDEE

NAME	IG-AC-EPOXOMBNDIOL PURE	
EXPNO	7	
PROCNO	1	
Date_	20100128	
Time	15.11	
INSTRUM	spect	
PROBHD	5 mm QNP 1H/13	
PULPROG	zgpg30	
TD	65536	
SOLVENT	CDCl3	
NS	256	
DS	4	
SWH	29761.904	Hz
FIDRES	0.454131	Hz
AQ	1.1010548	sec
RG	2050	
DW	16.800	usec
DE	6.00	usec
TE	300.2	K
D1	2.00000000	sec
d11	0.03000000	sec
DELTA	1.89999998	sec
TD0	1	
======== CHANNEL f1 ========		
NUC1	13C	
P1	7.80	usec
PL1	0.00	dB
SFO1	125.7703643	MHz
======== CHANNEL f2 ========		
CPDPRG2	waltz16	
NUC2	1H	
PCPD2	80.00	usec
PL2	-1.00	dB
PL12	16.00	dB
PL13	16.00	dB
SFO2	500.1320005	MHz
SI	32768	
SF	125.7577890	MHz
WDW	EM	
SSB	0	
LB	1.00	Hz
GB	0	
PC	1.40	

EPOXYOMBNDIOL
PROTON.d CDCl3 {C:\Bruker\TOPSPIN} IG 22

Compound 39

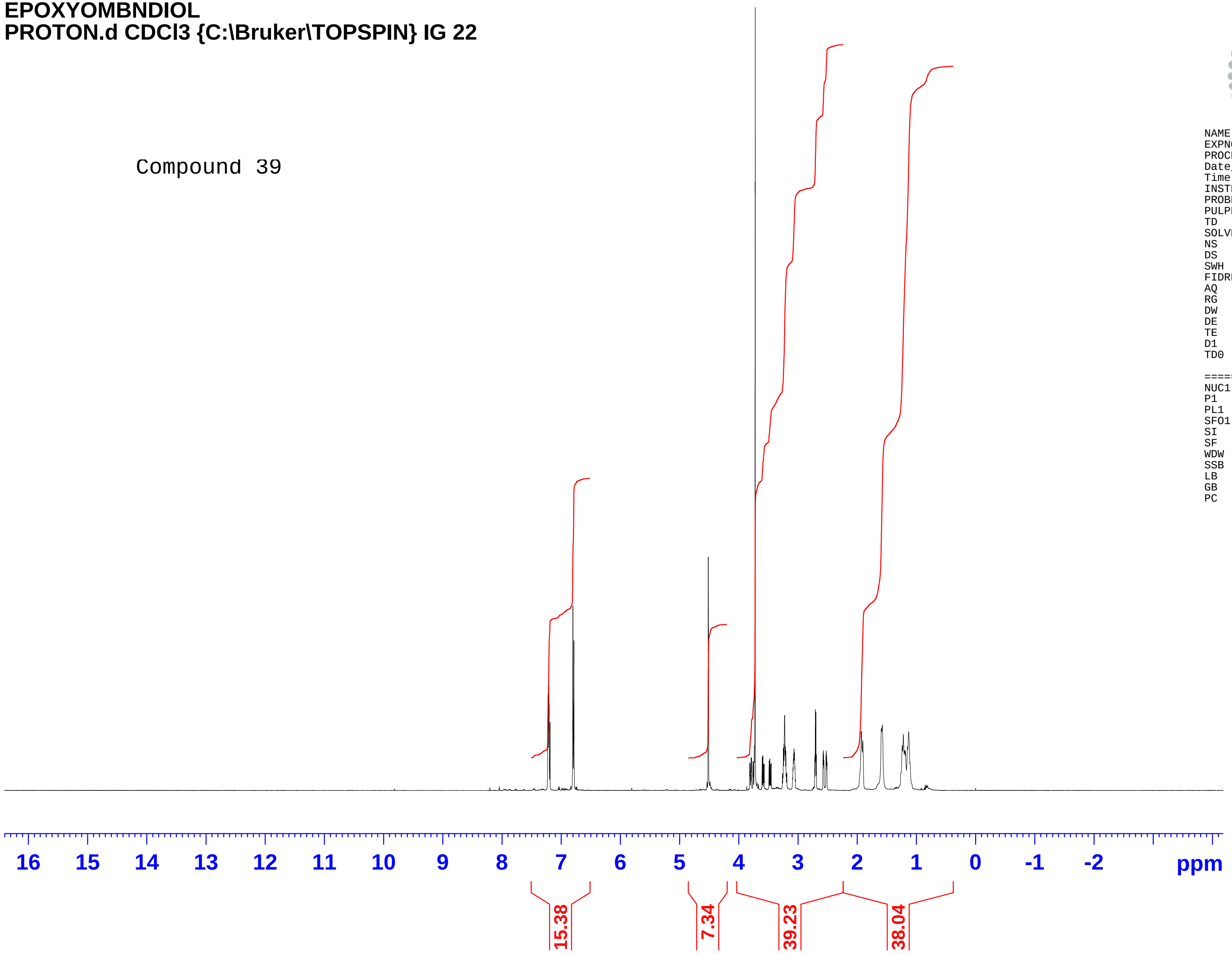

UNIVERSITY OF DUNDEE

NAME	IG-AC-EPOXOMBNDIOL PURE
EXPNO	1
PROCNO	1
Date_	20100120
Time	11.10
INSTRUM	spect
PROBHD	5 mm QNP 1H/13
PULPROG	zg30
TD	65536
SOLVENT	CDCl3
NS	16
DS	2
SWH	10330.578 Hz
FIDRES	0.157632 Hz
AQ	3.1719923 sec
RG	161
DW	48.400 usec
DE	6.00 usec
TE	300.2 K
D1	1.00000000 sec
TD0	1

======== CHANNEL f1 ========

NUC1	1H
P1	11.20 usec
PL1	-1.00 dB
SFO1	500.1330885 MHz
SI	65536
SF	500.1300481 MHz
WDW	EM
SSB	0
LB	0.30 Hz
GB	0
PC	1.40

DIOLOMBNDIOLDMSO
C13CPD.d CDCl3 {C:\Bruker\TOPSPIN} IG 1

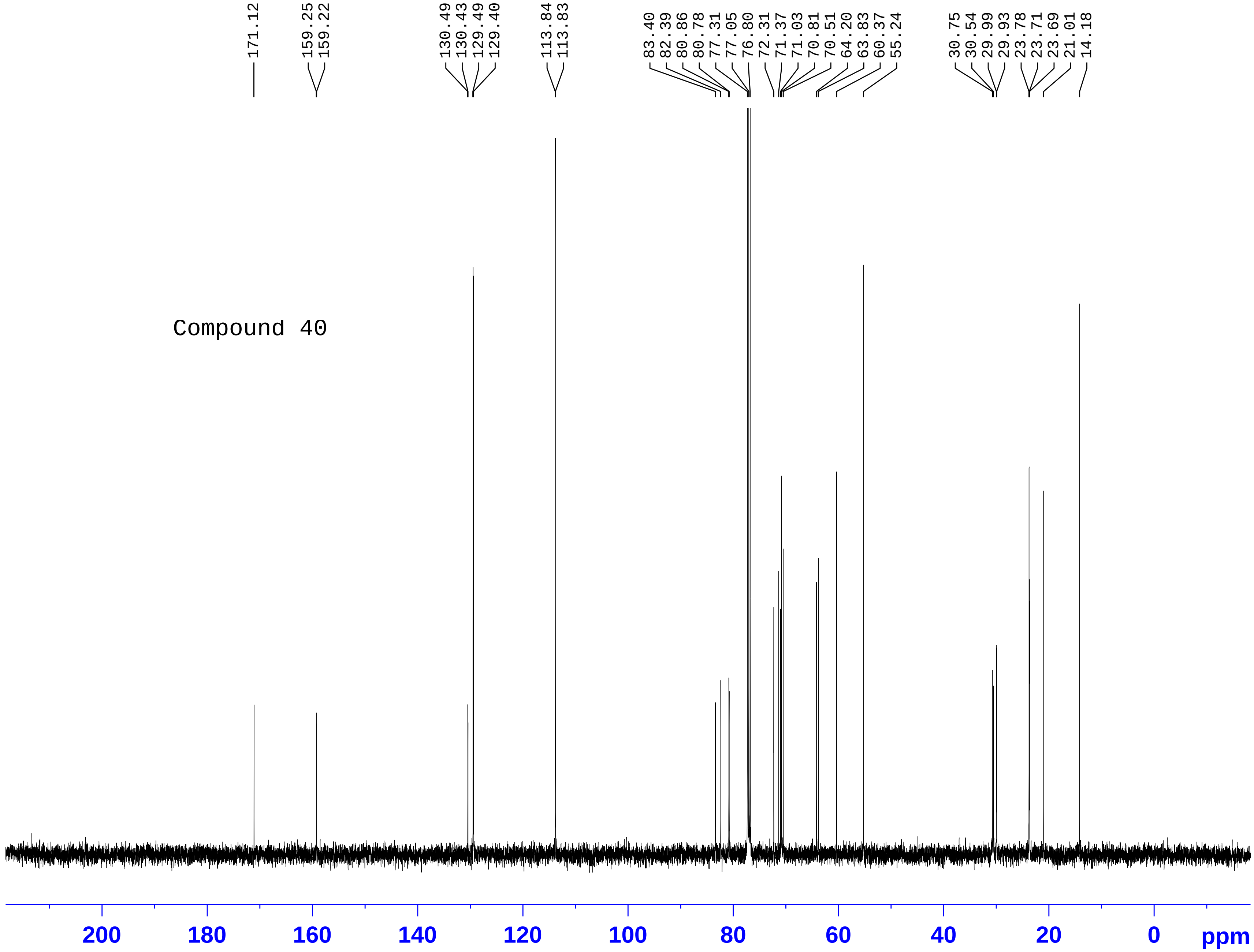

UNIVERSITY OF DUNDEE

NAME	IG-AC-DIOLOMBNDIOLDMSO
EXPNO	3
PROCNO	1
Date_	20100204
Time	10.29
INSTRUM	spect
PROBHD	5 mm QNP 1H/13
PULPROG	zgpg30
TD	65536
SOLVENT	CDCl3
NS	256
DS	4
SWH	29761.904 Hz
FIDRES	0.454131 Hz
AQ	1.1010548 sec
RG	2050
DW	16.800 usec
DE	6.00 usec
TE	300.2 K
D1	2.00000000 sec
d11	0.03000000 sec
DELTA	1.89999998 sec
TD0	1

======== CHANNEL f1 ========

NUC1	13C
P1	7.80 usec
PL1	0.00 dB
SFO1	125.7703643 MHz

======== CHANNEL f2 ========

CPDPRG2	waltz16
NUC2	1H
PCPD2	80.00 usec
PL2	-1.00 dB
PL12	16.00 dB
PL13	16.00 dB
SFO2	500.1320005 MHz
SI	32768
SF	125.7577890 MHz
WDW	EM
SSB	0
LB	1.00 Hz
GB	0
PC	1.40

DIOLOMBNDIOLDMSO
PROTON.d CDCl3 {C:\Bruker\TOPSPIN} IG 1

Compound 40

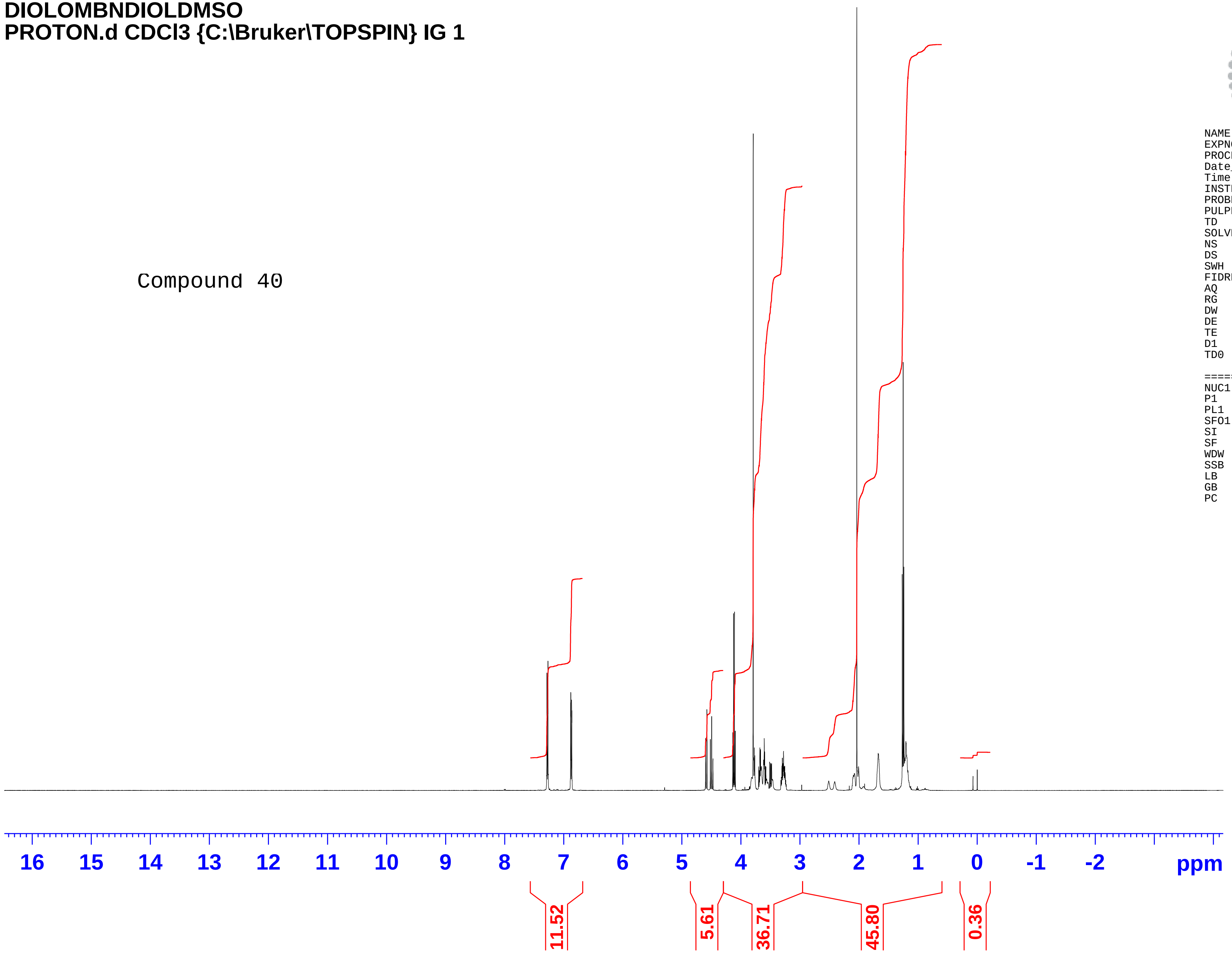

UNIVERSITY OF DUNDEE

NAME	IG-AC-DIOLOMBNDIOLDMSO
EXPNO	1
PROCNO	1
Date_	20100204
Time	10.04
INSTRUM	spect
PROBHD	5 mm QNP 1H/13
PULPROG	zg30
TD	65536
SOLVENT	CDCl3
NS	16
DS	2
SWH	10330.578 Hz
FIDRES	0.157632 Hz
AQ	3.1719923 sec
RG	80.6
DW	48.400 usec
DE	6.00 usec
TE	300.2 K
D1	1.00000000 sec
TD0	1

======== CHANNEL f1 ========

NUC1	1H
P1	11.20 usec
PL1	-1.00 dB
SFO1	500.1330885 MHz
SI	65536
SF	500.1300072 MHz
WDW	EM
SSB	0
LB	0.30 Hz
GB	0
PC	1.40

TBDPS OMBNDIOL
C13CPD.d CDCl3 {C:\Bruker\TOPSPIN} IG 16

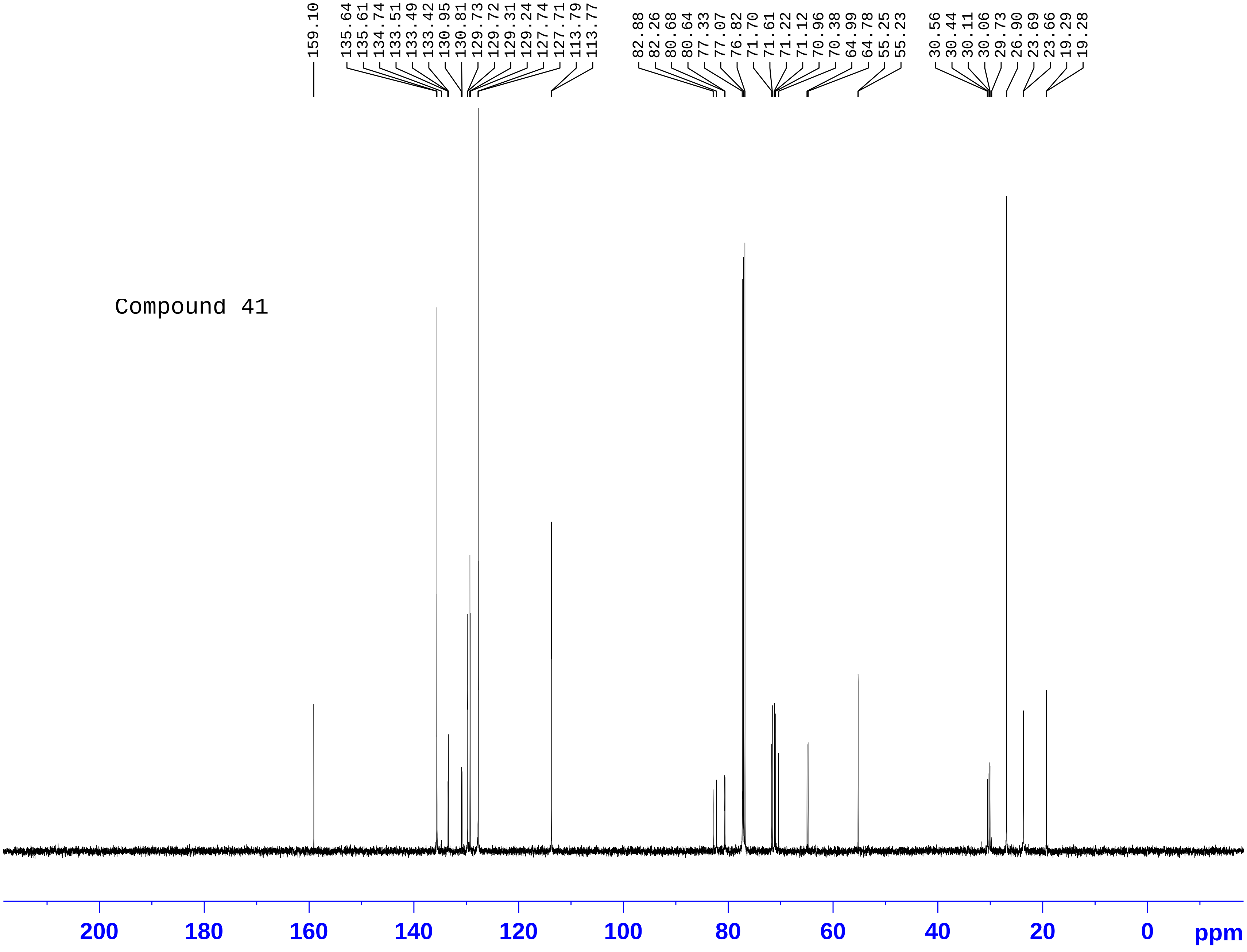

UNIVERSITY OF
DUNDEE

NAME	IG-AC-TBDPS DIOLOMBN
EXPNO	3
PROCNO	1
Date_	20100212
Time	16.10
INSTRUM	spect
PROBHD	5 mm QNP 1H/13
PULPROG	zgpg30
TD	65536
SOLVENT	CDCl3
NS	256
DS	4
SWH	29761.904 Hz
FIDRES	0.454131 Hz
AQ	1.1010548 sec
RG	2050
DW	16.800 usec
DE	6.00 usec
TE	300.2 K
D1	2.00000000 sec
d11	0.03000000 sec
DELTA	1.89999998 sec
TD0	1

======== CHANNEL f1 ========

NUC1	13C
P1	7.80 usec
PL1	0.00 dB
SFO1	125.7703643 MHz

======== CHANNEL f2 ========

CPDPRG2	waltz16
NUC2	1H
PCPD2	80.00 usec
PL2	-1.00 dB
PL12	16.00 dB
PL13	16.00 dB
SFO2	500.1320005 MHz
SI	32768
SF	125.7577890 MHz
WDW	EM
SSB	0
LB	1.00 Hz
GB	0
PC	1.40

TBDPS OMBNDIOL
PROTON.d CDCl3 {C:\Bruker\TOPSPIN} IG 16

Compound 41

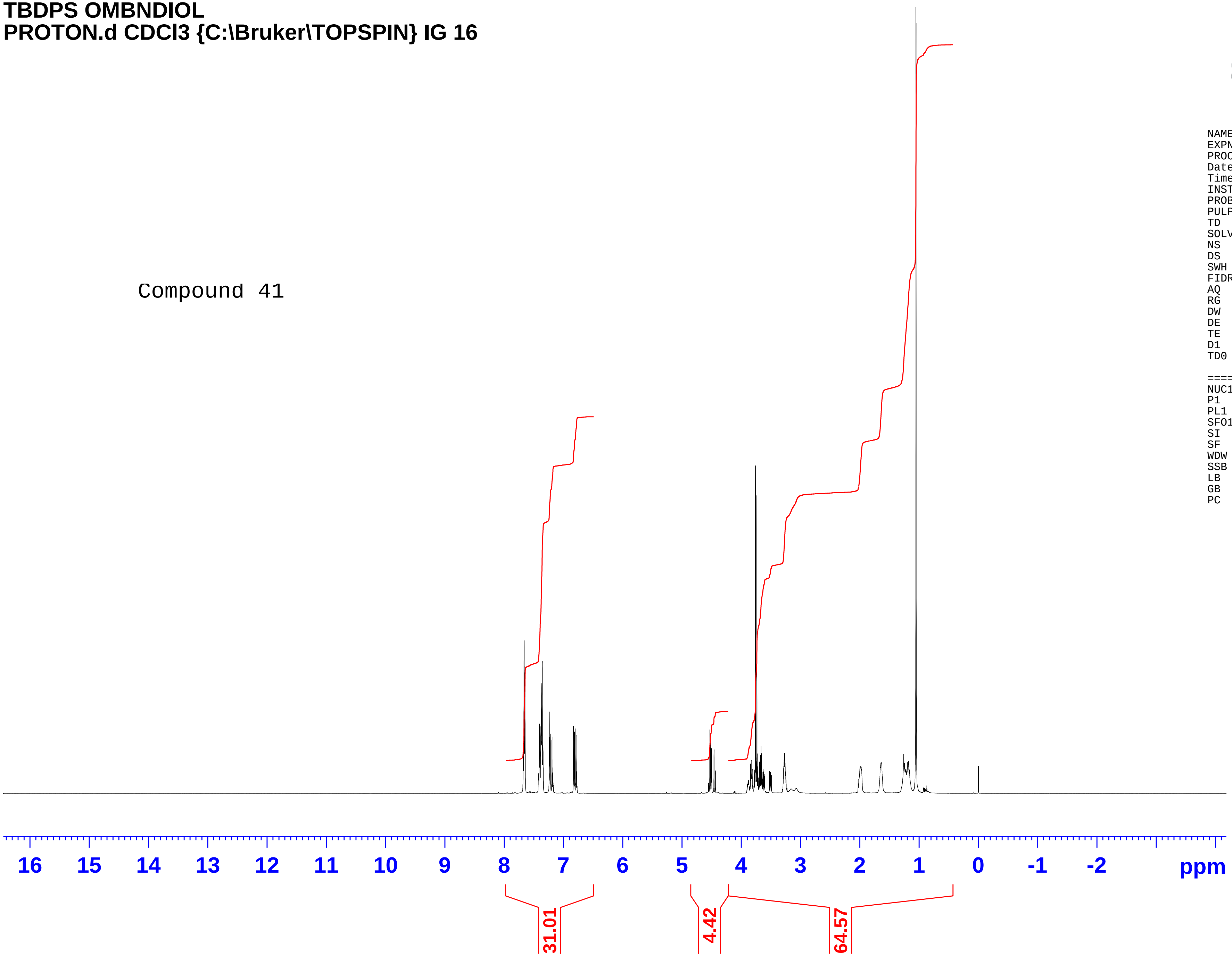

UNIVERSITY OF DUNDEE

NAME	IG-AC-TBDPS DIOLOMBN
EXPNO	1
PROCNO	1
Date_	20100212
Time	15.45
INSTRUM	spect
PROBHD	5 mm QNP 1H/13
PULPROG	zg30
TD	65536
SOLVENT	CDCl3
NS	16
DS	2
SWH	10330.578 Hz
FIDRES	0.157632 Hz
AQ	3.1719923 sec
RG	57
DW	48.400 usec
DE	6.00 usec
TE	300.2 K
D1	1.00000000 sec
TD0	1

======== CHANNEL f1 ========

NUC1	1H
P1	11.20 usec
PL1	-1.00 dB
SFO1	500.1330885 MHz
SI	65536
SF	500.1300265 MHz
WDW	EM
SSB	0
LB	0.30 Hz
GB	0
PC	1.40

OMS F2
C13CPD.d CDCl3 {C:\Bruker\TOPSPIN} IG 43

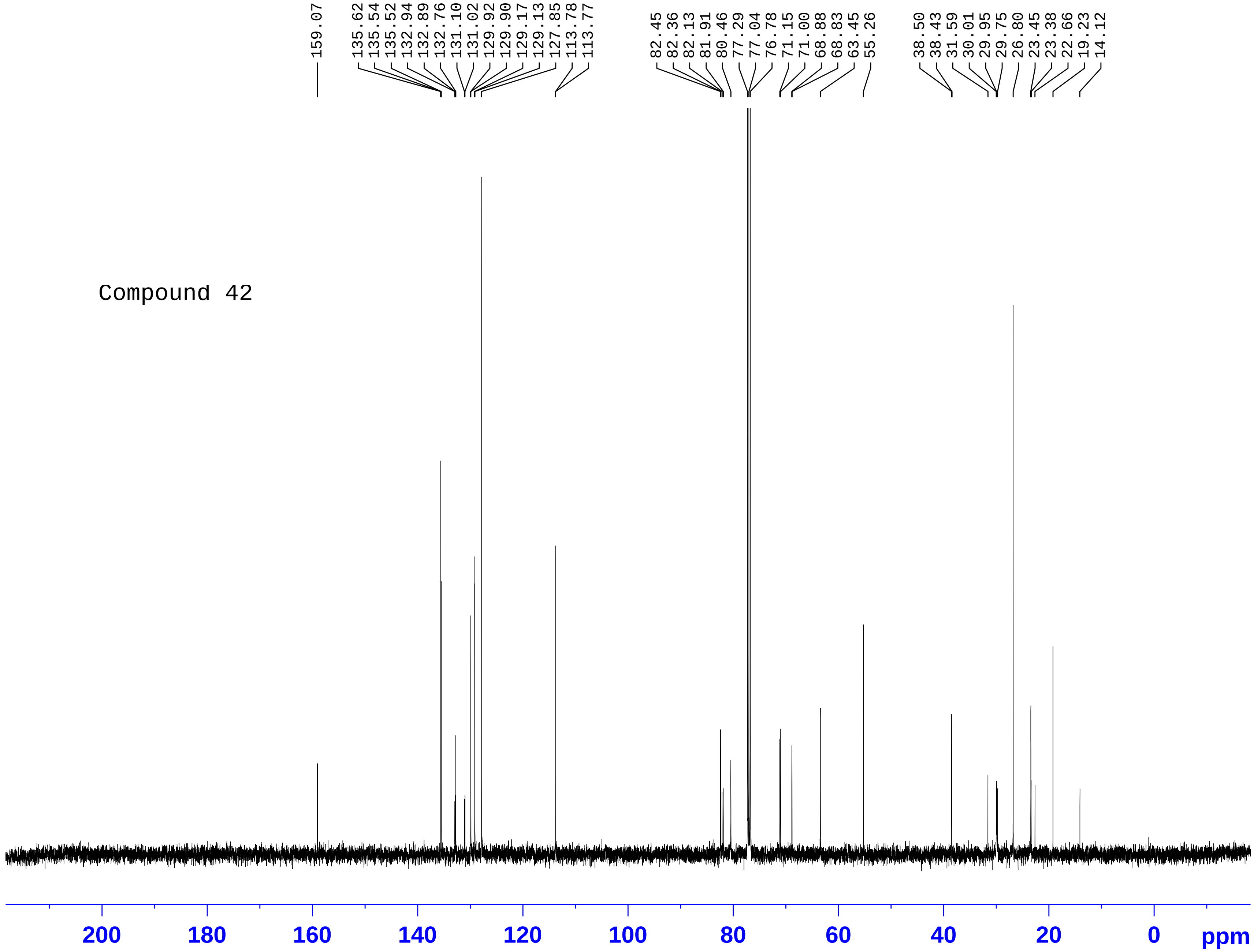


```
NAME       IG-AC-OMSOMBNDIOF2
EXPNO                       3
PROCNO                      1
Date_                20100430
Time                    14.47
INSTRUM                 spect
PROBHD      5 mm QNP 1H/13
PULPROG               zgpg30
TD                      65536
SOLVENT                 CDCl3
NS                        256
DS                          4
SWH                 29761.904 Hz
FIDRES               0.454131 Hz
AQ                  1.1010548 sec
RG                       2050
DW                     16.800 usec
DE                       6.00 usec
TE                      300.2 K
D1                 2.00000000 sec
d11                0.03000000 sec
DELTA              1.89999998 sec
TD0                         1

======== CHANNEL f1 ========
NUC1                      13C
P1                       7.80 usec
PL1                      0.00 dB
SFO1              125.7703643 MHz

======== CHANNEL f2 ========
CPDPRG2               waltz16
NUC2                       1H
PCPD2                   80.00 usec
PL2                     -1.00 dB
PL12                    16.00 dB
PL13                    16.00 dB
SFO2              500.1320005 MHz
SI                      32768
SF                125.7577890 MHz
WDW                        EM
SSB                         0
LB                       1.00 Hz
GB                          0
PC                       1.40
```

OMS F2
PROTON.d CDCl3 {C:\Bruker\TOPSPIN} IG 43

Compound 42

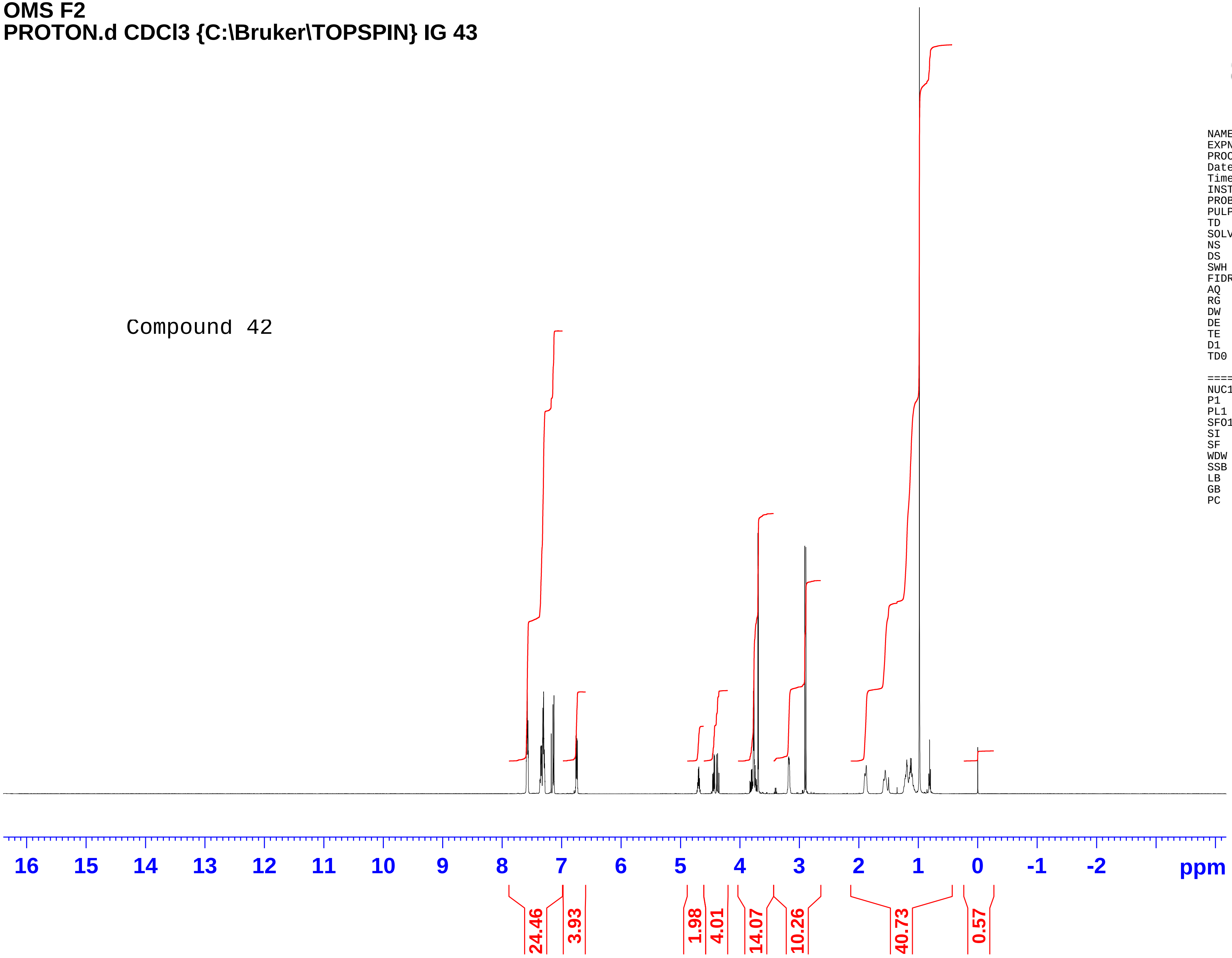

UNIVERSITY OF DUNDEE

NAME	IG-AC-OMSOMBNDIOF2	
EXPNO	1	
PROCNO	1	
Date_	20100430	
Time	14.22	
INSTRUM	spect	
PROBHD	5 mm QNP 1H/13	
PULPROG	zg30	
TD	65536	
SOLVENT	CDCl3	
NS	16	
DS	2	
SWH	10330.578	Hz
FIDRES	0.157632	Hz
AQ	3.1719923	sec
RG	161	
DW	48.400	usec
DE	6.00	usec
TE	300.2	K
D1	1.00000000	sec
TD0	1	

======== CHANNEL f1 ========		
NUC1	1H	
P1	11.20	usec
PL1	-1.00	dB
SFO1	500.1330885	MHz
SI	65536	
SF	500.1300551	MHz
WDW	EM	
SSB	0	
LB	0.30	Hz
GB	0	
PC	1.40	

OTBDS N3
C13CPD.d CDCl3 {C:\Bruker\TOPSPIN} IG 15

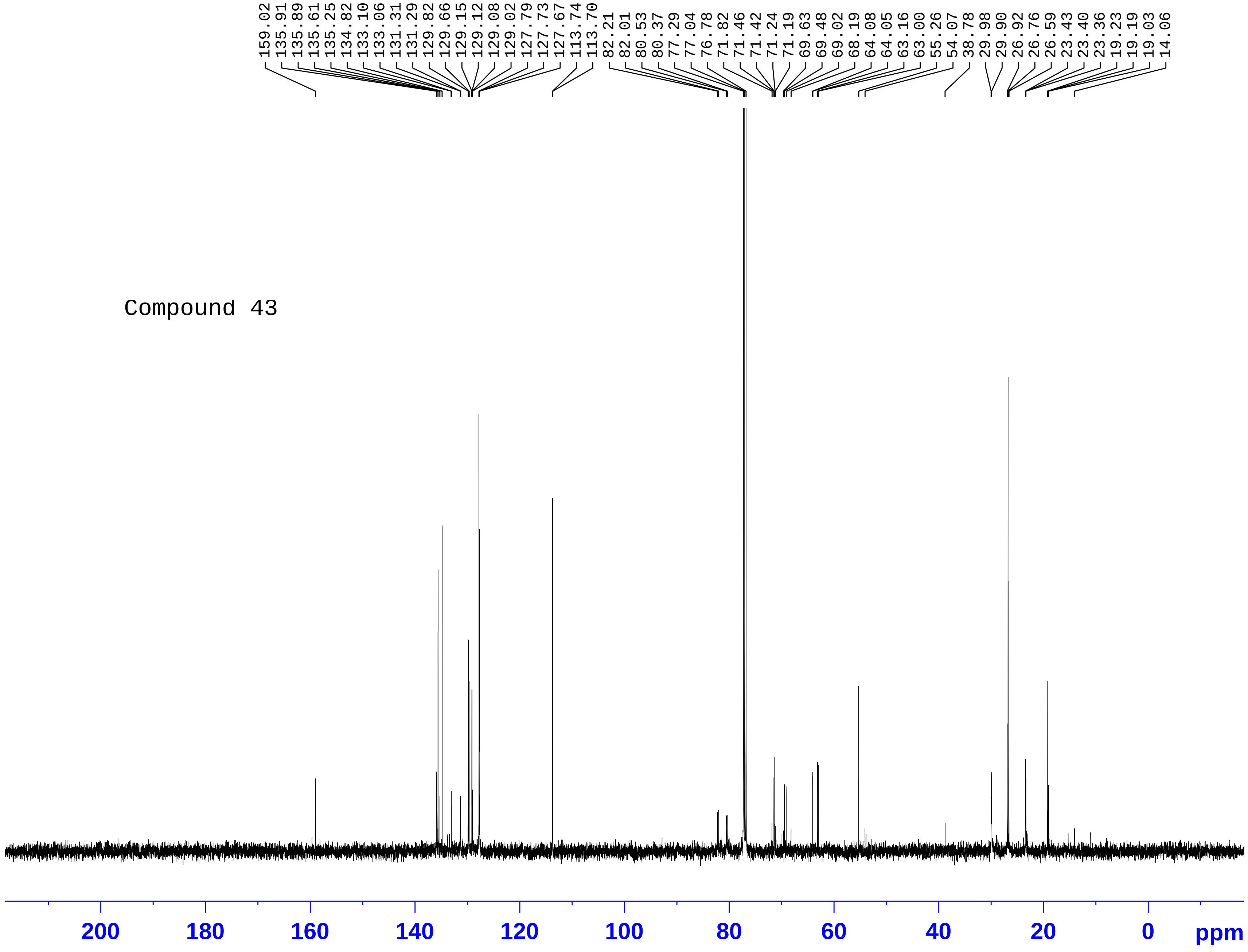


```
NAME      IG-AC-OTBDPS N3 DPPA
EXPNO                  3
PROCNO                 1
Date_           20100315
Time               15.48
INSTRUM            spect
PROBHD    5 mm QNP 1H/13
PULPROG          zgpg30
TD                 65536
SOLVENT            CDCl3
NS                   256
DS                     4
SWH            29761.904 Hz
FIDRES          0.454131 Hz
AQ             1.1010548 sec
RG                  2050
DW                16.800 usec
DE                  6.00 usec
TE                 300.2 K
D1            2.00000000 sec
d11           0.03000000 sec
DELTA         1.89999998 sec
TD0                    1

======== CHANNEL f1 ========
NUC1                 13C
P1                  7.80 usec
PL1                 0.00 dB
SFO1         125.7703643 MHz

======== CHANNEL f2 ========
CPDPRG2          waltz16
NUC2                  1H
PCPD2              80.00 usec
PL2                -1.00 dB
PL12               16.00 dB
PL13               16.00 dB
SFO2         500.1320005 MHz
SI                 32768
SF           125.7577890 MHz
WDW                   EM
SSB                    0
LB                  1.00 Hz
GB                     0
PC                  1.40
```

OTBDS N3
PROTON.d CDCl3 {C:\Bruker\TOPSPIN} IG 15

Compound 43

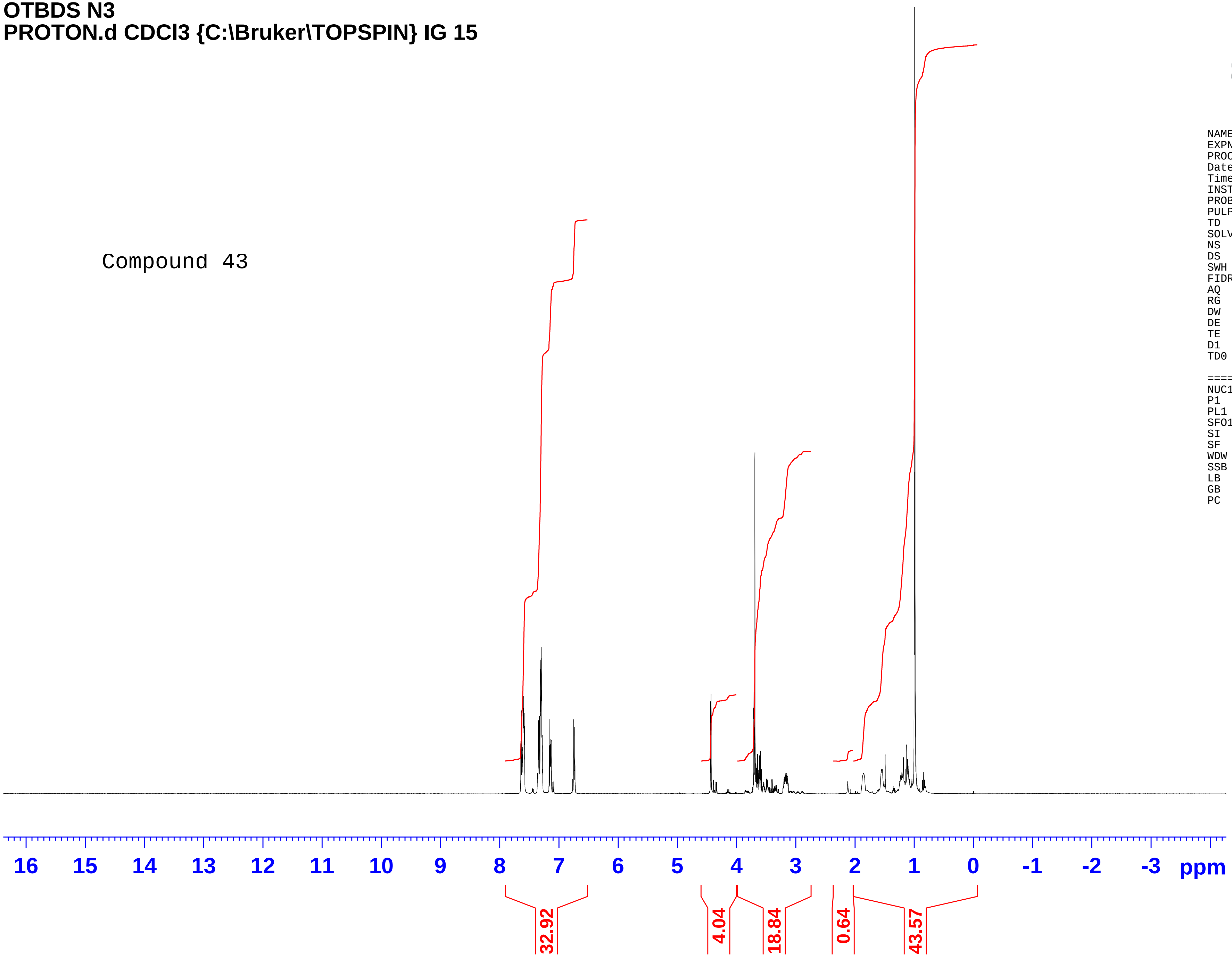

UNIVERSITY OF DUNDEE

NAME	IG-AC-OTBDPS N3 DPPA
EXPNO	1
PROCNO	1
Date_	20100315
Time	15.23
INSTRUM	spect
PROBHD	5 mm QNP 1H/13
PULPROG	zg30
TD	65536
SOLVENT	CDCl3
NS	16
DS	2
SWH	10330.578 Hz
FIDRES	0.157632 Hz
AQ	3.1719923 sec
RG	90.5
DW	48.400 usec
DE	6.00 usec
TE	300.2 K
D1	1.00000000 sec
TD0	1

======== CHANNEL f1 ========

NUC1	1H
P1	11.20 usec
PL1	-1.00 dB
SFO1	500.1330885 MHz
SI	65536
SF	500.1300592 MHz
WDW	EM
SSB	0
LB	0.30 Hz
GB	0
PC	1.40

N3 OMBNDEPRO
C13CPD6.d CDCl3 {C:\Bruker\TOPSPIN} IG 13

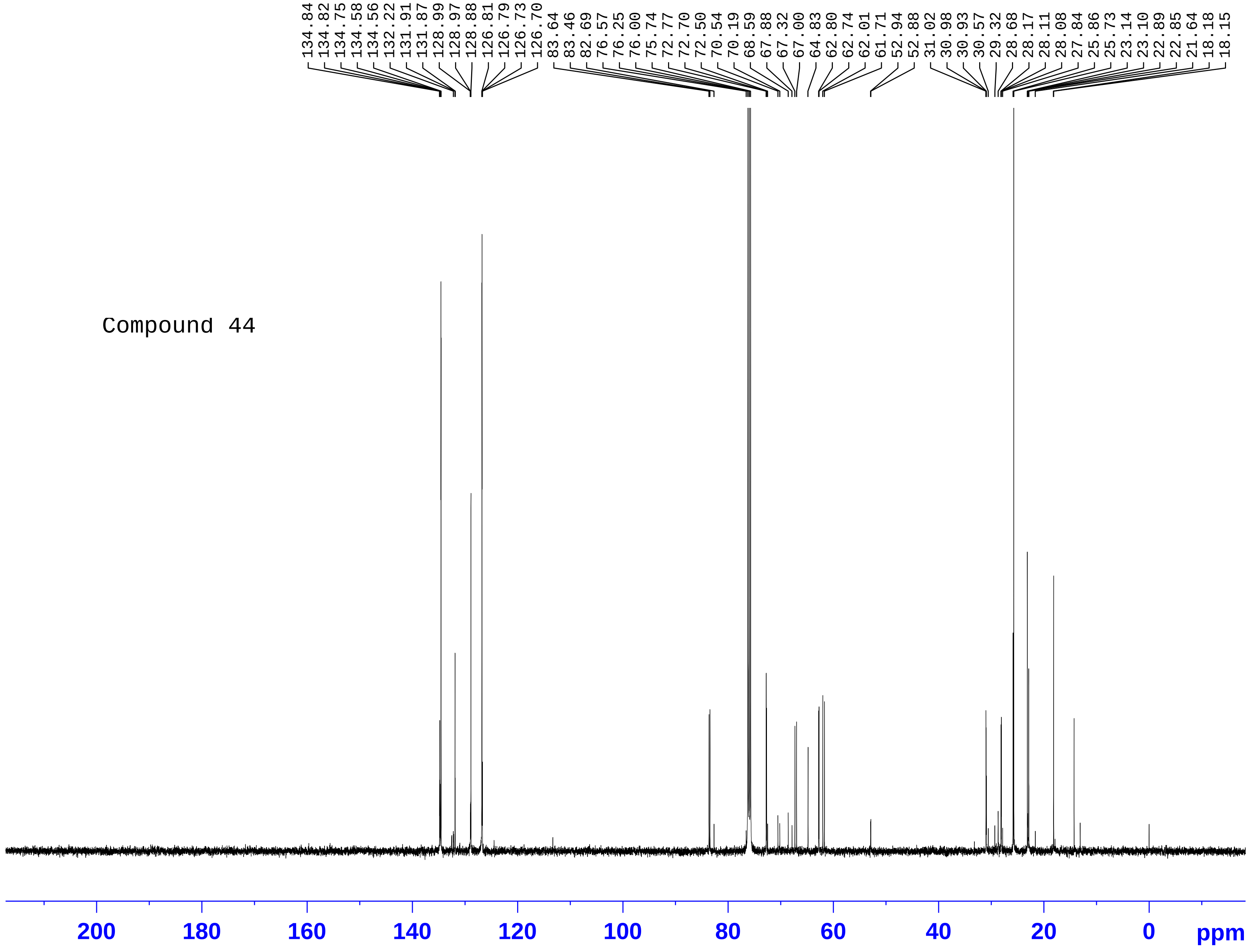

UNIVERSITY OF DUNDEE

```
NAME       IG-AC-N3 TFAOMBNDEPR
EXPNO                     3
PROCNO                    1
Date_              20100324
Time                   1.13
INSTRUM               spect
PROBHD     5 mm QNP 1H/13
PULPROG             zgpg30
TD                    65536
SOLVENT               CDCl3
NS                     6144
DS                        4
SWH              29761.904 Hz
FIDRES            0.454131 Hz
AQ               1.1010548 sec
RG                     2050
DW                   16.800 usec
DE                     6.00 usec
TE                    300.2 K
D1               2.00000000 sec
d11              0.03000000 sec
DELTA            1.89999998 sec
TD0                       1

======== CHANNEL f1 ========
NUC1                    13C
P1                     7.80 usec
PL1                    0.00 dB
SFO1            125.7703643 MHz

======== CHANNEL f2 ========
CPDPRG2             waltz16
NUC2                     1H
PCPD2                 80.00 usec
PL2                   -1.00 dB
PL12                  16.00 dB
PL13                  16.00 dB
SFO2            500.1320005 MHz
SI                    32768
SF              125.7579170 MHz
WDW                      EM
SSB                       0
LB                     1.00 Hz
GB                        0
PC                     1.40
```

AC423B FR 16-18
C13CPDfast.d CDCl3 {C:\Bruker\TOPSPIN} AC 30

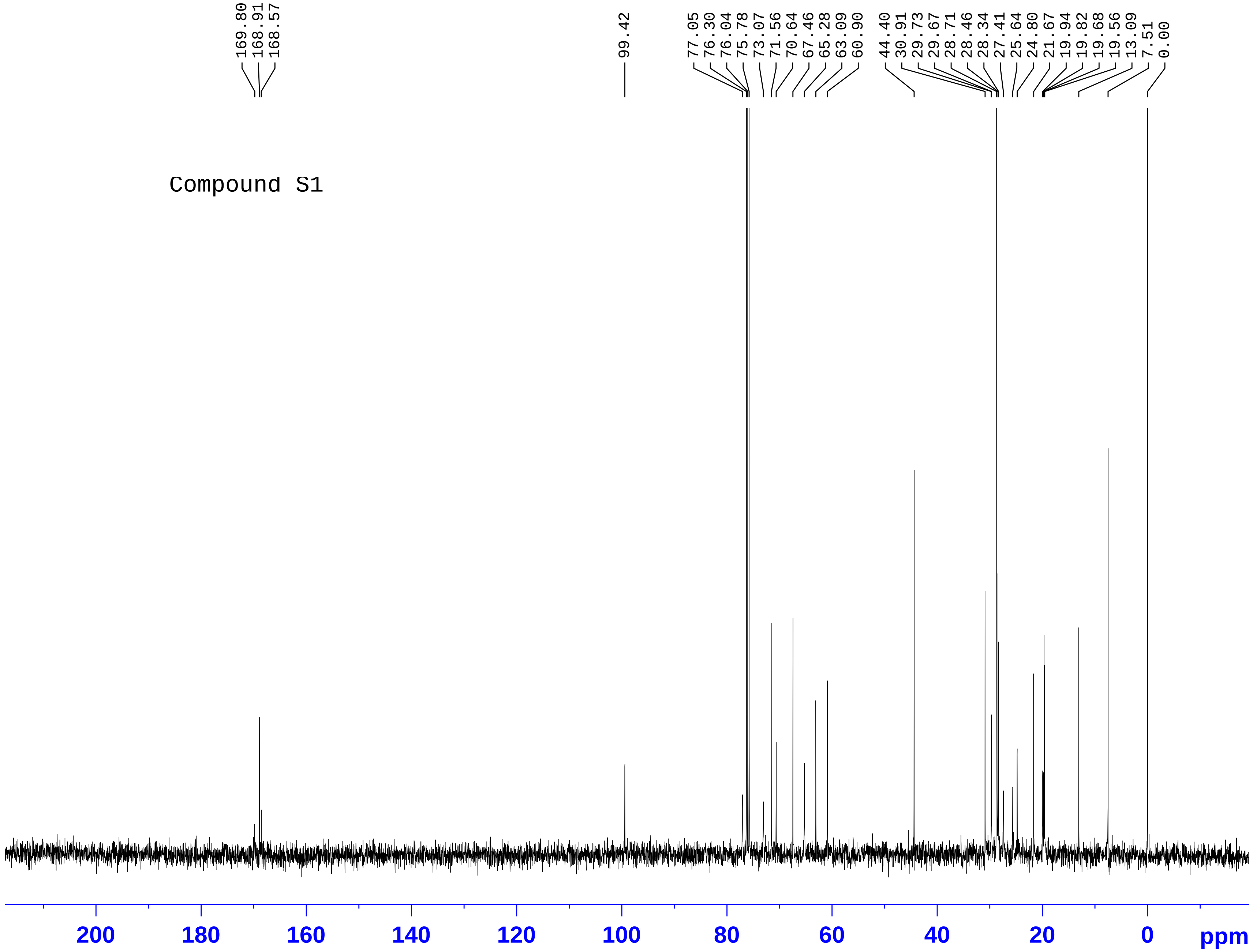

NAME	AC-AC423B
EXPNO	3
PROCNO	1
Date_	20130702
Time	12.50
INSTRUM	spect
PROBHD	5 mm QNP 1H/13
PULPROG	zgpg30
TD	16384
SOLVENT	CDCl3
NS	800
DS	4
SWH	29761.904 Hz
FIDRES	1.816522 Hz
AQ	0.2753012 sec
RG	2050
DW	16.800 usec
DE	6.00 usec
TE	300.2 K
D1	0.30000001 sec
d11	0.03000000 sec
DELTA	0.20000002 sec
TD0	1

======== CHANNEL f1 ========

NUC1	13C
P1	8.30 usec
PL1	0.00 dB
SFO1	125.7703643 MHz

======== CHANNEL f2 ========

CPDPRG2	waltz16
NUC2	1H
PCPD2	80.00 usec
PL2	-0.80 dB
PL12	16.28 dB
PL13	17.28 dB
SFO2	500.1320005 MHz
SI	8192
SF	125.7579139 MHz
WDW	EM
SSB	0
LB	1.00 Hz
GB	0
PC	1.40

N3 OMBNDEPRO
PROTON.d CDCl3 {C:\Bruker\TOPSPIN} IG 13

Compound 44

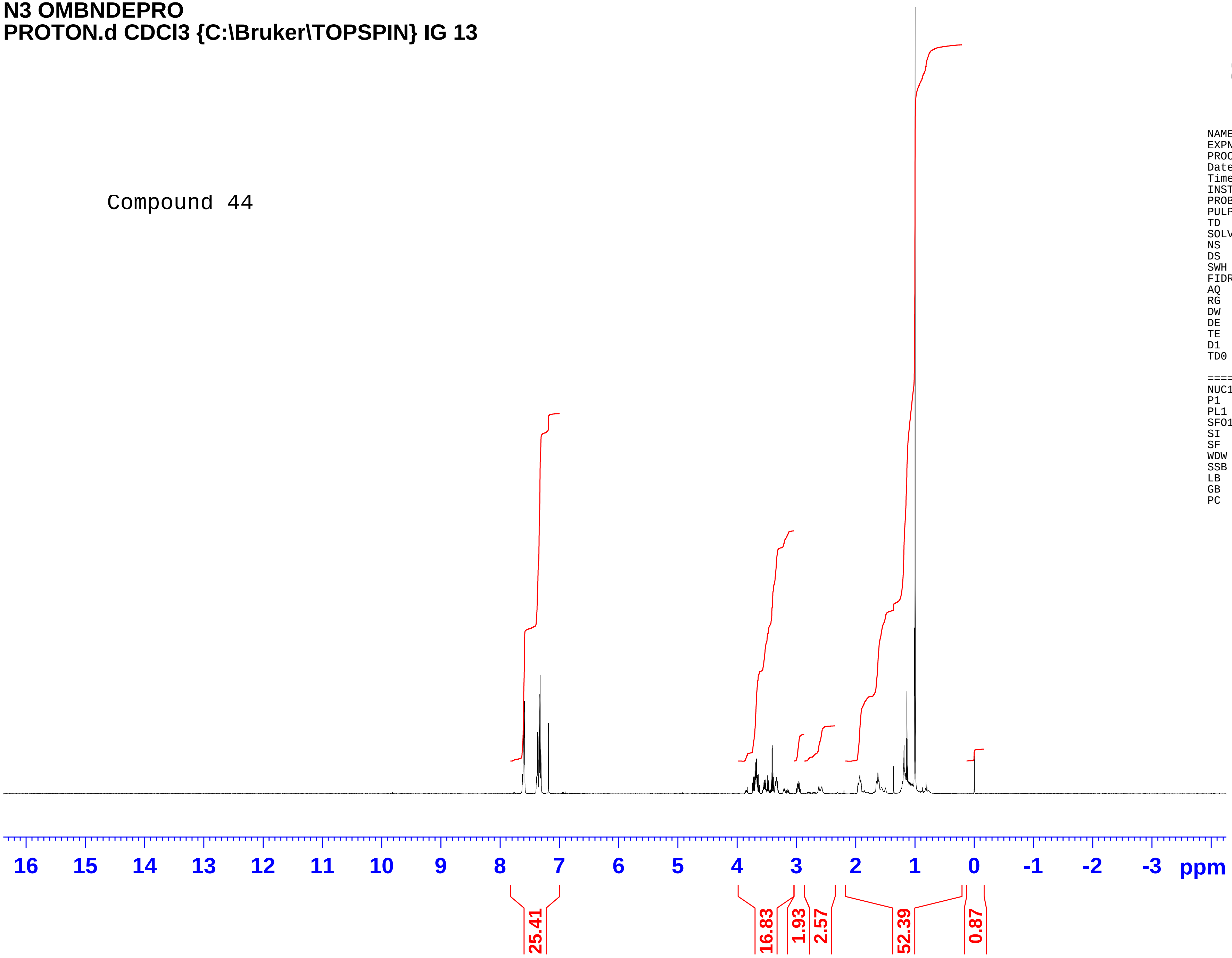

UNIVERSITY OF DUNDEE

NAME	IG-AC-N3 TFAOMBNDEPR	
EXPNO	1	
PROCNO	1	
Date_	20100323	
Time	16.44	
INSTRUM	spect	
PROBHD	5 mm QNP 1H/13	
PULPROG	zg30	
TD	65536	
SOLVENT	CDCl3	
NS	16	
DS	2	
SWH	10330.578	Hz
FIDRES	0.157632	Hz
AQ	3.1719923	sec
RG	256	
DW	48.400	usec
DE	6.00	usec
TE	300.2	K
D1	1.00000000	sec
TD0	1	

======== CHANNEL f1 ========

NUC1	1H	
P1	11.20	usec
PL1	-1.00	dB
SFO1	500.1330885	MHz
SI	65536	
SF	500.1300512	MHz
WDW	EM	
SSB	0	
LB	0.30	Hz
GB	0	
PC	1.40	

OTBDPS N3 PHOSPHATE
C13CPD.d CDCl3 {C:\Bruker\TOPSPIN} IG 2

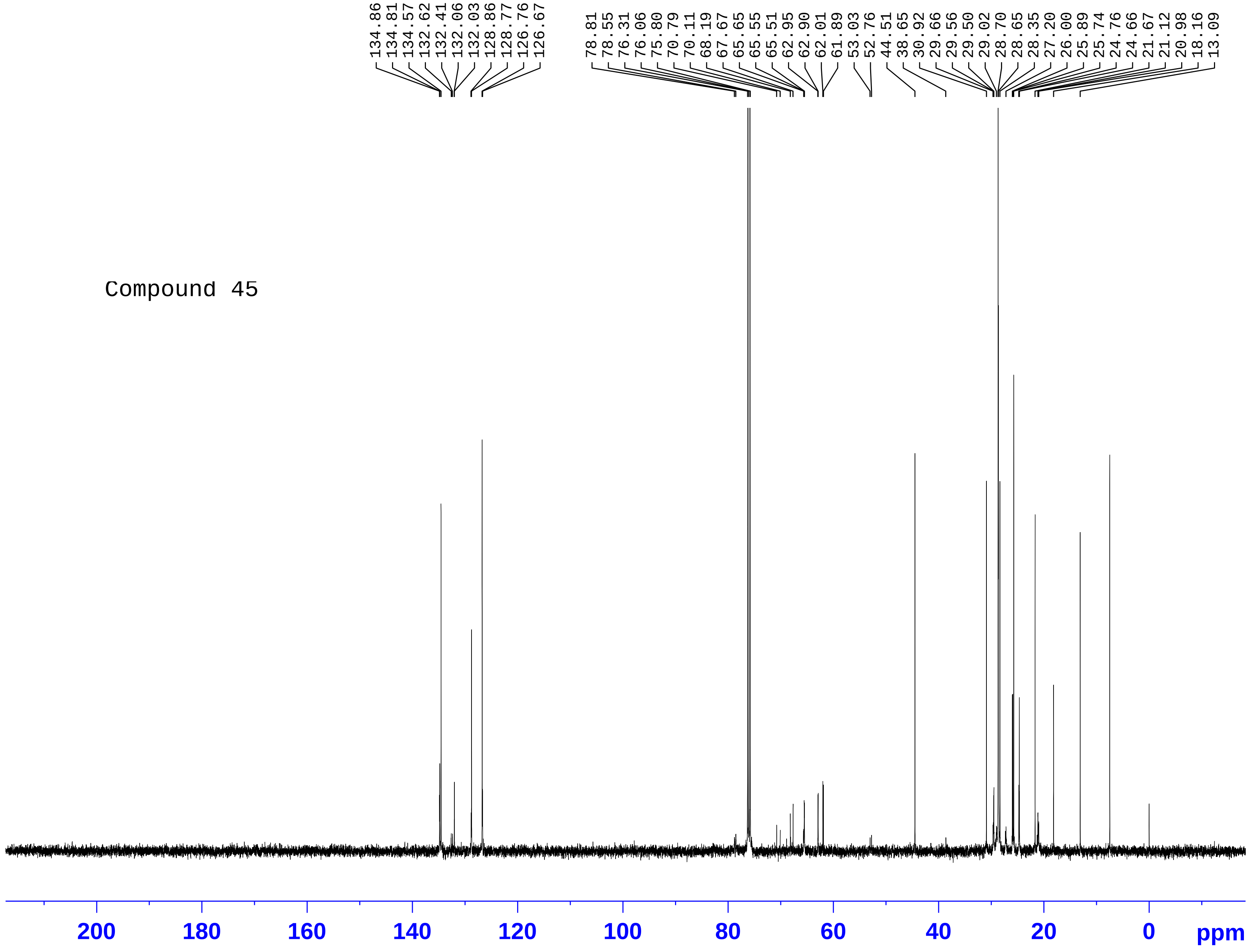

UNIVERSITY OF DUNDEE

NAME	IG-AC-OTBDPSN3 PHOSPHATE	
EXPNO	4	
PROCNO	1	
Date_	20100402	
Time	10.51	
INSTRUM	spect	
PROBHD	5 mm QNP 1H/13	
PULPROG	zgpg30	
TD	65536	
SOLVENT	CDCl3	
NS	256	
DS	4	
SWH	29761.904	Hz
FIDRES	0.454131	Hz
AQ	1.1010548	sec
RG	2050	
DW	16.800	usec
DE	6.00	usec
TE	300.2	K
D1	2.00000000	sec
d11	0.03000000	sec
DELTA	1.89999998	sec
TD0	1	

======== CHANNEL f1 ========

NUC1	13C	
P1	7.80	usec
PL1	0.00	dB
SFO1	125.7703643	MHz

======== CHANNEL f2 ========

CPDPRG2	waltz16	
NUC2	1H	
PCPD2	80.00	usec
PL2	-1.00	dB
PL12	16.00	dB
PL13	16.00	dB
SFO2	500.1320005	MHz
SI	32768	
SF	125.7579157	MHz
WDW	EM	
SSB	0	
LB	1.00	Hz
GB	0	
PC	1.40	

AC423B FR 16-18
PROTON.d CDCl3 {C:\Bruker\TOPSPIN} AC 30

Compound S1

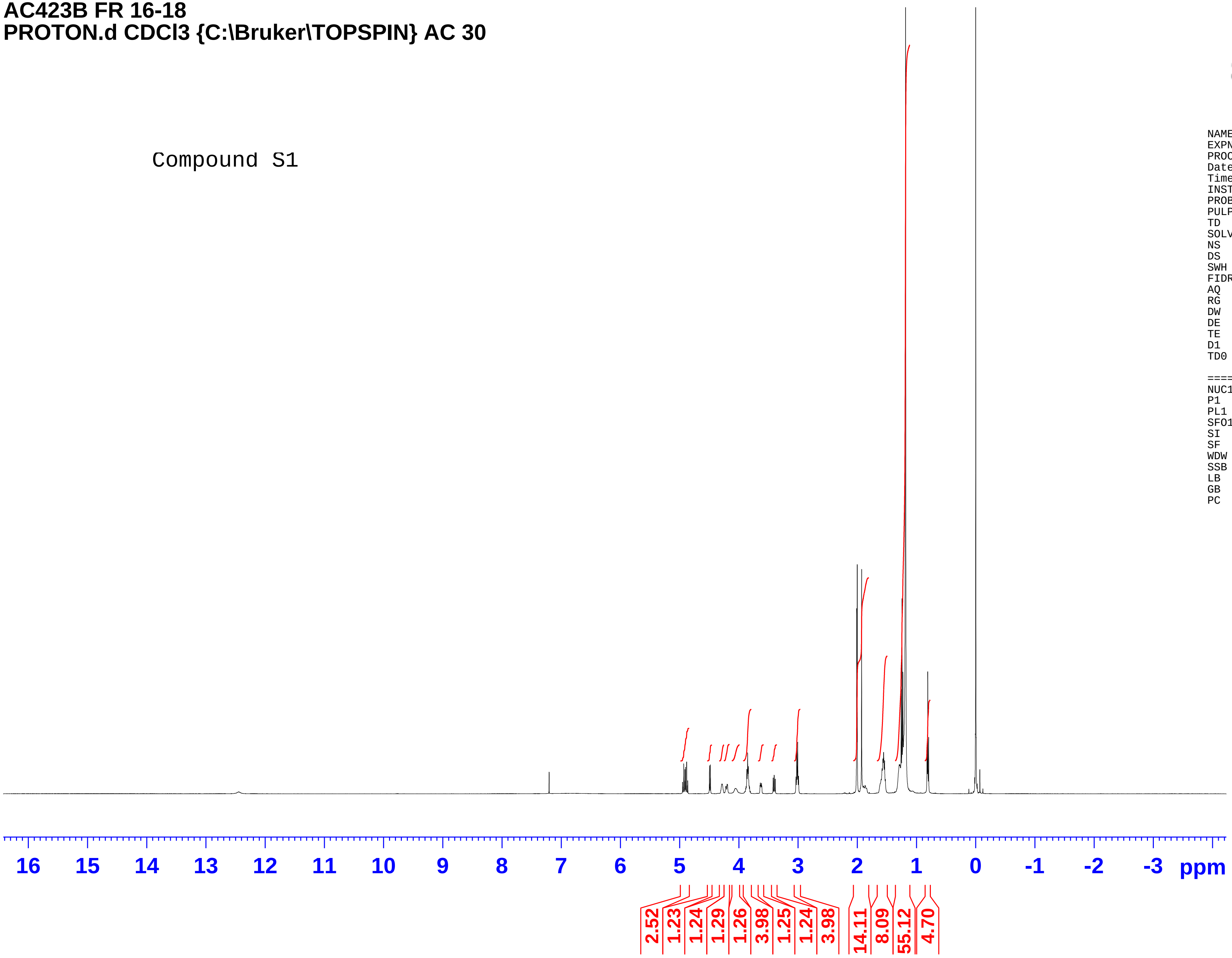

UNIVERSITY OF DUNDEE

NAME	AC-AC423B
EXPNO	1
PROCNO	1
Date_	20130702
Time	12.30
INSTRUM	spect
PROBHD	5 mm QNP 1H/13
PULPROG	zg30
TD	65536
SOLVENT	CDCl3
NS	16
DS	2
SWH	10330.578 Hz
FIDRES	0.157632 Hz
AQ	3.1719923 sec
RG	64
DW	48.400 usec
DE	6.00 usec
TE	300.2 K
D1	1.00000000 sec
TD0	1

======== CHANNEL f1 ========

NUC1	1H
P1	11.20 usec
PL1	-0.60 dB
SFO1	500.1330885 MHz
SI	65536
SF	500.1300401 MHz
WDW	EM
SSB	0
LB	0.30 Hz
GB	0
PC	1.40

OTBDPS N3 PHOSPHATE
PROTON.d CDCl3 {C:\Bruker\TOPSPIN} IG 2

Compound 45

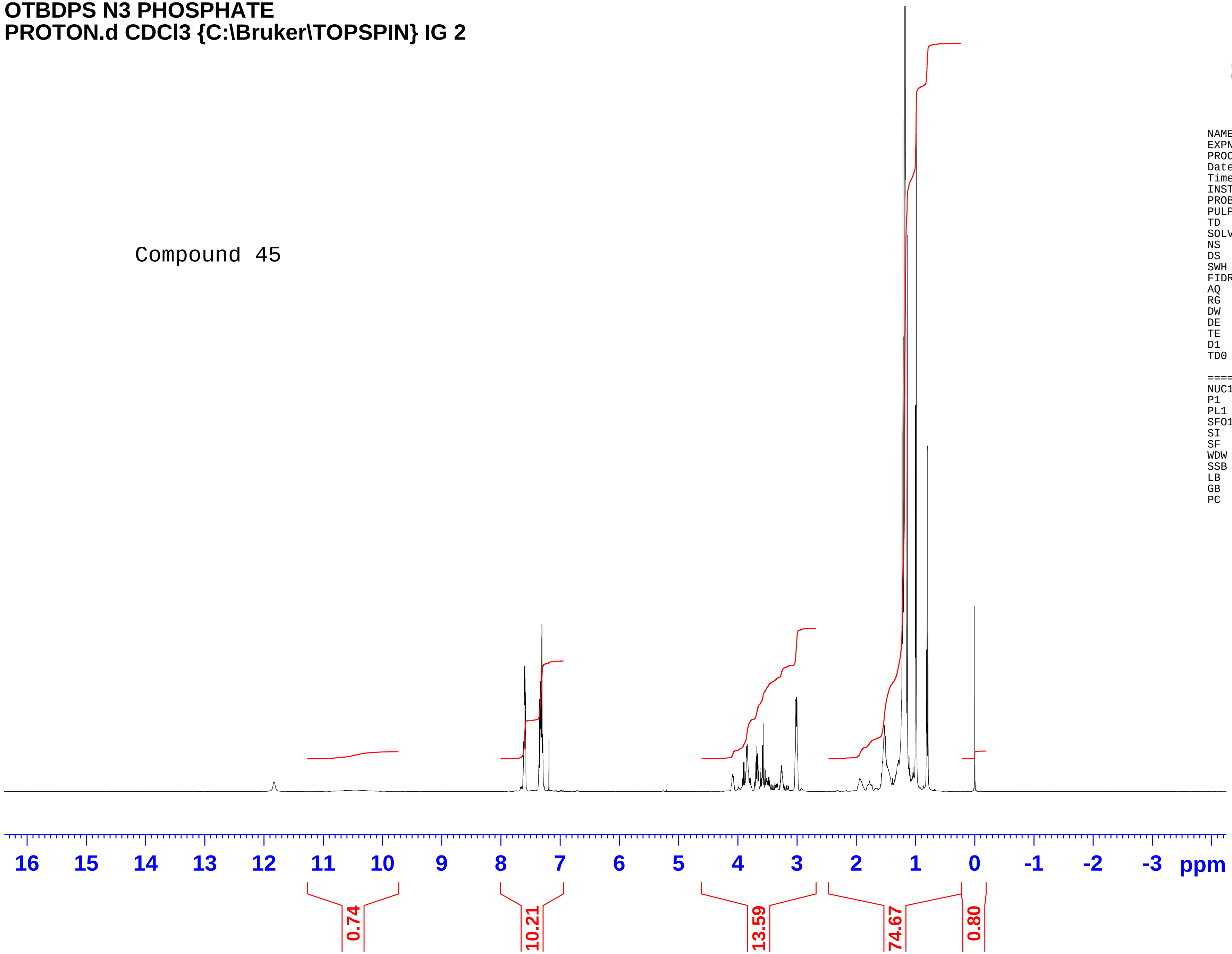

UNIVERSITY OF DUNDEE

NAME	IG-AC-OTBDPSN3 PHOSPHATE	
EXPNO	1	
PROCNO	1	
Date_	20100402	
Time	10.24	
INSTRUM	spect	
PROBHD	5 mm QNP 1H/13	
PULPROG	zg30	
TD	65536	
SOLVENT	CDCl3	
NS	16	
DS	2	
SWH	10330.578	Hz
FIDRES	0.157632	Hz
AQ	3.1719923	sec
RG	40.3	
DW	48.400	usec
DE	6.00	usec
TE	300.2	K
D1	1.00000000	sec
TD0	1	

======== CHANNEL f1 ========		
NUC1	1H	
P1	11.20	usec
PL1	-1.00	dB
SFO1	500.1330885	MHz
SI	65536	
SF	500.1300477	MHz
WDW	EM	
SSB	0	
LB	0.30	Hz
GB	0	
PC	1.40	

OH N3 CYCHEXDIOLPHOS
C13CPD.d CDCl3 {C:\Bruker\TOPSPIN} IG 4

Compound 46

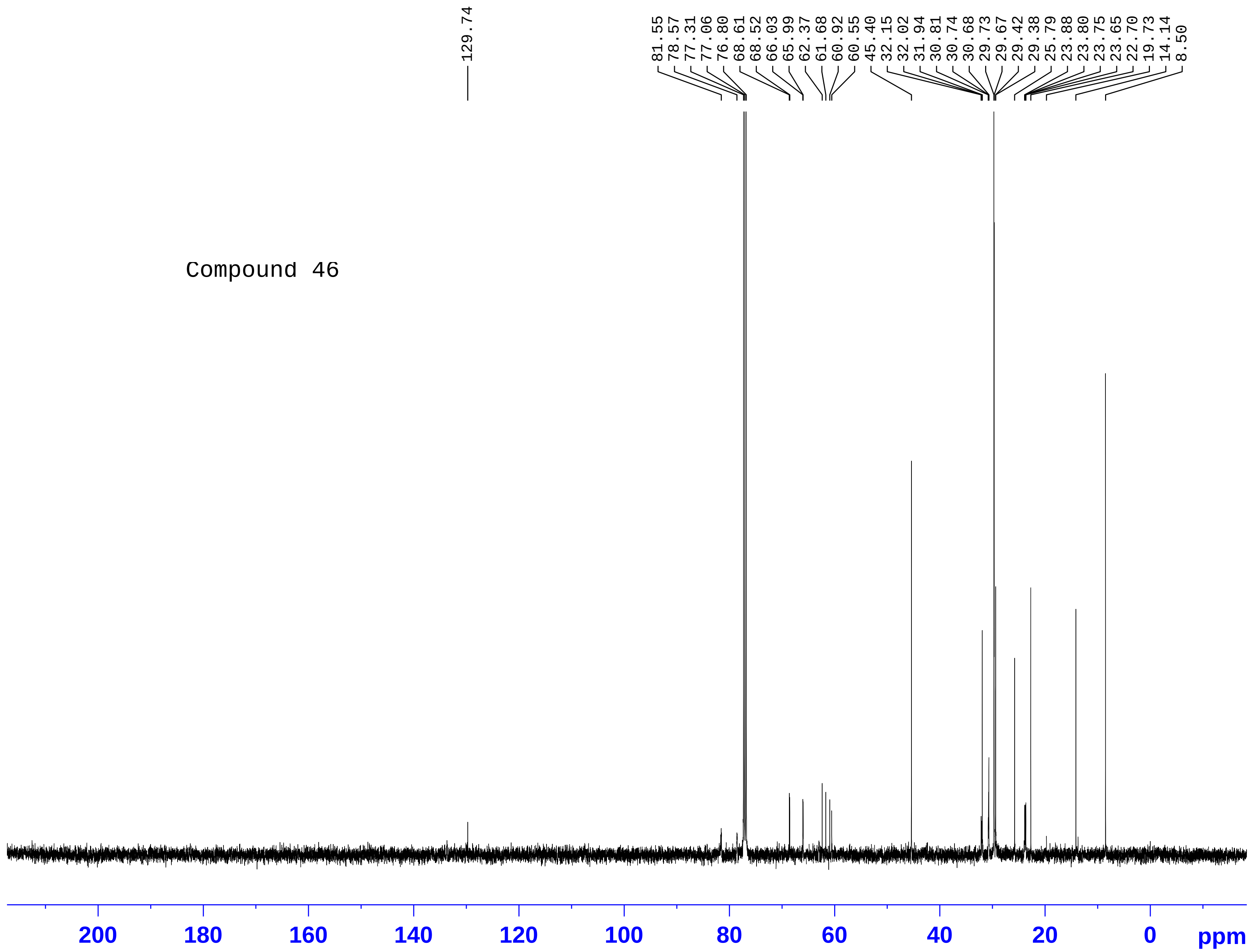

DUNDEE

NAME	IG-AC-OHN3CYCHEXDIOLPHOS	
EXPNO	3	
PROCNO	1	
Date_	20100513	
Time	10.52	
INSTRUM	spect	
PROBHD	5 mm QNP 1H/13	
PULPROG	zgpg30	
TD	65536	
SOLVENT	CDCl3	
NS	256	
DS	4	
SWH	29761.904	Hz
FIDRES	0.454131	Hz
AQ	1.1010548	sec
RG	2050	
DW	16.800	usec
DE	6.00	usec
TE	294.3	K
D1	2.00000000	sec
d11	0.03000000	sec
DELTA	1.89999998	sec
TD0	1	
======== CHANNEL f1 ========		
NUC1	13C	
P1	7.80	usec
PL1	0.00	dB
SFO1	125.7703643	MHz
======== CHANNEL f2 ========		
CPDPRG2	waltz16	
NUC2	1H	
PCPD2	80.00	usec
PL2	-1.00	dB
PL12	16.00	dB
PL13	16.00	dB
SFO2	500.1320005	MHz
SI	32768	
SF	125.7577890	MHz
WDW	EM	
SSB	0	
LB	1.00	Hz
GB	0	
PC	1.40	

AC426
C13CPDfast.d CDCl3 {C:\Bruker\TOPSPIN} AC 18

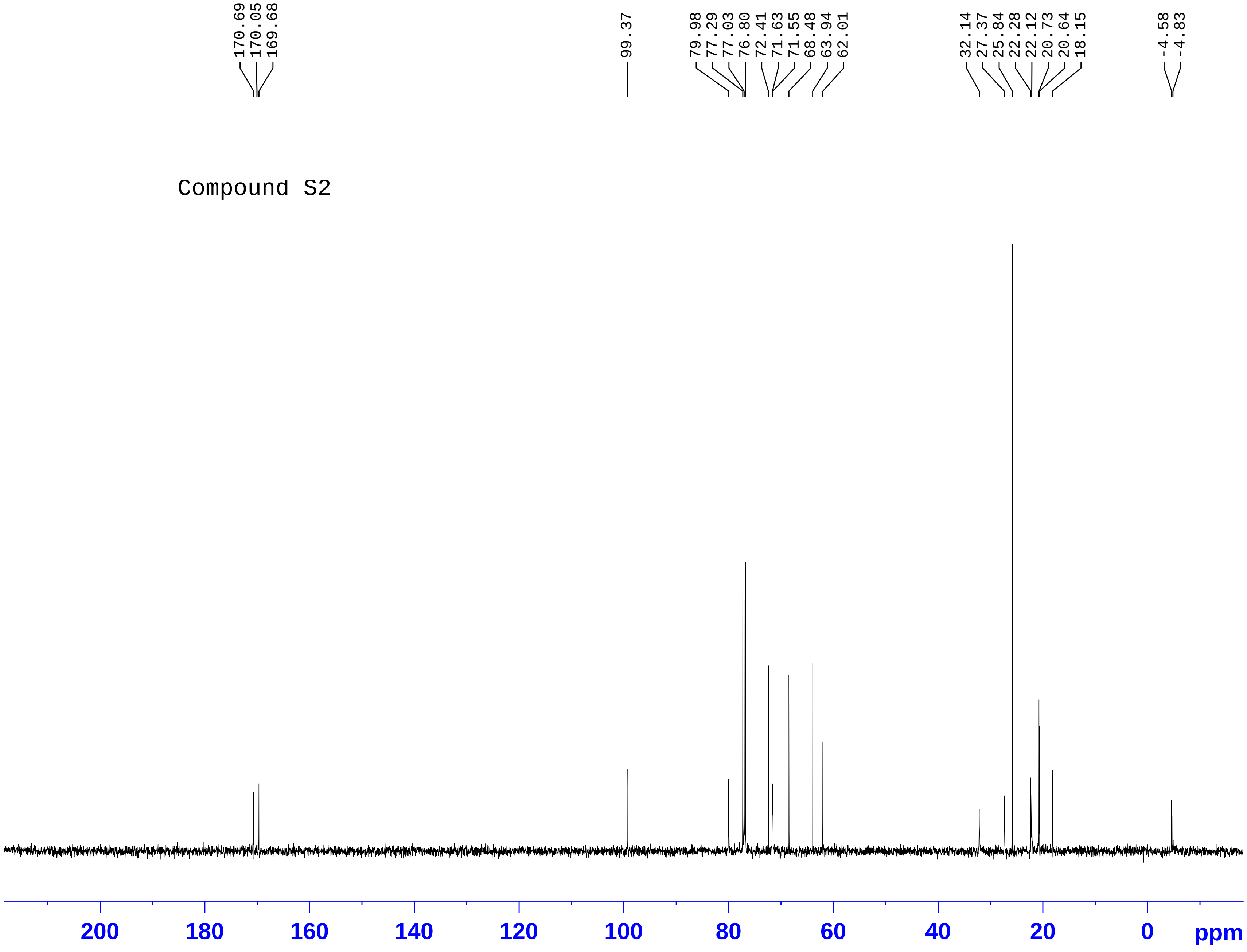

NAME	AC-AC426
EXPNO	4
PROCNO	1
Date_	20110526
Time	10.38
INSTRUM	spect
PROBHD	5 mm QNP 1H/13
PULPROG	zgpg30
TD	16384
SOLVENT	CDCl3
NS	800
DS	4
SWH	29761.904 Hz
FIDRES	1.816522 Hz
AQ	0.2753012 sec
RG	2050
DW	16.800 usec
DE	6.00 usec
TE	294.0 K
D1	0.30000001 sec
d11	0.03000000 sec
DELTA	0.20000002 sec
TD0	1

======== CHANNEL f1 ========

NUC1	13C
P1	8.18 usec
PL1	0.00 dB
SFO1	125.7703643 MHz

======== CHANNEL f2 ========

CPDPRG2	waltz16
NUC2	1H
PCPD2	80.00 usec
PL2	-1.00 dB
PL12	16.00 dB
PL13	16.00 dB
SFO2	500.1320005 MHz
SI	8192
SF	125.7577890 MHz
WDW	EM
SSB	0
LB	1.00 Hz
GB	0
PC	1.40

OH N3 CYCHEXDIOLPHOS
PROTON.d CDCl3 {C:\Bruker\TOPSPIN} IG 4

Compound 46

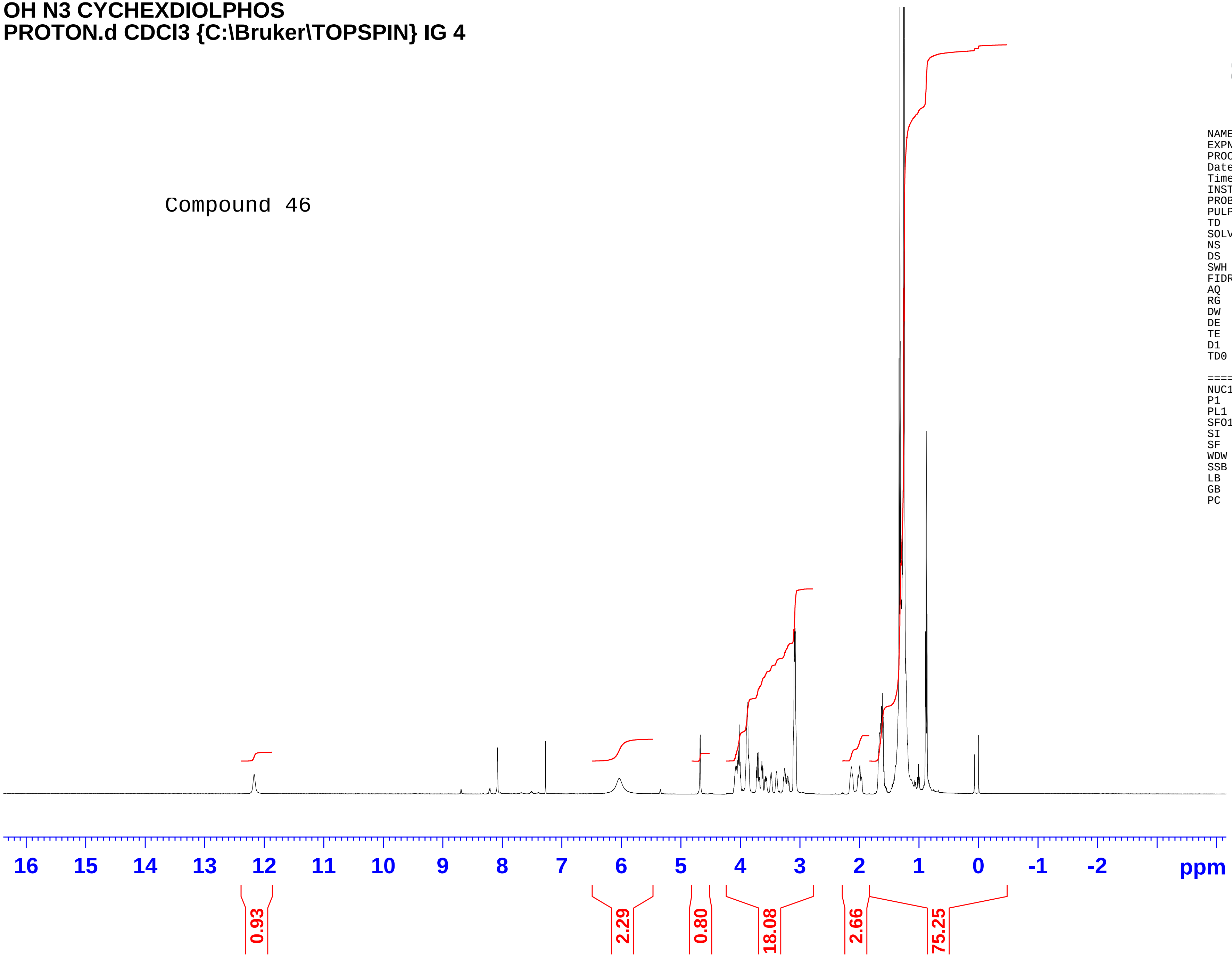

UNIVERSITY OF DUNDEE

NAME	IG-AC-OHN3CYCHEXDIOLPHOS
EXPNO	1
PROCNO	1
Date_	20100513
Time	10.27
INSTRUM	spect
PROBHD	5 mm QNP 1H/13
PULPROG	zg30
TD	65536
SOLVENT	CDCl3
NS	16
DS	2
SWH	10330.578 Hz
FIDRES	0.157632 Hz
AQ	3.1719923 sec
RG	71.8
DW	48.400 usec
DE	6.00 usec
TE	293.2 K
D1	1.00000000 sec
TD0	1

======== CHANNEL f1 ========

NUC1	1H
P1	11.20 usec
PL1	-1.00 dB
SFO1	500.1330885 MHz
SI	65536
SF	500.1300056 MHz
WDW	EM
SSB	0
LB	0.30 Hz
GB	0
PC	1.40

AC426
PROTON.d CDCl3 {C:\Bruker\TOPSPIN} AC 16

Compound S2

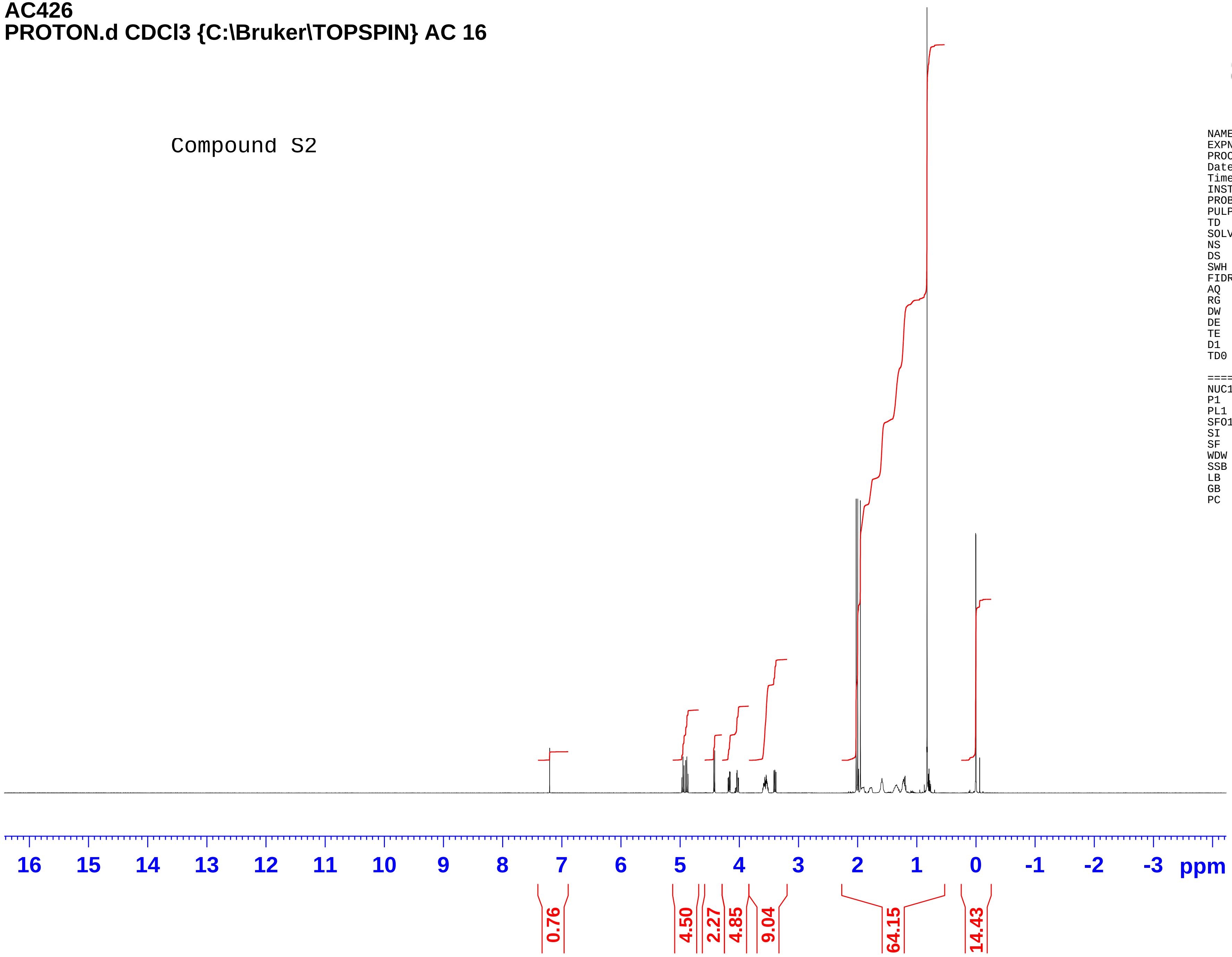

UNIVERSITY OF DUNDEE

NAME	AC-AC426
EXPNO	1
PROCNO	1
Date_	20110526
Time	10.04
INSTRUM	spect
PROBHD	5 mm QNP 1H/13
PULPROG	zg30
TD	65536
SOLVENT	CDCl3
NS	16
DS	2
SWH	10330.578 Hz
FIDRES	0.157632 Hz
AQ	3.1719923 sec
RG	161
DW	48.400 usec
DE	6.00 usec
TE	293.0 K
D1	1.00000000 sec
TD0	1

======== CHANNEL f1 ========

NUC1	1H
P1	11.20 usec
PL1	-1.00 dB
SFO1	500.1330885 MHz
SI	65536
SF	500.1300398 MHz
WDW	EM
SSB	0
LB	0.30 Hz
GB	0
PC	1.40

AC428B
C13CPDfast.d CDCl3 {C:\Bruker\TOPSPIN} AC 2

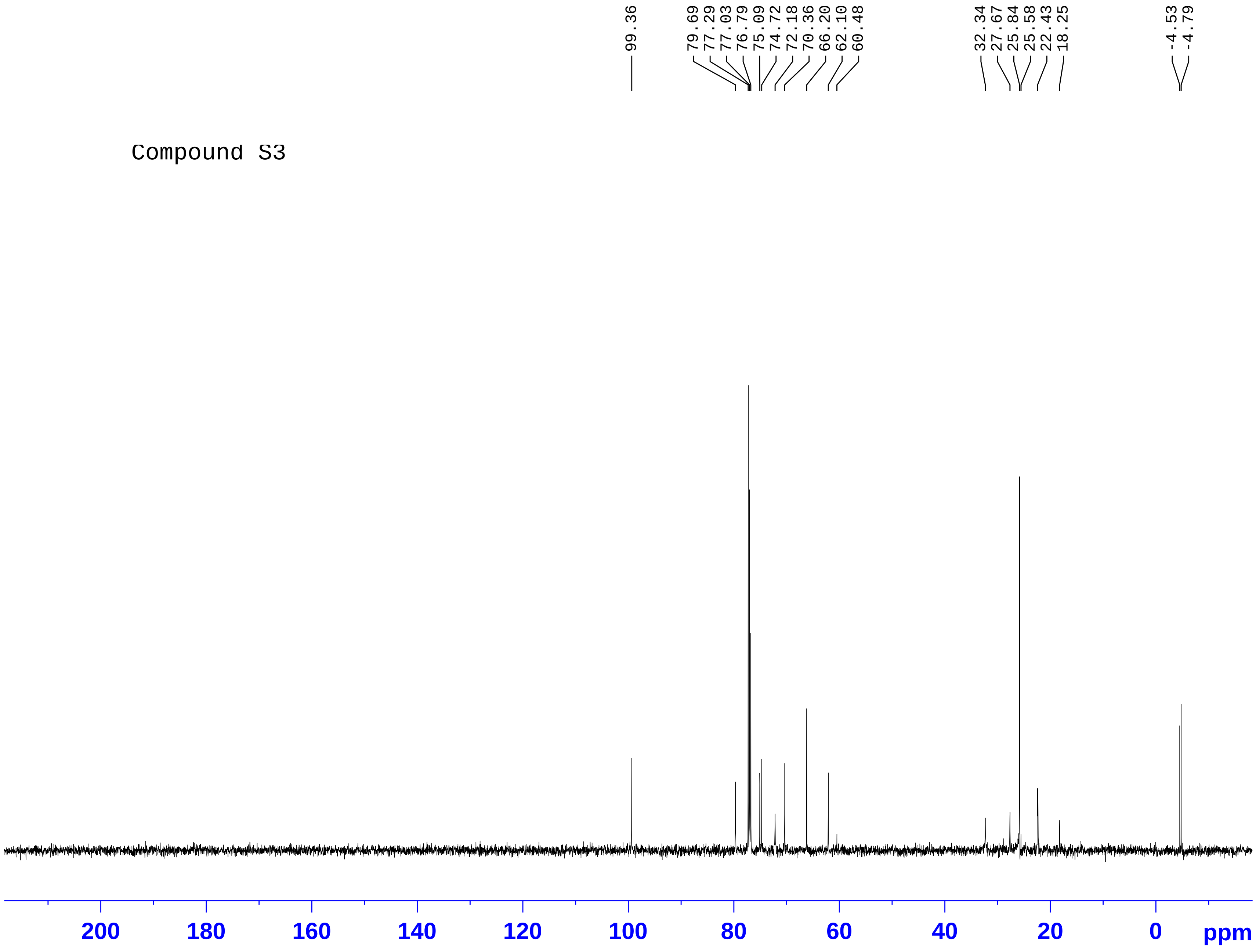

UNIVERSITY OF DUNDEE

NAME	AC-AC428B
EXPNO	2
PROCNO	1
Date_	20110614
Time	10.12
INSTRUM	spect
PROBHD	5 mm QNP 1H/13
PULPROG	zgpg30
TD	16384
SOLVENT	CDCl3
NS	800
DS	4
SWH	29761.904 Hz
FIDRES	1.816522 Hz
AQ	0.2753012 sec
RG	2050
DW	16.800 usec
DE	6.00 usec
TE	294.8 K
D1	0.30000001 sec
d11	0.03000000 sec
DELTA	0.20000002 sec
TD0	1

======== CHANNEL f1 ========

NUC1	13C
P1	8.18 usec
PL1	0.00 dB
SFO1	125.7703643 MHz

======== CHANNEL f2 ========

CPDPRG2	waltz16
NUC2	1H
PCPD2	80.00 usec
PL2	-1.00 dB
PL12	16.00 dB
PL13	16.00 dB
SFO2	500.1320005 MHz
SI	8192
SF	125.7577890 MHz
WDW	EM
SSB	0
LB	1.00 Hz
GB	0
PC	1.40

AC428B
PROTON.d CDCl3 {C:\Bruker\TOPSPIN} AC 1

Compound S3

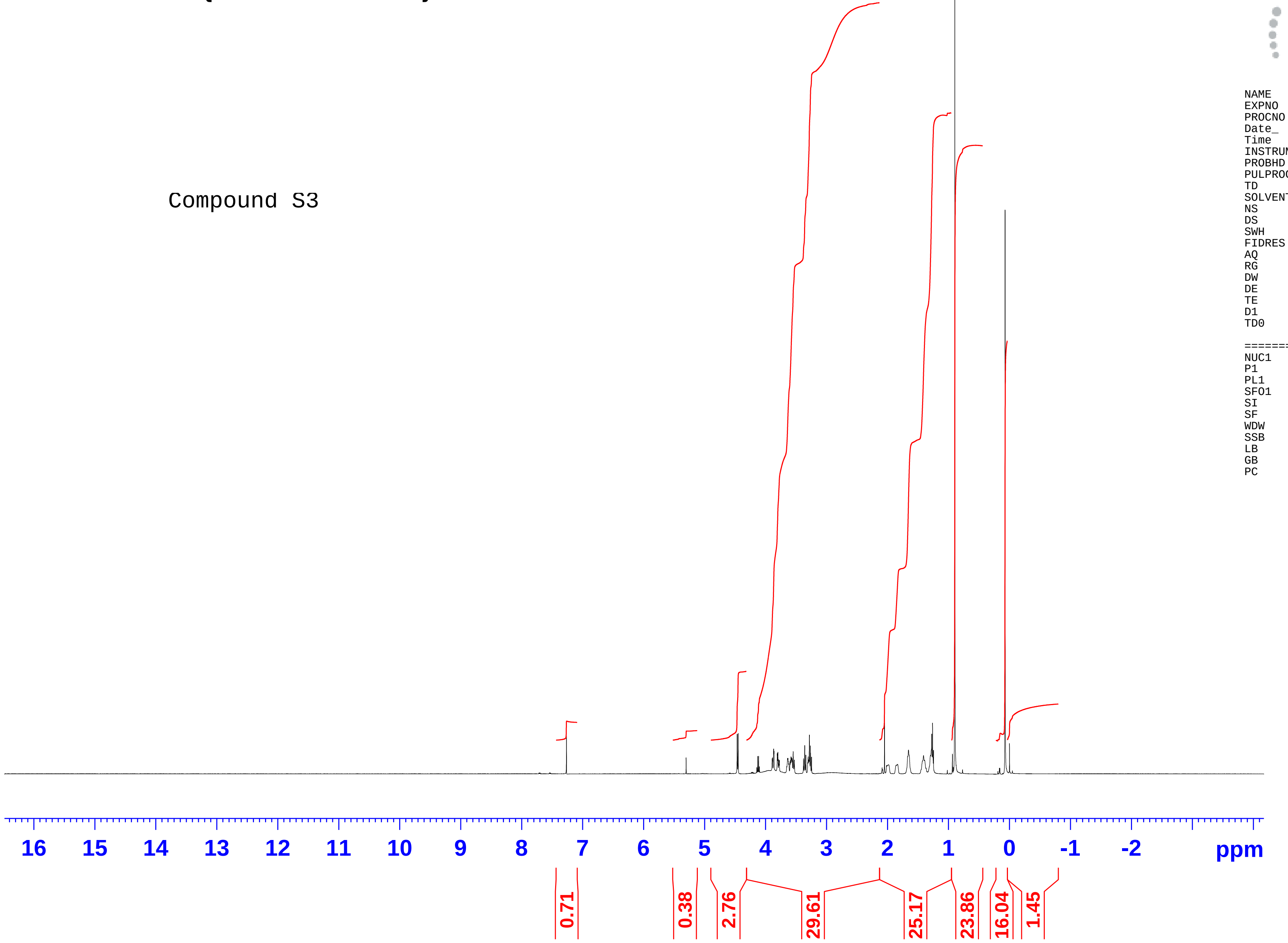

UNIVERSITY OF DUNDEE

NAME	AC-AC428B
EXPNO	1
PROCNO	1
Date_	20110614
Time	9.54
INSTRUM	spect
PROBHD	5 mm QNP 1H/13
PULPROG	zg30
TD	65536
SOLVENT	CDCl3
NS	16
DS	2
SWH	10330.578 Hz
FIDRES	0.157632 Hz
AQ	3.1719923 sec
RG	181
DW	48.400 usec
DE	6.00 usec
TE	293.9 K
D1	1.00000000 sec
TD0	1

======== CHANNEL f1 ========

NUC1	1H
P1	11.20 usec
PL1	-1.00 dB
SFO1	500.1330885 MHz
SI	65536
SF	500.1300102 MHz
WDW	EM
SSB	0
LB	0.30 Hz
GB	0
PC	1.40

AC430
C13CPDfast.d CDCl3 {C:\Bruker\TOPSPIN} AC 2

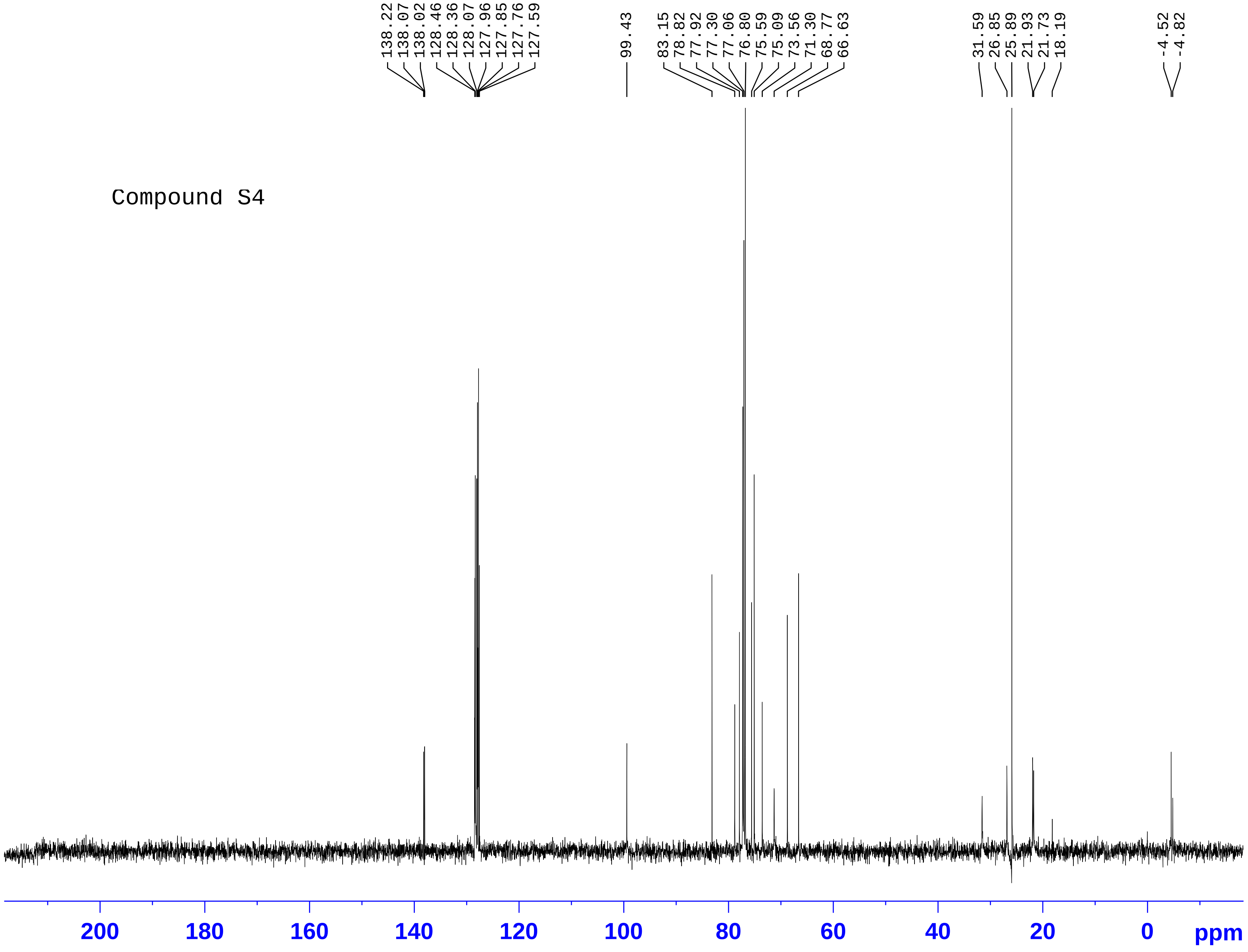

NAME	AC-AC430	
EXPNO	4	
PROCNO	1	
Date_	20110624	
Time	10.34	
INSTRUM	spect	
PROBHD	5 mm QNP 1H/13	
PULPROG	zgpg30	
TD	16384	
SOLVENT	CDCl3	
NS	800	
DS	4	
SWH	29761.904	Hz
FIDRES	1.816522	Hz
AQ	0.2753012	sec
RG	2050	
DW	16.800	usec
DE	6.00	usec
TE	294.7	K
D1	0.30000001	sec
d11	0.03000000	sec
DELTA	0.20000002	sec
TD0	1	

======== CHANNEL f1 ========

NUC1	13C	
P1	8.18	usec
PL1	0.00	dB
SFO1	125.7703643	MHz

======== CHANNEL f2 ========

CPDPRG2	waltz16	
NUC2	1H	
PCPD2	80.00	usec
PL2	-1.00	dB
PL12	16.00	dB
PL13	16.00	dB
SFO2	500.1320005	MHz
SI	8192	
SF	125.7577890	MHz
WDW	EM	
SSB	0	
LB	1.00	Hz
GB	0	
PC	1.40	

AC430
PROTON.d CDCl3 {C:\Bruker\TOPSPIN} AC 2

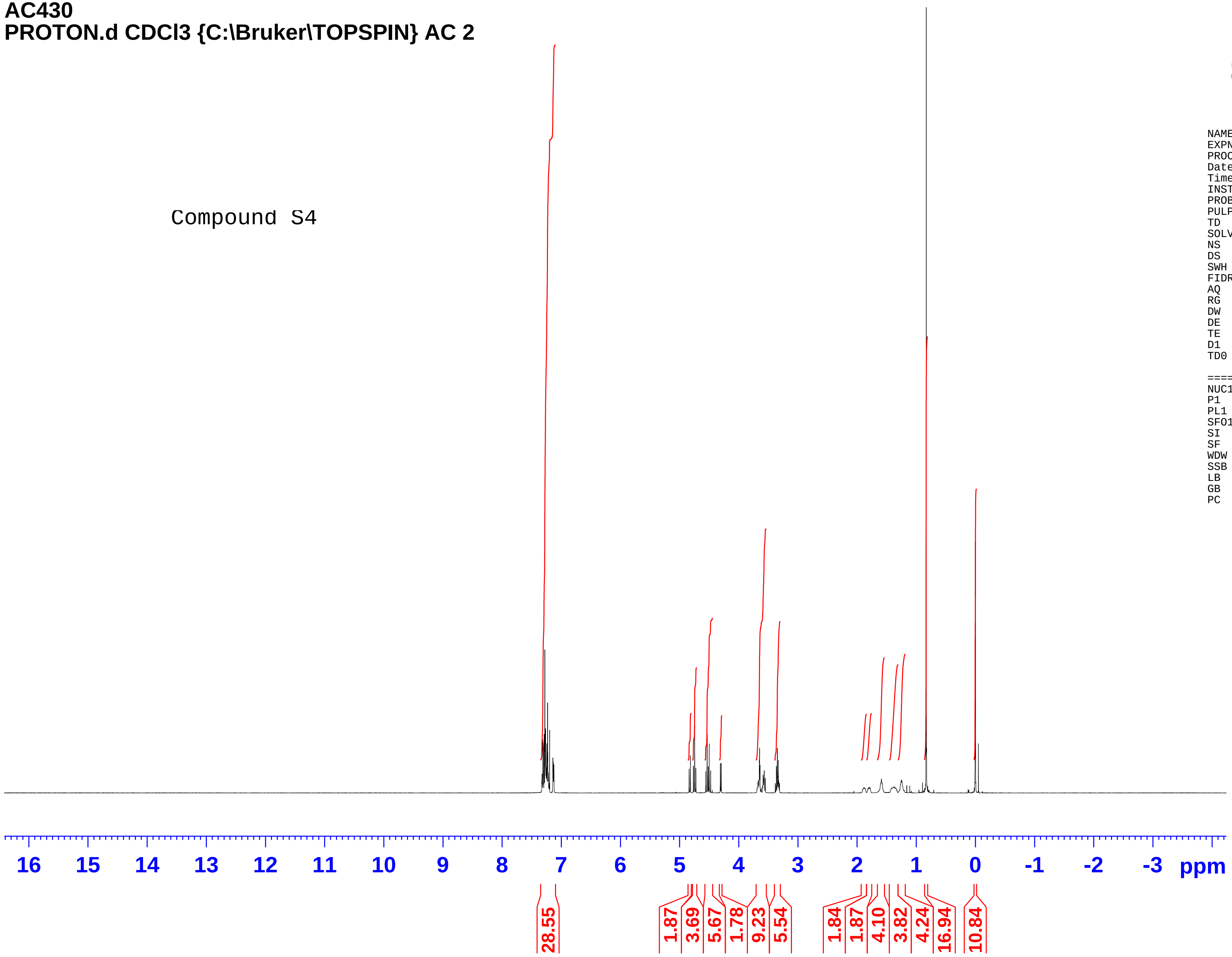

UNIVERSITY OF DUNDEE

NAME	AC-AC430	
EXPNO	2	
PROCNO	1	
Date_	20110624	
Time	10.14	
INSTRUM	spect	
PROBHD	5 mm QNP 1H/13	
PULPROG	zg30	
TD	65536	
SOLVENT	CDCl3	
NS	16	
DS	2	
SWH	10330.578	Hz
FIDRES	0.157632	Hz
AQ	3.1719923	sec
RG	181	
DW	48.400	usec
DE	6.00	usec
TE	293.7	K
D1	1.00000000	sec
TD0	1	

======== CHANNEL f1 ========

NUC1	1H	
P1	11.20	usec
PL1	-1.00	dB
SFO1	500.1330885	MHz
SI	65536	
SF	500.1300443	MHz
WDW	EM	
SSB	0	
LB	0.30	Hz
GB	0	
PC	1.40	

AC432 AMINE
C13CPDfast.d CDCl3 {C:\Bruker\TOPSPIN} AC 10

Compound S5

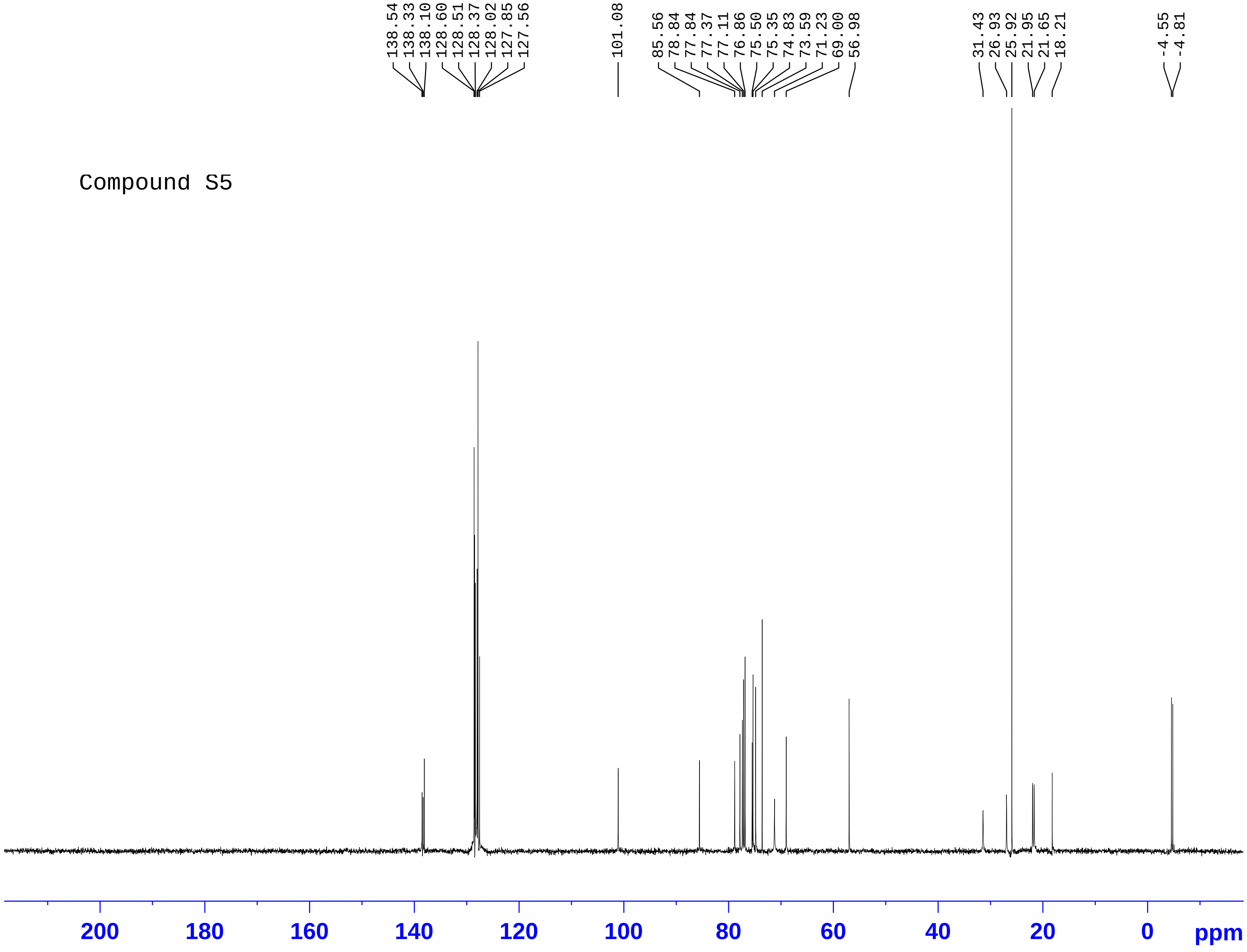

UNIVERSITY OF DUNDEE

```
NAME          AC-AC432NH2
EXPNO                   9
PROCNO                  1
Date_            20111014
Time                11.42
INSTRUM             spect
PROBHD   5 mm QNP 1H/13
PULPROG           zgpg30
TD                  16384
SOLVENT             CDCl3
NS                    800
DS                      4
SWH            29761.904 Hz
FIDRES          1.816522 Hz
AQ             0.2753012 sec
RG                   2050
DW                 16.800 usec
DE                   6.00 usec
TE                  295.4 K
D1            0.30000001 sec
d11           0.03000000 sec
DELTA         0.20000002 sec
TD0                     1

======== CHANNEL f1 ========
NUC1                  13C
P1                   8.18 usec
PL1                  0.00 dB
SFO1          125.7703643 MHz

======== CHANNEL f2 ========
CPDPRG2           waltz16
NUC2                   1H
PCPD2               80.00 usec
PL2                 -1.00 dB
PL12                16.00 dB
PL13                16.00 dB
SFO2          500.1320005 MHz
SI                   8192
SF            125.7577890 MHz
WDW                    EM
SSB                     0
LB                   1.00 Hz
GB                      0
PC                   1.40
```

AC432 AMINE
PROTON.d CDCl3 {C:\Bruker\TOPSPIN} AC 10

Compound S5

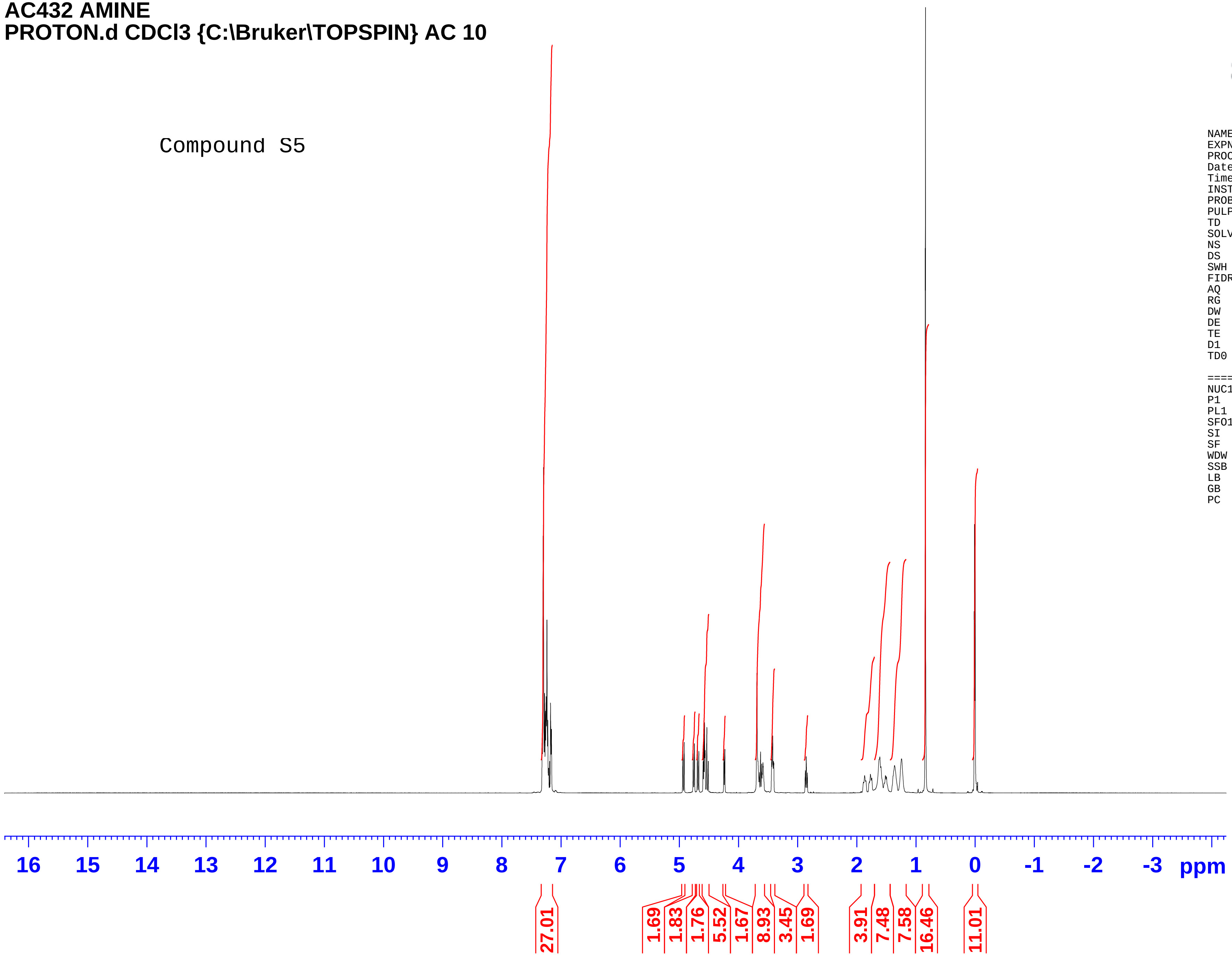

UNIVERSITY OF DUNDEE

NAME	AC-AC432NH2	
EXPNO	7	
PROCNO	1	
Date_	20111014	
Time	11.23	
INSTRUM	spect	
PROBHD	5 mm QNP 1H/13	
PULPROG	zg30	
TD	65536	
SOLVENT	CDCl3	
NS	16	
DS	2	
SWH	10330.578	Hz
FIDRES	0.157632	Hz
AQ	3.1719923	sec
RG	50.8	
DW	48.400	usec
DE	6.00	usec
TE	294.4	K
D1	1.00000000	sec
TD0	1	

======== CHANNEL f1 ========

NUC1	1H	
P1	11.20	usec
PL1	-1.00	dB
SFO1	500.1330885	MHz
SI	65536	
SF	500.1300465	MHz
WDW	EM	
SSB	0	
LB	0.30	Hz
GB	0	
PC	1.40	

AC432 BOC
C13CPDfast.d CDCl3 {C:\Bruker\TOPSPIN} AC 14

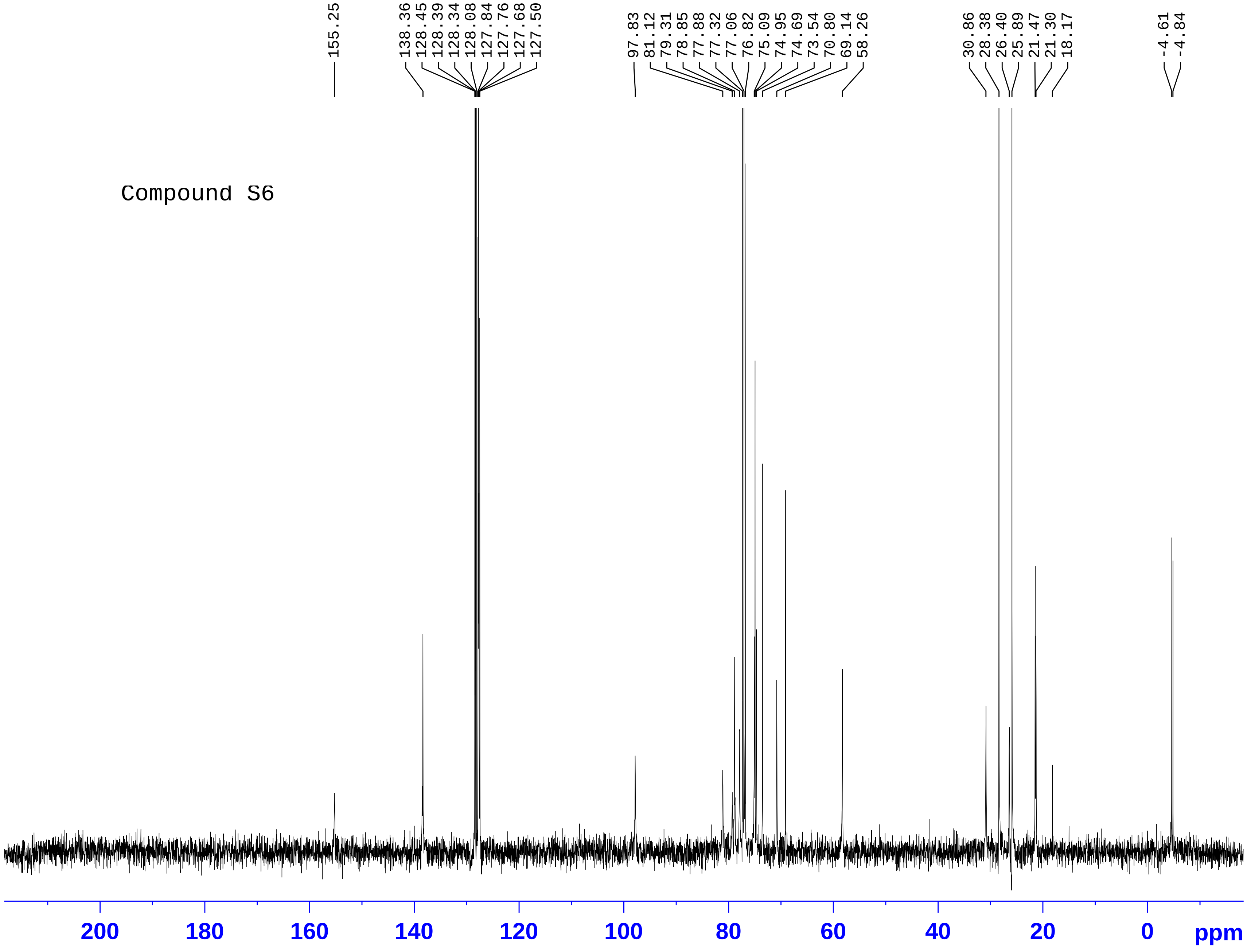

DUNDEE

NAME	AC-AC432BOC
EXPNO	8
PROCNO	1
Date_	20111024
Time	15.15
INSTRUM	spect
PROBHD	5 mm QNP 1H/13
PULPROG	zgpg30
TD	16384
SOLVENT	CDCl3
NS	800
DS	4
SWH	29761.904 Hz
FIDRES	1.816522 Hz
AQ	0.2753012 sec
RG	2050
DW	16.800 usec
DE	6.00 usec
TE	295.1 K
D1	0.30000001 sec
d11	0.03000000 sec
DELTA	0.20000002 sec
TD0	1

======== CHANNEL f1 ========

NUC1	13C
P1	8.18 usec
PL1	0.00 dB
SFO1	125.7703643 MHz

======== CHANNEL f2 ========

CPDPRG2	waltz16
NUC2	1H
PCPD2	80.00 usec
PL2	-1.00 dB
PL12	16.00 dB
PL13	16.00 dB
SFO2	500.1320005 MHz
SI	8192
SF	125.7577890 MHz
WDW	EM
SSB	0
LB	1.00 Hz
GB	0
PC	1.40

AC432BOC
PROTON.d CDCl3 {C:\Bruker\TOPSPIN} AC 18

Compound S6

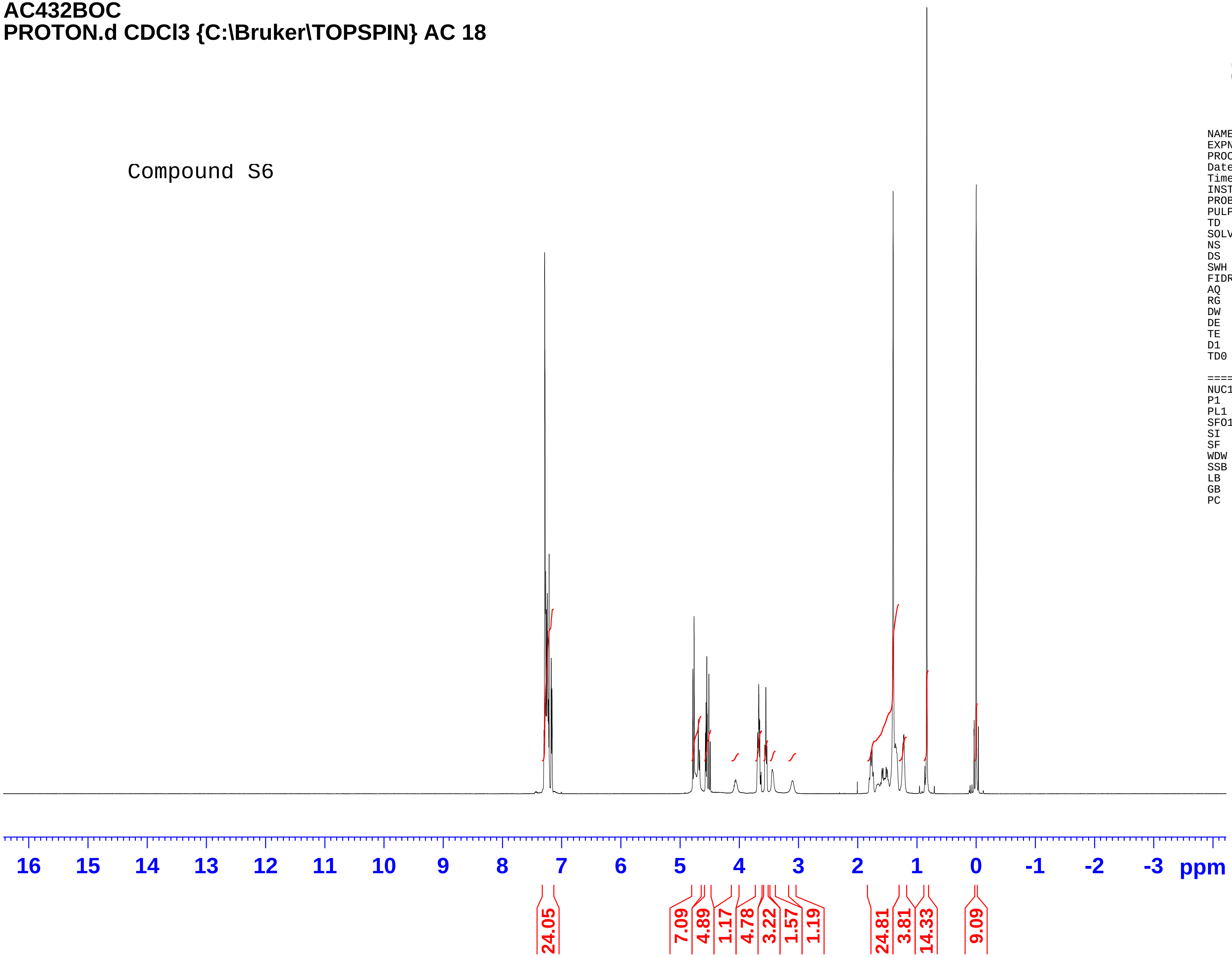

UNIVERSITY OF DUNDEE

NAME	AC-AC432BOC
EXPNO	1
PROCNO	1
Date_	20110721
Time	9.54
INSTRUM	spect
PROBHD	5 mm QNP 1H/13
PULPROG	zg30
TD	65536
SOLVENT	CDCl3
NS	16
DS	2
SWH	10330.578 Hz
FIDRES	0.157632 Hz
AQ	3.1719923 sec
RG	101
DW	48.400 usec
DE	6.00 usec
TE	294.3 K
D1	1.00000000 sec
TD0	1

======== CHANNEL f1 ========

NUC1	1H
P1	11.20 usec
PL1	-1.00 dB
SFO1	500.1330885 MHz
SI	65536
SF	500.1300363 MHz
WDW	EM
SSB	0
LB	0.30 Hz
GB	0
PC	1.40

AC440C AFTER PUR.
C13CPDfast.d CDCl3 {C:\Bruker\TOPSPIN} AC 16

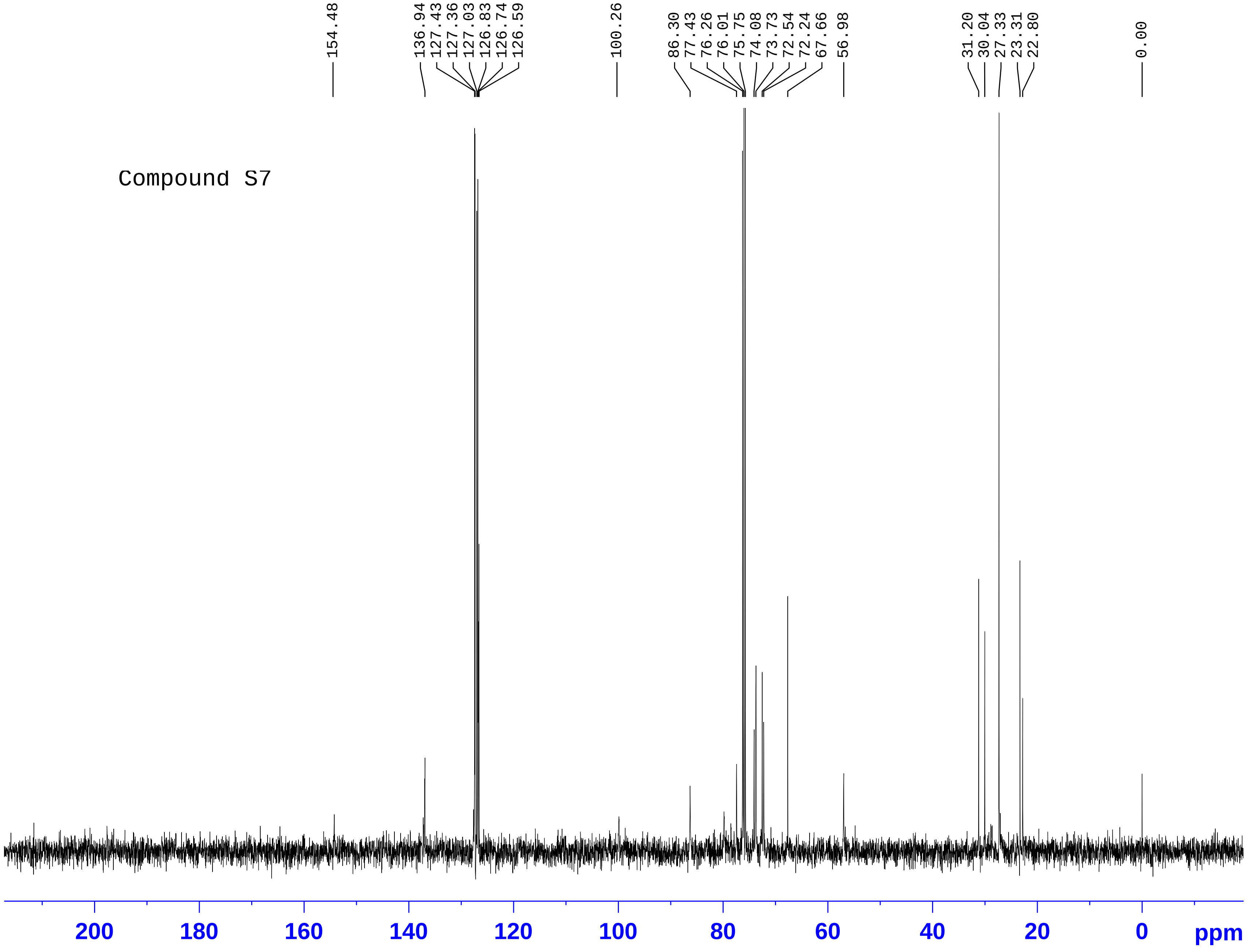

UNIVERSITY OF DUNDEE

NAME	AC-AC440C	
EXPNO	5	
PROCNO	1	
Date_	20111004	
Time	9.59	
INSTRUM	spect	
PROBHD	5 mm QNP 1H/13	
PULPROG	zgpg30	
TD	16384	
SOLVENT	CDCl3	
NS	800	
DS	4	
SWH	29761.904	Hz
FIDRES	1.816522	Hz
AQ	0.2753012	sec
RG	2050	
DW	16.800	usec
DE	6.00	usec
TE	295.4	K
D1	0.30000001	sec
d11	0.03000000	sec
DELTA	0.20000002	sec
TD0	1	

======== CHANNEL f1 ========

NUC1	13C	
P1	8.18	usec
PL1	0.00	dB
SFO1	125.7703643	MHz

======== CHANNEL f2 ========

CPDPRG2	waltz16	
NUC2	1H	
PCPD2	80.00	usec
PL2	-1.00	dB
PL12	16.00	dB
PL13	16.00	dB
SFO2	500.1320005	MHz
SI	8192	
SF	125.7579211	MHz
WDW	EM	
SSB	0	
LB	1.00	Hz
GB	0	
PC	1.40	

AC440C AFTER PUR.
PROTON.d CDCl3 {C:\Bruker\TOPSPIN} AC 16

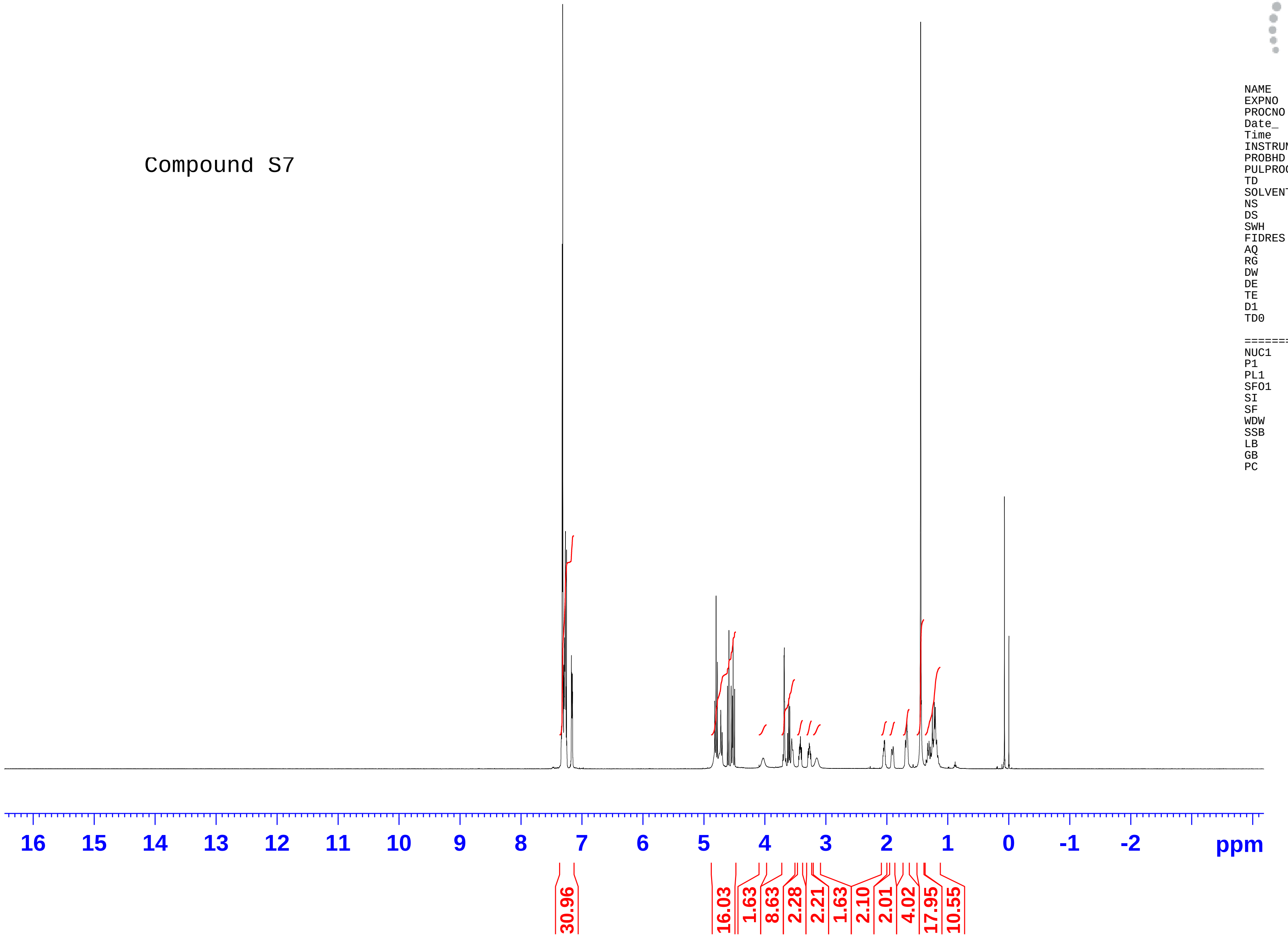

UNIVERSITY OF DUNDEE

```
NAME              AC-AC440C
EXPNO                     3
PROCNO                    1
Date_              20111004
Time                   9.39
INSTRUM               spect
PROBHD    5 mm QNP 1H/13
PULPROG                zg30
TD                    65536
SOLVENT               CDCl3
NS                       16
DS                        2
SWH           10330.578 Hz
FIDRES         0.157632 Hz
AQ            3.1719923 sec
RG                      203
DW               48.400 usec
DE                 6.00 usec
TE                294.4 K
D1           1.00000000 sec
TD0                       1

======== CHANNEL f1 ========
NUC1                     1H
P1                11.20 usec
PL1               -1.00 dB
SFO1        500.1330885 MHz
SI                    65536
SF          500.1300158 MHz
WDW                      EM
SSB                       0
LB                  0.30 Hz
GB                        0
PC                     1.40
```

AC444
C13CPDfast.d CDCl3 {C:\Bruker\TOPSPIN} AC 22

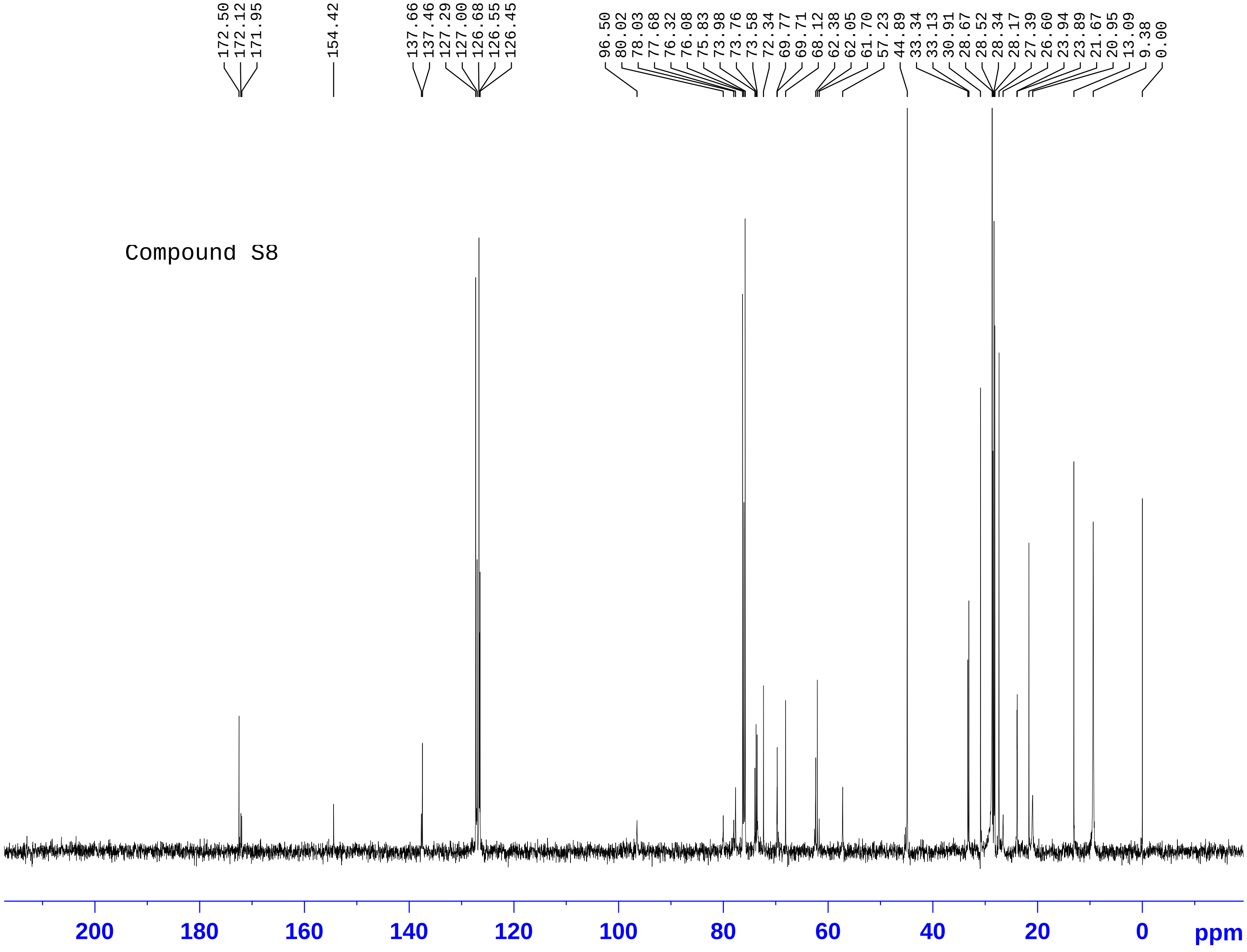

NAME	AC-AC444
EXPNO	5
PROCNO	1
Date_	20111104
Time	15.00
INSTRUM	spect
PROBHD	5 mm QNP 1H/13
PULPROG	zgpg30
TD	16384
SOLVENT	CDCl3
NS	800
DS	4
SWH	29761.904 Hz
FIDRES	1.816522 Hz
AQ	0.2753012 sec
RG	2050
DW	16.800 usec
DE	6.00 usec
TE	300.2 K
D1	0.30000001 sec
d11	0.03000000 sec
DELTA	0.20000002 sec
TD0	1

======== CHANNEL f1 ========

NUC1	13C
P1	8.18 usec
PL1	0.00 dB
SFO1	125.7703643 MHz

======== CHANNEL f2 ========

CPDPRG2	waltz16
NUC2	1H
PCPD2	80.00 usec
PL2	-1.00 dB
PL12	16.00 dB
PL13	16.00 dB
SFO2	500.1320005 MHz
SI	8192
SF	125.7579140 MHz
WDW	EM
SSB	0
LB	1.00 Hz
GB	0
PC	1.40

AC444
PROTON.d CDCl3 {C:\Bruker\TOPSPIN} AC 22

Compound S8

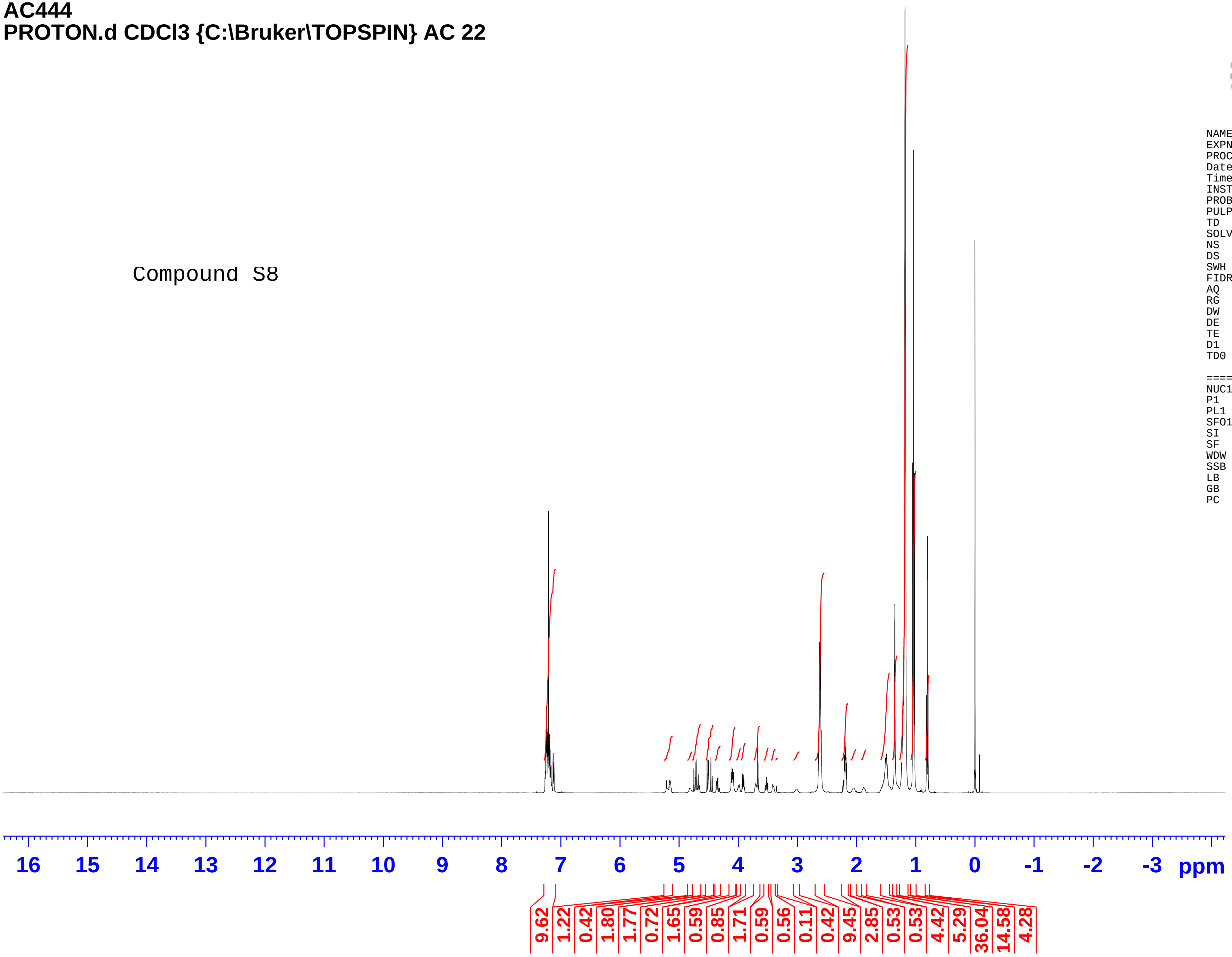

UNIVERSITY OF DUNDEE

NAME	AC-AC444
EXPNO	3
PROCNO	1
Date_	20111104
Time	14.40
INSTRUM	spect
PROBHD	5 mm QNP 1H/13
PULPROG	zg30
TD	65536
SOLVENT	CDCl3
NS	16
DS	2
SWH	10330.578 Hz
FIDRES	0.157632 Hz
AQ	3.1719923 sec
RG	45.2
DW	48.400 usec
DE	6.00 usec
TE	300.2 K
D1	1.00000000 sec
TD0	1

======== CHANNEL f1 ========

NUC1	1H
P1	11.20 usec
PL1	-1.00 dB
SFO1	500.1330885 MHz
SI	65536
SF	500.1300396 MHz
WDW	EM
SSB	0
LB	0.30 Hz
GB	0
PC	1.40

AC445C
C13CPDlong.d MeOD {C:\Bruker\TOPSPIN} AC 26

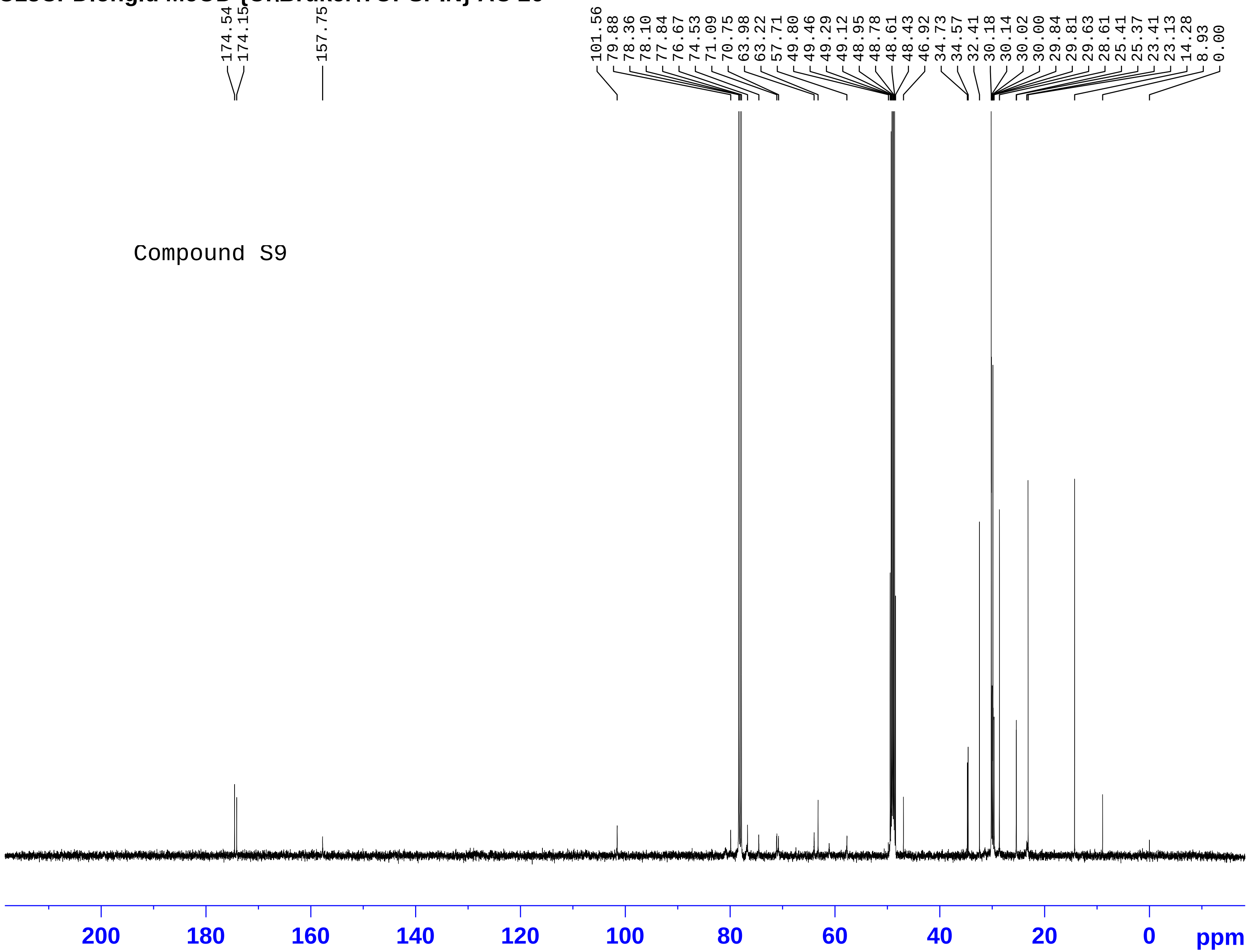

UNIVERSITY OF DUNDEE

NAME	AC-AC445C
EXPNO	8
PROCNO	1
Date_	20111122
Time	2.14
INSTRUM	spect
PROBHD	5 mm QNP 1H/13
PULPROG	zgpg30
TD	65536
SOLVENT	MeOD
NS	2048
DS	4
SWH	29761.904 Hz
FIDRES	0.454131 Hz
AQ	1.1010548 sec
RG	2050
DW	16.800 usec
DE	6.00 usec
TE	295.5 K
D1	2.00000000 sec
d11	0.03000000 sec
DELTA	1.89999998 sec
TD0	8

======== CHANNEL f1 ========

NUC1	13C
P1	8.18 usec
PL1	0.00 dB
SFO1	125.7703643 MHz

======== CHANNEL f2 ========

CPDPRG2	waltz16
NUC2	1H
PCPD2	80.00 usec
PL2	-1.00 dB
PL12	16.00 dB
PL13	16.00 dB
SFO2	500.1320005 MHz
SI	32768
SF	125.7577812 MHz
WDW	EM
SSB	0
LB	1.00 Hz
GB	0
PC	1.40

AC445
PROTON.d MeOD {C:\Bruker\TOPSPIN} AC 20

Compound S9

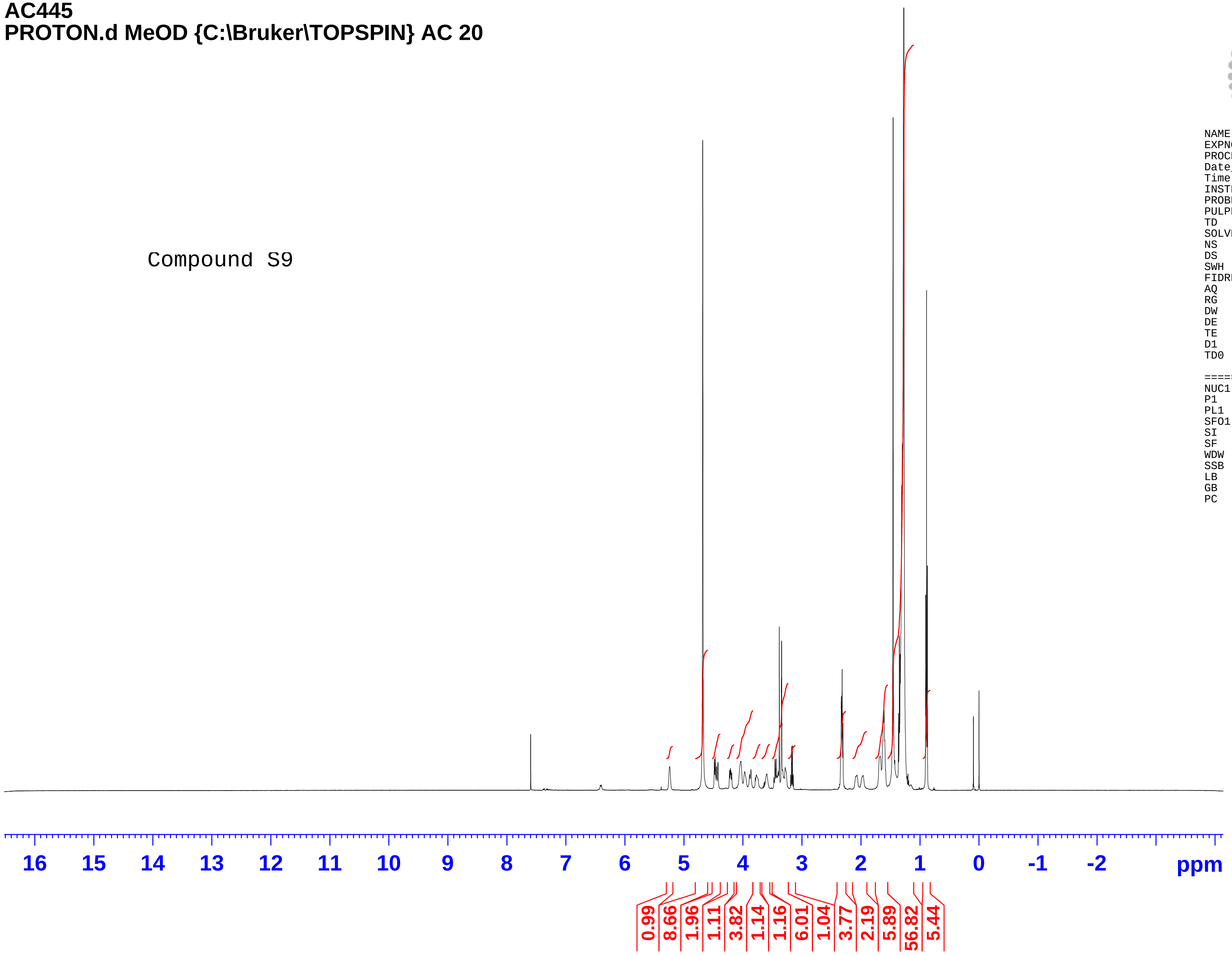

UNIVERSITY OF DUNDEE

NAME	AC-AC445C
EXPNO	1
PROCNO	1
Date_	20111121
Time	14.45
INSTRUM	spect
PROBHD	5 mm QNP 1H/13
PULPROG	zg30
TD	65536
SOLVENT	MeOD
NS	16
DS	2
SWH	10330.578 Hz
FIDRES	0.157632 Hz
AQ	3.1719923 sec
RG	144
DW	48.400 usec
DE	6.00 usec
TE	294.4 K
D1	1.00000000 sec
TD0	1

======== CHANNEL f1 ========

NUC1	1H
P1	11.20 usec
PL1	-1.00 dB
SFO1	500.1330885 MHz
SI	65536
SF	500.1299932 MHz
WDW	EM
SSB	0
LB	0.30 Hz
GB	0
PC	1.40
